# Supplementary material for: Practical and stereoselective electrocatalytic 1,2-diamination of alkenes
Source: Nat Commun. 2019 Oct 31;10:4953. doi: 10.1038/s41467-019-13024-5 (PMC6823458; doi:10.1038/s41467-019-13024-5)
Supplement: Supplementary file 2 — Supplementary Information [file 41467_2019_13024_MOESM2_ESM.pdf]

Practical and stereoselective electrocatalytic 1,2-diamination of alkenes

Chen-Yan Cai, Xiao-Min Shu & Hai-Chao Xu

Supplementary Methods

General considerations

The commercially available reagents were used without purification. Flash column chromatography was performed with silica gel (200–300 mesh). Cyclic voltammograms were recorded on a CHI 760E potentiostat. NMR spectra were recorded on Bruker AV-400, Bruker AV-500 and Bruker AV-600 instruments. Data were reported as chemical shifts in ppm relative to TMS (0.00 ppm) for ^{1}H and $CDCl_3$ (77.2 ppm), acetone-d_6 (30.6 ppm) for ^{13}C. The abbreviations used for explaining the multiplicities were as follows: s = singlet, d = doublet, t = triplet, q = quartet, m = multiplet, br = broad. Infrared spectra were recorded on a Nicolet AVATER FTIR330 spectrometer. High resolution mass spectra (ESI) were recorded by the instrumentation center of Department of Chemistry, Xiamen University, on a Micromass QTOF2 Quadruple/Time-of-Flight Tandem mass spectrometer. The reticulated vitreous carbon (100 pores per inch) can be obtained from Goodfellow.

Alkenes employed for the diamination reaction

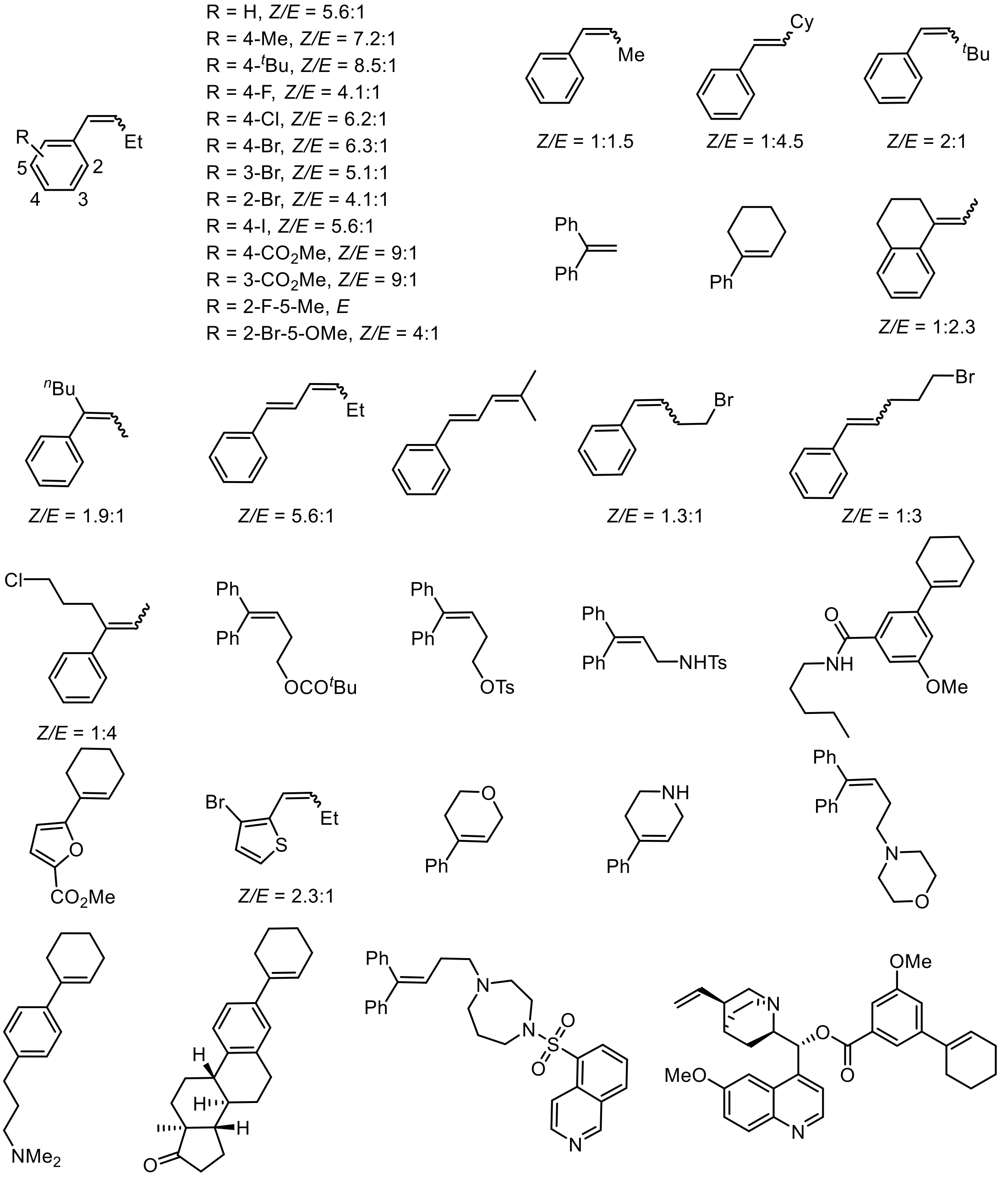

Supplementary Figure 1. Alkenes employed for the diamination reaction.

Synthesis and characterization of new substrates

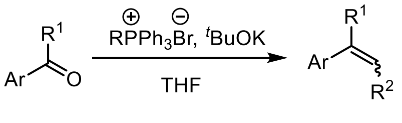

General procedure A for the synthesis of alkenes: To a suspension of triphenylphosphonium bromide (1.5 equiv) in THF at 0 °C was added ^{t}BuOK (1.5 equiv). The reaction was warmed to ambient temperature and stirred for 0.5 h. The ketone or

aldehyde (1.0 equiv) was added. The resulting mixture was stirred at rt until complete consumption of the carbonyl substrate (monitored by TLC or ^{1}H NMR). H_2O (20 mL) was added to quench the reaction. The resulting mixture was extracted with Et_2O (2 x 50 mL). The combined organic solution was dried over $MgSO_4$, filtered, and concentrated under reduced pressure. The residue was chromatographed through silica gel eluting with EtOAc/hexanes to afford the alkene.

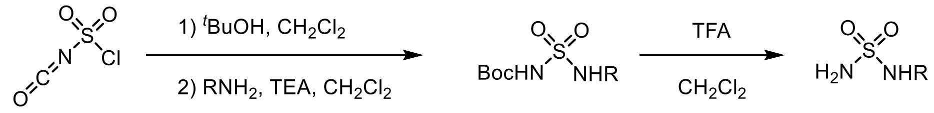

General procedure B for the synthesis of sulfamides[1]: *N*-chlorosulfonyl (*tert*-butyl) carbamate was prepared by addition of ^{t}BuOH (1.4 equiv) into a solution of chlorosulfonyl isocyanate (1 equiv) in CH_2Cl_2. The newly prepared reagent was slowly added at 0 °C into a solution containing a primary amine (1 equiv) and triethylamine (1.1 equiv) in CH_2Cl_2. The reaction mixture was stirred for 45–60 min at 0 °C and then 2–3 h at RT. The reaction was diluted with CH_2Cl_2, washed with HC1 (0.1 N) twice, dried over anhydrous Na_2SO_4, filtered and concentrated under reduced pressure. The residue was purified by column chromatography followed by recrystallization to give the *N*-Boc sulfamide. A solution of TFA (50% in CH_2Cl_2, 5 equiv) was added dropwise into a stirred solution of *N*-Boc-sulfamide (1 equiv) in CH_2Cl_2 at 0 °C. The reaction mixture was stirred for 2 h at 0 °C and then at rt for 12 h. The reaction mixture was concentrated under reduced pressure. The residue was chromatographed through silica gel to afford the final sulfamide.

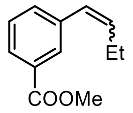

Methyl 3-(but-1-en-1-yl)benzoate (S1). The title compound was obtained as a colorless oil in 74% yield (0.70 g) starting from 2-bromo-5-methoxybenzaldehyde (0.82 g, 5.0 mmol) and propyltriphenylphosphonium bromide (2.9 g, 7.5 mmol) by following the general procedure A. *Z*:*E* = 9:1; ^{1}H NMR (500 MHz, $CDCl_3$) δ 8.05–8.01 (m, 0.1H), 7.97–7.93 (m, 0.9H), 7.92–7.87 (m, 0.9H), 7.87–7.83 (m, 0.1H), 7.53–7.49 (m, 0.1H), 7.48–7.43 (m, 0.9H), 7.42–7.33 (m, 1H), 6.47–6.31 (m, 1.1H), 5.72 (dt, J = 11.6, 7.3 Hz, 0.9H), 3.92 (s, 3H), 2.38–2.30 (m, 1.8H), 2.29–2.21 (m, 0.2H), 1.12–1.05 (m, 3H); ^{13}C NMR (126 MHz, $CDCl_3$) δ 167.3, 138.2, 136.0, 134.2, 133.3, 130.5, 130.2, 130.0, 128.7, 128.3, 128.1, 127.9, 127.7, 127.5, 127.2, 52.3 (2C), 26.2, 22.1, 14.5, 13.7; IR (neat, cm^{-1}): 2963, 1725, 1278, 765.

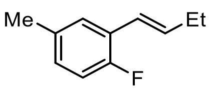

(*E*)-2-(But-1-en-1-yl)-1-fluoro-4-methylbenzene (S2). The title compound was obtained as a colorless oil in 72% yield (0.59 g) starting from 2-fluoro-5-methylbenzaldehyde (0.69 g, 5.0 mmol) and propyltriphenylphosphonium bromide (2.9 g, 7.5 mmol) by following the general procedure A. ^{1}H NMR (500 MHz, $CDCl_3$) δ 7.24–7.17 (m, 1H), 6.97–6.90 (m, 1H), 6.87 (dd, *J* = 10.4, 8.3 Hz, 1H), 6.49 (dt, *J* = 16.0, 1.7 Hz, 1H), 6.31 (dt, *J* = 16.0, 6.5 Hz, 1H), 2.33–2.19 (m, 5H), 1.09 (t, *J* = 7.5 Hz, 3H); ^{13}C NMR (126 MHz, $CDCl_3$) δ 158.4 (d, $J_{C\text{-}F}$ = 245.4 Hz), 135.1 (d, $J_{C\text{-}F}$ = 4.4 Hz), 133.4 (d, $J_{C\text{-}F}$ = 3.5 Hz), 128.6 (d, $J_{C\text{-}F}$ = 8.1 Hz), 127.5 (d, $J_{C\text{-}F}$ = 3.9 Hz), 125.3 (d, $J_{C\text{-}F}$ = 12.6 Hz), 121.5 (d, $J_{C\text{-}F}$ = 3.5 Hz), 115.4 (d, $J_{C\text{-}F}$ = 22.3 Hz), 26.6, 20.9, 13.7; ^{19}F NMR (471 MHz, $CDCl_3$) δ −124.3; IR (neat, cm^{-1}): 2965, 1493, 1210, 810.

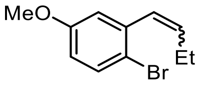

1-Bromo-2-(but-1-en-1-yl)-4-methoxybenzene (S3). The title compound was obtained as a colorless oil in 64% yield (0.78 g) starting from 2-bromo-5-methoxybenzaldehyde (1.1 g, 5.0 mmol) and propyltriphenylphosphonium bromide (2.9 g, 7.5 mmol) by following the general procedure A. *Z:E* = 4:1; ^{1}H NMR (500 MHz, $CDCl_3$) δ 7.44 (d, *J* = 8.8 Hz, 0.8H), 7.39 (d, *J* = 8.7 Hz, 0.2H), 7.02 (d, *J* = 3.1 Hz, 0.2H), 6.82 (d, *J* = 3.1 Hz, 0.8H), 6.70–6.60 (m, 1.2H), 6.38 (dt, *J* = 11.4, 1.8 Hz, 0.8H), 6.20 (dt, *J* = 15.7, 6.5 Hz, 0.2H), 5.75 (dt, *J* = 11.5, 7.4 Hz, 0.8H), 3.81–3.75 (m, 3H), 2.30–2.17 (m, 2H), 1.11 (t, *J* = 7.5 Hz, 0.6H), 1.03 (t, *J* = 7.5 Hz, 2.4H); ^{13}C NMR (126 MHz, $CDCl_3$) δ 159.1, 158.5, 138.6, 135.9 (2C), 133.5, 133.2, 128.0 (2C), 116.4, 114.7, 114.5, 114.1, 114.0, 112.1, 55.6, 26.3, 22.0, 14.4, 13.6; IR (neat, cm^{-1}): 2963, 1566, 1463, 1231, 1017.

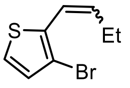

3-Bromo-2-(but-1-en-1-yl)thiophene (S4). The title compound was obtained as a colorless oil in 85% yield (0.67 g) starting from 3-bromothiophene-2-carbaldehyde (0.95 g, 5.0 mmol) and propyltriphenylphosphonium bromide (2.9 g, 7.5 mmol) by following the general procedure A. *Z:E* = 2.3:1. ^{1}H NMR (500 MHz, $CDCl_3$) δ 7.27–7.22 (m, 0.7H), 7.05 (d, *J* = 5.3 Hz, 0.3H), 6.98 (d, *J* = 5.3 Hz, 0.7H), 6.90 (d, *J* = 5.4 Hz, 0.3H), 6.59–6.51 (m, 1H), 6.18 (dt, *J* = 15.8, 6.6 Hz, 0.3H), 5.71 (dt, *J* = 11.6, 7.3 Hz, 0.7H), 2.47–2.35 (m, 1.4H), 2.26–2.19 (m, 0.6H), 1.16–1.06 (m, 3H); ^{13}C NMR (126 MHz, $CDCl_3$) δ 137.5, 135.2 (2C), 134.6, 130.6, 130.0, 124.9, 123.0, 120.8, 119.9, 111.2, 108.8, 26.3, 22.8, 14.0, 13.5; IR (neat, cm^{-1}): 3396, 2919, 1384, 1086, 711; ESI HRMS *m/z* $(M+Na)^+$ calcd 238.9501, obsd 238.9502.

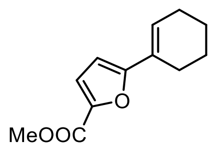

Methyl 5-(cyclohex-1-en-1-yl)furan-2-carboxylate (S5). The title compound was prepared by following the procedure described for the synthesis of **S12.** Light yellow oil; Yield = 98%; ^{1}H NMR (500 MHz, $CDCl_3$) δ 7.14 (d, *J* = 3.6 Hz, 1H), 6.56 (tt, *J* = 3.9, 1.7 Hz, 1H), 6.25 (d, *J* = 3.6 Hz, 1H), 3.87 (s, 3H), 2.34–2.28 (m, 2H), 2.25–2.18 (m, 2H), 1.78–1.71 (m, 2H), 1.65 (dtt, *J* = 10.1, 7.1, 3.1 Hz, 2H); ^{13}C NMR (126 MHz, $CDCl_3$) δ 159.5, 159.4, 142.6, 127.3, 126.8, 119.9, 105.6, 51.9, 25.5, 24.9, 22.3, 22.1; IR (neat, cm^{-1}): 3478, 2944, 1728, 1305, 763; ESI HRMS *m/z* $(M+Na)^+$ calcd 229.0835, obsd 229.0834.

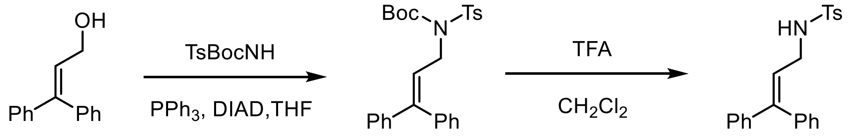

S6 **S7** **S8**

***tert*-Butyl (3,3-diphenylallyl)(tosyl)carbamate (S7).** Diisopropyl azodicarboxylate (DIAD, 0.76 mL, 4.3 mmol, 1.3 equiv) was added dropwise to a solution of **S6**[2] (0.69 g, 3.3 mmol, 1.0 equiv), *N*-(*tert*-butoxycarbonyl)-*p*-toluenesulfonamide (1.2 g, 4.3 mmol, 1.3 equiv) and PPh_3 (1.1 g, 4.3 mmol, 1.3 equiv) in THF (30 mL) at 0 °C. The reaction mixture was stirred at rt for 12 h and then concentrated under reduced pressure. The residue was chromatographed through silica gel eluting with EtOAc/hexanes to give the title compound as a white solid (0.81 g, 53%). ^{1}H NMR (500 MHz, $CDCl_3$) δ 7.85–7.73 (m, 2H), 7.44–7.38 (m, 2H), 7.38–7.32 (m, 1H), 7.32–7.20 (m, 9H), 6.09 (t, *J* = 6.0 Hz, 1H), 4.55 (d, *J* = 6.0 Hz, 2H), 2.43 (s, 3H), 1.33 (s, 9H); ^{13}C NMR (126 MHz, $CDCl_3$) δ 151.0, 144.3, 143.9, 141.8, 139.0, 137.6, 130.0, 129.4, 128.5, 128.4, 128.2, 127.8, 127.7, 127.6, 125.0, 84.4, 46.5, 28.1, 21.8; IR (neat, cm^{-1}): 3296, 2981, 1729, 1357, 1154; ESI HRMS *m/z* $(M+Na)^+$ calcd 486.1710, obsd 486.1718.

***N*-(3,3-Diphenylallyl)-4-methylbenzenesulfonamide (S8).** TFA (0.57 mL, 7.5 mmol, 5.0 equiv) was added dropwise to a solution of **S7** (0.69 g, 1.5 mmol, 1.0 equiv) in CH_2Cl_2 (5 mL) at RT. The reaction was stirred for 2 h and quenched with 1 N $NaHCO_3$. The reaction mixture was extracted with CH_2Cl_2 (3 x 10 mL). The combined organic solution was dried over anhydrous Na_2SO_4, filtered and evaporated under reduced pressure. The residue was purified by silica gel column chromatography to afford the product **S8** as a white solid (0.44 g, 80%). ^{1}H NMR (500 MHz, $CDCl_3$) δ 7.69 (d, *J* = 8.2 Hz, 2H), 7.35–7.28 (m, 3H), 7.28–7.20 (m, 5H), 7.09–7.01 (m, 4H), 5.91 (t, *J* = 7.1 Hz, 1H), 4.57 (t, *J* = 6.0 Hz, 1H), 3.66 (dd, *J* = 7.1, 6.0 Hz, 2H), 2.41 (s, 3H); ^{13}C NMR (126 MHz, $CDCl_3$) δ 145.6, 143.6, 141.4, 138.6, 137.3, 129.9, 129.6, 128.6, 128.3, 127.9,

127.8, 127.6, 127.3, 123.4, 42.6, 21.7; IR (neat, cm^{-1}): 3284, 3056, 1323, 1159, 702; ESI HRMS *m/z* $(M+Na)^+$ calcd 386.1185, obsd 386.1186.

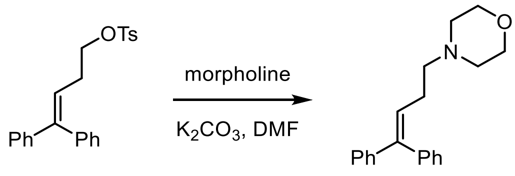

S9 **S10**

4-(4,4-Diphenylbut-3-en-1-yl)morpholine (S10). **S9**[2] (0.76 g, 2.0 mmol, 1.0 equiv) was dissolved in DMF (15 mL) and treated with K_2CO_3 (0.33 g, 2.4 mmol, 1.2 equiv) and then morpholine (0.35 mL, 4.0 mmol, 2.0 equiv). The reaction mixture was heated to 65 °C and stirred at that temperature for 3 h. The reaction mixture was poured into ice water and extracted with EtOAc (2 x 10 mL). The combined EtOAc layers were washed with water (3 x 10 mL) and brine, dried over anhydrous Na_2SO_4, filtered and concentrated. The residue was purified by silica gel column chromatography to afford the product **S10** as a light yellow oil (0.43 g, 73%). ^{1}H NMR (500 MHz, $CDCl_3$) δ 7.39–7.33 (m, 2H), 7.33–7.28 (m, 1H), 7.28–7.23 (m, 2H), 7.23–7.19 (m, 3H), 7.19–7.15 (m, 2H), 6.08 (t, *J* = 7.3 Hz, 1H), 3.68 (d, *J* = 4.6 Hz, 4H), 2.46 (t, *J* = 7.6 Hz, 2H), 2.39 (t, *J* = 4.6 Hz, 4H), 2.35–2.27 (m, 2H); ^{13}C NMR (126 MHz, $CDCl_3$) δ 142.9, 142.7, 140.2, 130.0, 128.4, 128.3, 127.4, 127.2, 127.2, 127.1, 67.2, 59.0, 53.8, 27.4; IR (neat, cm^{-1}): 2957, 2805, 1444, 1118, 701; ESI HRMS *m/z* $(M+H)^+$ calcd 294.1852, obsd 294.1856.

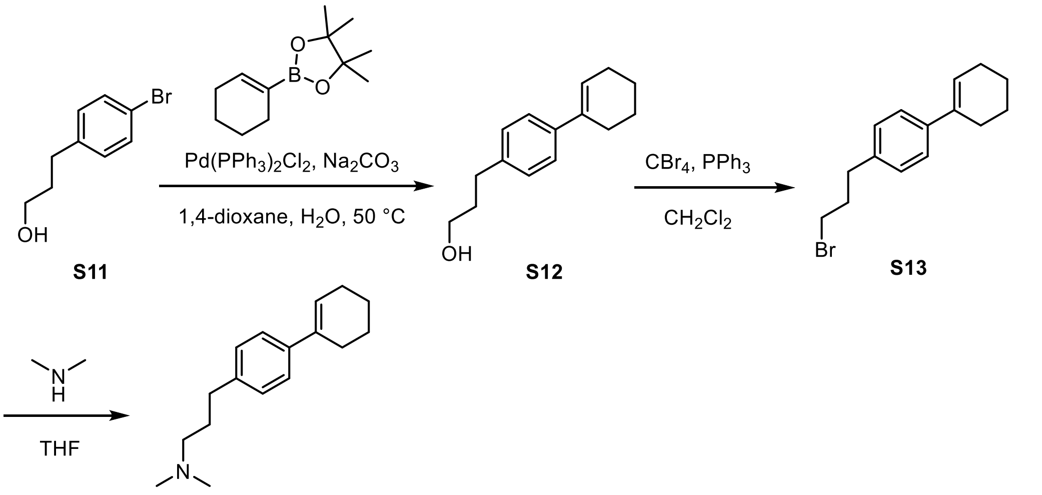

S11 **S12** **S13** **S14**

3-(2',3',4',5'-Tetrahydro-[1,1'-biphenyl]-4-yl)propan-1-ol (S12). 2-(cyclohex-1-en-1-yl)-4,4,5,5-tetramethyl-1,3,2-dioxaborolane (3.12 g, 15.0 mmol, 1.5 equiv), **S11** (2.15 g, 10.0 mmol, 1.0 equiv), $Pd(PPh_3)_2Cl_2$ (0.60 g, 0.50 mmol, 0.05 equiv) and Na_2CO_3 (4.20 g, 40.0 mmol, 4.0 equiv) were dissolved in a mixture of 1,4-dioxane/H_2O (2.5:1, 28 mL). The resulting mixture was deoxygenated with a stream of argon for 10 min, then

heated to 50 °C until completion of the reaction (monitored by ^{1}H NMR). The mixture was cooled down to rt and quenched with saturated NH_4Cl (10 mL). The reaction mixture was extracted with EtOAc (3 x 25 mL). The combined organic solution was washed with H_2O, brine, dried over anhydrous Na_2SO_4, filtered and evaporated under reduced pressure. The residue was purified by silica gel column chromatography to give the desired **S12** as a light yellow oil (1.69 g, 78%). ^{1}H NMR (500 MHz, $CDCl_3$) δ 7.33–7.27 (m, 2H), 7.16–7.10 (m, 2H), 6.15–6.02 (m, 1H), 3.66 (t, *J* = 6.4 Hz, 2H), 2.68 (dd, *J* = 8.7, 6.7 Hz, 2H), 2.39 (tq, *J* = 6.3, 2.3 Hz, 2H), 2.19 (tq, *J* = 6.2, 2.6 Hz, 2H), 1.93–1.83 (m, 2H), 1.81–1.74 (m, 2H), 1.68–1.61 (m, 2H), 1.48 (brs, 1H); ^{13}C NMR (126 MHz, $CDCl_3$) δ 140.5, 140.2, 136.4, 128.4, 125.1, 124.3, 62.4, 34.3, 31.8, 27.5, 26.0, 23.2, 22.4; IR (neat, cm^{-1}): 3347, 2928, 1437, 1058, 796; ESI HRMS *m/z* $(M+Na)^+$ calcd 239.1406, obsd 239.1410.

4'-(3-Bromopropyl)-2,3,4,5-tetrahydro-1,1'-biphenyl (S13). To a vigorously stirred solution of **S12** (1.08 g, 5.00 mmol, 1.00 equiv) and CBr_4 (1.82 g, 5.50 mmol, 1.10 equiv) in CH_2Cl_2 (30 mL) was added PPh_3 (1.38 g, 5.75 mmol, 1.05 equiv) in portions at 0 °C. The mixture was stirred 12 h and then concentrated under reduced pressure. To the residue was added 75 mL of Et_2O. The suspension was then filtered and washed with Et_2O (3 x 50 mL). The filtrate was concentrated under reduced pressure. The residue was purified by silica gel column chromatography to give the desired **S13** as a colorless oil (1.07 g, 78%). ^{1}H NMR (500 MHz, $CDCl_3$) δ 7.35–7.29 (m, 2H), 7.17–7.11 (m, 2H), 6.13–6.06 (m, 1H), 3.39 (t, *J* = 6.6 Hz, 2H), 2.75 (t, *J* = 7.3 Hz, 2H), 2.45–2.35 (m, 2H), 2.25–2.11 (m, 4H), 1.83–1.73 (m, 2H), 1.71–1.60 (m, 2H); ^{13}C NMR (126 MHz, $CDCl_3$) δ 140.8, 138.9, 136.4, 128.6, 125.2, 124.5, 34.3, 33.7, 33.3, 27.6, 26.1, 23.3, 22.4; IR (neat, cm^{-1}): 3355, 2919, 1651, 1384, 700; ESI HRMS *m/z* $(M+Na)^+$ calcd 301.0562, obsd 301.0577.

***N,N*-Dimethyl-3-(2',3',4',5'-tetrahydro-[1,1'-biphenyl]-4-yl)propan-1-amine (S14).** To a solution of dimethylamine (2 M in THF, 26 mL, 26 equiv) was added **S13** (0.56 g, 2.0 mmol, 1.0 equiv). The resulting mixture was stirred at rt for 12 h before being poured into water. The mixture was extracted with EtOAc (2 x 10 mL). The combined EtOAc solution were washed with water (3 x 10 mL) and brine, dried over anhydrous Na_2SO_4, filtered and concentrated. The residue was purified by silica gel column chromatography to afford the product **S14** as a light yellow oil (0.38 g, 78%). ^{1}H NMR (500 MHz, $CDCl_3$) δ 7.32–7.28 (m, 2H), 7.15–7.10 (m, 2H), 6.13–6.06 (m, 1H), 2.66–2.58 (m, 2H), 2.43–2.36 (m, 2H), 2.35–2.29 (m, 2H), 2.24 (s, 6H), 2.19 (ddt, *J* = 8.8, 6.3, 3.6 Hz, 2H), 1.84–1.73 (m, 4H), 1.70–1.61 (m, 2H); ^{13}C NMR (126 MHz, $CDCl_3$) δ 140.6, 140.4, 136.5, 128.4, 125.0, 124.3, 59.4, 45.6, 33.4, 29.5, 27.6, 26.0, 23.3, 22.4; IR (neat, cm^{-1}): 2931, 2856, 1457, 1137, 822; ESI HRMS *m/z* $(M+H)^+$ calcd 244.2060, obsd 244.2063.

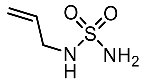

***N*-2-Propen-1-ylsulfamide (S15).** The title compound was obtained as a brown oil in 78% yield (1.36 g) by following the general procedure B. ^{1}H NMR (500 MHz, acetone-d_6) δ 5.94 (ddt, J = 17.2, 10.3, 5.8 Hz, 1H), 5.86 (s, 2H), 5.77 (s, 1H), 5.26 (dq, J = 17.2, 1.7 Hz, 1H), 5.10 (dq, J = 10.3, 1.5 Hz, 1H), 3.69 (tt, J = 5.9, 1.6 Hz, 2H); ^{13}C NMR (126 MHz, acetone-d_6) δ 136.5, 117.3, 47.4; IR (neat, cm^{-1}): 3288, 1557, 1430, 1328, 1159, 544.

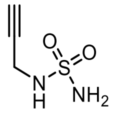

***N*-2-Propyn-1-ylsulfamide (S16).** The title compound was obtained as a light yellow oil in 80% yield (0.58 g) by following the general procedure B. ^{1}H NMR (500 MHz, acetone-d_6) δ 6.08 (d, J = 6.4 Hz, 1H), 5.96 (s, 2H), 3.87 (dd, J = 6.2, 2.5 Hz, 2H), 2.76 (t, J = 2.5 Hz, 1H); ^{13}C NMR (126 MHz, acetone-d_6) δ 81.4, 74.3, 34.1; IR (neat, cm^{-1}): 3285, 1333, 1159, 922.

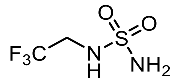

(2,2,2-Trifluoroethyl)sulfamide (S17). The title compound was obtained as a white solid in 39% yield (1.66 g) by following the general procedure B. ^{1}H NMR (500 MHz, acetone-d_6) δ 6.63 (t, J = 7.7 Hz, 1H), 6.19 (brs, 2H), 3.78 (qd, J = 9.3, 7.1 Hz, 2H); ^{13}C NMR (126 MHz, acetone-d_6) δ 126.3 (q, J = 277.2 Hz), 46.0 (q, J = 34.6 Hz); ^{19}F NMR (471 MHz, acetone-d_6) δ −73.0; IR (neat, cm^{-1}): 3288, 1336, 1158, 663, 541.

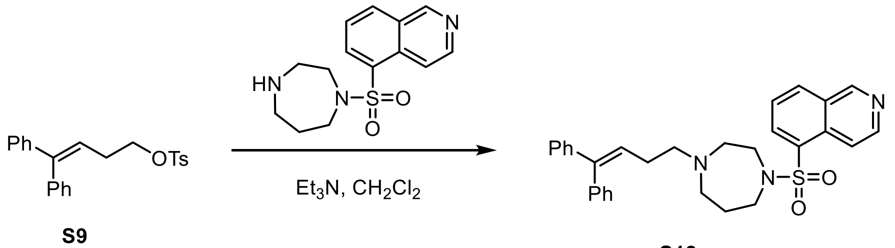

5-((4-(4,4-Diphenylbut-3-en-1-yl)-1,4-diazepan-1-yl)sulfonyl)isoquinoline (S18). Fasudil (0.17 g, 0.60 mmol, 1.0 equiv) was dissolved in CH_2Cl_2 (2 mL). Et_3N (100 μL, 0.72 mmol, 1.2 equiv) was added followed by **S9**[2] (0.24 g, 0.60 equiv, 1.0 equiv). The reaction mixture was stirred for 2 days and concentrated under reduced pressure. The residue was chromatographed through silica gel to afford **S18** as a colorless oil (0.15 g, 50%). ^{1}H NMR (500 MHz, $CDCl_3$) δ 9.33 (d, J = 0.9 Hz, 1H), 8.67 (d, J = 6.1 Hz, 1H), 8.45 (d, J = 6.1 Hz, 1H), 8.32 (dd, J = 7.3, 1.2 Hz, 1H), 8.16 (d, J = 8.2 Hz, 1H), 7.68–

7.62 (m, 1H), 7.35 (dd, J = 8.1, 6.5 Hz, 2H), 7.32–7.27 (m, 1H), 7.25–7.16 (m, 5H), 7.16–7.12 (m, 2H), 6.02 (t, J = 7.4 Hz, 1H), 3.49–3.42 (m, 4H), 2.67–2.63 (m, 2H), 2.63–2.60 (m, 2H), 2.60–2.55 (m, 2H), 2.27–2.19 (m, 2H), 1.83–1.75 (m, 2H); ^{13}C NMR (126 MHz, $CDCl_3$) δ 153.4, 145.2, 142.9, 142.6, 140.2, 134.8, 133.4, 133.0, 131.8, 129.9, 129.4, 128.4, 128.3, 127.3, 127.2 (2C), 127.1, 126.0, 117.8, 57.8, 56.1, 54.2, 48.6, 47.0, 28.4, 28.1; IR (neat, cm^{-1}): 3443, 1636, 1327, 1157, 1136, 761, 703, 591; ESI HRMS *m/z* $(M+H)^+$ calcd 498.2210, obsd 498.2209.

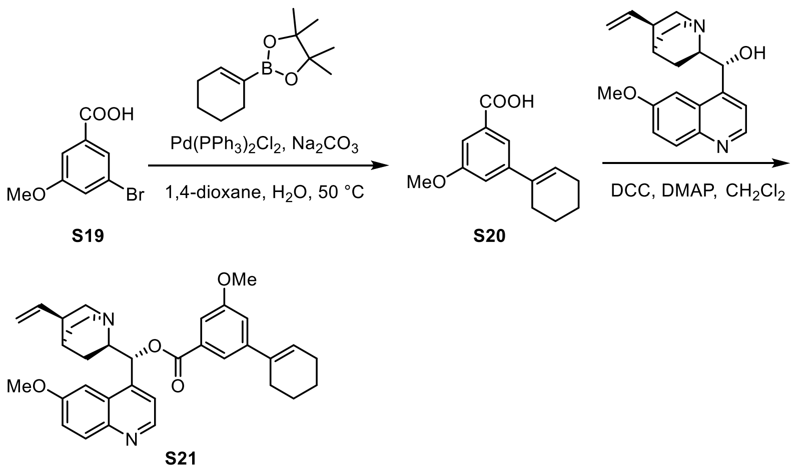

5-Methoxy-2',3',4',5'-tetrahydro-[1,1'-biphenyl]-3-carboxylic acid (S20). The title compound was prepared by following the procedure described for the synthesis of **S12**. White solid; Yield = 52%; ^{1}H NMR (500 MHz, $CDCl_3$) δ 7.76 (d, J = 1.5 Hz, 1H), 7.48 (dd, J = 2.7, 1.3 Hz, 1H), 7.19–7.14 (m, 1H), 6.25–6.18 (m, 1H), 3.87 (s, 3H), 2.44–2.39 (m, 2H), 2.26–2.18 (m, 2H), 1.83–1.76 (m, 2H), 1.71–1.63 (m, 2H); ^{13}C NMR (126 MHz, $CDCl_3$) δ 172.3, 159.8, 144.7, 135.8, 130.4, 126.5, 119.9, 117.4, 112.4, 55.7, 27.6, 26.0, 23.1, 22.2; IR (neat, cm^{-1}): 2929, 2856, 1691, 1590, 1457, 1410, 1329, 1200, 883, 685; ESI HRMS *m/z* $(M+Na)^+$ calcd 255.0992, obsd 255.0996.

(*R*)-(6-Methoxyquinolin-4-yl)((1*S*,2*R*,4*S*,5*R*)-5-vinylquinuclidin-2-yl)methyl 5-methoxy-2',3',4',5'-tetrahydro-[1,1'-biphenyl]-3-carboxylate (S21). Quinine (0.65 g, 2.0 mmol, 1.0 equiv), **S20** (0.45 mg, 2.0 mmol, 1.0 equiv) and DMAP (0.02 g, 0.20 mmol, 0.10 equiv) were dissolved in CH_2Cl_2 (6 mL). The reaction mixture was cooled to 0 °C and treated with a solution of dicyclohexylcarbodiimide (0.45 g, 2.2 mmol, 1.1 equiv) in CH_2Cl_2 (4 mL). The resulting reaction mixture allowed to warm to rt and stirred for 24 h. The reaction mixture was filtered and concentrated. The residue was chromatographed through silica gel to afford **S21** as a white foamy solid (0.16 g, 15%). ^{1}H NMR (500 MHz, $CDCl_3$) δ 8.72 (d, J = 4.5 Hz, 1H), 8.02 (d, J = 9.1 Hz, 1H), 7.72 (s, 1H), 7.52 (d, J = 2.7 Hz, 1H), 7.46 (s, 1H), 7.42 (d, J = 4.4 Hz, 1H), 7.38 (dd, J = 9.2, 2.6 Hz, 1H), 7.13 (s, 1H), 6.72 (d, J = 6.5 Hz, 1H), 6.21–6.12 (m, 1H), 5.84 (ddd, J = 17.5, 10.3, 7.4 Hz, 1H), 5.07–4.93 (m, 2H), 3.98 (s, 3H), 3.85 (s, 3H), 3.55–3.43 (m, 1H),

3.29–3.18 (m, 1H), 3.10 (dd, J = 13.9, 10.1 Hz, 1H), 2.76–2.62 (m, 2H), 2.45–2.35 (m, 2H), 2.34–2.27 (m, 1H), 2.26–2.18 (m, 2H), 2.02–1.91 (m, 2H), 1.84–1.75 (m, 3H), 1.71–1.64 (m, 3H), 1.63–1.54 (m, 1H); ^{13}C NMR (126 MHz, $CDCl_3$) δ 165.9, 159.9, 158.2, 147.7, 145.0, 144.9, 143.8, 141.9, 135.9, 132.0, 130.9, 127.1, 126.6, 122.0, 119.2, 118.9, 116.4, 114.7, 112.6, 101.7, 75.0, 59.5, 57.0, 55.8, 55.7, 42.8, 39.9, 28.1, 27.9, 27.6, 26.0, 24.3, 23.1, 22.2; IR (neat, cm^{-1}): 3447, 2932, 1623, 1311, 1227, 1030, 641; ESI HRMS *m/z* $(M+H)^+$ calcd 539.2904, obsd 539.2904.

Procedures for the electrolysis

Note: The surface area of RVC (100 PPI) is reported to be about 65 cm^2 cm^{-3}. The scale-up reactions employed larger electrodes, which allows the use of higher constant current to ensure good productivity.

The procedure for the small-scale synthesis has been provided in the manuscript. The assembling of the cell is detailed below.

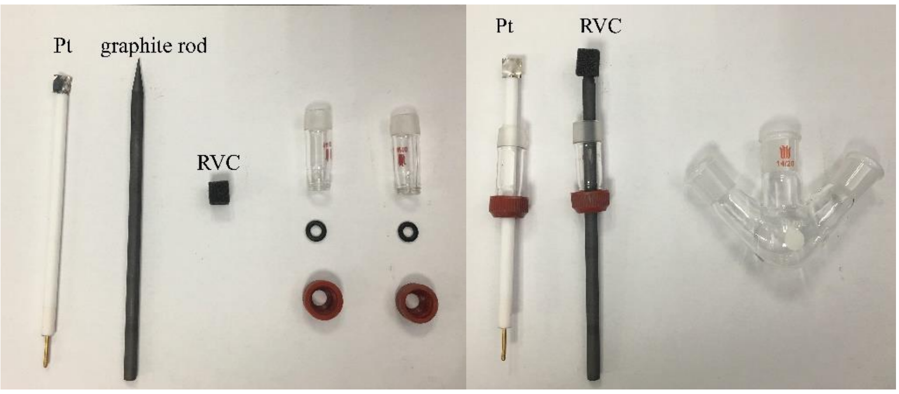

Supplementary Figure 2. Electrodes and reaction flask for small scale electrolysis.

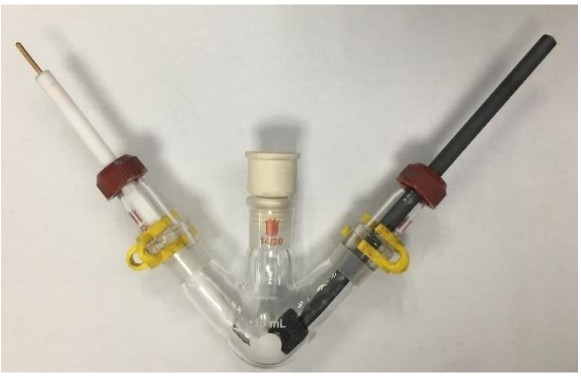

Supplementary Figure 3. Reaction setup for small scale electrolysis.

The decagram scale synthesis of **22** was conducted in a 1-L beaker-type cell with two pieces of RVC (5 cm x 8 cm x 1.2 cm) as the anode, a Pt plate cathode (5 cm x 5 cm x 0.1 mm), and a constant current of 1,000 mA. The three electrodes were placed in parallel with the Pt plate cathode sandwiched between two RVC anodes. The reaction mixture consisted **62** (8.38 g, 45.0 mmol), **4** (8.65 g, 90.0 mmol), Et_4NPF_6 (12.39 g, 45.0 mmol), $^iPrCO_2H$ (8.37 mL, 90.0 mmol), **1** (3.21 g, 4.50 mmol), $BF_3{\cdot}Et_2O$ (2.90 mL, 22.5 mmol), MeCN (300 mL) and CH_2Cl_2 (600 mL). The reaction time was 2.4 h.

The gram scale synthesis of **48** was conducted in a 200-mL beaker-type cell with two pieces of RVC (2.5 cm x 4.5 cm x 1.2 cm) as the anode, a Pt plate cathode (2 cm x 2 cm x 0.1 mm), and a constant current of 281 mA. The three electrodes were placed in parallel

with the Pt plate cathode sandwiched between two RVC anodes. The reaction mixture consisted **62** (0.84 g, 4.5 mmol), **63** (3.73 g, 27.0 mmol), Et_4NPF_6 (1.24 g, 4.50 mmol), $^iPrCO_2H$ (0.84 mL, 9.0 mmol), **1** (0.32 g, 0.45 mmol), $BF_3{\cdot}Et_2O$ (0.59 mL, 4.5 mmol), MeCN (45 mL) and CH_2Cl_2 (90 mL). The reaction time was 1.1 h.

The gram scale synthesis of **59** was conducted in a 200-mL beaker-type cell with two pieces of RVC (3.5 cm x 4 cm x 1.2 cm) as the anode, a Pt plate cathode (2 cm x 2 cm x 0.1 mm), and a constant current of 350 mA. The three electrodes were placed in parallel with the Pt plate cathode sandwiched between two RVC anodes. The reaction mixture consisted **62** (1.02 g, 5.50 mmol), **64** (4.49 g, 33.0 mmol), Et_4NPF_6 (1.51 g, 5.50 mmol), $^iPrCO_2H$ (1.02 mL, 11.0 mmol), **1** (0.39 g, 0.55 mmol), $BF_3{\cdot}Et_2O$ (0.72 mL, 5.50 mmol), MeCN (55 mL) and CH_2Cl_2 (110 mL). The reaction time was 0.9 h.

The 300 mmol scale synthesis of **21** was conducted in a 2-L beaker-type cell with two pieces of RVC (5 cm x 10 cm x 1.2 cm) as the anode, a Pt plate cathode (5 cm x 5 cm x 0.1 mm), and a constant current of 1,000 mA. The three electrodes were placed in parallel with the Pt plate cathode sandwiched between two RVC anodes. The electrolytic cell was linked with a 5-L glass bottle as a reservoir for reaction mixture. The continuous circulation of solution between the cell and the reservoir was achieved by a peristaltic pump through Teflon catheter with a flow rate 17.5 mL min^{-1}. The solution in electrolytic cell consisted **61** (12.9 mL, 100 mmol), **4** (57.6 g, 600 mmol), Et_4NPF_6 (27.5 g, 100 mmol), $^iPrCO_2H$ (18.6 mL, 200 mmol), **1** (7.1 g, 10 mmol), $BF_3{\cdot}Et_2O$ (6.5 mL, 50 mmol), MeCN (0.66 L) and CH_2Cl_2 (1.34 L). The solution in 5-L glass bottle consisted **61** (25.9 mL, 200 mmol), Et_4NPF_6 (55.1 g, 200 mmol), $^iPrCO_2H$ (37.2 mL, 400 mmol), **1** (14.3 g, 20 mmol), $BF_3{\cdot}Et_2O$ (13.0 mL, 100 mmol), MeCN (1.33 L) and CH_2Cl_2 (2.67 L). The reaction time was 16.5 h.

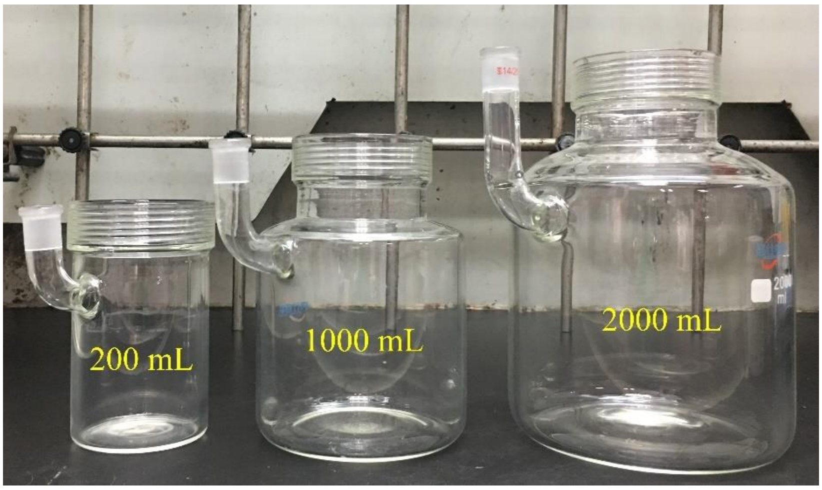

Supplementary Figure 4. Cells employed for the gram scale electrolysis.

The 300 mmol scale electrolysis was conducted using the following setup.

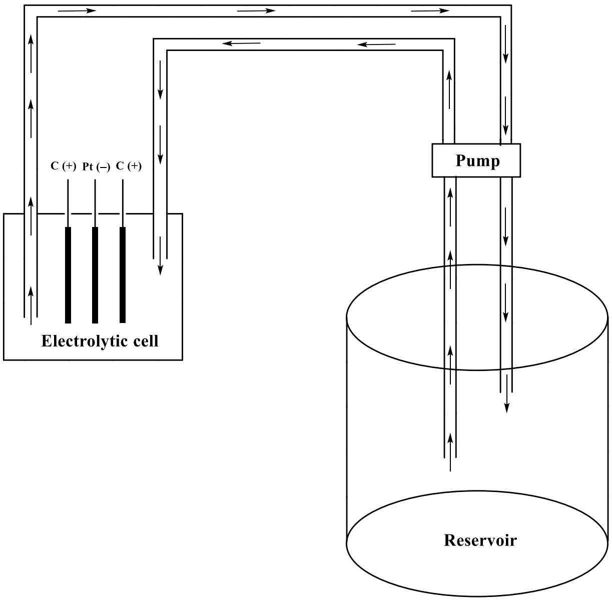

Supplementary Figure 5. Schematic representation of reaction setup for 300 mmol scale electrolysis.

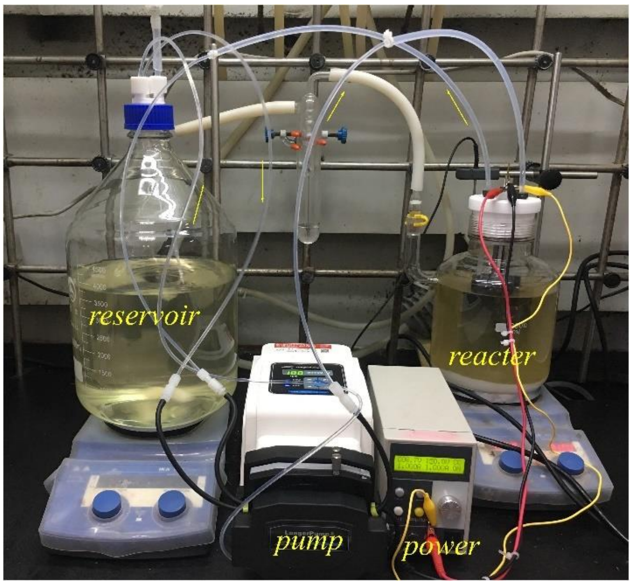

Supplementary Figure 6. Reaction setup for 300 mmol scale electrolysis.

The assembling of electrolysis setup for the 300 mmol scale reaction is detailed below.

1. Cut a piece of reticulated vitreous carbon (10 cm x 10 cm x 1.2 cm) in half.

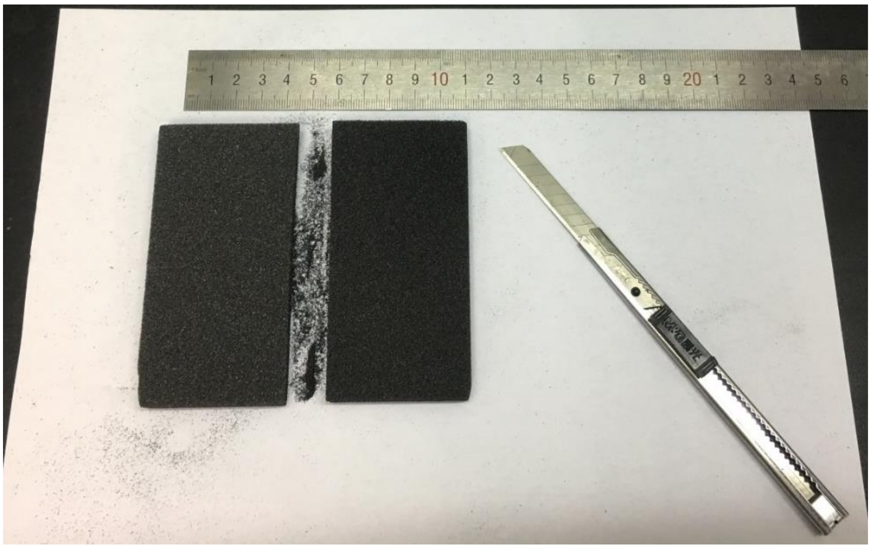

Supplementary Figure 7. Preparation of RVC.

2. Sharpen two graphite rods (∅ 6 mm) with a pencil sharpener. Insert the graphite rod in the top of RVC.

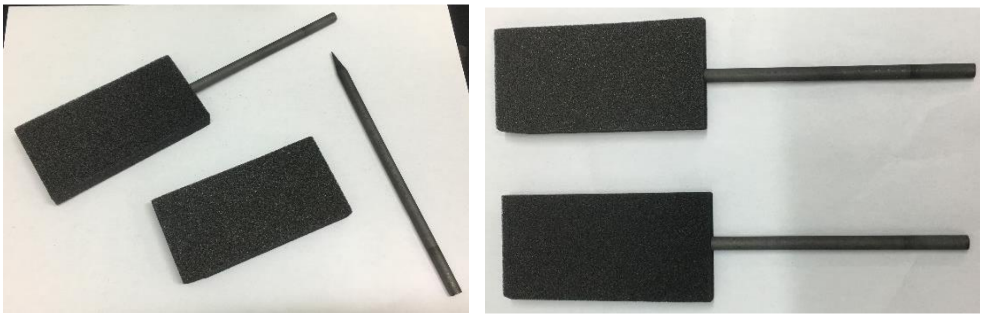

Supplementary Figure 8. Preparation of RVC electrodes.

3. Insert the electrodes into the hole of the cap. The three electrodes were placed in parallel with the Pt plate cathode sandwiched between two RVC anodes.

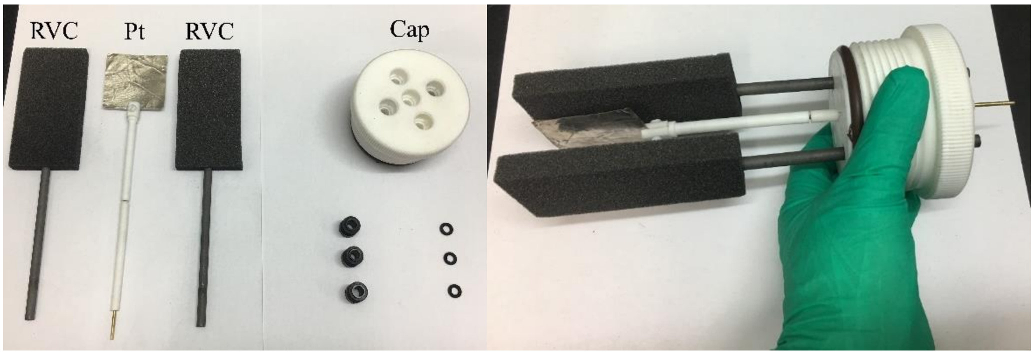

Supplementary Figure 9. Assembling of electrodes.

4. Put the cap with electrodes onto the cell.

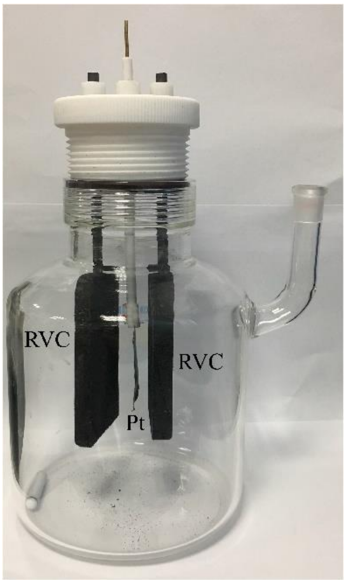

Supplementary Figure 10. The electrolysis cell.

5. Link two catheters together and bundle them with cable ties.

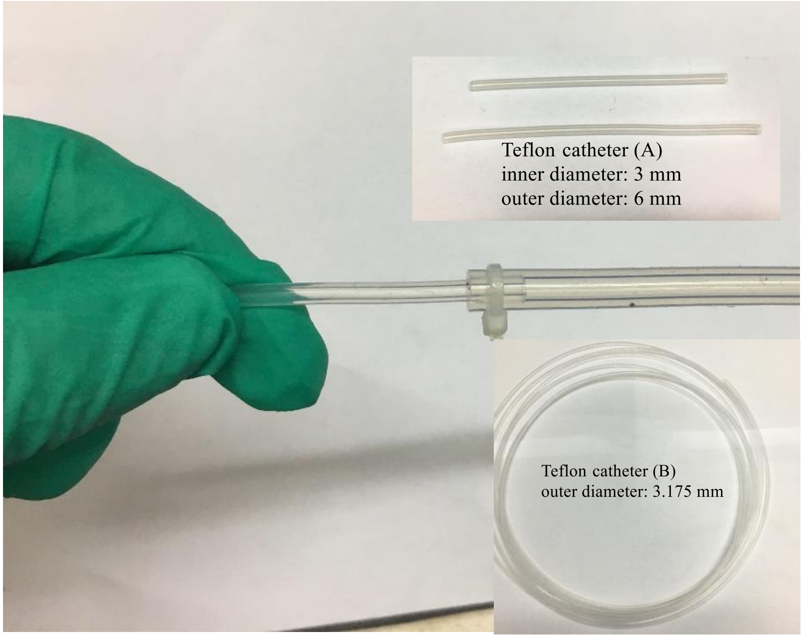

Supplementary Figure 11. Preparation of pumping tube.

6. Link a syringe filter with catheter B using a syringe. Insert to the bottom of catheter A. This home-made filter could prevent clogging of catheter.

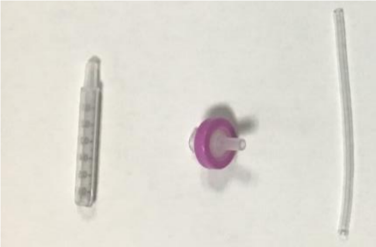

Supplementary Figure 12. Components for the filter.

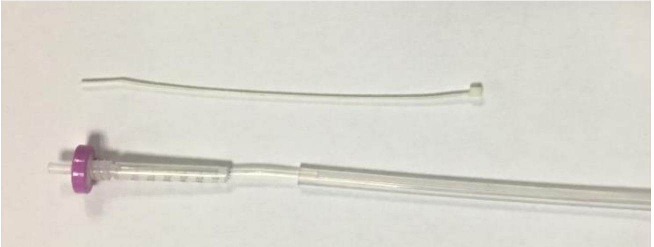

Supplementary Figure 13. Preparation of filter.

7. Insert the catheter into the hole of the cell cap.

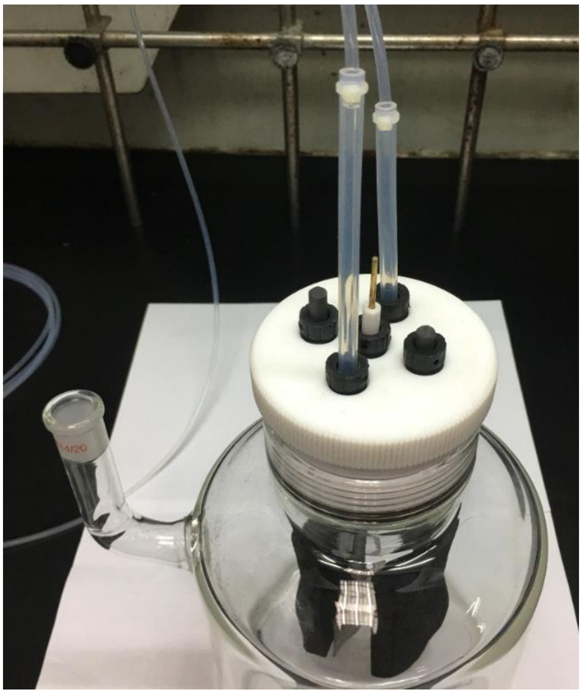

Supplementary Figure 14. The electrolysis cell equipped with pumping tube.

8. Insert catheter B to the cap of reservoir. Install a non-return valve on the hole of the cap to balance the pressure inside the reservoir.

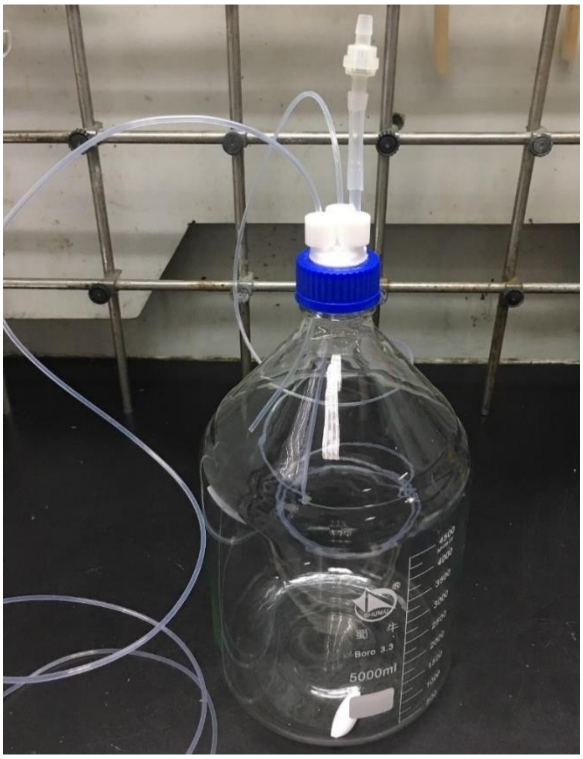

Supplementary Figure 15. The reservoir.

9. Link the tubes on the electrolytic cell, reservoir, and peristaltic pump that contains two channels (Longer peristaltic pump BT100-2J, pump head DG15-28; Power supply: Rainworm CE0200010T).

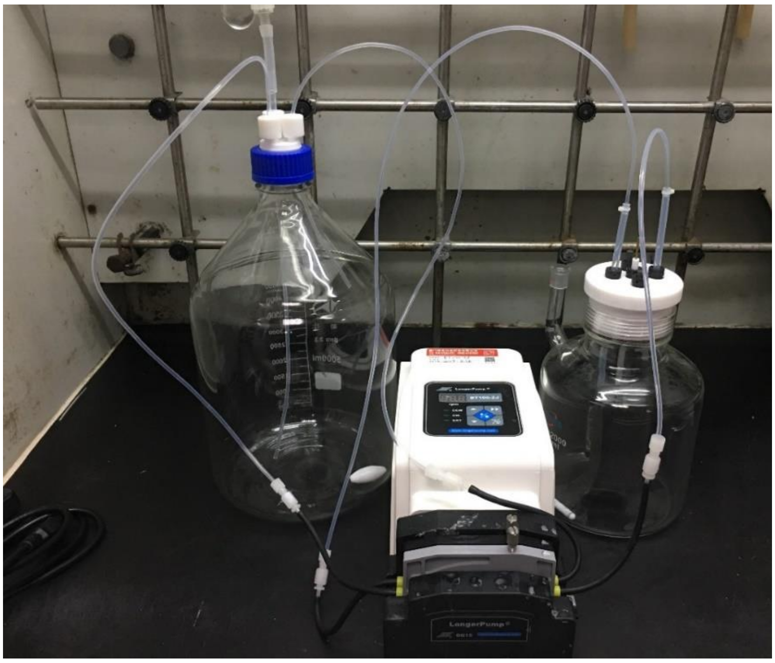

Supplementary Figure 16. Assembled setup.

Characterization data for the electrolysis products

Note: The consumed charge F mol^{-1} denotes Faraday per mol of the alkene substrate.

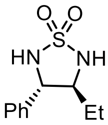

3-Ethyl-4-phenyl-1,2,5-thiadiazolidine 1,1-dioxide (5). The configuration was determined using NOE experiment. Light yellow oil; Yield = 72%; Electricity = 2.2 F mol^{-1}; ^{1}H NMR (500 MHz, acetone-d_6) δ 7.53–7.49 (m, 2H), 7.41–7.37 (m, 2H), 7.35–7.31 (m, 1H), 6.46 (d, J = 6.3 Hz, 1H), 6.15 (d, J = 8.3 Hz, 1H), 4.51 (t, J = 6.7 Hz, 1H), 3.54–3.49 (m, 1H), 1.77–1.71 (m, 2H), 0.97 (t, J = 7.4 Hz, 3H); ^{13}C NMR (126 MHz, acetone-d_6) δ 141.5, 130.2, 129.6, 128.8, 67.8, 67.6, 27.2, 12.2; IR (neat, cm^{-1}): 3258, 2933, 1303, 1165, 702; ESI HRMS *m/z* (M+Na)$^+$ calcd 249.0668, obsd 249.0674.

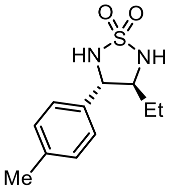

3-Ethyl-4-(*p*-tolyl)-1,2,5-thiadiazolidine 1,1-dioxide (9). Light yellow solid; Yield = 86%; Electricity = 2.5 F mol^{-1}; ^{1}H NMR (500 MHz, $CDCl_3$) δ 7.32–7.28 (m, 2H), 7.20 (d, J = 7.8 Hz, 2H), 4.64 (d, J = 5.9 Hz, 1H), 4.57 (d, J = 8.6 Hz, 1H), 4.43 (dd, J = 8.4, 5.9 Hz, 1H), 3.67–3.58 (m, 1H), 2.36 (s, 3H), 1.70 (ddq, J = 15.1, 7.6, 3.5 Hz, 1H), 1.63–1.56 (m, 1H), 0.96 (t, J = 7.4 Hz, 3H); ^{13}C NMR (126 MHz, $CDCl_3$) δ 139.2, 134.2, 130.0, 127.3, 67.1, 66.4, 25.4, 21.3, 11.1; IR (neat, cm^{-1}):3268, 2919, 1384, 1164; ESI HRMS *m/z* (M+Na)$^+$ calcd 263.0825, obsd 263.0830.

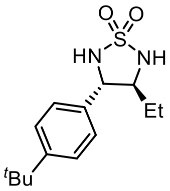

3-(4-(*tert*-Butyl)phenyl)-4-ethyl-1,2,5-thiadiazolidine 1,1-dioxide (10). Colorless oil; Yield = 65%; Electricity = 2.5 F mol^{-1}; ^{1}H NMR (500 MHz, acetone-d_6) δ 7.47–7.40 (m, 4H), 6.36 (d, J = 6.2 Hz, 1H), 6.12 (d, J = 8.4 Hz, 1H), 4.47 (dd, J = 7.4, 6.1 Hz, 1H), 3.54–3.50 (m, 1H), 1.76–1.69 (m, 2H), 1.32 (s, 9H), 0.97 (t, J = 7.4 Hz, 3H); ^{13}C NMR (126 MHz, acetone-d_6) δ 152.5, 138.3, 128.6, 127.0, 67.6, 67.5, 35.8, 32.4, 27.2, 12.2; IR (neat, cm^{-1}): 3258, 2965, 2871, 1301, 1166, 832; ESI HRMS *m/z* (M+Na)$^+$ calcd 305.1294, obsd 305.1298.

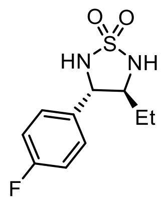

3-Ethyl-4-(4-fluorophenyl)-1,2,5-thiadiazolidine 1,1-dioxide (11). Light yellow solid; Yield = 76%; Electricity = 2.0 F mol^{-1}; ^{1}H NMR (500 MHz, acetone-d_6) δ 7.58–7.55 (m, 2H), 7.18–7.14 (m, 2H), 6.52 (d, J = 6.3 Hz, 1H), 6.17 (d, J = 8.2 Hz, 1H), 4.55 (t, J = 6.7 Hz, 1H), 3.51 (ddt, J = 8.2, 7.1, 5.4 Hz, 1H), 1.78–1.71 (m, 2H), 0.98 (t, J = 7.4 Hz, 3H); ^{13}C NMR (126 MHz, acetone-d_6) δ 164.1 (d, $J_{C\text{-}F}$ = 244.3 Hz), 137.7 (d, $J_{C\text{-}F}$ = 3.1 Hz), 130.8 (d, $J_{C\text{-}F}$ = 8.3 Hz), 116.9 (d, $J_{C\text{-}F}$ = 21.7 Hz), 67.6, 67.0, 27.2, 12.1; ^{19}F NMR (471 MHz, acetone-d_6) δ -116.0; IR (neat, cm^{-1}): 3259, 1512, 1158, 836; ESI HRMS *m/z* (M+Na)$^+$ calcd 267.0574, obsd 267.0582.

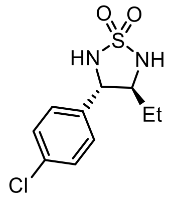

3-(4-Chlorophenyl)-4-ethyl-1,2,5-thiadiazolidine 1,1-dioxide (12). Light yellow solid; Yield = 72%; Electricity = 2.0 F mol^{-1}; ^{1}H NMR (500 MHz, acetone-d_6) δ 7.56–7.53 (m, 2H), 7.43–7.40 (m, 2H), 6.56 (d, J = 6.3 Hz, 1H), 6.19 (d, J = 8.2 Hz, 1H), 4.56 (t, J = 6.7 Hz, 1H), 3.50 (ddt, J = 8.3, 6.8, 5.2 Hz, 1H), 1.80–1.73 (m, 2H), 0.98 (t, J = 7.4 Hz, 3H); ^{13}C NMR (126 MHz, acetone-d_6) δ 140.7, 134.8, 130.5, 130.2, 67.5, 66.9, 27.3, 12.1; IR (neat, cm^{-1}): 3250, 2927, 1162, 827; ESI HRMS *m/z* (M+Na)$^+$ calcd 283. 0278, obsd 283.0287.

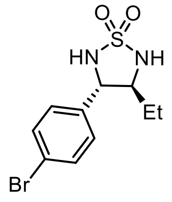

3-(4-Bromophenyl)-4-ethyl-1,2,5-thiadiazolidine 1,1-dioxide (13). White solid; Yield = 70%; Electricity = 2.3 F mol^{-1}; ^{1}H NMR (500 MHz, acetone-d_6) δ 7.58–7.55 (m, 2H), 7.50–7.48 (m, 2H), 6.55 (d, J = 6.3 Hz, 1H), 6.18 (d, J = 8.3 Hz, 1H), 4.55 (t, J = 6.5 Hz, 1H), 3.50 (ddt, J = 8.2, 6.7, 5.1 Hz, 1H), 1.80–1.73 (m, 2H), 0.98 (t, J = 7.4 Hz, 3H); ^{13}C NMR (126 MHz, acetone-d_6) δ 141.2, 133.2, 130.9, 123.0, 67.5, 67.0, 27.4, 12.1; IR (neat, cm^{-1}): 3254, 2926, 1164, 1010, 822; ESI HRMS *m/z* (M+Na)$^+$ calcd 326.9773, obsd 326.9782.

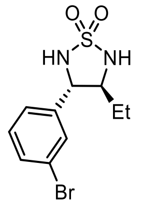

3-(3-Bromophenyl)-4-ethyl-1,2,5-thiadiazolidine 1,1-dioxide (14). Light yellow oil; Yield = 61%; Electricity = 2.5 F mol^{-1}; ^{1}H NMR (500 MHz, acetone-d_6) δ 7.75–7.74 (m, 1H), 7.53–7.50 (m, 2H), 7.37–7.34 (m, 1H), 6.61 (d, J = 6.3 Hz, 1H), 6.23 (d, J = 8.2 Hz, 1H), 4.57 (dd, J = 6.4 Hz, 1H), 3.52 (tdd, J = 8.5, 6.5, 5.0 Hz, 1H), 1.83–1.76 (m, 2H), 1.00 (t, J = 7.4 Hz, 3H); ^{13}C NMR (126 MHz, acetone-d_6) δ 144.8, 132.6, 132.1, 131.6, 127.8, 123.8, 67.5, 66.9, 27.5, 12.1; IR (neat, cm^{-1}): 3256, 2921, 1298, 1164, 695; ESI HRMS *m/z* (M+Na)$^+$ calcd 326.9773, obsd 326.9782.

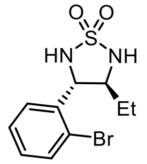

3-(2-Bromophenyl)-4-ethyl-1,2,5-thiadiazolidine 1,1-dioxide (15). White solid; Yield = 57%; Electricity = 2.5 F mol^{-1}; ^{1}H NMR (500 MHz, acetone-d_6) δ 7.86 (dd, J = 7.9, 1.7 Hz, 1H), 7.62 (dd, J = 8.0, 1.2 Hz, 1H), 7.47–7.44 (m, 1H), 7.28–7.25 (m, 1H), 6.68 (d, J = 6.5 Hz, 1H), 6.26 (d, J = 7.3 Hz, 1H), 5.00 (dd, J = 6.5, 4.2 Hz, 1H), 3.56–3.51 (m, 1H), 1.96–1.90 (m, 2H), 1.02 (t, J = 7.4 Hz, 3H); ^{13}C NMR (126 MHz, acetone-d_6) δ 141.4, 134.3, 131.2, 130.9, 129.6, 123.8, 67.7, 67.0, 28.0, 12.0; IR (neat, cm^{-1}): 3256, 2917, 2849, 1162, 591; ESI HRMS *m/z* (M+Na)$^+$ calcd 326.9773, obsd 326.9784.

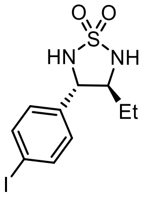

3-Ethyl-4-(4-iodophenyl)-1,2,5-thiadiazolidine 1,1-dioxide (16). White solid; Yield = 61%; Electricity = 2.1 F mol^{-1}; ^{1}H NMR (500 MHz, acetone-d_6) δ 7.78–7.75 (m, 2H), 7.36–7.34 (m, 2H), 6.56 (d, J = 6.3 Hz, 1H), 6.19 (d, J = 8.2 Hz, 1H), 4.52 (t, J = 6.7 Hz, 1H), 3.49 (ddt, J = 8.3, 6.8, 5.1 Hz, 1H), 1.80–1.73 (m, 2H), 0.98 (t, J = 7.4 Hz, 3H); ^{13}C NMR (126 MHz, acetone-d_6) δ 141.8, 139.2, 131.0, 94.6, 67.5, 67.1, 27.4, 12.2; IR (neat, cm^{-1}): 3258, 2926, 1485, 1163, 1006; ESI HRMS *m/z* (M+Na)$^+$ calcd 374.9635, obsd 374.9646.

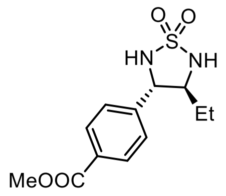

Methyl 4-(4-ethyl-1,1-dioxido-1,2,5-thiadiazolidin-3-yl)benzoate (17). Light yellow oil; Yield = 45%; Electricity = 2.9 F mol^{-1}; ^{1}H NMR (500 MHz, $CDCl_3$) δ 8.05–8.04 (m, 2H), 7.52–7.50 (m, 2H), 5.05 (d, *J* = 5.9 Hz, 1H), 4.77 (d, *J* = 8.3 Hz, 1H), 4.54 (dd, *J* = 7.7, 5.9 Hz, 1H), 3.92 (s, 3H), 3.62–3.56 (m, 1H), 1.75–1.64 (m, 2H), 0.98 (t, *J* = 7.4 Hz, 3H); ^{13}C NMR (126 MHz, $CDCl_3$) δ 166.7, 142.9, 130.9, 130.5, 127.3, 66.4, 66.0, 52.5, 25.7, 11.1; IR (neat, cm^{-1}): 3258, 2928, 1719, 1283, 1163; ESI HRMS *m/z* $(M+Na)^+$ calcd 307.0723, obsd 307.0732.

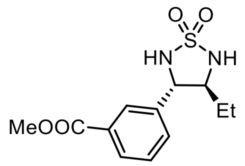

Methyl 3-(4-ethyl-1,1-dioxido-1,2,5-thiadiazolidin-3-yl)benzoate (18). Light yellow oil; Yield = 52%; Electricity = 2.6 F mol^{-1}; ^{1}H NMR (500 MHz, acetone-d_6) δ 8.17–8.15 (m, 1H), 7.99–7.96 (m, 1H), 7.81–7.79 (m, 1H), 7.56–7.53 (m, 1H), 6.63 (d, *J* = 6.2 Hz, 1H), 6.24 (d, *J* = 8.1 Hz, 1H), 4.65 (dd, *J* = 6.6 Hz, 1H), 3.90 (s, 3H), 3.54 (ddt, *J* = 7.2 Hz, 1H), 1.82–1.76 (m, 2H), 0.99 (t, *J* = 7.4 Hz, 3H); ^{13}C NMR (126 MHz, acetone-d_6) δ 167.8, 142.5, 133.5, 132.3, 130.6, 130.5, 129.7, 67.5, 67.3, 53.2, 27.4, 12.2; IR (neat, cm^{-1}): 3254, 2967, 1712, 1294, 1163, 755; ESI HRMS *m/z* $(M+Na)^+$ calcd 307.0723, obsd 307.0735.

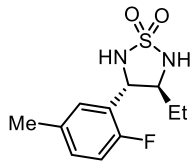

3-Ethyl-4-(2-fluoro-5-methylphenyl)-1,2,5-thiadiazolidine 1,1-dioxide (19). Light yellow solid; Yield = 82%; Electricity = 2.1 F mol^{-1}; ^{1}H NMR (500 MHz, $CDCl_3$) δ 7.40–7.38 (m, 1H), 7.12–7.09 (m, 1H), 6.97–6.93 (m, 1H), 4.94 (d, *J* = 6.7 Hz, 1H), 4.83–4.80 (m, 1H), 4.74 (d, *J* = 8.2 Hz, 1H), 3.66–3.60 (m, 1H), 2.34 (s, 3H), 1.80–1.68 (m, 2H), 1.00 (t, *J* = 7.4 Hz, 3H); ^{13}C NMR (126 MHz, $CDCl_3$) δ 158.6 (d, J_{C-F} = 244.3 Hz), 134.8 (d, J_{C-F} = 3.5 Hz), 130.9 (d, J_{C-F} = 8.1 Hz), 128.8 (d, J_{C-F} = 3.2 Hz), 124.4 (d, J_{C-F} = 12.5 Hz), 115.6 (d, J_{C-F} = 21.7 Hz), 66.0 (d, J_{C-F} = 1.3 Hz), 60.3 (d, J_{C-F} = 2.8 Hz), 25.8, 20.9, 10.9; ^{19}F NMR (471 MHz, $CDCl_3$) δ -123.7; IR (neat, cm^{-1}): 3260, 2970, 1503, 1168, 815; ESI HRMS *m/z* $(M+Na)^+$ calcd 281.0730, obsd 281.0739.

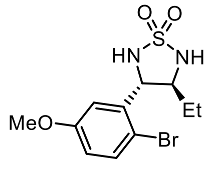

3-(2-Bromo-5-methoxyphenyl)-4-ethyl-1,2,5-thiadiazolidine 1,1-dioxide (20). White solid; Yield = 61%; Electricity = 2.3 F mol^{-1}; ^1H NMR (500 MHz, acetone-d_6) δ 7.50–7.48 (m, 2H), 6.85 (dd, *J* = 8.8, 3.1 Hz, 1H), 6.70 (d, *J* = 6.6 Hz, 1H), 6.28 (d, *J* = 7.3 Hz, 1H), 4.94 (dd, *J* = 6.6, 3.7 Hz, 1H), 3.81 (s, 3H), 3.56–3.48 (m, 1H), 1.97–1.91 (m, 2H), 1.03 (t, *J* = 7.4 Hz, 3H); ^{13}C NMR (126 MHz, acetone-d_6) δ 160.4, 141.8, 134.2, 116.1, 115.6, 112.8, 67.0, 66.3, 55.8, 27.4, 11.2; IR (neat, cm^{-1}): 3269, 2920, 1164, 1132, 601; ESI HRMS *m/z* $(M+Na)^+$ calcd 356.9879, obsd 356.9887.

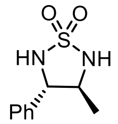

3-Methyl-4-phenyl-1,2,5-thiadiazolidine 1,1-dioxide (21). Colorless oil; Yield = 63%; Electricity = 2.6 F mol^{-1}; ^1H NMR (500 MHz, acetone-d_6) δ 7.53–7.51 (m, 2H), 7.41–7.38 (m, 2H), 7.36–7.32 (m, 1H), 6.48 (d, *J* = 6.3 Hz, 1H), 6.12 (d, *J* = 8.6 Hz, 1H), 4.46 (dd, *J* = 7.9, 6.3 Hz, 1H), 3.70–3.63 (m, 1H), 1.33 (d, *J* = 6.5 Hz, 3H); ^{13}C NMR (126 MHz, acetone-d_6) δ 140.8, 130.2, 129.8, 128.8, 69.9, 61.7, 18.2; IR (neat, cm^{-1}): 3259, 1317, 1164, 701; ESI HRMS *m/z* $(M+Na)^+$ calcd 235.0512, obsd 235.0508;

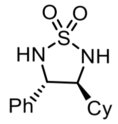

3-Cyclohexyl-4-phenyl-1,2,5-thiadiazolidine 1,1-dioxide (22). Light yellow oil; Yield = 88%; Electricity = 2.9 F mol^{-1}; ^1H NMR (500 MHz, acetone-d_6) δ 7.55–7.53 (m, 2H), 7.39–7.36 (m, 2H), 7.32–7.29 (m, 1H), 6.38 (d, *J* = 6.1 Hz, 1H), 6.17 (d, *J* = 7.6 Hz, 1H), 4.76 (dd, *J* = 6.1 Hz, *J* = 5.9 Hz, 1H), 3.51–3.47 (m, 1H), 2.04–1.97 (m, 1H), 1.80–1.73 (m, 2H), 1.71–1.63 (m, 2H), 1.32–1.26 (m, 1H), 1.26–1.17 (m, 2H), 1.17–1.07 (m, 2H), 1.06–0.97 (m, 1H); ^{13}C NMR (126 MHz, acetone-d_6) δ 142.7, 130.1, 129.4, 128.9, 70.6, 64.4, 42.0, 32.0, 30.2, 27.7, 27.5, 27.3; IR (neat, cm^{-1}): 3263, 2927, 2853, 1165, 701; ESI HRMS *m/z* $(M+Na)^+$ calcd 303.1138, obsd 303.1140.

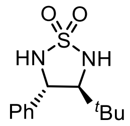

3-(*tert*-Butyl)-4-phenyl-1,2,5-thiadiazolidine 1,1-dioxide (23). 1.2 mmol of sulfamide and 0.2 mmol of $BF_3{\cdot}Et_2O$ were used. Light yellow oil; Yield = 53%; Electricity = 2.1 F mol^{-1}; ^{1}H NMR (500 MHz, acetone-d_6) δ 7.61–7.57 (m, 2H), 7.37 (dd, *J* = 8.3, 6.9 Hz, 2H), 7.32–7.27 (m, 1H), 6.45 (d, *J* = 6.5 Hz, 1H), 6.32 (d, *J* = 7.5 Hz, 1H), 4.82 (dd, *J* = 6.5, 5.0 Hz, 1H), 3.62 (dd, *J* = 7.4, 4.9 Hz, 1H), 1.00 (s, 9H); ^{13}C NMR (126 MHz, acetone-d_6) δ 144.1, 130.1, 129.2, 129.0, 74.6, 61.6, 36.2, 27.3; IR (neat, cm^{-1}): 3268, 2961, 2872, 1163, 746, 701; ESI HRMS *m/z* $(M+Na)^+$ calcd 277.0981, obsd 277.0986.

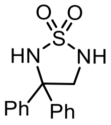

3,3-Diphenyl-1,2,5-thiadiazolidine 1,1-dioxide (24). White solid; Yield = 40%; ^{1}H NMR (500 MHz, acetone-d_6) δ 7.55–7.47 (m, 4H), 7.35 (dd, *J* = 8.5, 6.9 Hz, 4H), 7.30–7.24 (m, 2H), 6.56 (brs, 1H), 6.23 (t, *J* = 8.2 Hz, 1H), 4.25 (d, *J* = 8.2 Hz, 2H); ^{13}C NMR (126 MHz, acetone-d_6) δ 144.2, 129.2, 128.3, 127.7, 73.6, 57.1; IR (neat, cm^{-1}): 3356, 2919, 2850, 1384, 1093; ESI HRMS *m/z* $(M+Na)^+$ calcd 297.0668, obsd 297.0665.

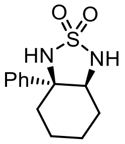

3*a*-Phenyloctahydrobenzo[*c*][1,2,5]thiadiazole 2,2-dioxide (25). The configuration was determined using NOE experiment. Light yellow oil; Yield = 77%; Electricity = 2.8 F mol^{-1}; ^{1}H NMR (500 MHz, acetone-d_6) δ 7.68–7.67 (m, 2H), 7.39–7.32 (m, 2H), 7.29–7.24 (m, 1H), 6.22 (s, 1H), 6.04 (d, *J* = 5.8 Hz, 1H), 4.11 (dt, *J* = 9.1, 5.9 Hz, 1H), 2.15–2.09 (m, 1H), 2.08–2.01 (m, 1H), 2.01–1.89 (m, 2H), 1.85–1.76 (m, 1H), 1.76–1.65 (m, 1H), 1.58–1.47 (m, 2H); ^{13}C NMR (126 MHz, acetone-d_6) δ 146.0, 129.7, 128.7, 128.4, 71.0, 63.5, 38.6, 29.8, 23.7, 22.7; IR (neat, cm^{-1}): 3272, 2938, 1170, 702; ESI HRMS *m/z* $(M+Na)^+$ calcd 275.0825, obsd 275.0829.

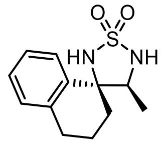

4'-Methyl-3,4-dihydro-2*H*-spiro[naphthalene-1,3'-[1,2,5]thiadiazolidine] 1',1'-dioxide (26). Using the standard condition but without $BF_3{\cdot}Et_2O$. The relative stereochemistry was determined using NOE experiment. Light yellow solid; Yield = 37%; Electricity = 2.8 F mol^{-1}; ^{1}H NMR (500 MHz, acetone-d_6) δ 7.68 (dd, *J* = 7.6, 1.6 Hz, 1H), 7.29–7.19 (m, 2H), 7.16–7.11 (m, 1H), 6.19 (d, *J* = 10.3 Hz, 1H), 5.98 (brs, 1H), 4.27 (dq, *J* = 10.3, 6.7 Hz, 1H), 2.78 (t, *J* = 6.2 Hz, 2H), 2.18–2.12 (m, 1H), 2.12–2.07 (m, 1H),

1.93–1.86 (m, 2H), 1.20 (d, J = 6.7 Hz, 3H); ^{13}C NMR (126 MHz, acetone-d_6) δ 140.9, 137.2, 130.9, 129.4, 128.6, 128.1, 69.1, 64.0, 31.3, 31.0, 20.6, 14.5; IR (neat, cm^{-1}): 3255, 2926, 1321, 1161, 762; ESI HRMS *m/z* (M+Na)$^+$ calcd 275.0825, obsd 275.0833.

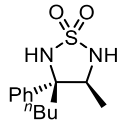

3-Butyl-4-methyl-3-phenyl-1,2,5-thiadiazolidine 1,1-dioxide (27). Isolated as a 9:1 mixture of diastereomers and only the major isomer was shown. The relative stereochemistry of major isomer was determined using NOE experiment. White solid; Yield = 79%; Electricity = 2.4 F mol^{-1}; ^{1}H NMR (500 MHz, acetone-d_6) δ 7.63–7.59 (m, 2H), 7.37 (dd, J = 8.4, 7.0 Hz, 2H), 7.31–7.25 (m, 1H), 6.31 (brs, 1H), 6.10 (d, J = 7.9 Hz, 1H), 3.94–3.82 (m, 1H), 2.05–2.00 (m, 2H), 1.38 (d, J = 6.9 Hz, 3H), 1.28 (dtd, J = 12.9, 7.8, 7.2, 4.8 Hz, 3H), 0.93–0.85 (m, 1H), 0.80 (t, J = 7.2 Hz, 3H); ^{13}C NMR (126 MHz, acetone-d_6) δ 144.4, 129.8, 128.7, 128.2, 74.7, 65.0, 36.1, 28.0, 24.4, 15.6, 14.9; IR (neat, cm^{-1}): 3257, 2958, 1325, 1170, 703; ESI HRMS *m/z* (M+Na)$^+$ calcd 291.1138, obsd 291.1143.

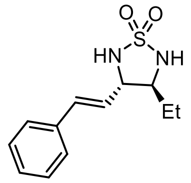

3-Ethyl-4-((*E*)-styryl)-1,2,5-thiadiazolidine 1,1-dioxide (28). The relative stereochemistry of major isomer was determined using NOE experiment. Light yellow oil; Yield = 67%; Electricity = 2.4 F mol^{-1}; ^{1}H NMR (500 MHz, $CDCl_3$) δ 7.40–7.37 (m, 2H), 7.36–7.32 (m, 2H), 7.31–7.27 (m, 1H), 6.68 (d, J = 15.7 Hz, 1H), 6.11 (dd, J = 15.7, 8.0 Hz, 1H), 4.65 (d, J = 6.1 Hz, 1H), 4.57 (d, J = 8.4 Hz, 1H), 4.09 (td, J = 7.9, 5.9 Hz, 1H), 3.51 (qd, J = 8.4, 4.1 Hz, 1H), 1.80 (dqd, J = 15.0, 7.6, 4.2 Hz, 1H), 1.68–1.58 (m, 1H), 1.03 (t, J = 7.4 Hz, 3H); ^{13}C NMR (126 MHz, $CDCl_3$) δ 135.7, 135.5, 128.9, 128.8, 126.9, 124.6, 65.8, 64.5, 25.5, 11.2; IR (neat, cm^{-1}): 3256, 2928, 1302, 1166, 970, 752, 694; ESI HRMS *m/z* (M+Na)$^+$ calcd 275.0825, obsd 275.0833.

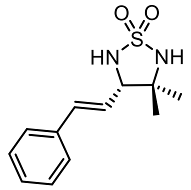

(*E*)-3,3-Dimethyl-4-styryl-1,2,5-thiadiazolidine 1,1-dioxide (29). Light yellow oil; Yield = 47%; Electricity = 2.4 F mol^{-1}; ^{1}H NMR (500 MHz, $CDCl_3$) δ 7.41–7.38 (m, 2H), 7.37–7.32 (m, 2H), 7.32–7.27 (m, 1H), 6.70 (d, *J* = 15.7 Hz, 1H), 6.07 (dd, *J* = 15.8, 8.0 Hz, 1H), 4.82 (brs, 1H), 4.55 (brs, 1H), 4.19 (d, *J* = 7.9 Hz, 1H), 1.41 (s, 3H), 1.37 (s, 3H); ^{13}C NMR (126 MHz, $CDCl_3$) δ 135.9, 135.6, 129.0, 128.8, 126.9, 121.8, 70.1, 64.7, 26.2, 23.1; IR (neat, cm^{-1}): 3263, 2922, 2851, 1384, 1162, 700; ESI HRMS *m/z* $(M+Na)^+$ calcd 275.0825, obsd 275.0834.

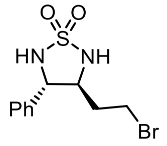

3-(2-Bromoethyl)-4-phenyl-1,2,5-thiadiazolidine 1,1-dioxide (30). Light yellow oil; Yield = 61%; Electricity = 2.0 F mol^{-1}; ^{1}H NMR (500 MHz, $CDCl_3$) δ 7.46–7.34 (m, 5H), 5.11–5.02 (m, 2H), 4.53 (t, *J* = 6.6 Hz, 1H), 3.96–3.85 (m, 1H), 3.50–3.36 (m, 2H), 2.29 (ddt, *J* = 15.2, 10.4, 5.5 Hz, 1H), 2.21–2.10 (m, 1H); ^{13}C NMR (126 MHz, $CDCl_3$) δ 137.1, 129.4, 129.3, 127.1, 66.1, 63.0, 35.5, 29.5; IR (neat, cm^{-1}): 3263, 2924, 1309, 1164, 701; ESI HRMS *m/z* $(M+Na)^+$ calcd 326.9773, obsd 326.9780.

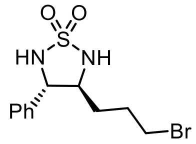

3-(3-Bromopropyl)-4-phenyl-1,2,5-thiadiazolidine 1,1-dioxide (31). Colorless oil; Yield = 55%; Electricity = 2.6 F mol^{-1}; ^{1}H NMR (500 MHz, $CDCl_3$) δ 7.47–7.34 (m, 5H), 4.49 (d, *J* = 8.0 Hz, 1H), 3.70 (td, *J* = 8.0, 4.6 Hz, 1H), 3.44–3.33 (m, 2H), 2.04–1.95 (m, 1H), 1.91–1.78 (m, 3H); ^{13}C NMR (126 MHz, $CDCl_3$) δ 137.1, 129.4, 129.3, 127.3, 67.3, 64.2, 32.9, 30.8, 29.6; IR (neat, cm^{-1}): 3248, 2923, 2852, 1317, 1168, 701.

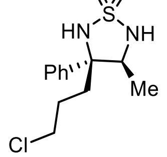

3-(3-Chloropropyl)-4-methyl-3-phenyl-1,2,5-thiadiazolidine 1,1-dioxide (32). Isolated as an 8:1 mixture of diastereomers and only the major isomer was shown. Light yellow solid; Yield = 74%; Electricity = 2.3 F mol^{-1}; ^{1}H NMR (500 MHz, $CDCl_3$) δ 7.54–7.48 (m, 2H), 7.45–7.39 (m, 2H), 7.37–7.31 (m, 1H), 4.83 (s, 1H), 4.62 (d, *J* = 8.0 Hz, 1H), 3.97–3.87 (m, 1H), 3.59–3.47 (m, 2H), 2.21 (ddd, *J* = 14.0, 11.4, 4.4 Hz, 1H), 2.07 (ddd, *J* = 13.8, 11.2, 4.7 Hz, 1H), 1.81–1.70 (m, 1H), 1.59–1.46 (m, 1H), 1.39 (d, *J* = 6.9

Hz, 3H); ^{13}C NMR (126 MHz, $CDCl_3$) δ 140.1, 129.3, 128.4, 126.2, 73.3, 64.5, 45.1, 31.9, 27.6, 14.5; IR (neat, cm^{-1}): 3249, 2923, 1324, 1170, 703; ESI HRMS *m/z* $(M+Na)^+$ calcd 311.0591, obsd 311.0598.

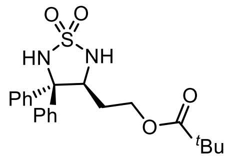

2-(1,1-Dioxido-4,4-diphenyl-1,2,5-thiadiazolidin-3-yl)ethyl pivalate (33). Colorless oil; Yield = 61%; Electricity = 2.3 F mol^{-1}; ^{1}H NMR (500 MHz, $CDCl_3$) δ 7.41–7.27 (m, 10H), 4.88 (s, 1H), 4.78–4.69 (m, 1H), 4.50 (d, *J* = 10.4 Hz, 1H), 4.23 (ddd, *J* = 11.2, 5.4, 4.3 Hz, 1H), 4.14 (ddd, *J* = 11.3, 9.9, 4.1 Hz, 1H), 2.15 (dddd, *J* = 15.2, 9.8, 5.4, 2.2 Hz, 1H), 1.26 (s, 9H), 1.21 (td, *J* = 5.7, 5.1, 4.2 Hz, 1H); ^{13}C NMR (126 MHz, $CDCl_3$) δ 178.5, 141.7, 139.9, 129.3, 129.0, 128.6, 128.3, 128.1, 127.0, 76.3, 61.6, 61.5, 39.0, 30.3, 27.4; IR (neat, cm^{-1}): 3248, 2973, 1716, 1168, 702; ESI HRMS *m/z* $(M+Na)^+$ calcd 441.1455, obsd 441.1253.

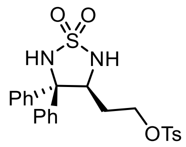

2-(1,1-Dioxido-4,4-diphenyl-1,2,5-thiadiazolidin-3-yl)ethyl 4-methylbenzenesulfon-ate (34). White solid; Yield = 80%; Electricity = 2.4 F mol^{-1}; ^{1}H NMR (500 MHz, acetone-d_6) δ 7.85–7.79 (m, 2H), 7.55–7.51 (m, 2H), 7.49–7.44 (m, 2H), 7.39–7.33 (m, 4H), 7.33–7.27 (m, 3H), 7.27–7.22 (m, 1H), 6.90 (s, 1H), 6.24 (d, *J* = 8.5 Hz, 1H), 4.83 (ddd, *J* = 11.6, 8.5, 2.8 Hz, 1H), 4.33–4.20 (m, 2H), 2.44 (s, 3H), 2.11–1.99 (m, 1H), 1.60 (dddd, *J* = 14.5, 11.8, 5.8, 4.0 Hz, 1H); ^{13}C NMR (126 MHz, acetone-d_6) δ 146.6, 144.6, 142.8, 134.9, 131.7, 130.1, 129.7, 129.4, 129.3, 129.2, 129.0, 128.7, 77.2, 69.6, 61.7, 31.5, 22.3; IR (neat, cm^{-1}): 3261, 3062, 2925, 1355, 1174, 703, 554; ESI HRMS *m/z* $(M+Na)^+$ calcd 495.1019, obsd 495.1022.

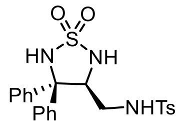

***N*-((1,1-Dioxido-4,4-diphenyl-1,2,5-thiadiazolidin-3-yl)methyl)-4-methylbenzenesulf onamide (35).** Light yellow solid; Yield = 66%; Electricity = 2.4 F mol^{-1}; ^{1}H NMR (500 MHz, $CDCl_3$) δ 7.68–7.58 (m, 2H), 7.42–7.36 (m, 2H), 7.36–7.20 (m, 10H), 5.27 (dd, *J* = 8.3, 4.5 Hz, 1H), 5.24 (s, 1H), 5.17 (d, *J* = 6.4 Hz, 1H), 4.73 (ddd, *J* = 10.1, 6.2, 3.4 Hz, 1H), 3.15 (ddd, *J* = 14.2, 8.2, 3.5 Hz, 1H), 2.73 (ddd, *J* = 14.5, 10.2, 4.5 Hz, 1H), 2.39 (s, 3H);

^{13}C NMR (126 MHz, $CDCl_3$) δ 144.0, 141.8, 139.5, 136.7, 130.0, 129.3, 129.0, 128.6, 128.4, 127.2, 126.7, 126.6, 74.3, 63.9, 43.3, 21.7; IR (neat, cm^{-1}): 3270, 1326, 1162, 703, 551; ESI HRMS *m/z* $(M+Na)^+$ calcd 480.1022, obsd 480.1040.

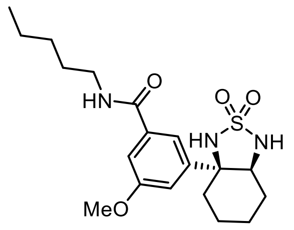

3-(2,2-Dioxidohexahydrobenzo[*c*][1,2,5]thiadiazol-3a(3*H*)-yl)-5-methoxy-*N*-pentylbenzamide (36). Light yellow solid; Yield = 45%; Electricity = 8.7 F mol^{-1}; ^{1}H NMR (500 MHz, acetone-d_6) δ 7.75 (t, J = 6.0 Hz, 1H), 7.71–7.67 (m, 1H), 7.47 (dd, J = 2.5, 1.7 Hz, 1H), 7.33 (dd, J = 2.5, 1.4 Hz, 1H), 6.37–6.30 (m, 1H), 6.15 (d, J = 5.5 Hz, 1H), 4.12 (dt, J = 9.7, 5.9 Hz, 1H), 3.84 (s, 3H), 3.37 (td, J = 7.3, 5.9 Hz, 2H), 2.14–2.09 (m, 1H), 2.04–1.89 (m, 3H), 1.84–1.77 (m, 1H), 1.77–1.67 (m, 1H), 1.63–1.52 (m, 4H), 1.37–1.32 (m, 4H), 0.92–0.88 (m, 3H); ^{13}C NMR (126 MHz, acetone-d_6) δ 167.8, 161.2, 148.3, 138.2, 119.1, 117.8, 112.7, 71.4, 63.6, 56.5, 41.2, 38.5, 30.9, 30.7, 30.0, 24.0, 23.8, 22.6, 15.1; IR (neat, cm^{-1}): 3419, 2932, 1640, 1593, 1543, 1313, 1167, 696, 559; ESI HRMS *m/z* $(M+Na)^+$ calcd 418.1771, obsd 418.1763.

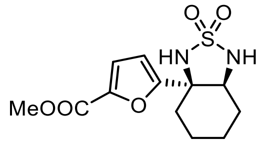

Methyl 5-(2,2-dioxidohexahydrobenzo[*c*][1,2,5]thiadiazol-3*a*(3*H*)-yl)furan-2-carboxylate (37). White solid; Yield = 90%; Electricity = 2.3 F mol^{-1}; ^{1}H NMR (500 MHz, acetone-d_6) δ 7.17 (d, J = 3.5 Hz, 1H), 6.75 (d, J = 3.5 Hz, 1H), 6.56 (s, 1H), 6.33 (d, J = 6.6 Hz, 1H), 4.12 (ddd, J = 7.8, 6.6, 5.5 Hz, 1H), 3.84 (s, 3H), 2.20–2.15 (m, 2H), 2.02 (ddd, J = 11.6, 6.7, 2.6 Hz, 1H), 1.95–1.88 (m, 1H), 1.84–1.75 (m, 1H), 1.75–1.65 (m, 1H), 1.55–1.43 (m, 2H); ^{13}C NMR (126 MHz, acetone-d_6) δ 161.3, 160.0, 145.7, 120.1, 112.3, 66.9, 60.9, 52.8, 34.1, 28.5, 22.7, 22.4; IR (neat, cm^{-1}): 3267, 2924, 2853, 1723, 1309, 1146; ESI HRMS *m/z* $(M+Na)^+$ calcd 323.0672, obsd 323.0684.

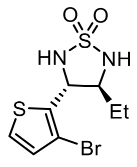

3-(3-Bromothiophen-2-yl)-4-ethyl-1,2,5-thiadiazolidine 1,1-dioxide (38). Light yellow oil; Yield = 69%; Electricity = 2.5 F mol^{-1}; ^{1}H NMR (500 MHz, $CDCl_3$) δ 7.35 (d, J = 5.3

Hz, 1H), 6.97 (d, J = 5.3 Hz, 1H), 5.09 (d, J = 5.9 Hz, 1H), 4.96 (t, J = 6.6 Hz, 1H), 4.87 (d, J = 8.2 Hz, 1H), 3.74 (ddt, J = 12.6, 8.2, 4.3 Hz, 1H), 1.89–1.81 (m, 1H), 1.78–1.70 (m, 1H), 1.02 (t, J = 7.4 Hz, 3H); ^{13}C NMR (126 MHz, $CDCl_3$) δ 136.1, 130.6, 126.7, 110.8, 66.2, 61.4, 25.8, 11.0; IR (neat, cm^{-1}): 3269, 2922, 1384, 1165, 717; ESI HRMS *m/z* (M+Na)$^+$ calcd 332.9338, obsd 332.9349.

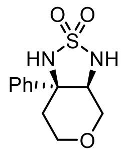

7a-Phenylhexahydro-3*H*-pyrano[3,4-*c*][1,2,5]thiadiazole 2,2-dioxide (39). Light yellow oil; Yield = 57%; Electricity = 3.0 F mol^{-1}; ^{1}H NMR (500 MHz, acetone-d_6) δ 7.59–7.53 (m, 2H), 7.29–7.23 (m, 2H), 7.21–7.14 (m, 1H), 6.55 (s, 1H), 6.08 (d, J = 6.2 Hz, 1H), 3.96 (dt, J = 8.9, 6.0 Hz, 1H), 3.82 (dd, J = 12.3, 5.8 Hz, 1H), 3.72–3.65 (m, 2H), 3.61 (dt, J = 12.0, 4.3 Hz, 1H), 2.08–1.97 (m, 2H); ^{13}C NMR (126 MHz, acetone-d_6) δ 145.0, 129.9, 129.1, 128.4, 68.7, 68.2, 65.0, 60.9, 38.1; IR (neat, cm^{-1}): 3280, 2918, 1384, 1170, 702; ESI HRMS *m/z* (M+Na)$^+$ calcd 277.0617, obsd 277.0624.

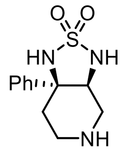

7a-Phenyloctahydro-[1,2,5]thiadiazolo[3,4-*c*]pyridine 2,2-dioxide (40). HBF_4 (1.5 equiv) was used to instead of $BF_3{\cdot}Et_2O$. Light yellow solid; Yield = 59%; Electricity = 5.5 F mol^{-1}; ^{1}H NMR (500 MHz, DMSO-d_6) δ 7.66–7.54 (m, 3H), 7.42–7.33 (m, 2H), 7.30–7.22 (m, 1H), 7.06 (brs, 1H), 3.76 (dd, J = 8.3, 5.6 Hz, 1H), 2.94, 2.86 (AB of ABX_1, 2H, J_{AB} = 13.6 Hz, J_{AX} = 5.7 Hz, J_{BX} = 8.4 Hz), 2.78, 2.59 (AB of ABX_2, 2H, J_{AB} = 13.2 Hz, J_{AX} = 6.6 Hz, J_{BX} = 4.6 Hz), 1.92–1.74 (m, 2H); 13C NMR (126 MHz, DMSO-d6) δ 145.5, 129.0, 128.0, 127.6, 68.3, 60.2, 48.2, 42.5, 38.3; IR (neat, cm−1): 3357, 2921, 1633, 1447, 1163, 762, 702; ESI HRMS m/z (M+Na)+ calcd 276.0777, obsd 276.0779.

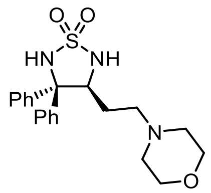

4-(2-Morpholinoethyl)-3,3-diphenyl-1,2,5-thiadiazolidine 1,1-dioxide (41). HBF_4 (1.5 equiv) was used instead of $BF_3{\cdot}Et_2O$. White solid; Yield = 95%; Electricity = 2.5 F mol^{-1}; ^{1}H NMR (500 MHz, DMSO-d_6) δ 8.13 (s, 1H), 7.45 (d, J = 7.5 Hz, 2H), 7.39–7.33 (m, 2H), 7.31–7.24 (m, 6H), 7.24–7.20 (m, 1H), 4.58 (ddd, J = 9.6, 6.8, 2.4 Hz, 1H), 3.69–

3.45 (m, 4H), 2.42 (dt, J = 12.3, 7.5 Hz, 1H), 2.37–2.29 (m, 3H), 2.29–2.20 (m, 2H), 1.53 (dtd, J = 14.0, 7.6, 3.8 Hz, 1H), 1.22–1.11 (m, 1H); ^{13}C NMR (126 MHz, DMSO-d_6) δ 144.9, 143.0, 129.2, 128.7, 128.5, 128.2, 128.0 (2C), 74.9, 67.2, 62.1, 56.5, 54.4, 28.4; IR (neat, cm^{-1}): 3258, 2901, 1291, 1164, 760; ESI HRMS *m/z* (M+Na)$^+$ calcd 410.1509, obsd 410. 1506.

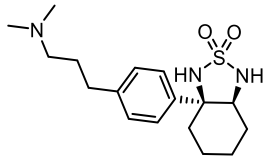

3a-(4-(3-(Dimethylamino)propyl)phenyl)octahydrobenzo[*c*][1,2,5]thiadiazole 2,2-dioxide (42). HBF_4 (1.5 equiv) was used instead of $BF_3{\cdot}Et_2O$. Colorless oil; Yield = 72%; Electricity = 2.9 F mol^{-1}; ^{1}H NMR (500 MHz, $CDCl_3$) δ 7.50–7.44 (m, 2H), 7.24–7.17 (m, 2H), 4.87 (brs, 2H), 4.05 (dd, J = 7.7, 5.6 Hz, 1H), 2.65–2.58 (m, 2H), 2.28 (dd, J = 8.5, 6.5 Hz, 2H), 2.23–2.12 (m, 7H), 2.09–1.93 (m, 3H), 1.81–1.69 (m, 3H), 1.62–1.43 (m, 3H); ^{13}C NMR (126 MHz, $CDCl_3$) δ 142.4, 139.3, 128.9, 126.6, 69.7, 62.8, 59.3, 45.4, 36.2, 33.3, 29.2, 27.2, 21.7, 21.6; IR (neat, cm^{-1}): 3060, 2924, 1297, 1158, 1115, 702; ESI HRMS *m/z* (M+H)$^+$ calcd 338.1897, obsd 338.1908.

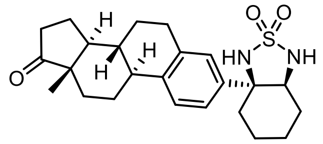

(8*R*,9*S*,13*S*,14*S*)-3-((3a*S*,7a*S*)-2,2-Dioxidohexahydrobenzo[*c*][1,2,5]thiadiazol-3a(3*H*)-yl)-13-methyl-6,7,8,9,11,12,13,14,15,16-decahydro-17*H*-cyclopenta[a]phenanthren-17-one (43). Isolated as a 1:1 mixture of diastereomers. Light yellow solid; Yield = 76%; Electricity = 3.1 F mol^{-1}; ^{1}H NMR (500 MHz, $CDCl_3$) δ 7.34–7.28 (m, 3H), 4.67–4.60 (m, 2H), 4.07 (dd, J = 6.8 Hz, 1H), 3.00–2.87 (m, 2H), 2.51 (dd, J = 18.9, 8.7 Hz, 1H), 2.44–2.37 (m, 1H), 2.29 (td, J = 11.1, 4.1 Hz, 1H), 2.20–2.12 (m, 2H), 2.11–2.00 (m, 4H), 2.00–1.93 (m, 2H), 1.75 (dp, J = 11.8, 5.2, 4.2 Hz, 1H), 1.68–1.56 (m, 3H), 1.56–1.40 (m, 6H), 0.91 (s, 3H); ^{13}C NMR (126 MHz, $CDCl_3$) δ 220.9, 139.9 (2C), 139.1 (2C), 137.1 (2C), 127.2, 125.9 (2C), 123.9 (2C), 77.4, 69.6, 62.7 (2C), 50.6, 48.1, 44.4, 38.1 (2C), 36.0 (2C), 31.7, 29.7, 27.1, 26.6 (2C), 25.8, 25.7, 21.7 (2C), 21.6, 14.0; IR (neat, cm^{-1}): 3266, 2933, 1733, 1171, 735; ESI HRMS *m/z* (M+ Na)$^+$ calcd 451.2026, obsd 451.2255.

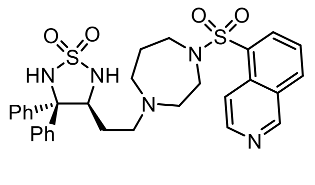

4-(2-(4-(Isoquinolin-5-ylsulfonyl)-1,4-diazepan-1-yl)ethyl)-3,3-diphenyl-1,2,5-thiadia

zolidine 1,1-dioxide (44). HBF_4 (2 equiv) was used instead of $BF_3{\cdot}Et_2O$. White foamy solid; Yield = 82%; Electricity = 3.0 F mol^{-1}; ^{1}H NMR (500 MHz, $CDCl_3$) δ 9.29 (s, 1H), 8.63 (d, *J* = 6.1 Hz, 1H), 8.42 (d, *J* = 6.2 Hz, 1H), 8.30 (d, *J* = 7.3 Hz, 1H), 8.17 (d, *J* = 8.2 Hz, 1H), 7.72–7.64 (m, 1H), 7.40–7.27 (m, 10H), 6.87 (brs, 1H), 5.06 (s, 1H), 4.72 (dd, *J* = 10.3, 2.8 Hz, 1H), 3.55–3.38 (m, 4H), 2.79–2.72 (m, 1H), 2.71–2.61 (m, 3H), 2.61–2.55 (m, 1H), 2.55–2.47 (m, 1H), 1.94–1.82 (m, 2H), 1.51–1.43 (m, 1H), 1.41–1.30 (m, 1H); ^{13}C NMR (126 MHz, $CDCl_3$) δ 153.3, 145.1, 143.0, 140.9, 134.5, 133.6, 133.0, 131.8, 129.3, 129.1, 128.6, 128.5, 128.0, 127.9, 127.1, 126.1, 117.7, 74.6, 64.3, 56.9, 55.8, 54.7, 47.9, 47.0, 28.2, 27.7; IR (neat, cm^{-1}):3444, 1635, 1448, 1324, 1158, 734, 704, 592; ESI HRMS *m/z* (M+Na)$^+$ calcd 614.1866, obsd 614.1853.

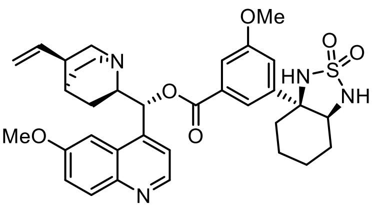

(*R*)-(6-Methoxyquinolin-4-yl)((1*S*,2*R*,4*S*,5*R*)-5-vinylquinuclidin-2-yl)methyl 3-((3a*S*,7a*S*)-2,2-dioxidohexahydrobenzo[*c*][1,2,5]thiadiazol-3a(3*H*)-yl)-5-methoxyb enzoate (45). Isolated as a 1:1 mixture of diastereomers. HBF_4 (1.5 equiv) was used instead of $BF_3{\cdot}Et_2O$. White solid; Yield = 54%; Electricity = 3.0 F mol^{-1}; ^{1}H NMR (500 MHz, $CDCl_3$) δ 8.65 (d, *J* = 4.5 Hz, 0.6H), 8.60 (d, *J* = 4.6 Hz, 0.4H), 8.02–7.97 (m, 1H), 7.97–7.94 (m, 0.4H), 7.85–7.82 (m, 0.6H), 7.54 (dd, *J* = 2.5, 1.3 Hz, 0.6H), 7.52–7.47 (m, 1.6H), 7.45 (d, *J* = 2.7 Hz, 0.4H), 7.40–7.38 (m, 1H), 7.38–7.34 (m, 1.4H), 6.73–6.63 (m, 1H), 5.85–5.73 (m, 1H), 5.64 (brs, 0.6H), 5.32 (brs, 0.4H), 5.22–5.05 (m, 1H), 5.03–4.90 (m, 2H), 4.03–3.95 (m, 3.6H), 3.83 (d, *J* = 5.8 Hz, 3H), 3.81–3.74 (m, 0.4H), 3.48–3.36 (m, 1H), 3.20–3.08 (m, 1H), 3.08–2.98 (m, 1H), 2.69–2.61 (m, 1H), 2.61–2.47 (m, 1H), 2.31–2.22 (m, 1H), 2.16–2.08 (m, 1H), 2.07–1.92 (m, 2H), 1.92–1.67 (m, 6H), 1.63–1.34 (m, 4H); ^{13}C NMR (126 MHz, $CDCl_3$) δ 165.3 (2C), 160.1, 158.3, 158.2, 147.5, 145.9, 145.7, 144.7, 144.6, 143.6 (2C), 141.7, 131.9, 131.8, 131.4, 131.3, 126.8, 126.7, 122.1, 122.0, 120.3, 120.0, 118.6, 118.4, 114.8, 114.2, 113.8, 101.6, 101.4, 75.3, 70.2 (2C), 63.1, 63.0, 59.3, 57.0, 57.0, 56.0, 55.9, 42.8, 42.7, 39.7, 36.9, 36.8, 28.3 (2C), 28.1, 28.0, 27.8, 27.7, 23.8, 23.6, 22.6, 22.5, 21.1; IR (neat, cm^{-1}): 3443, 2942, 1623, 1458, 1302, 1228, 1168, 1029, 734; ESI HRMS *m/z* (M+Na)$^+$ calcd 655.2561, obsd 655.2559.

2-Butyl-4-cyclohexyl-3-phenyl-1,2,5-thiadiazolidine 1,1-dioxide (46). 6 equiv of sulfamide and 1 equiv of $BF_3{\cdot}Et_2O$ were used. Light yellow oil; Yield = 70%; Electricity = 2.6 F mol^{-1}; ^{1}H NMR (500 MHz, $CDCl_3$) δ 7.45–7.33 (m, 5H), 4.91 (d, J = 8.1 Hz, 1H), 4.19 (d, J = 7.6 Hz, 1H), 3.44 (td, J = 7.8, 5.5 Hz, 1H), 2.97 (ddd, J = 13.7, 8.6, 5.4 Hz, 1H), 2.73 (ddd, J = 13.4, 9.0, 7.0 Hz, 1H), 1.88 (dd, J = 12.4, 3.6 Hz, 1H), 1.77 (t, J = 6.7 Hz, 1H), 1.66 (dt, J = 15.0, 8.0 Hz, 2H), 1.57 (d, J = 12.7 Hz, 1H), 1.53–1.46 (m, 2H), 1.29–1.08 (m, 7H), 0.97–0.89 (m, 1H), 0.76 (t, J = 7.4 Hz, 3H); ^{13}C NMR (126 MHz, $CDCl_3$) δ 138.6, 129.1, 128.9, 128.2, 68.0, 65.7, 45.6, 39.9, 30.7, 29.6, 28.1, 26.2, 26.1, 25.8, 20.1, 13.6; IR (neat, cm^{-1}): 3262, 2928, 2854, 1167, 702; ESI HRMS *m/z* (M+ Na)$^+$ calcd 359.1764, obsd 359.1764.

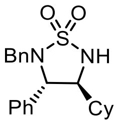

2-Benzyl-4-cyclohexyl-3-phenyl-1,2,5-thiadiazolidine 1,1-dioxide (47). 6 equiv of sulfamide and 1 equiv of $BF_3{\cdot}Et_2O$ were used. Light yellow oil; Yield = 68%; Electricity = 2.4 F mol^{-1}; ^{1}H NMR (500 MHz, acetone-d_6) δ 7.47–7.42 (m, 2H), 7.38–7.27 (m, 3H), 7.22–7.12 (m, 5H), 6.46 (d, J = 8.2 Hz, 1H), 4.29 (d, J = 6.8 Hz, 1H), 4.05, 4.01 (ABq, J_{AB} = 15.0 Hz, 2H), 3.45 (dt, J = 8.3, 6.7 Hz, 1H), 1.98–1.90 (m, 1H), 1.77–1.68 (m, 1H), 1.67–1.51 (m, 4H), 1.27–1.03 (m, 4H), 0.93–0.84 (m, 1H); ^{13}C NMR (126 MHz, acetone-d_6) δ 141.0, 137.7, 130.4, 130.3, 129.9, 129.5, 128.9, 69.0, 66.8, 50.1, 42.1, 31.8, 30.0, 27.7, 27.4, 27.2; IR (neat, cm^{-1}): 3257, 2926, 2853, 1303, 1167, 758, 699; ESI HRMS *m/z* (M+ Na)$^+$ calcd 393.1607, obsd 393.1615.

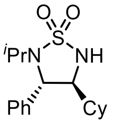

4-Cyclohexyl-2-isopropyl-3-phenyl-1,2,5-thiadiazolidine 1,1-dioxide (48). 6 equiv of sulfamide and 1 equiv of $BF_3{\cdot}Et_2O$ were used. Light yellow oil; Yield = 88%; Electricity = 2.6 F mol^{-1}; ^{1}H NMR (500 MHz, $CDCl_3$) δ 7.46–7.42 (m, 2H), 7.41–7.36 (m, 2H), 7.35–7.30 (m, 1H), 4.65 (d, J = 7.9 Hz, 1H), 4.52 (d, J = 5.6 Hz, 1H), 3.55 (hept, J = 6.7 Hz, 1H), 3.24 (td, J = 7.5, 5.8 Hz, 1H), 2.00–1.92 (m, 1H), 1.82–1.71 (m, 3H), 1.69–1.63 (m, 1H), 1.59 (tdd, J = 11.6, 6.8, 3.3 Hz, 1H), 1.34 (d, J = 6.9 Hz, 3H), 1.30–1.19 (m, 2H), 1.14 (ddt, J = 25.3, 12.6, 3.2 Hz, 1H), 1.05 (d, J = 6.7 Hz, 3H), 1.04–0.92 (m, 2H); ^{13}C NMR (126 MHz, $CDCl_3$) δ 140.6, 129.1, 128.5, 127.4, 66.1, 64.0, 48.1, 39.8, 30.8, 28.5, 26.3, 26.1, 25.8, 20.8, 20.0; IR (neat, cm^{-1}): 3257, 2927, 2853, 1181, 1153, 701; ESI HRMS *m/z* (M+ Na)$^+$ calcd 345.1607, obsd 345.1608.

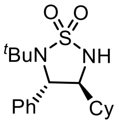

2-(*tert*-Butyl)-4-cyclohexyl-3-phenyl-1,2,5-thiadiazolidine 1,1-dioxide (49). 6 equiv of sulfamide and 1 equiv of $BF_3{\cdot}Et_2O$ were used. Colorless oil; Yield = 67%; Electricity = 2.6 F mol^{-1}; ^{1}H NMR (500 MHz, $CDCl_3$) δ 7.37 (d, *J* = 7.2 Hz, 2H), 7.33–7.28 (m, 2H), 7.23–7.18 (m, 1H), 4.69 (s, 1H), 4.54 (d, *J* = 6.8 Hz, 1H), 2.68 (ddd, *J* = 10.6, 6.8, 1.2 Hz, 1H), 2.15–2.01 (m, 2H), 1.83–1.72 (m, 2H), 1.71–1.60 (m, 2H), 1.27 (s, 9H), 1.26–1.17 (m, 2H), 1.16–1.05 (m, 1H), 1.04–0.93 (m, 1H), 0.78–0.69 (m, 1H); ^{13}C NMR (126 MHz, $CDCl_3$) δ 143.8, 129.2, 127.7, 125.5, 66.9, 65.2, 56.8, 39.1, 31.0, 29.6, 28.5, 26.5, 26.0, 25.6; IR (neat, cm^{-1}): 3252, 2925, 2852, 1155, 703, 489; ESI HRMS *m/z* (M+ Na)$^+$ calcd 359.1764, obsd 359.1767.

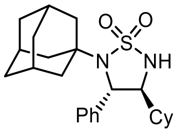

2-(Adamantan-1-yl)-4-cyclohexyl-3-phenyl-1,2,5-thiadiazolidine 1,1-dioxide (50). 6 equiv of sulfamide and 1 equiv of $BF_3{\cdot}Et_2O$ were used. Light yellow solid; Yield = 91%; Electricity = 2.7 F mol^{-1}; ^{1}H NMR (500 MHz, $CDCl_3$) δ 7.45 (d, *J* = 7.5 Hz, 2H), 7.40–7.35 (m, 2H), 7.30–7.25 (m, 1H), 4.87 (s, 1H), 4.60 (d, *J* = 6.7 Hz, 1H), 2.73 (dd, *J* = 10.7, 6.6 Hz, 1H), 2.23–2.12 (m, 2H), 2.08–1.95 (m, 9H), 1.87 (qt, *J* = 10.9, 3.5 Hz, 2H), 1.77–1.68 (m, 2H), 1.67–1.58 (m, 6H), 1.43–1.25 (m, 2H), 1.17 (qt, *J* = 12.5, 3.8 Hz, 1H), 1.08 (qd, *J* = 12.3, 3.6 Hz, 1H), 0.87–0.76 (m, 1H); ^{13}C NMR (126 MHz, $CDCl_3$) δ 144.0, 129.2, 127.6, 125.5, 77.4, 67.3, 63.4, 57.7, 40.7, 39.0, 36.1, 31.1, 29.7, 29.6, 26.5, 26.0, 25.6; IR (neat, cm^{-1}): 3291, 2919, 1164, 703; ESI HRMS *m/z* (M+ Na)$^+$ calcd 437.2233, obsd 437.2230.

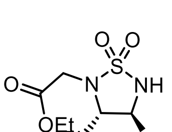

Ethyl 2-(4-cyclohexyl-1,1-dioxido-3-phenyl-1,2,5-thiadiazolidin-2-yl)acetate (51). 6 equiv of sulfamide and 1 equiv of $BF_3{\cdot}Et_2O$ were used. White solid; Yield = 83%; Electricity = 2.4 F mol^{-1}; ^{1}H NMR (500 MHz, $CDCl_3$) δ 7.44–7.32 (m, 5H), 4.72 (d, *J* = 8.1 Hz, 1H), 4.63 (d, *J* = 10.0 Hz, 1H), 4.21–4.08 (m, 2H), 3.93 (d, *J* = 18.2 Hz, 1H), 3.56–3.44 (m, 2H), 1.94 (dt, *J* = 12.7, 3.3 Hz, 1H), 1.84–1.77 (m, 1H), 1.71–1.55 (m, 4H), 1.32–1.15 (m, 5H), 1.15–1.05 (m, 2H), 1.05–0.94 (m, 1H); ^{13}C NMR (126 MHz,

$CDCl_3$) δ 169.1, 137.7, 129.2, 129.0, 128.3, 68.0, 65.9, 61.6, 45.2, 39.3, 30.9, 28.3, 26.2, 26.1, 25.9, 14.2; IR (neat, cm^{-1}): 3259, 2928, 2853, 1743, 1169, 702; ESI HRMS *m/z* (M+ Na)$^+$ calcd 389.1505, obsd 389.1520.

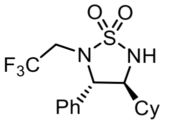

4-Cyclohexyl-3-phenyl-2-(2,2,2-trifluoroethyl)-1,2,5-thiadiazolidine 1,1-dioxide (52). 6 equiv of sulfamide and 1 equiv of $BF_3{\cdot}Et_2O$ were used. Colorless oil; Yield = 70%; Electricity = 2.5 F mol^{-1}; ^1H NMR (500 MHz, $CDCl_3$) δ 7.46–7.37 (m, 5H), 4.71 (d, J = 8.8 Hz, 1H), 4.48 (d, J = 7.1 Hz, 1H), 3.54 (dt, J = 8.8, 6.7 Hz, 1H), 3.50, 3.47 (ABq, 2H, J_{AB} = 8.9 Hz), 1.91–1.85 (m, 1H), 1.81–1.75 (m, 1H), 1.72–1.54 (m, 3H), 1.29–1.01 (m, 5H), 0.93 (qd, J = 12.2, 3.4 Hz, 1H); ^{13}C NMR (126 MHz, $CDCl_3$) δ 136.7, 129.5, 128.4, 123.8 (q, $J_{C\text{-}F}$ = 279.7 Hz), 69.2, 66.1, 45.28 (q, $J_{C\text{-}F}$ = 35.6 Hz), 40.0, 30.5, 28.5, 26.1, 26.0, 25.8; ^{19}F NMR (471 MHz, $CDCl_3$) δ -70.0; IR (neat, cm^{-1}): 3259, 2930, 2855, 1170, 702; ESI HRMS *m/z* (M+ Na)$^+$ calcd 385.1168, obsd 385.1184.

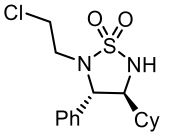

2-(2-Chloroethyl)-4-cyclohexyl-3-phenyl-1,2,5-thiadiazolidine 1,1-dioxide (53). 6 equiv of sulfamide and 1 equiv of $BF_3{\cdot}Et_2O$ were used. Colorless oil; Yield = 70%; Electricity = 2.5 F mol^{-1}; ^1H NMR (500 MHz, CDCl) δ 7.46–7.36 (m, 5H), 4.85 (d, J = 8.1 Hz, 1H), 4.32 (d, J = 7.6 Hz, 1H), 3.58–3.41 (m, 3H), 3.35 (ddd, J = 13.6, 7.5, 5.8 Hz, 1H), 3.10 (ddd, J = 14.4, 8.0, 6.4 Hz, 1H), 1.88 (dt, J = 12.9, 3.2 Hz, 1H), 1.78 (dd, J = 13.5, 3.9 Hz, 1H), 1.72–1.61 (m, 2H), 1.60–1.48 (m, 2H), 1.29–1.03 (m, 4H), 0.93 (qd, J = 12.2, 3.5 Hz, 1H); ^{13}C NMR (126 MHz, $CDCl_3$) δ 137.7, 129.4 (2C), 128.3, 68.1, 66.0, 46.6, 41.0, 39.8, 30.7, 28.1, 26.2, 26.0, 25.8; IR (neat, cm^{-1}): 3261, 2927, 2853, 1304, 1165, 701; ESI HRMS *m/z* (M+ Na)$^+$ calcd 365.1061, obsd 365.1071.

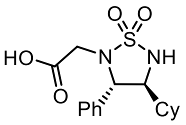

2-(4-Cyclohexyl-1,1-dioxido-3-phenyl-1,2,5-thiadiazolidin-2-yl)acetic acid (54). Light yellow oil; Yield = 47%; Electricity = 4.3 F mol^{-1}; ^1H NMR (500 MHz, acetone-d_6) δ 7.53–7.48 (m, 2H), 7.44–7.38 (m, 2H), 7.38–7.33 (m, 1H), 6.23 (d, J = 8.9 Hz, 1H), 4.79 (d, J = 7.0 Hz, 1H), 3.81, 3.53 (ABq, J_{AB} = 18.1 Hz, 2H), 3.50–3.41 (m, 1H), 2.04–1.98 (m,

1H), 1.78–1.67 (m, 3H), 1.67–1.57 (m, 2H), 1.27–1.18 (m, 2H), 1.18–1.05 (m, 2H), 1.01–0.90 (m, 1H); ^{13}C NMR (126 MHz, acetone-d_6) δ 170.9, 140.5, 130.4, 130.1, 129.9, 68.8, 67.3, 45.9, 41.7, 31.9, 30.2, 27.6, 27.4, 27.2; IR (neat, cm^{-1}): 3443, 2927, 2853, 1636, 1401, 1165; ESI HRMS *m/z* (M+Na)$^+$ calcd 361.1192, obsd 361.1191.

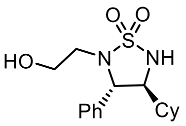

4-Cyclohexyl-2-(2-hydroxyethyl)-3-phenyl-1,2,5-thiadiazolidine 1,1-dioxide (55). Light yellow oil; Yield = 54%; Electricity = 4.2 F mol^{-1}; ^{1}H NMR (500 MHz, $CDCl_3$) δ 7.44–7.35 (m, 5H), 4.79 (d, *J* = 8.1 Hz, 1H), 4.32 (d, *J* = 7.6 Hz, 1H), 3.78–3.69 (m, 1H), 3.57–3.44 (m, 2H), 3.18–3.11 (m, 1H), 3.00 (ddd, *J* = 14.1, 7.7, 4.2 Hz, 1H), 2.35–2.27 (m, 1H), 1.93–1.85 (m, 1H), 1.83–1.74 (m, 1H), 1.67–1.62 (m, 2H), 1.61–1.48 (m, 2H), 1.28–1.04 (m, 4H), 0.97–0.87 (m, 1H); ^{13}C NMR (126 MHz, $CDCl_3$) δ 137.9, 129.5, 129.3, 128.2, 68.0, 66.2, 59.8, 47.6, 39.9, 30.7, 28.2, 26.2, 26.1, 25.8; IR (neat, cm^{-1}): 3362, 2923, 2852, 1654, 1633, 1450, 1164, 744, 702, 620; ESI HRMS *m/z* (M+Na)$^+$ calcd 347.1400, obsd 347.1405.

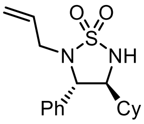

2-Allyl-4-cyclohexyl-3-phenyl-1,2,5-thiadiazolidine 1,1-dioxide (56). 6 equiv of sulfamide and 1 equiv of $BF_3{\cdot}Et_2O$ were used. Colorless oil; Yield = 79%; Electricity = 2.8 F mol^{-1}; ^{1}H NMR (400 MHz, $CDCl_3$) δ 7.41–7.30 (m, 5H), 5.83 (dddd, *J* = 17.1, 10.2, 8.3, 5.0 Hz, 1H), 5.13 (dq, *J* = 10.2, 1.3 Hz, 1H), 5.07 (dq, *J* = 17.1, 1.5 Hz, 1H), 4.81 (d, *J* = 8.0 Hz, 1H), 4.28 (d, *J* = 7.5 Hz, 1H), 3.63 (ddt, *J* = 15.0, 5.1, 1.6 Hz, 1H), 3.48–3.34 (m, 2H), 1.95–1.84 (m, 1H), 1.82–1.73 (m, 1H), 1.71–1.46 (m, 4H), 1.28–1.05 (m, 4H), 0.92 (qd, *J* = 12.2, 3.5 Hz, 1H); ^{13}C NMR (126 MHz, $CDCl_3$) δ 138.0, 131.9, 129.1, 128.9, 128.3, 119.9, 66.4, 65.8, 47.6, 39.9, 30.7, 28.1, 26.2, 26.1, 25.8; IR (neat, cm^{-1}): 3259, 2926, 2852, 1304, 1167, 702; ESI HRMS *m/z* (M+ Na)$^+$ calcd 343.1451, obsd 343.1455.

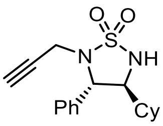

4-Cyclohexyl-3-phenyl-2-(prop-2-yn-1-yl)-1,2,5-thiadiazolidine 1,1-dioxide (57). 6 equiv of sulfamide and 1 equiv of $BF_3{\cdot}Et_2O$ were used. Colorless oil; Yield = 79%;

Electricity = 2.6 F mol^{-1}; ^{1}H NMR (500 MHz, $CDCl_3$) δ 7.48–7.34 (m, 5H), 4.85 (d, *J* = 8.2 Hz, 1H), 4.51 (d, *J* = 7.8 Hz, 1H), 3.95, 3.43 (AB of ABX_1, 2H, J_{AB} = 18.1 Hz, J_{AX} = 2.0 Hz, J_{BX} = 2.3 Hz), 3.50 (td, *J* = 8.0, 5.6 Hz, 1H), 2.33 (t, *J* = 2.3 Hz, 1H), 1.96–1.87 (m, 1H), 1.81–1.75 (m, 1H), 1.71–1.57 (m, 3H), 1.29–1.04 (m, 5H), 0.98–0.88 (m, 1H); ^{13}C NMR (126 MHz, $CDCl_3$) δ 137.2, 129.2 (2C), 128.4, 76.4, 74.5, 65.5 (2C), 39.9, 33.8, 30.7, 28.2, 26.2, 26.1, 25.8; IR (neat, cm^{-1}): 3278, 2927, 2853, 1170, 702, 666; ESI HRMS *m/z* (M+ Na)$^+$ calcd 341.1294, obsd 341.1304.

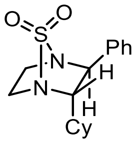

2-Cyclohexyl-3-phenyl-7-thia-1,4-diazabicyclo[2.2.1]heptane 7,7-dioxide (58). 6 equiv of sulfamide and 1 equiv of $BF_3{\cdot}Et_2O$ were used. Colorless oil; Yield = 44%; Electricity = 2.6 F mol^{-1}; ^{1}H NMR (500 MHz, $CDCl_3$) δ 7.47 (d, *J* = 7.4 Hz, 2H), 7.35 (dd, *J* = 8.3, 6.5 Hz, 2H), 7.32–7.27 (m, 1H), 4.07 (dd, *J* = 10.6, 7.0 Hz, 1H), 3.68 (ddd, *J* = 12.0, 8.9, 5.0 Hz, 1H), 3.51–3.40 (m, 2H), 3.19 (ddd, *J* = 12.6, 9.1, 5.0 Hz, 1H), 2.96 (ddd, *J* = 11.9, 9.1, 5.0 Hz, 1H), 2.03–1.90 (m, 1H), 1.86–1.74 (m, 1H), 1.68 (ddd, *J* = 13.4, 5.6, 2.8 Hz, 2H), 1.46–1.34 (m, 2H), 1.30–1.08 (m, 4H), 0.96 (dtd, *J* = 14.5, 11.8, 3.2 Hz, 1H); ^{13}C NMR (126 MHz, $CDCl_3$) δ 138.2, 128.5, 128.4, 128.3, 74.1, 67.5, 54.0, 42.2, 40.9, 32.0, 30.5, 26.2, 26.1, 25.7; IR (neat, cm^{-1}): 2929, 2853, 1374, 1184, 805; ESI HRMS *m/z* (M+ Na)$^+$ calcd 329.1294, obsd 329.1301.

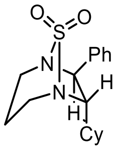

6-Cyclohexyl-7-phenyl-8-thia-1,5-diazabicyclo[3.2.1]octane 8,8-dioxide (59). 6 equiv of sulfamide and 1 equiv of $BF_3{\cdot}Et_2O$ were used. The configuration was determined using X-ray crystallography experiment. White solid; Yield = 77%; Electricity = 2.5 F mol^{-1}; ^{1}H NMR (500 MHz, $CDCl_3$) δ 7.55–7.50 (m, 2H), 7.37–7.32 (m, 2H), 7.32–7.27 (m, 1H), 4.34–4.24 (m, 2H), 4.14 (ddd, *J* = 15.0, 12.8, 6.1 Hz, 1H), 3.91 (dd, *J* = 11.0, 7.8 Hz, 1H), 3.41 (dddd, *J* = 28.0, 15.0, 7.1, 2.1 Hz, 2H), 2.01–1.87 (m, 2H), 1.83–1.73 (m, 2H), 1.68–1.60 (m, 2H), 1.54–1.47 (m, 1H), 1.40–1.33 (m, 1H), 1.27–1.20 (m, 2H), 1.16 (ddt, *J* = 13.8, 11.4, 6.0 Hz, 2H), 0.74–0.65 (m, 1H); ^{13}C NMR (126 MHz, $CDCl_3$) δ 139.3, 128.8 (2C), 128.5, 70.0, 67.7, 56.1, 47.3, 38.5, 32.6, 30.3, 26.2, 26.0, 25.8, 16.4; IR (neat, cm^{-1}): 3304, 2919, 2850, 1384, 1181, 754; ESI HRMS *m/z* (M+ Na)$^+$ calcd 343.1451, obsd 343.1465.

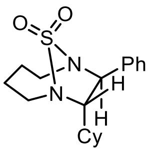

7-Cyclohexyl-8-phenyl-9-thia-1,6-diazabicyclo[4.2.1]nonane 9,9-dioxide (60). 6 equiv of sulfamide and 1 equiv of $BF_3 \cdot Et_2O$ were used. White solid; Yield = 75%; Electricity = 3.1 F mol^{-1}; ^{1}H NMR (500 MHz, $CDCl_3$) δ 7.55–7.50 (m, 2H), 7.38–7.33 (m, 2H), 7.33–7.28 (m, 1H), 4.16 (dd, *J* = 9.3, 1.7 Hz, 1H), 3.84 (td, *J* = 10.0, 9.3, 1.7 Hz, 1H), 3.56–3.42 (m, 2H), 3.26–3.17 (m, 1H), 2.99–2.89 (m, 1H), 2.69–2.54 (m, 1H), 2.06–1.91 (m, 4H), 1.78–1.64 (m, 2H), 1.62–1.51 (m, 2H), 1.49–1.42 (m, 1H), 1.29–1.01 (m, 4H), 0.51–0.38 (m, 1H); ^{13}C NMR (126 MHz, $CDCl_3$) δ 141.4, 129.1, 128.3, 128.2, 75.9, 67.8, 50.0, 44.5, 38.8, 33.6, 30.3, 28.1, 27.9, 26.0, 25.6; IR (neat, cm^{-1}): 2930, 2852, 1173, 733, 705; ESI HRMS *m/z* $(M+Na)^+$ calcd 357.1607, obsd 357.1619.

Transformations of electrolysis products

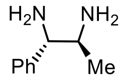

1-Phenylpropane-1,2-diamine (65). The title compound was prepared from **21** by following the procedure described for the synthesis of **67**. Light yellow solid; Yield = 76%; ^{1}H NMR (500 MHz, $CDCl_3$) δ 7.34–7.30 (m, 2H), 7.30–7.27 (m, 2H), 7.26–7.22 (m, 1H), 3.59 (d, J = 6.9 Hz, 1H), 3.03–2.96 (m, 1H), 1.94 (brs, 5H), 0.96 (d, J = 6.4 Hz, 3H); ^{13}C NMR (126 MHz, $CDCl_3$) δ 144.5, 128.5, 127.2, 126.9, 62.8, 52.9, 20.9. Analytical data agree with those reported in the literature[3].

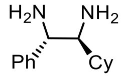

1-Cyclohexyl-2-phenylethane-1,2-diamine (66). The following procedure was adapted from a reported method[4]. **22** (0.280 g, 1.00 mmol) was dissolved in hydrazine monohydrate (10 mL) and stirred at 110 °C under atmospheric conditions for 14 h. The hydrazine was removed under reduced pressure. EtOAc (20 mL) was added to the and the resulting suspension was filtered through celite. The filtrate was extracted with HCl (1 N, 2 x 10 mL). The combined aqueous layer was then adjusted to pH 14 by addition of aqueous NaOH (6 N). The aqueous phase was extracted with EtOAc (2 x 15 mL). The combined organic solution was washed with brine (20 mL), dried over $MgSO_4$, and concentrated under reduced pressure to yield **66** as a light yellow oil (0.230 g, 95% yield). ^{1}H NMR (400 MHz, $CDCl_3$) δ 7.35–7.26 (m, 4H), 7.25–7.19 (m, 1H), 3.92 (d, J = 6.0 Hz, 1H), 2.63–2.53 (m, 1H), 1.82–1.69 (m, 3H), 1.69–1.51 (m, 6H), 1.28–1.07 (m, 5H), 1.05–0.91 (m, 1H); ^{13}C NMR (126 MHz, $CDCl_3$) δ 145.3, 128.6, 127.0, 126.9, 62.2, 57.5, 39.9, 30.9, 28.1, 26.7, 26.6, 26.3; IR (neat, cm^{-1}): 3309, 2928, 1449, 701; ESI HRMS *m/z* $(M+H)^+$ calcd 219.1856, obsd 219.1856.

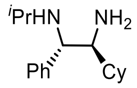

2-Cyclohexyl-*N*^{1}-isopropyl-1-phenylethane-1,2-diamine (67). The following procedure was adapted from a reported method[5]. To a 10 mL two-necked round-bottom flask equipped with a magnetic stir bar and reflux condenser was added **48** (0.328 g, 1.02 mmol) and phenol (0.600 g, 6.38 mmol). HBr (2 N, 5 mL) was added and the mixture was refluxed until complete consumption of **48**. The reaction mixture cooled to rt and washed with EtOAc to remove excess phenol. The acidic aqueous layer was basified with solid NaOH and extracted with Et_2O. The combined organic layers were dried over Na_2SO_4, filtered, and concentrated to yield **67** as a light yellow oil (0.255 g, 96% yield).

^{1}H NMR (500 MHz, $CDCl_3$) δ 7.33–7.28 (m, 2H), 7.26–7.20 (m, 3H), 3.60 (d, J = 7.5 Hz, 1H), 2.60–2.49 (m, 2H), 1.74–1.65 (m, 3H), 1.61–1.56 (m, 1H), 1.56–1.44 (m, 4H), 1.21 (td, J = 11.7, 3.1 Hz, 1H), 1.17–1.01 (m, 5H), 0.99 (d, J = 6.1 Hz, 3H), 0.93 (d, J = 6.4 Hz, 3H); ^{13}C NMR (126 MHz, $CDCl_3$) δ 143.6, 128.3, 127.5, 126.8, 62.6, 61.8, 45.5, 39.4, 31.2, 26.7, 26.6 (2C), 26.2, 24.6, 22.1; IR (neat, cm^{-1}): 3308, 2924, 2851, 1450, 702; ESI HRMS *m/z* $(M+H)^+$ calcd 261.2325, obsd 261.2331.

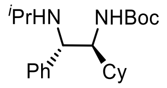

***tert*-Butyl (1-cyclohexyl-2-(isopropylamino)-2-phenylethyl)carbamate (68).** The following procedure was adapted from a reported method[6]. **67** (32 mg, 0.12 mmol, 1.0 equiv) and triethylamine (50 µL, 0.36 mmol, 3.0 equiv) were dissolved in CH_2Cl_2 (2 mL). $(Boc)_2O$ (40 µL, 0.18 mmol, 1.5 equiv) was added slowly. Upon completion, the reaction mixture was concentrated. The residue was purified by flash column chromatography to give **68** (42 mg, 98%). Light pink solid; ^{1}H NMR (500 MHz, $CDCl_3$) δ 7.31–7.24 (m, 4H), 7.24–7.19 (m, 1H), 4.89–4.45 (m, 1H), 3.86 (d, J = 5.8 Hz, 1H), 3.55–3.21 (m, 1H), 2.56 (hept, J = 6.3 Hz, 1H), 1.79–1.66 (m, 4H), 1.61 (d, J = 6.9 Hz, 1H), 1.43–1.28 (m, 9H), 1.22–1.06 (m, 6H), 1.02–0.90 (m, 7H); ^{13}C NMR (126 MHz, $CDCl_3$) δ 156.6, 142.8, 128.4, 127.7, 127.0, 78.9, 60.9, 60.4, 45.7, 39.5, 30.7, 28.6, 26.5, 26.3, 26.2, 24.6, 22.1; IR (neat, cm^{-1}): 3443, 2924, 1636, 1365, 1173, 560; ESI HRMS *m/z* $(M+H)^+$ calcd 361.2850, obsd 361.2850.

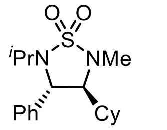

3-Cyclohexyl-5-isopropyl-2-methyl-4-phenyl-1,2,5-thiadiazolidine 1,1-dioxide (69'). Sulfamide **48** (0.33 g, 1.0 mmol, 1.0 equiv) was dissolved in DMF (8 mL) and treated with NaH (0.20 g, 60% dispersion in mineral oil, 5.0 mmol, 5.0 equiv) at rt. After 10 minutes, iodomethane (0.25 mL, 4.0 mmol, 4.0 equiv) was added. Upon completion, the reaction mixture was poured into water (10 mL) and extracted with EtOAc (3 x 20 mL). The combined organic layers were washed with water (2 x 20 mL), dried over Na_2SO_4, and concentrated. The resulting light yellow oil was purified by flash column chromatography to give the title compound as a colorless oil (0.29 g, 88%). ^{1}H NMR (500 MHz, $CDCl_3$) δ 7.45–7.41 (m, 2H), 7.39–7.34 (m, 2H), 7.33–7.29 (m, 1H), 4.31 (d, J = 6.0 Hz, 1H), 3.58 (hept, J = 6.8 Hz, 1H), 2.93–2.88 (m, 1H), 2.81 (s, 3H), 1.85–1.72 (m, 3H), 1.71–1.60 (m, 3H), 1.32 (d, J = 6.8 Hz, 3H), 1.28–1.06 (m, 4H), 1.05–0.94 (m, 4H); ^{13}C NMR (126 MHz, $CDCl_3$) δ 141.4, 129.0, 128.4, 128.0, 72.7, 60.3, 48.8, 40.8, 36.8, 29.6, 29.2, 26.6, 26.4, 20.8, 19.8; IR (neat, cm^{-1}):3450, 2928, 2853, 1368, 1151,

747, 702; ESI HRMS *m/z* $(M+Na)^+$ calcd 359.1764, obsd 359.1757.

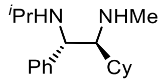

1-Cyclohexyl-*N*²-isopropyl-*N*¹-methyl-2-phenylethane-1,2-diamine (69). The title compound was prepared by following the procedure described for the synthesis of **67**. Light yellow oil; Yield = 83%; ^{1}H NMR (500 MHz, $CDCl_3$) δ 7.33–7.26 (m, 4H), 7.25–7.18 (m, 1H), 3.66 (d, *J* = 7.0 Hz, 1H), 2.54 (hept, *J* = 6.3 Hz, 1H), 2.36 (s, 3H), 2.23 (dd, *J* = 7.0, 3.7 Hz, 1H), 1.94 (brs, 2H), 1.72–1.54 (m, 5H), 1.31–1.17 (m, 2H), 1.15–1.02 (m, 3H), 1.01–0.90 (m, 7H); ^{13}C NMR (126 MHz, $CDCl_3$) δ 144.0, 128.3, 127.8, 126.8, 71.0, 61.6, 45.5, 40.5, 38.3, 31.4, 28.6, 26.9, 26.8, 26.7, 24.6, 22.1; IR (neat, cm^{-1}): 3322, 2925, 1600, 1450, 1120, 701; ESI HRMS *m/z* $(M+H)^+$ calcd 275.2482, obsd 275.2481.

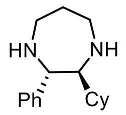

2-Cyclohexyl-3-phenyl-1,4-diazepane (70). The title compound was prepared from **59** by following the procedure described for the synthesis of **67**. Light yellow oil; Yield = 95%; ^{1}H NMR (500 MHz, $CDCl_3$) δ 7.33–7.26 (m, 4H), 7.26–7.20 (m, 1H), 3.60 (d, *J* = 9.8 Hz, 1H), 3.16 (tdd, *J* = 9.8, 8.1, 4.8 Hz, 2H), 2.95 (ddd, *J* = 14.1, 8.3, 6.4 Hz, 1H), 2.87 (ddd, *J* = 13.5, 8.7, 6.4 Hz, 1H), 2.63 (dd, *J* = 9.8, 2.1 Hz, 1H), 2.01 (brs, 2H), 1.80–1.71 (m, 3H), 1.70–1.64 (m, 1H), 1.63–1.58 (m, 1H), 1.57–1.51 (m, 1H), 1.35–1.22 (m, 2H), 1.14–0.92 (m, 5H); ^{13}C NMR (126 MHz, $CDCl_3$) δ 144.2, 128.5, 127.4, 127.2, 71.5, 69.9, 47.3, 47.1, 40.0, 32.8, 31.7, 26.6, 26.5 (2C); IR (neat, cm^{-1}): 3028, 2924, 2850, 1449, 701; ESI HRMS *m/z* $(M+H)^+$ calcd 259.2169, obsd 259.2171.

X-ray crystallography

Single crystals suitable for X-ray diffraction were obtained by slow evaporation of a saturated solution of **59** (cyclohexane/CH_2Cl_2) in a loosely capped vial.

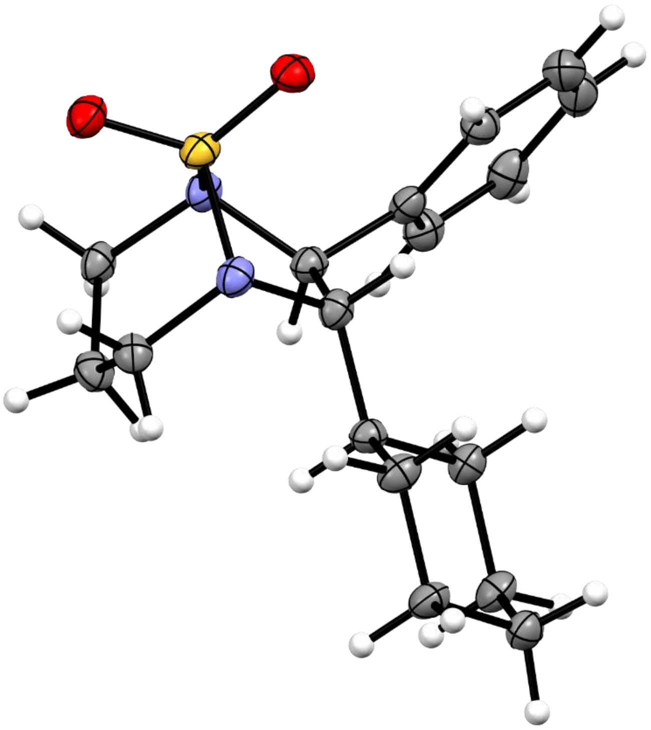

Supplementary Figure 17. ORTEP representation of compound 59. Thermal ellipsoids are shown at 50% probability.

Cyclic voltammetry studies

The cyclic voltammograms were recorded at rt in an electrolyte of Et_4NPF_6 (0.1 M) in MeCN/CH_2Cl_2 (1:2) using a glassy carbon disk working electrode (diameter, 1 mm), a Pt wire auxiliary electrode and a SCE reference electrode. The scan rate is 100 mV/s. The concentrations employed were as following:

Black: **1** (1.5 mM).

Green: **2** (3 mM).

Pink: **1** (1.5 mM), **2** (3 mM).

Blue: **3** (3 mM).

Gray: **1** (1.5 mM), **3** (30 mM).

NMR spectra

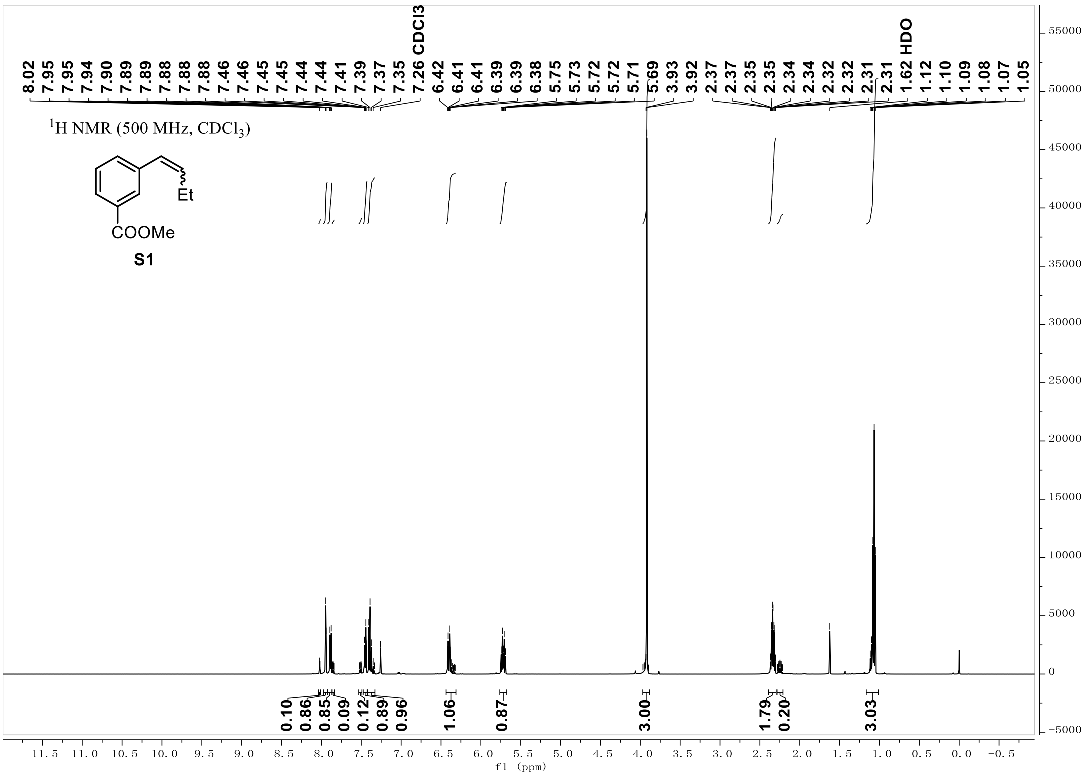

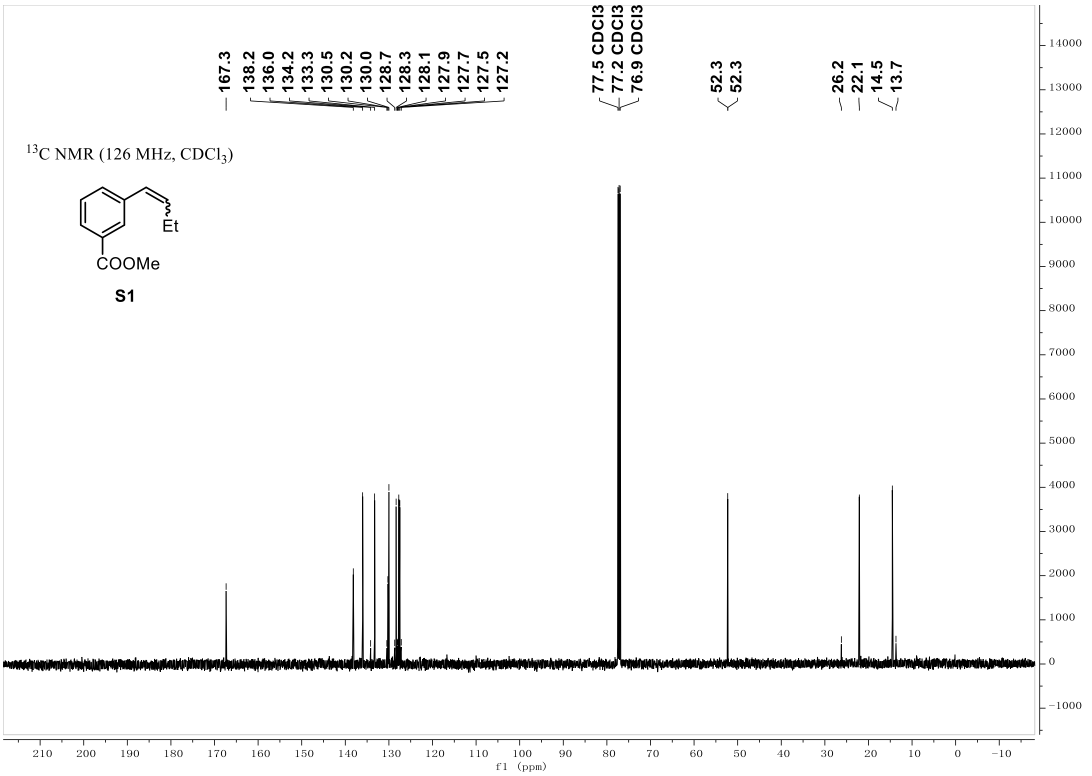

Supplementary Figure 18. ^{1}H NMR and ^{13}C NMR spectra of compound S1.

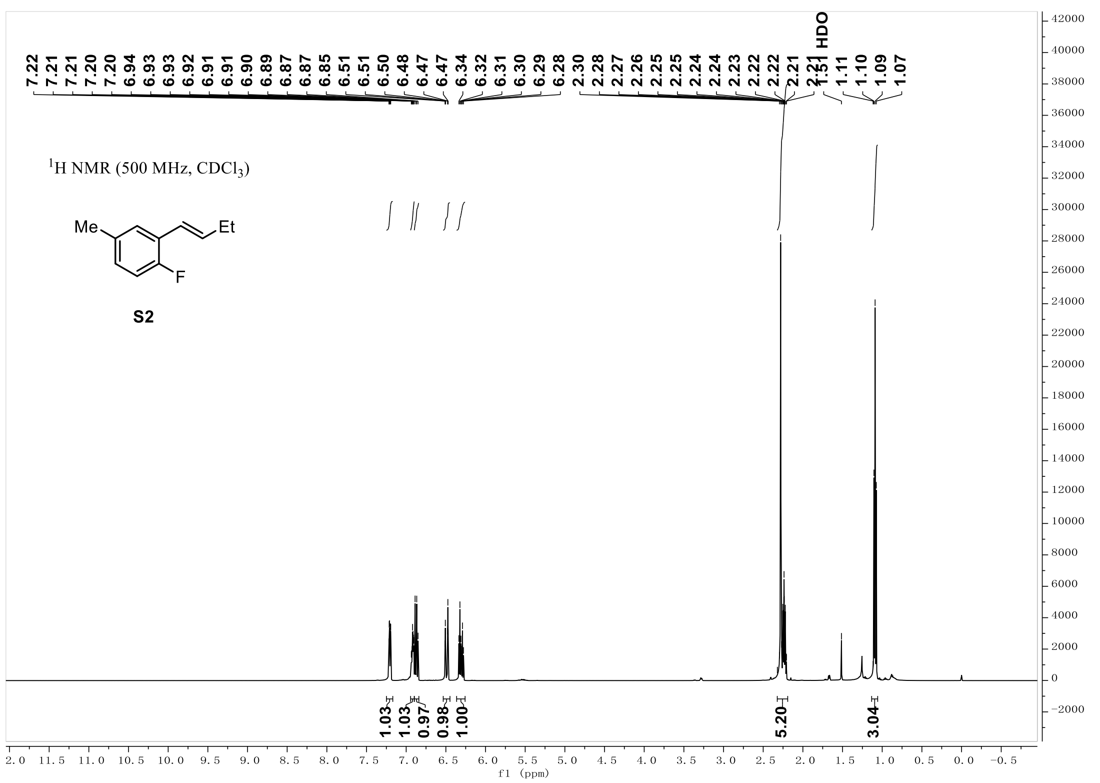

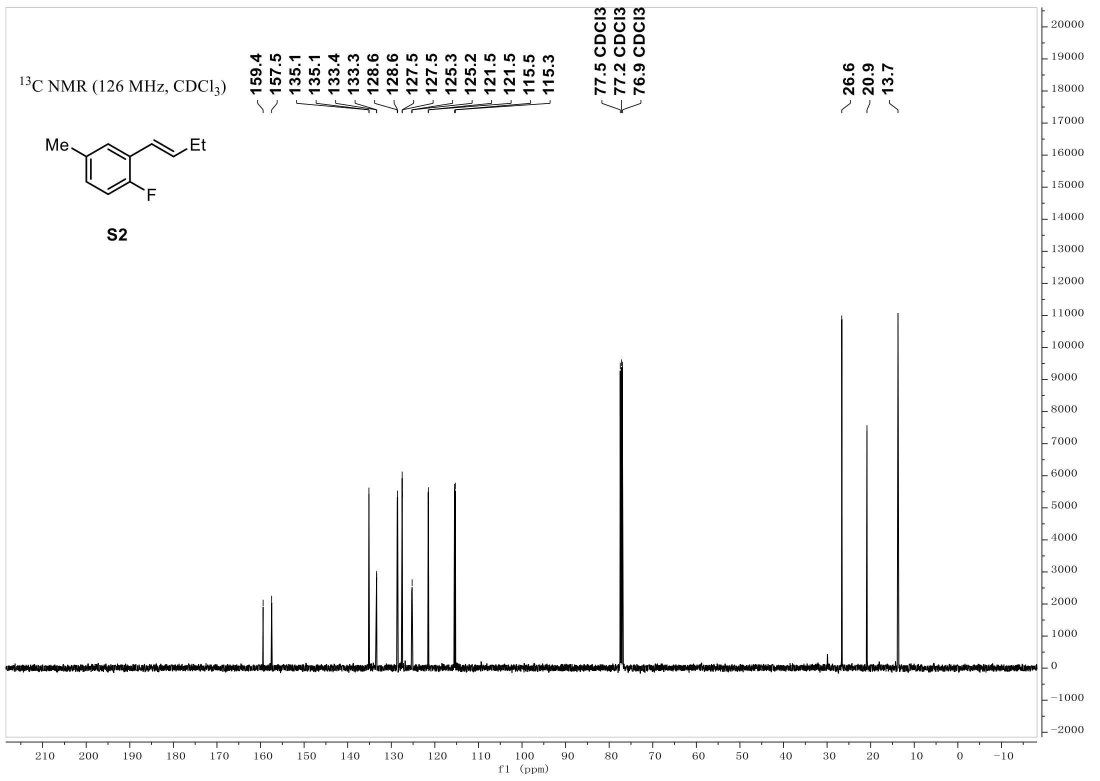

Supplementary Figure 19. ^{1}H NMR and ^{13}C NMR spectra of compound S2.

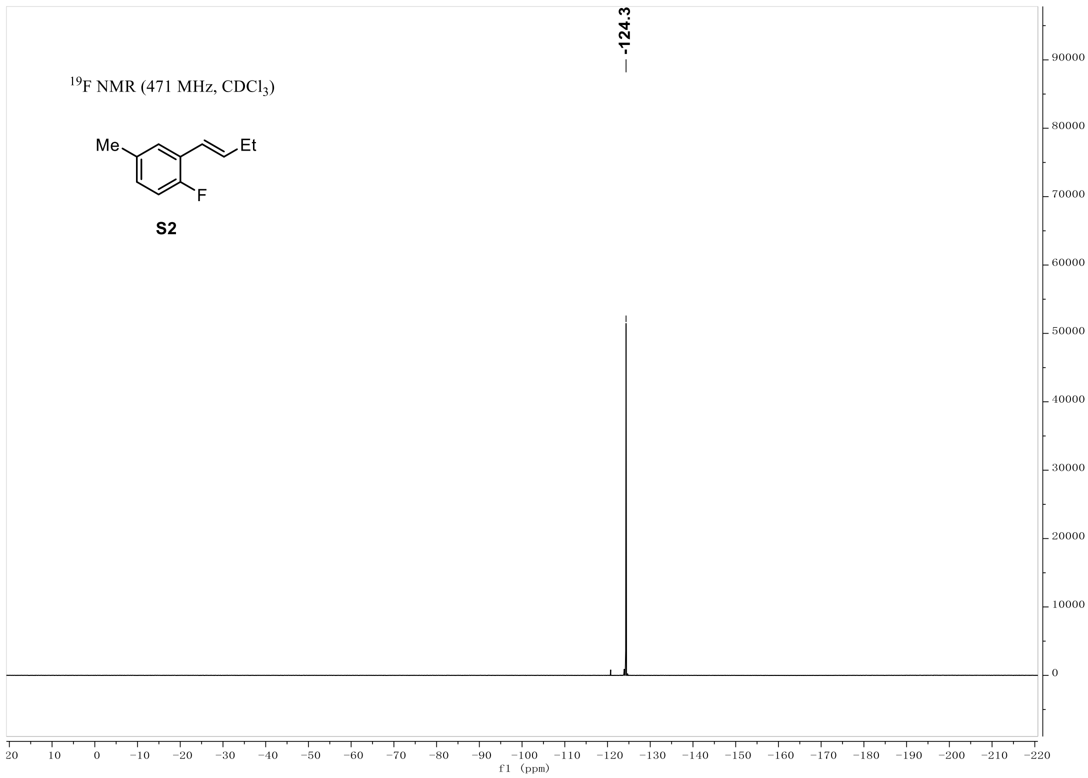

Supplementary Figure 20. ^{19}F NMR spectra of compound S2.

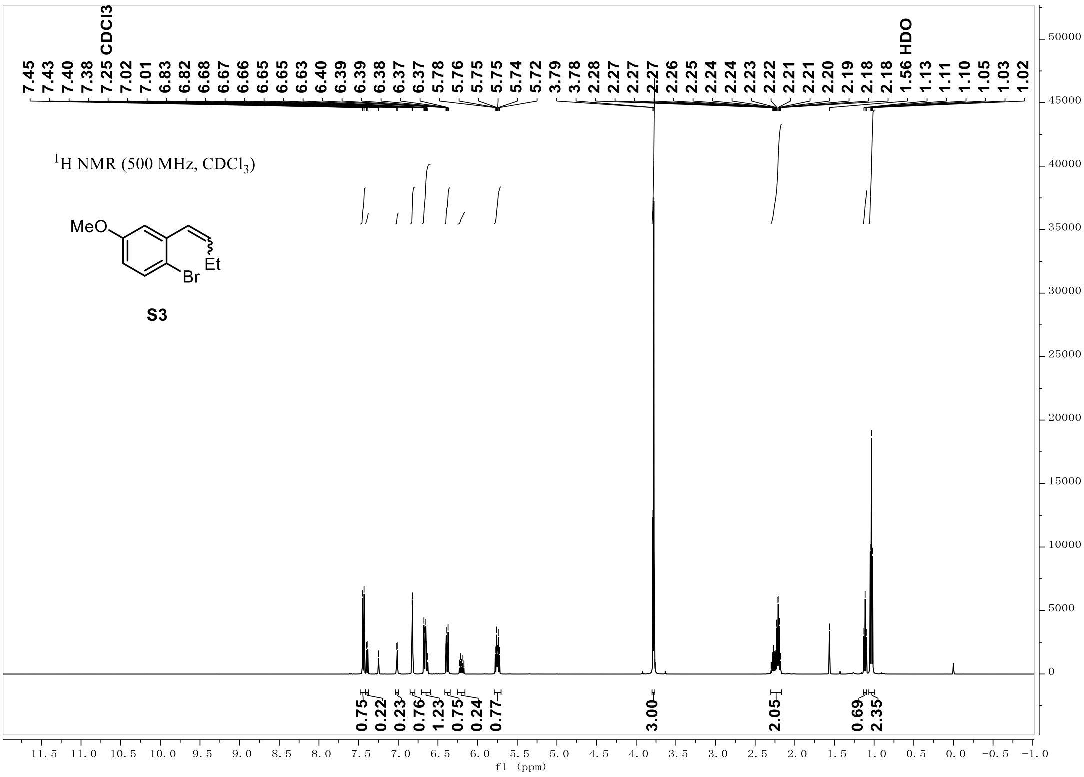

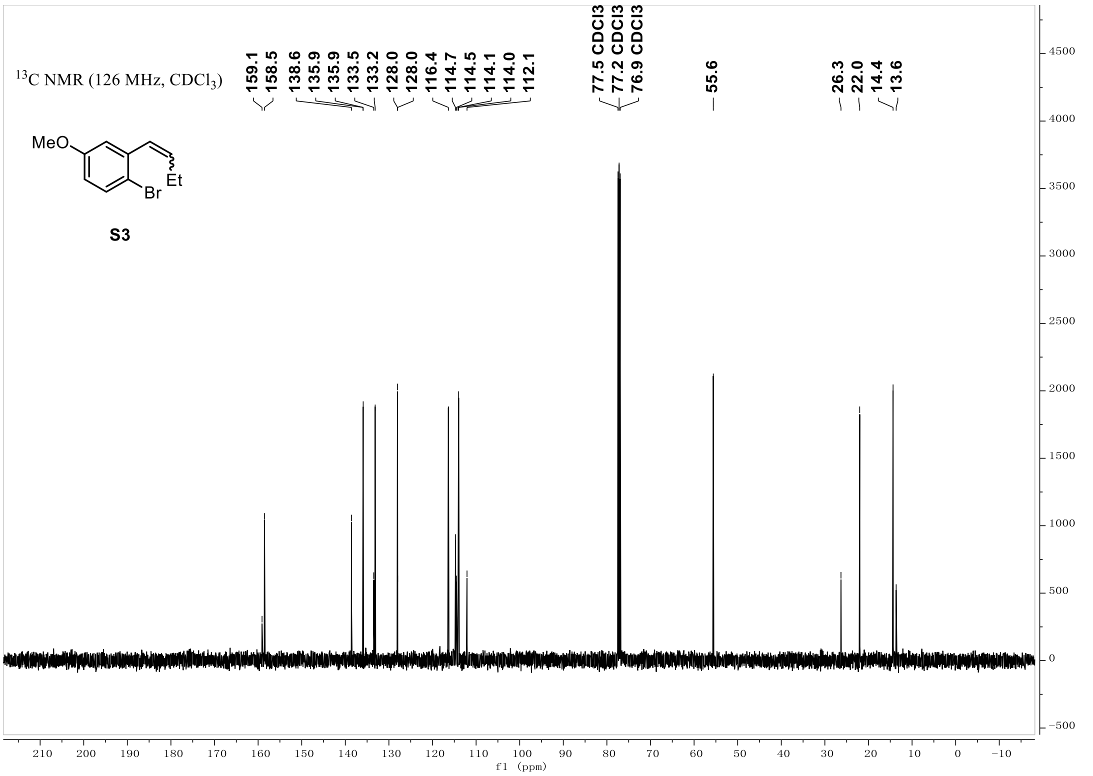

Supplementary Figure 21. ^{1}H NMR and ^{13}C NMR spectra of compound S3.

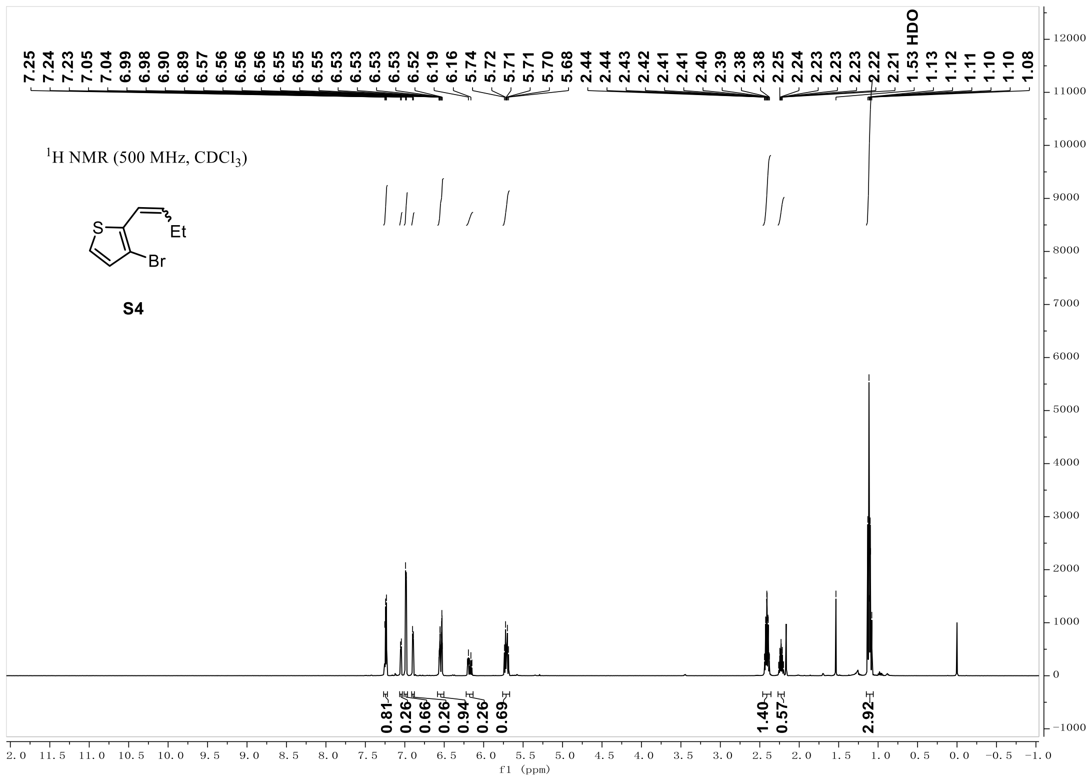

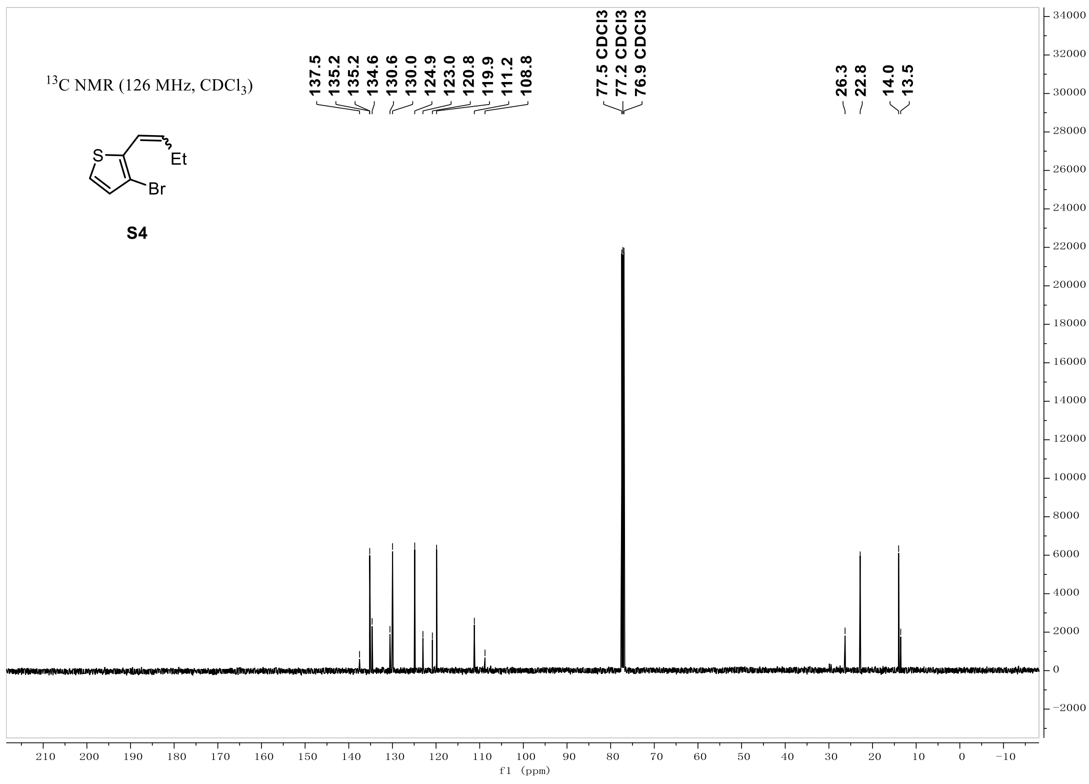

Supplementary Figure 22. ^{1}H NMR and ^{13}C NMR spectra of compound S4.

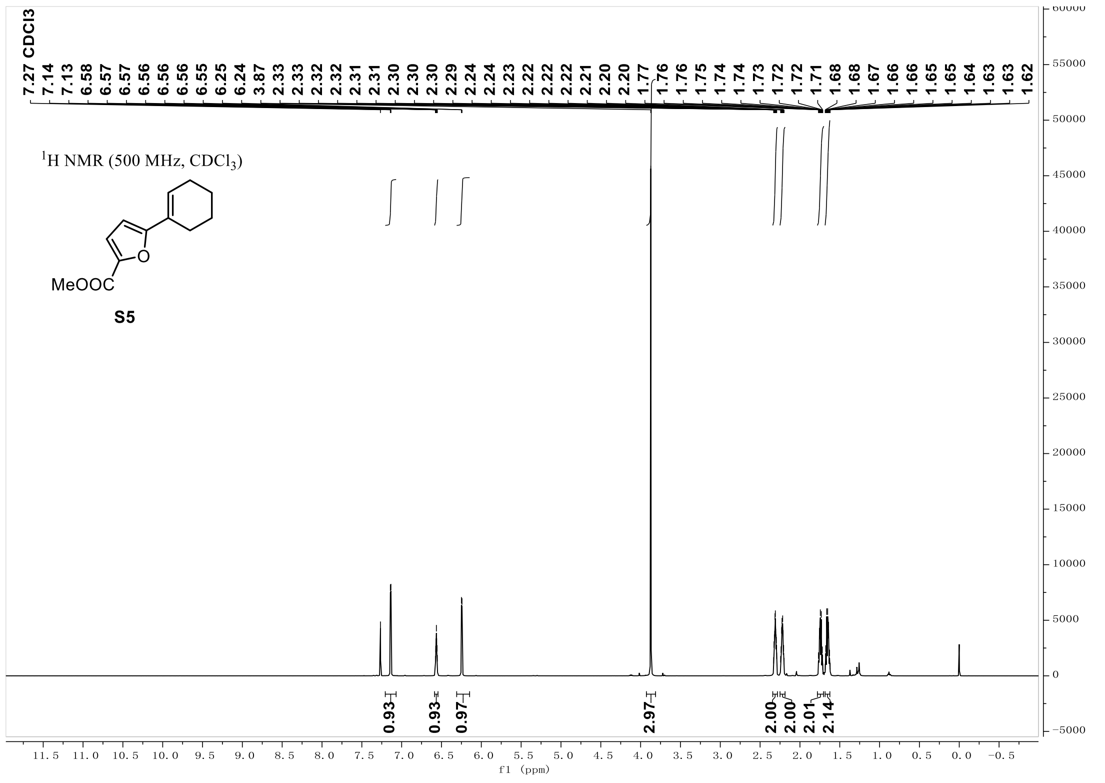

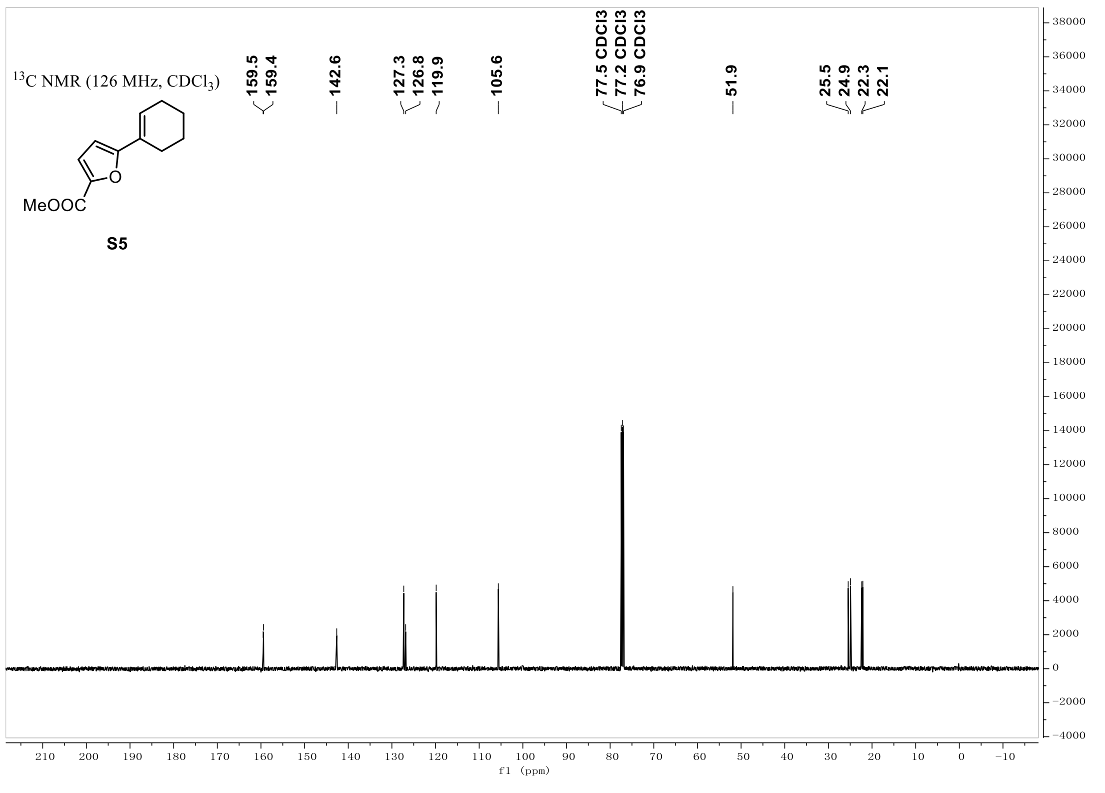

Supplementary Figure 23. ^{1}H NMR and ^{13}C NMR spectra of compound S5.

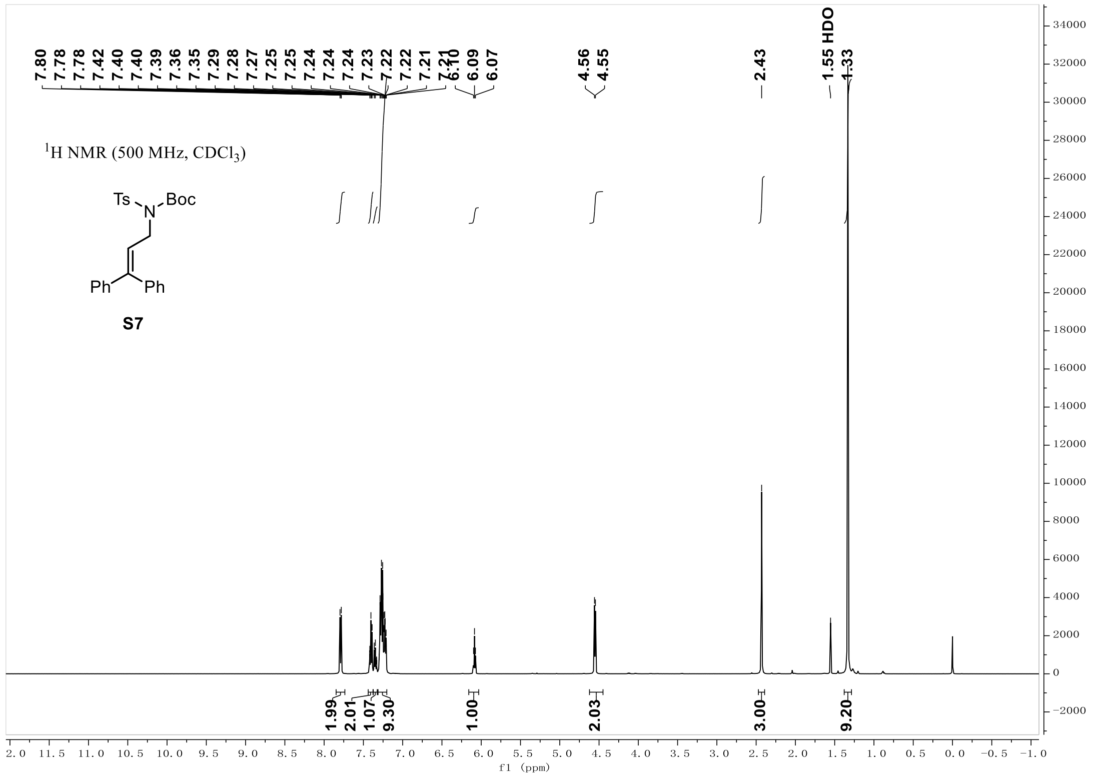

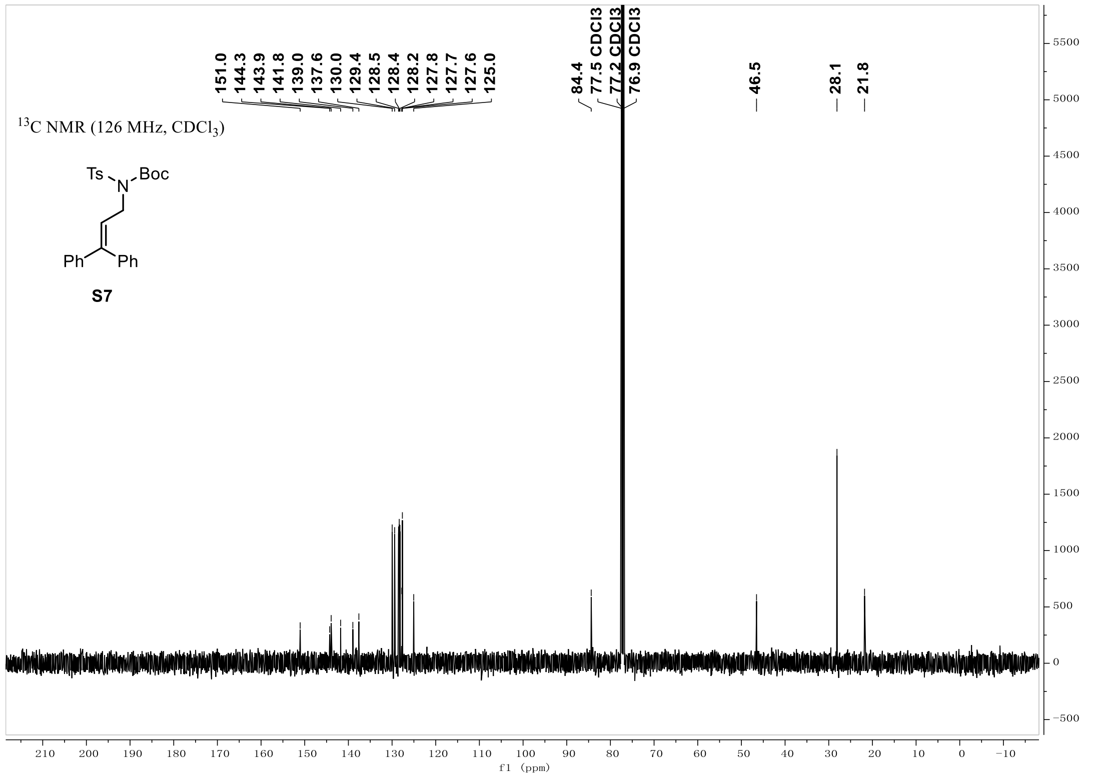

Supplementary Figure 24. ^{1}H NMR and ^{13}C NMR spectra of compound S7.

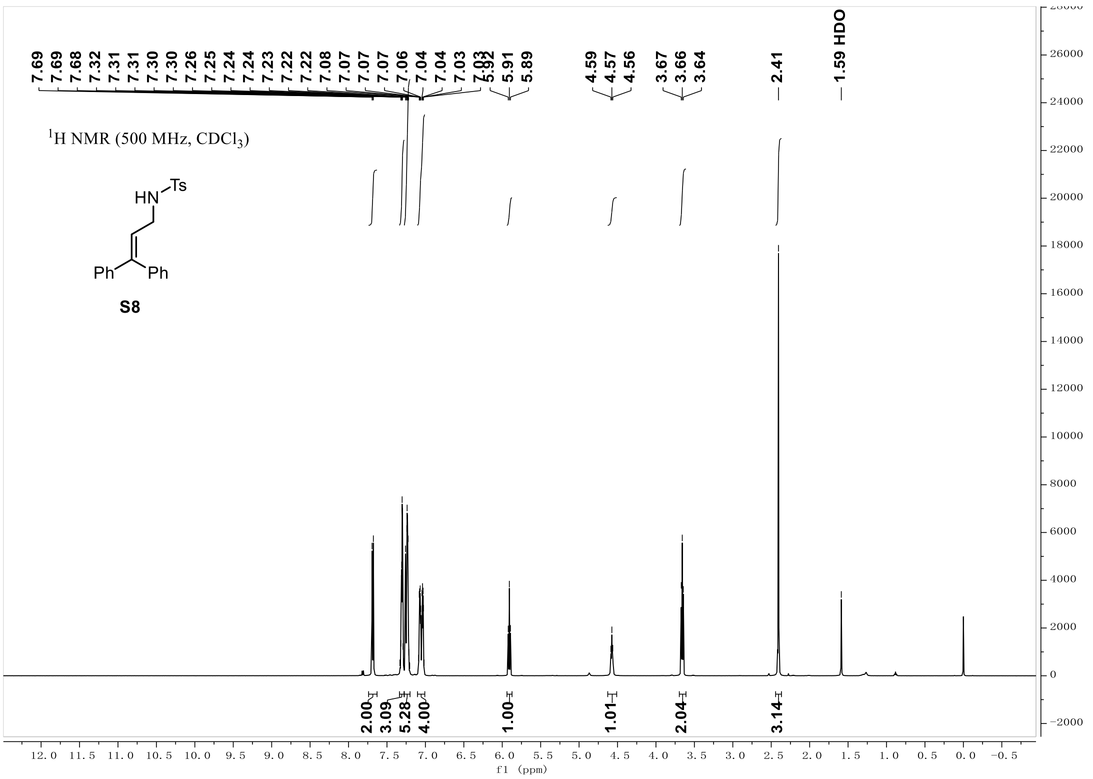

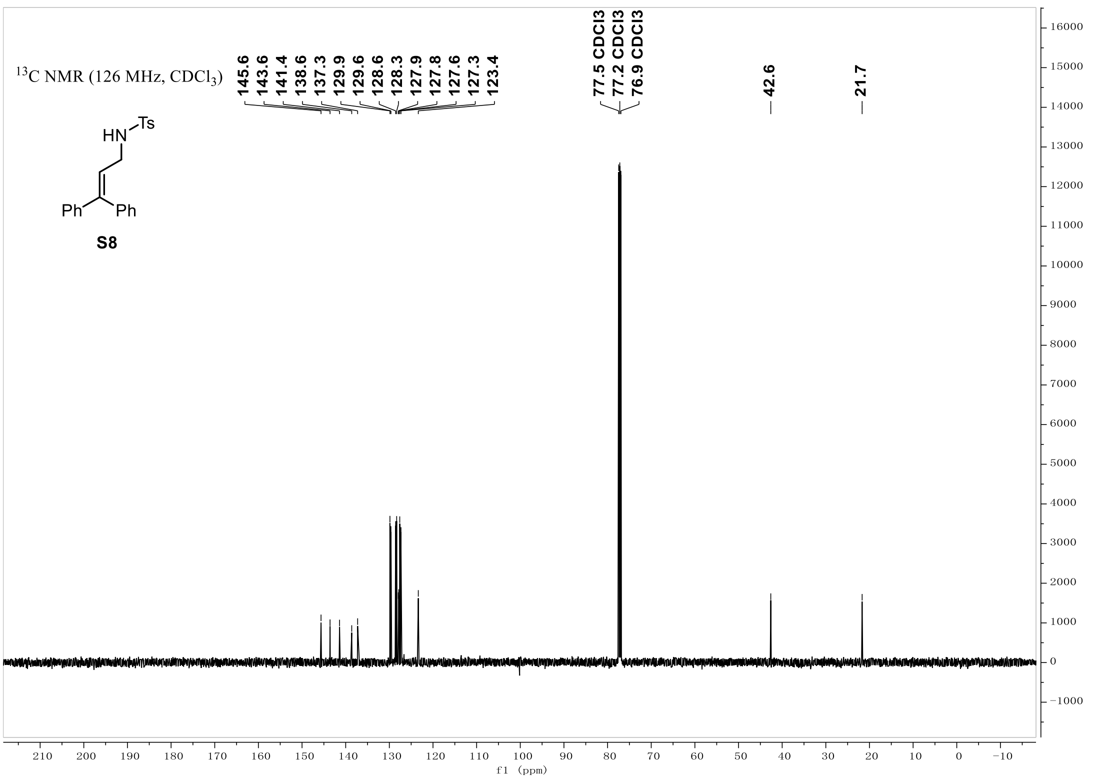

Supplementary Figure 25. ^{1}H NMR and ^{13}C NMR spectra of compound S8.

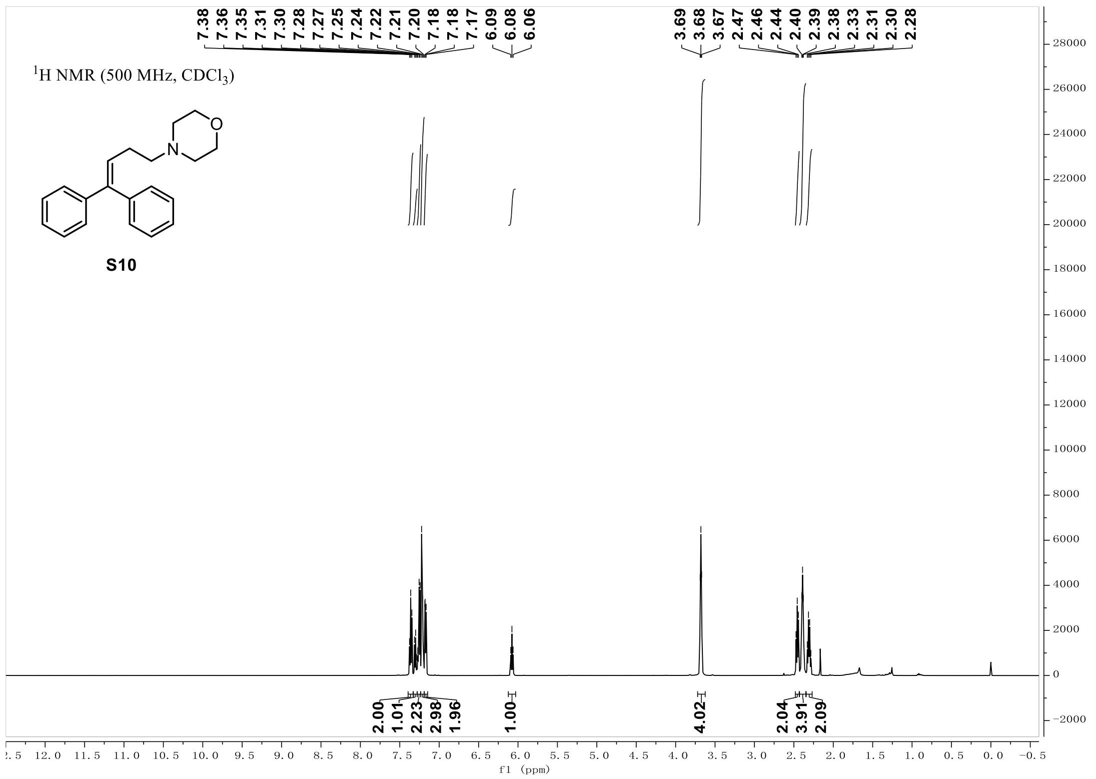

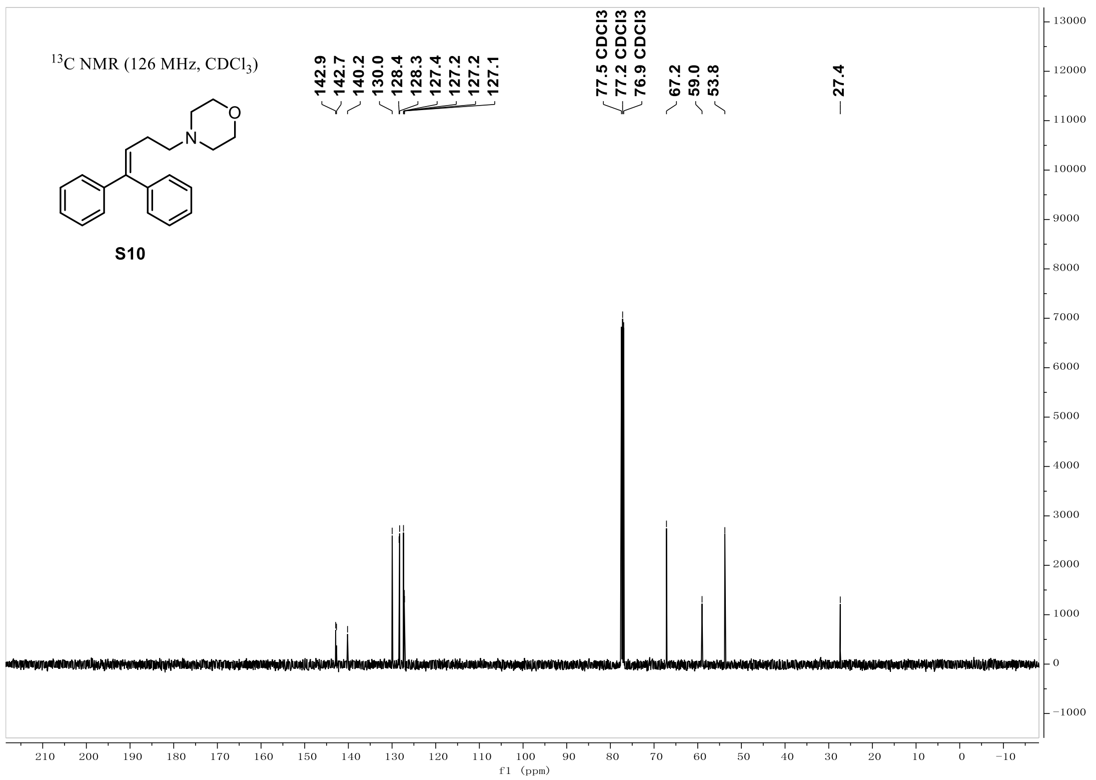

Supplementary Figure 26. ^{1}H NMR and ^{13}C NMR spectra of compound S10.

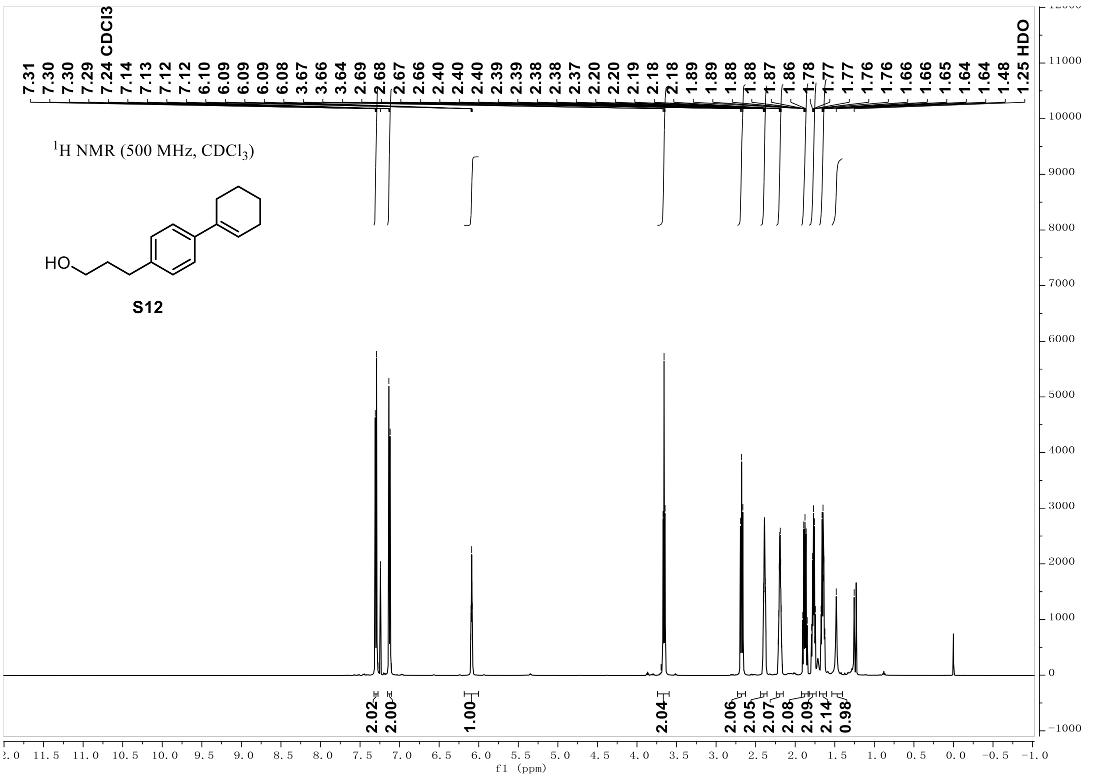

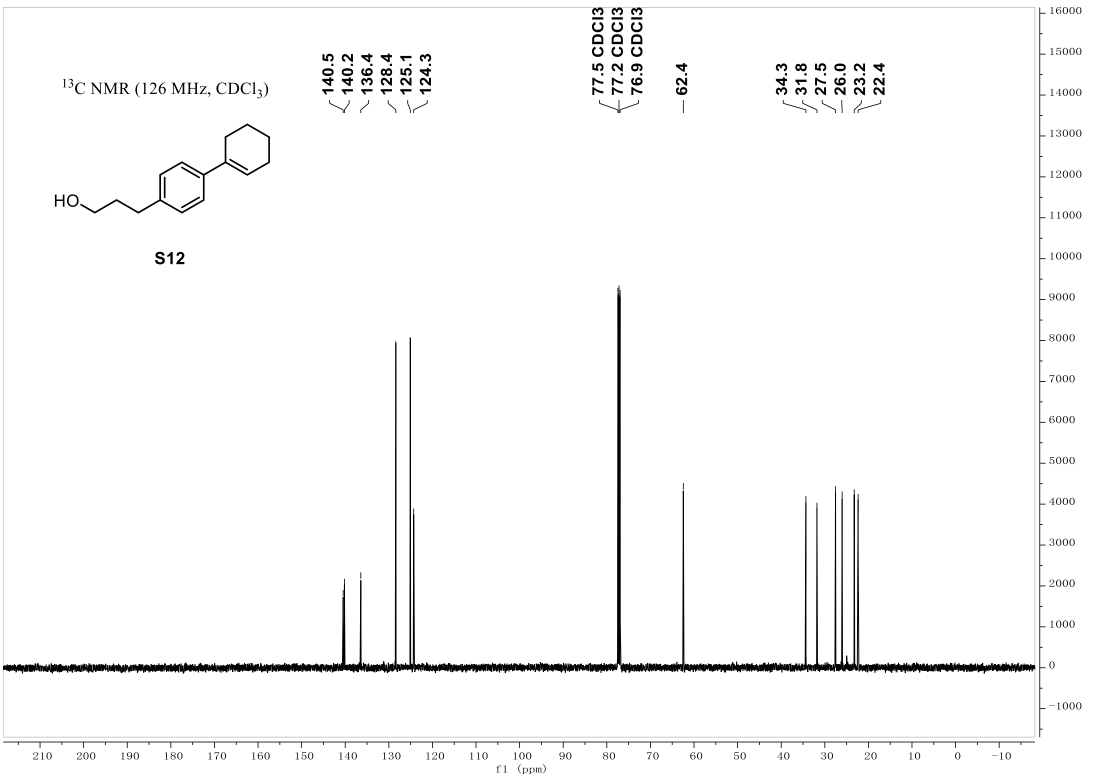

Supplementary Figure 27. ^{1}H NMR and ^{13}C NMR spectra of compound S12.

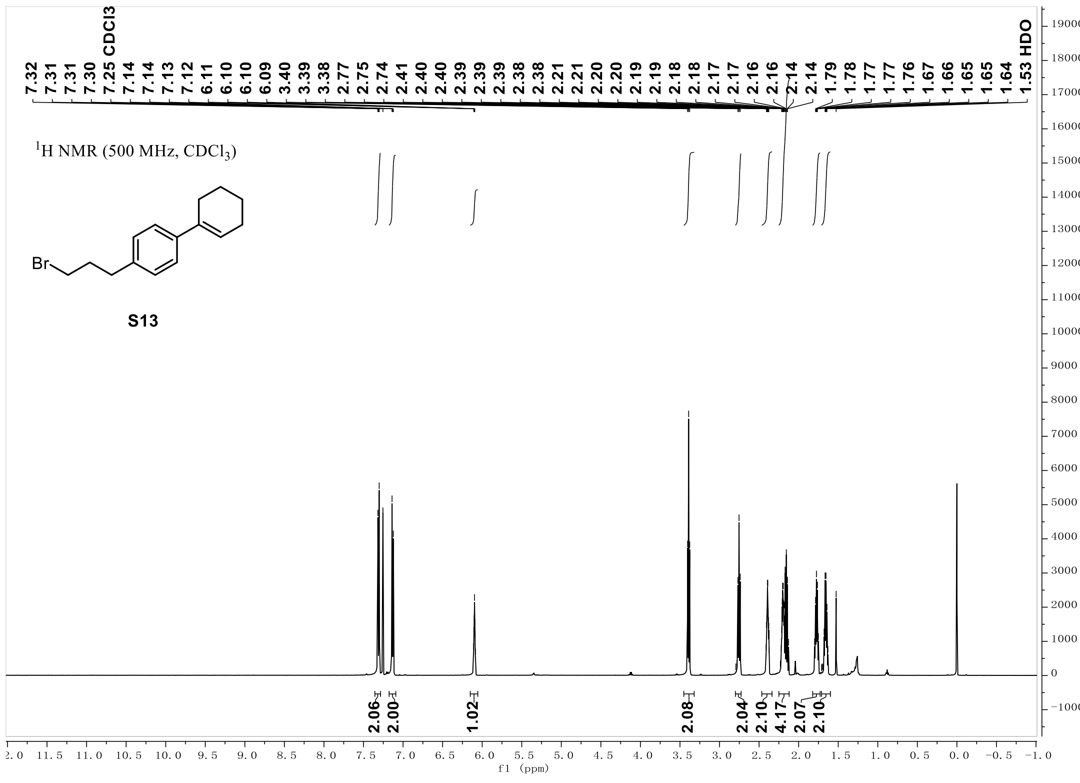

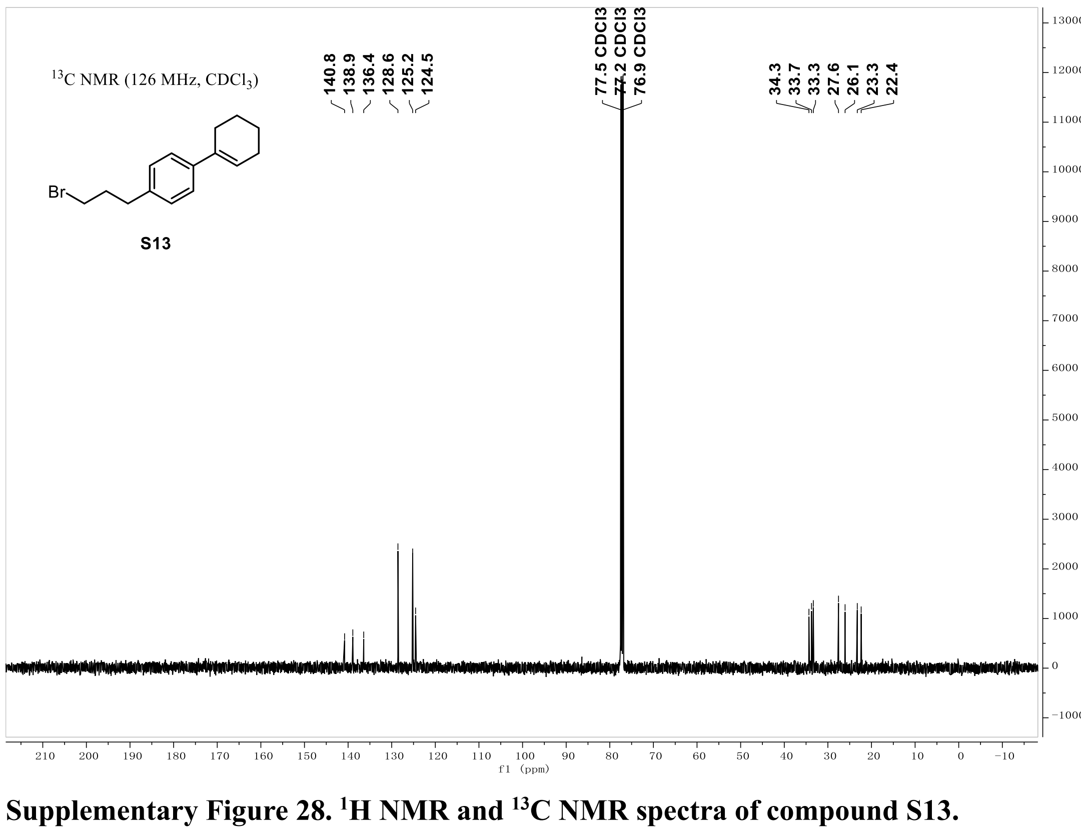

Supplementary Figure 28. ^{1}H NMR and ^{13}C NMR spectra of compound S13.

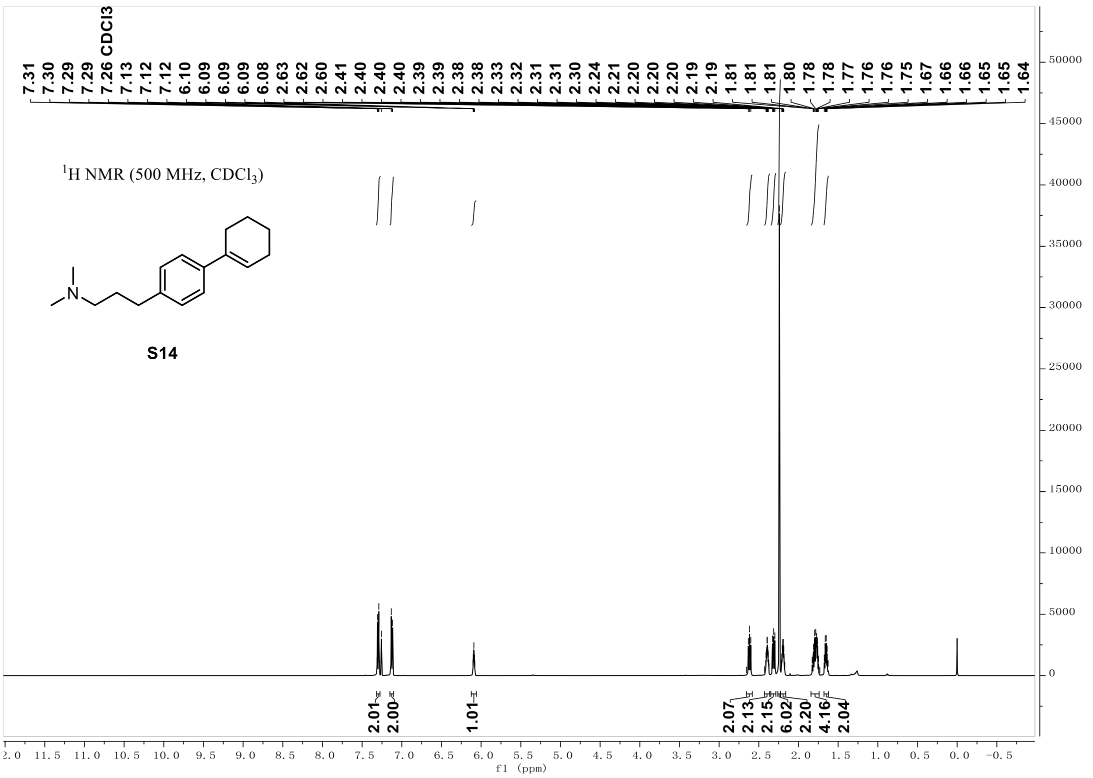

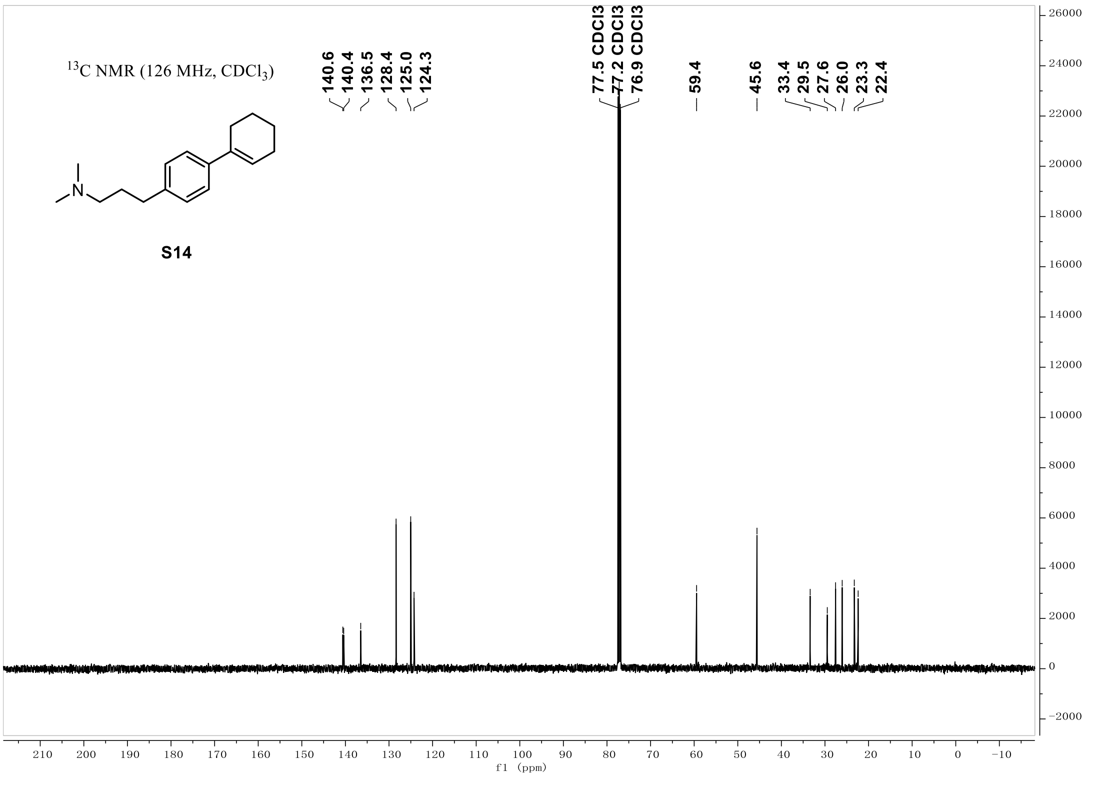

Supplementary Figure 29. ^{1}H NMR and ^{13}C NMR spectra of compound S14.

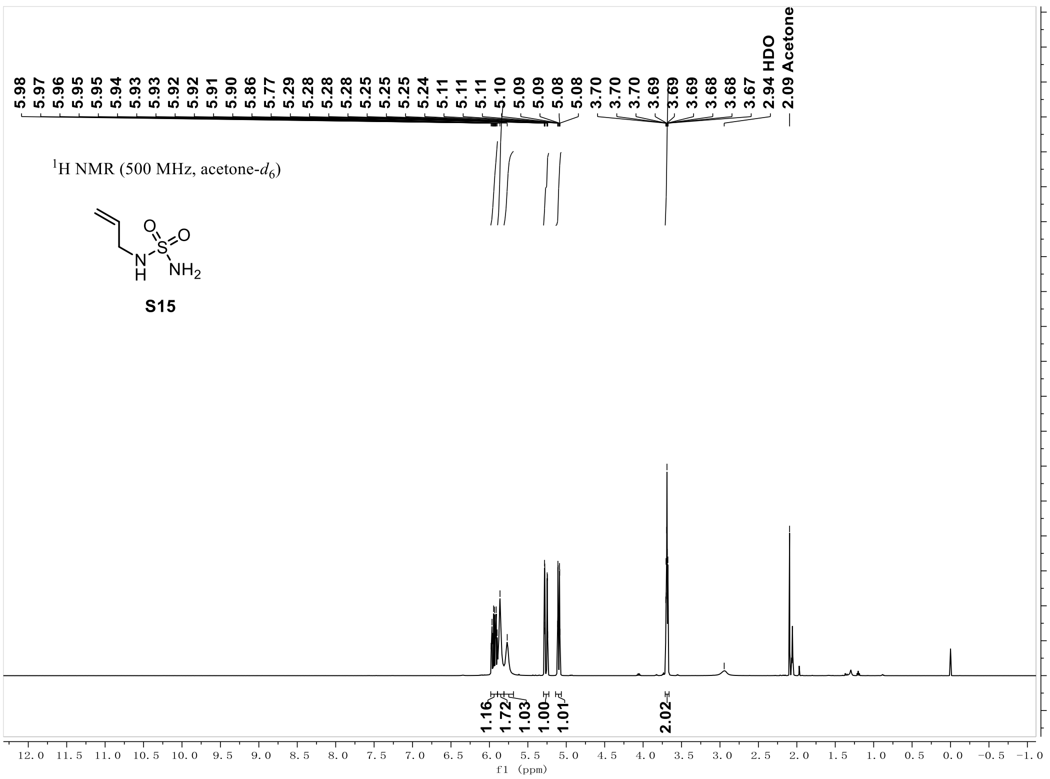

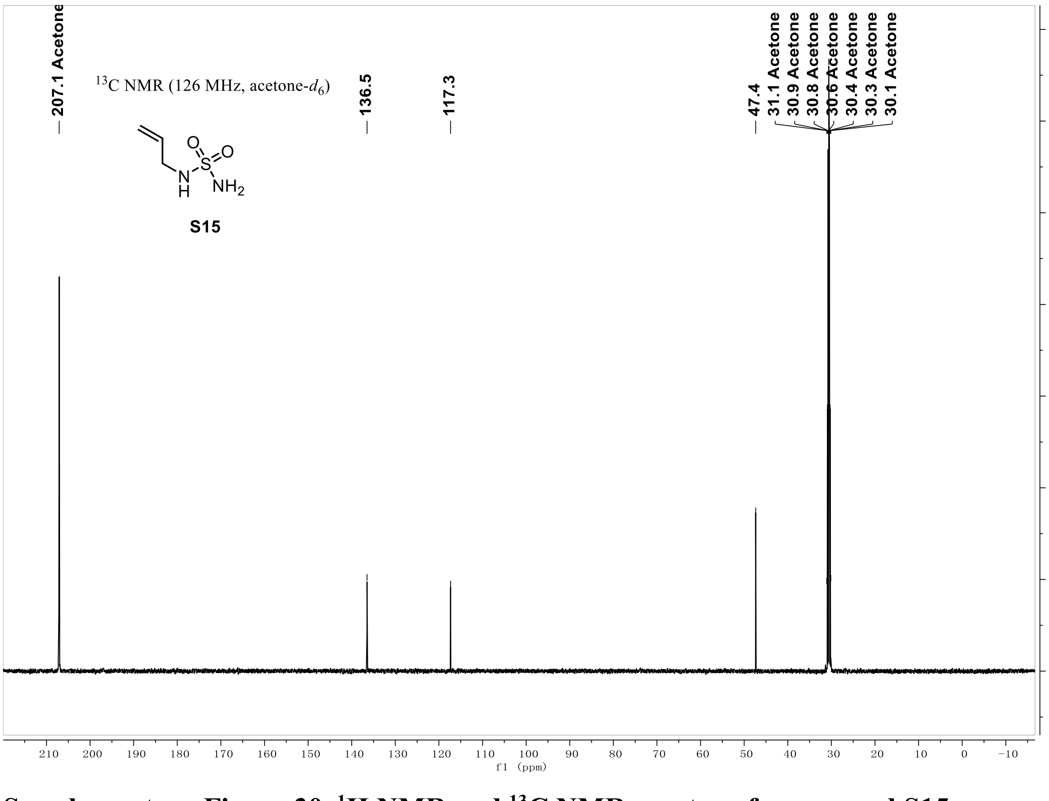

Supplementary Figure 30. ^{1}H NMR and ^{13}C NMR spectra of compound S15.

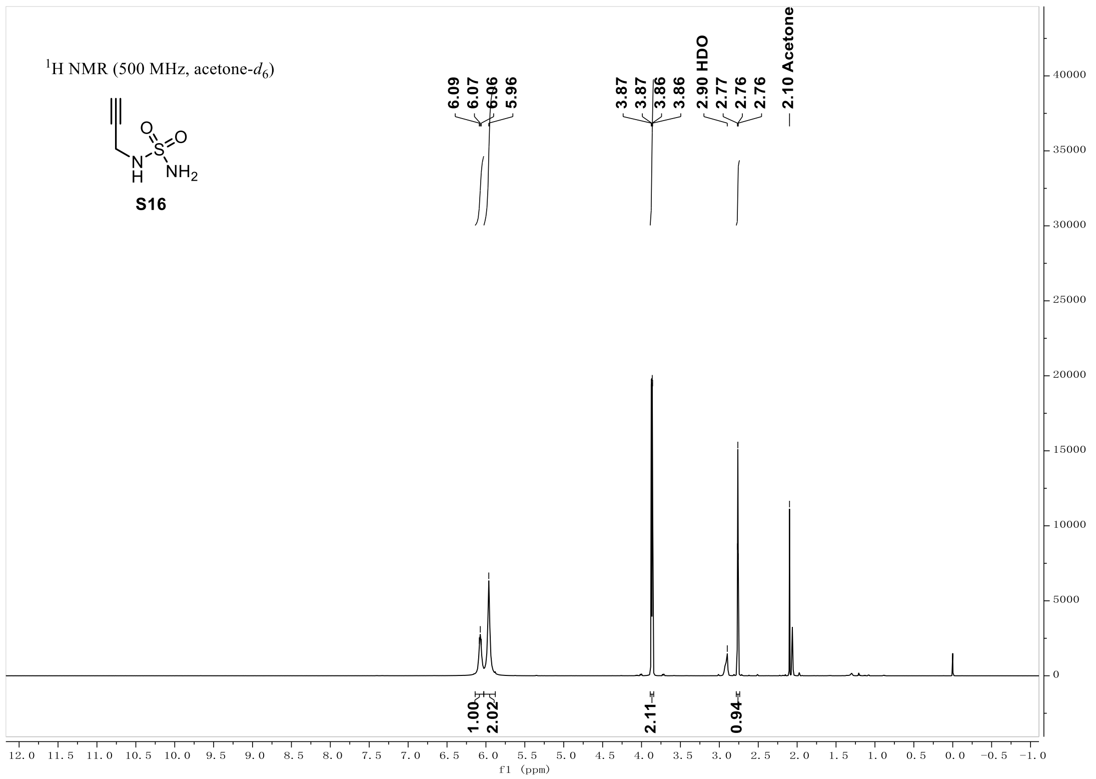

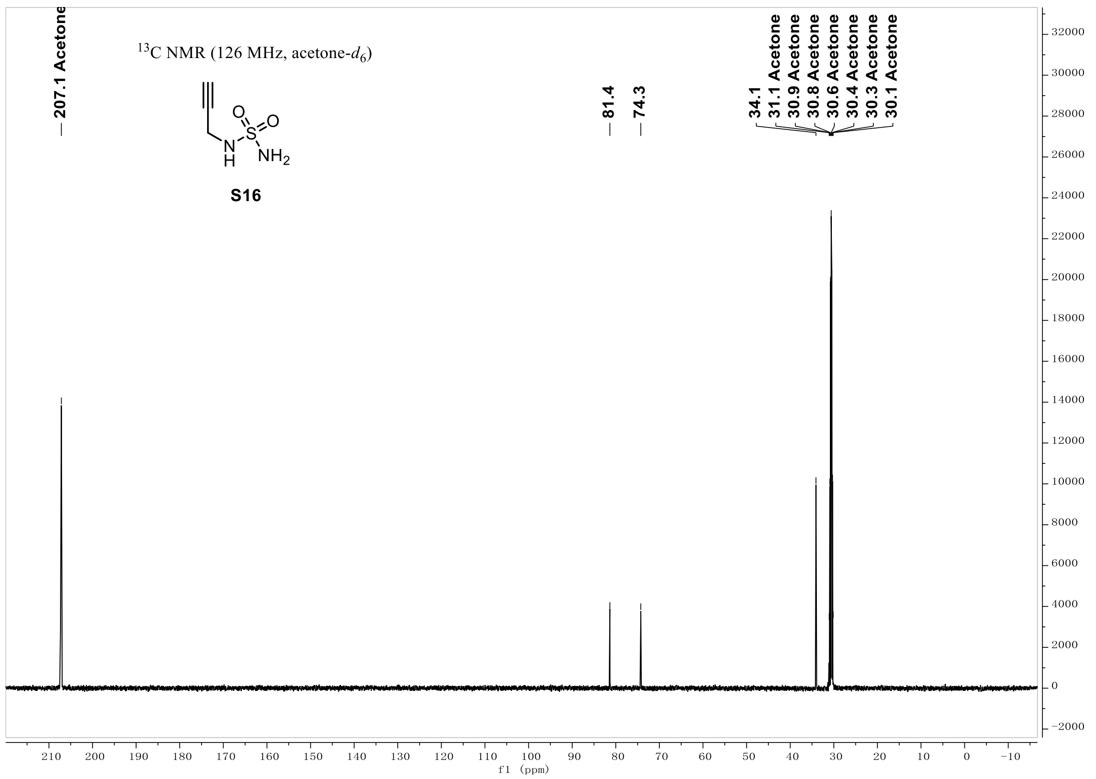

Supplementary Figure 31. ^{1}H NMR and ^{13}C NMR spectra of compound S16.

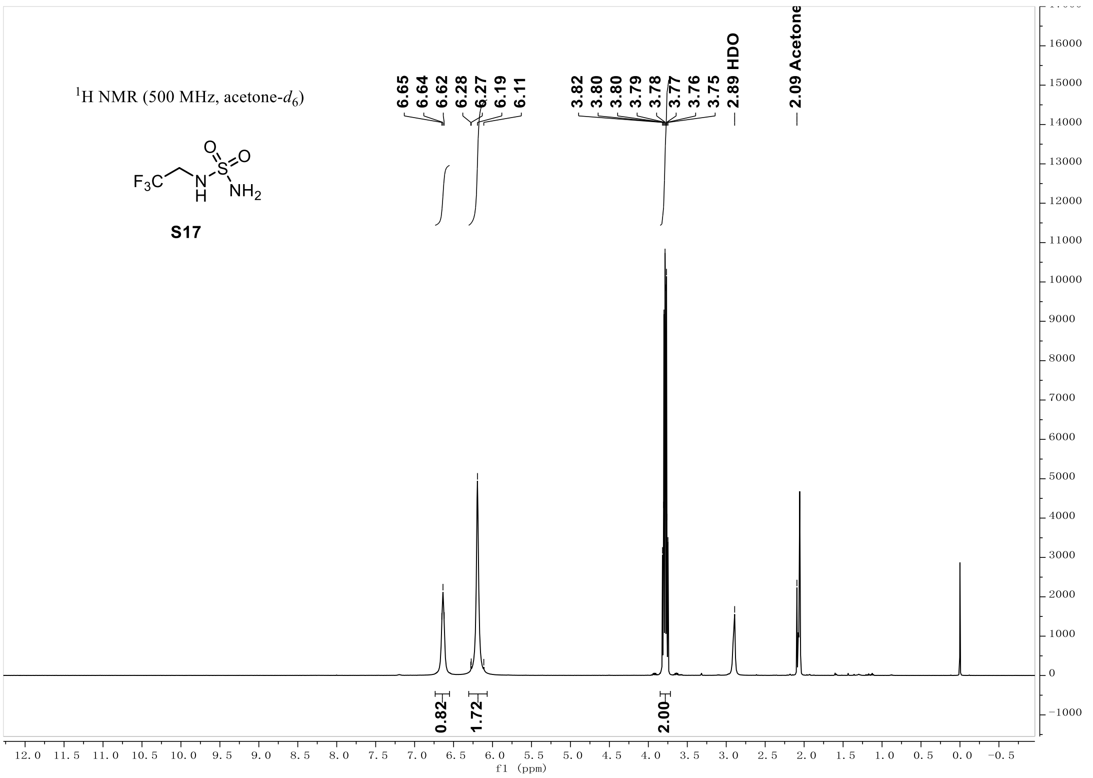

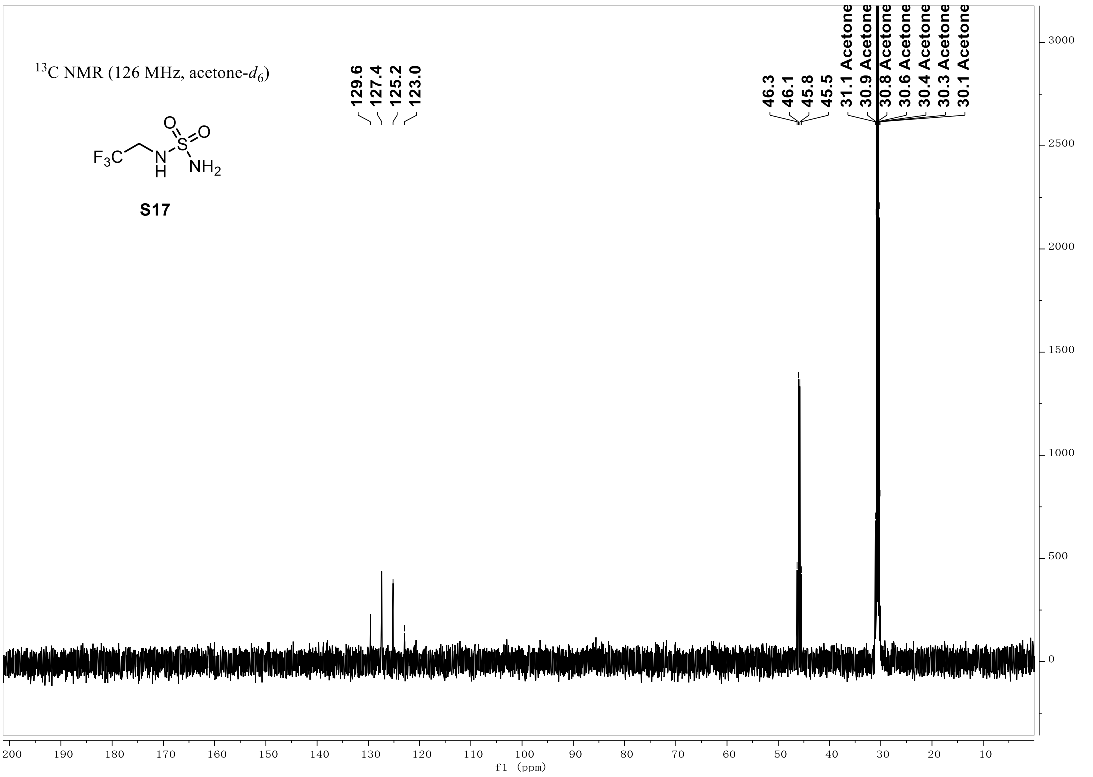

Supplementary Figure 32. ^{1}H NMR and ^{13}C NMR spectra of compound S17.

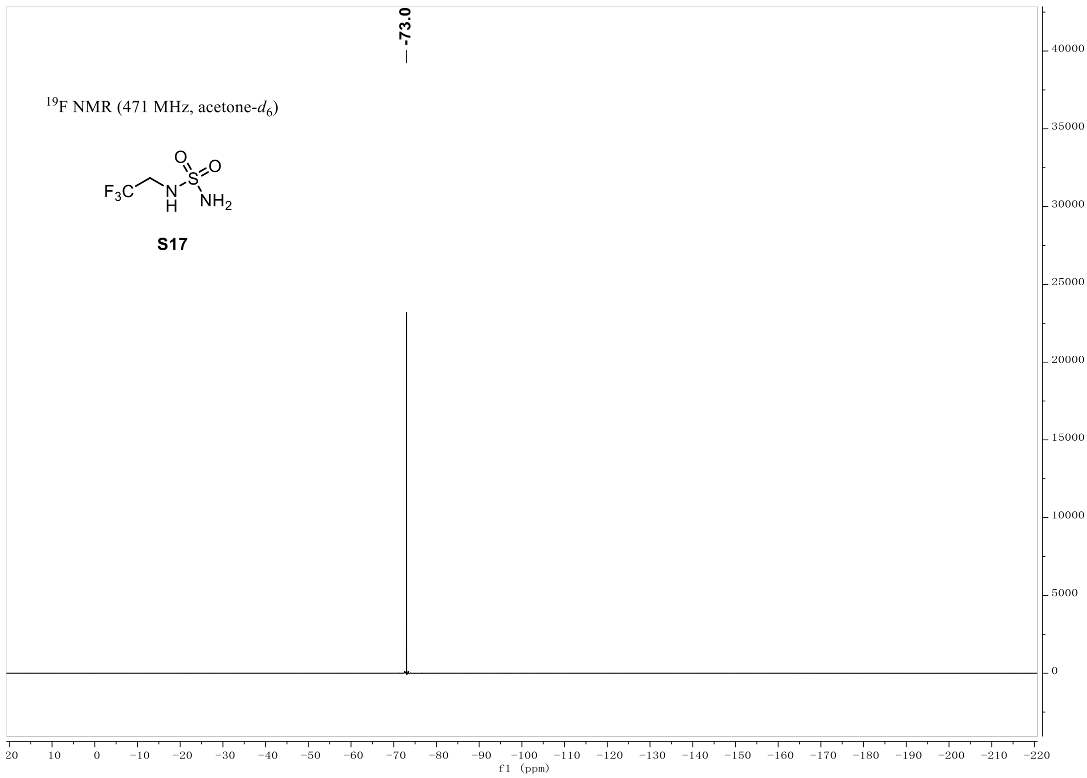

Supplementary Figure 33. ^{19}F NMR spectra of compound S17.

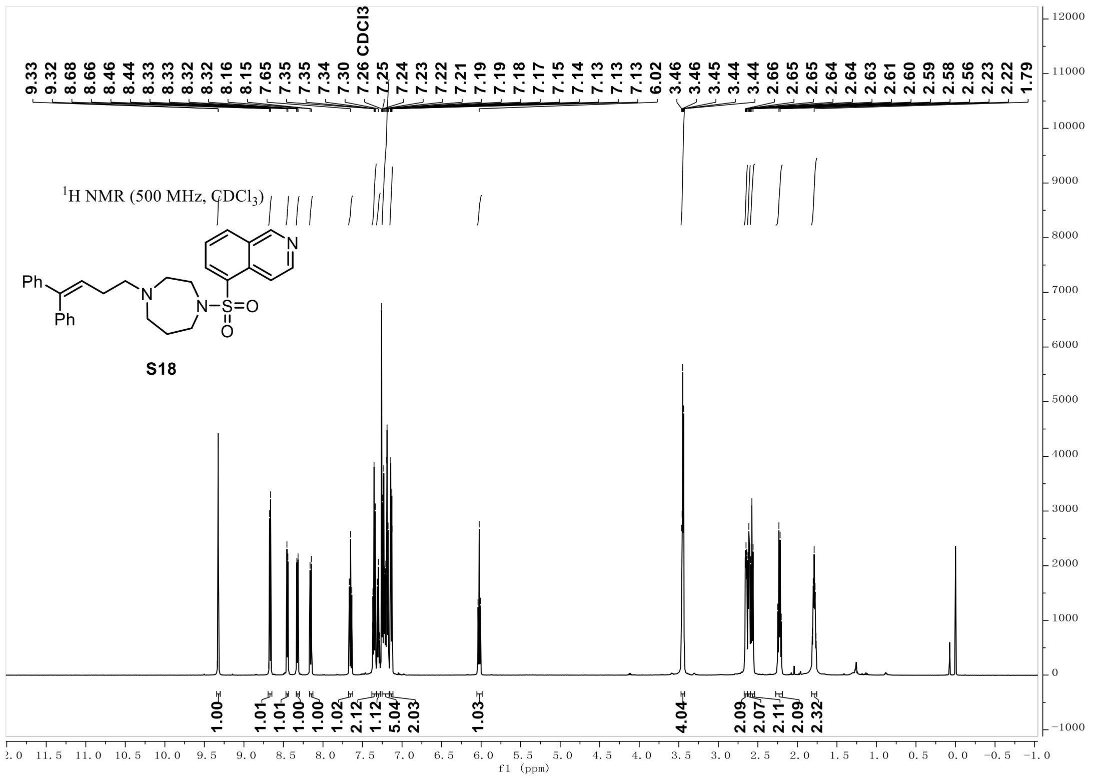

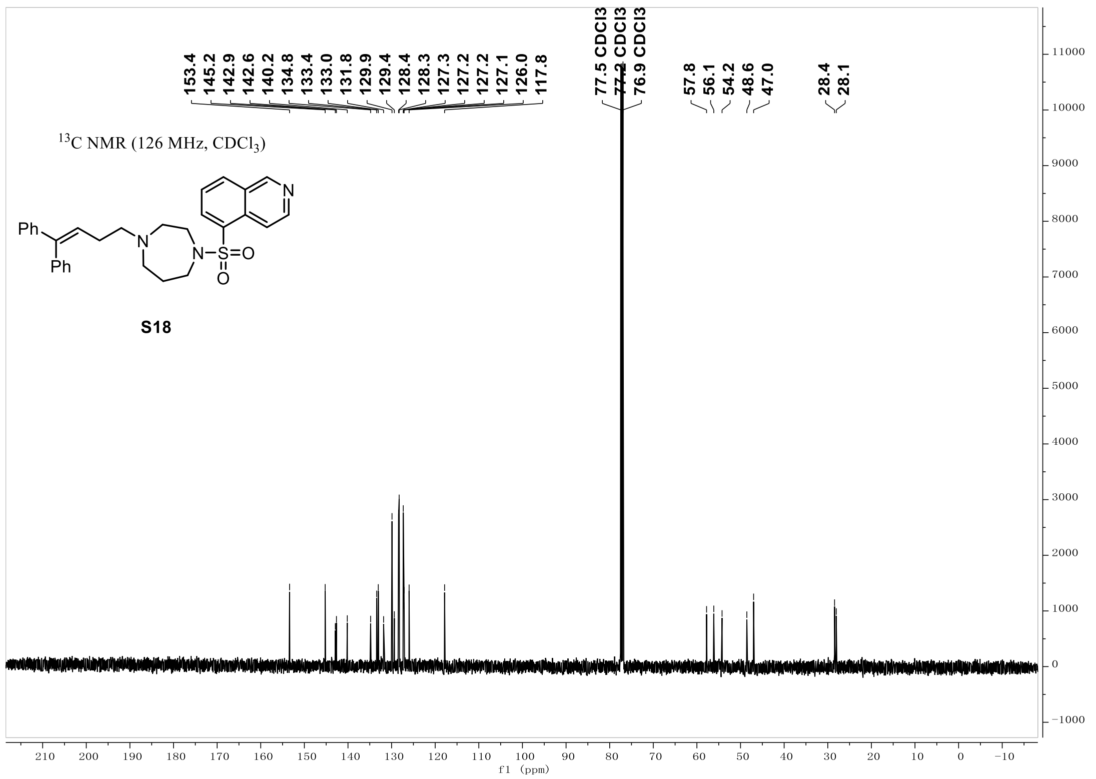

Supplementary Figure 34. ^{1}H NMR and ^{13}C NMR spectra of compound S18.

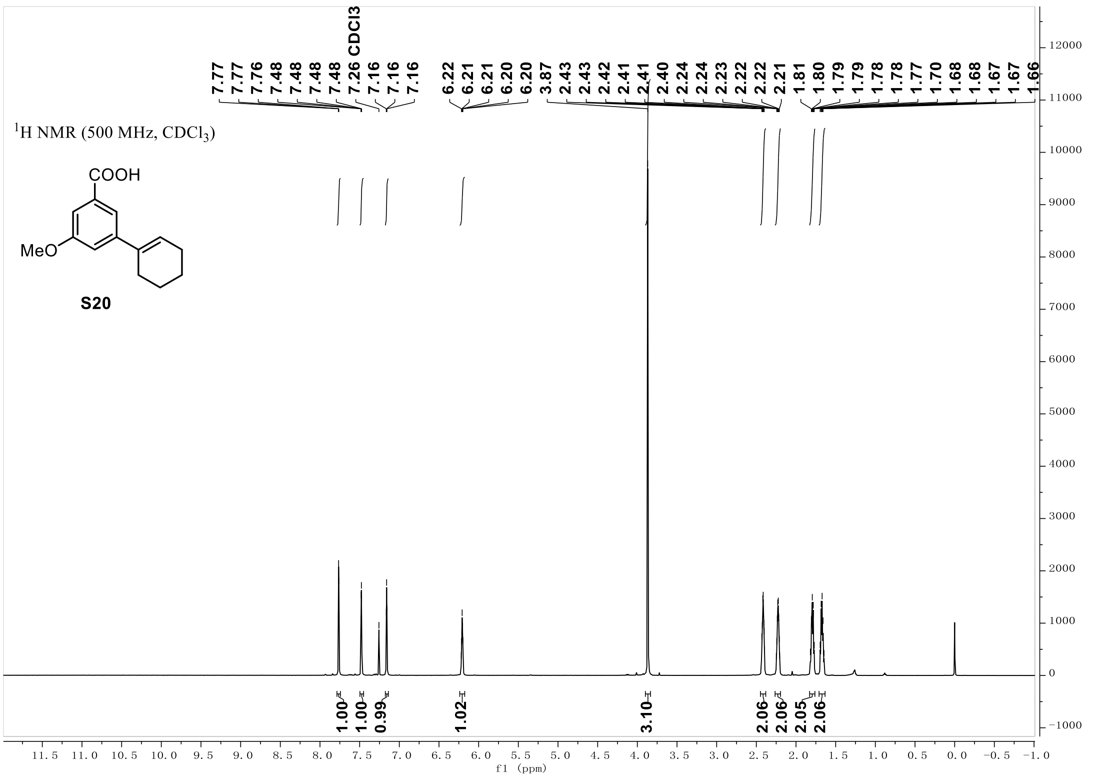

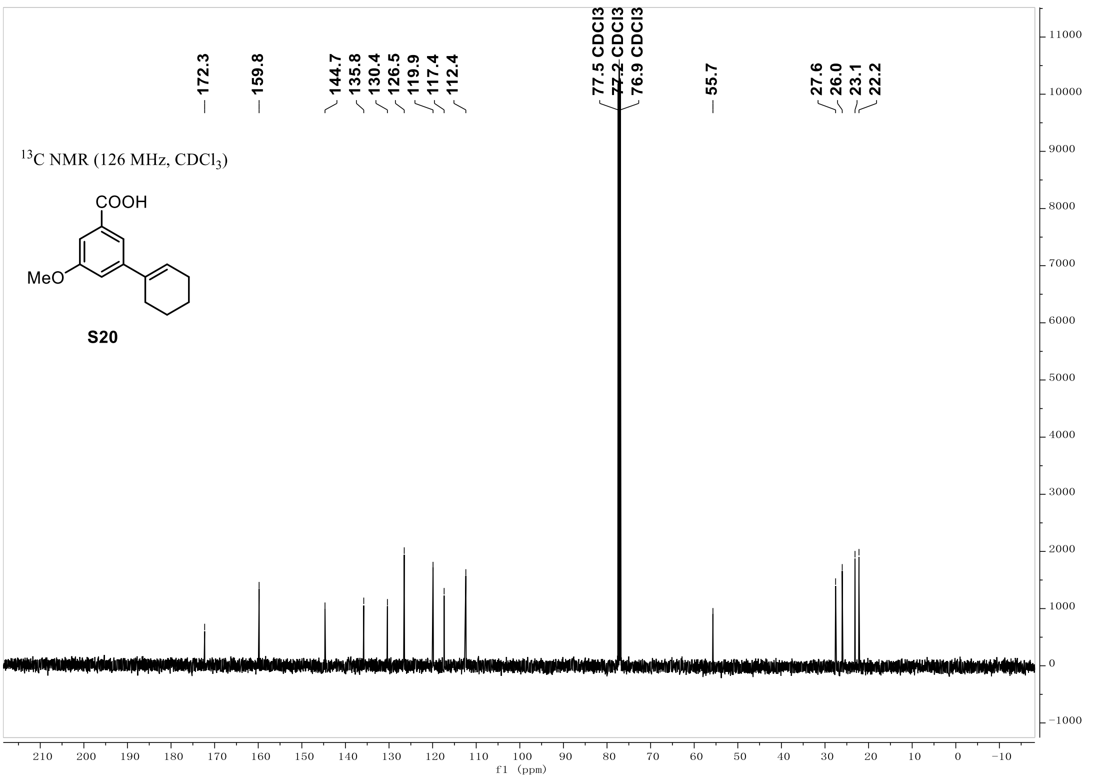

Supplementary Figure 35. ^{1}H NMR and ^{13}C NMR spectra of compound S20.

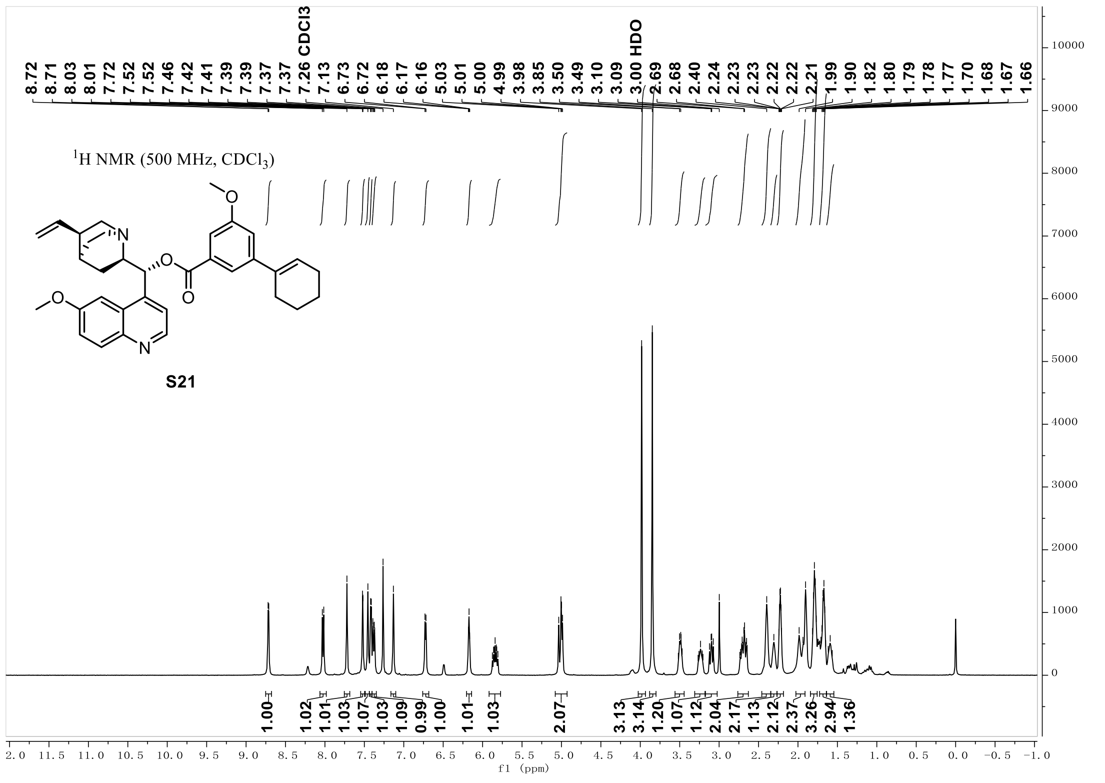

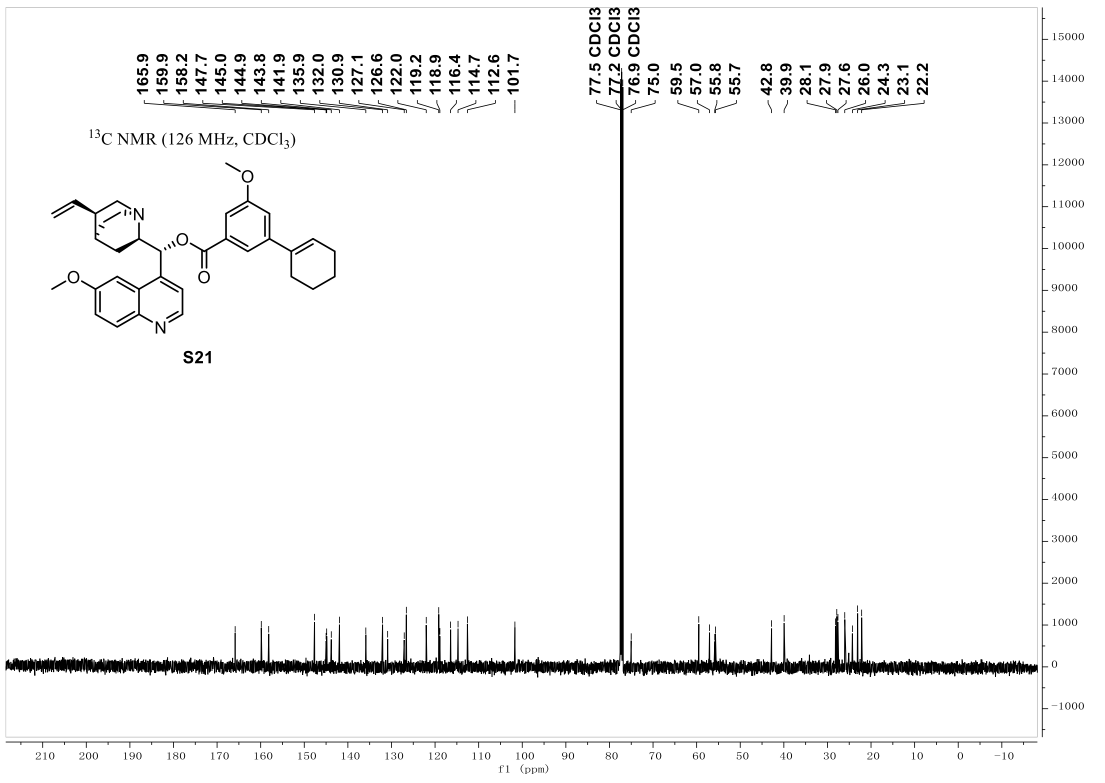

Supplementary Figure 36. ^{1}H NMR and ^{13}C NMR spectra of compound S21.

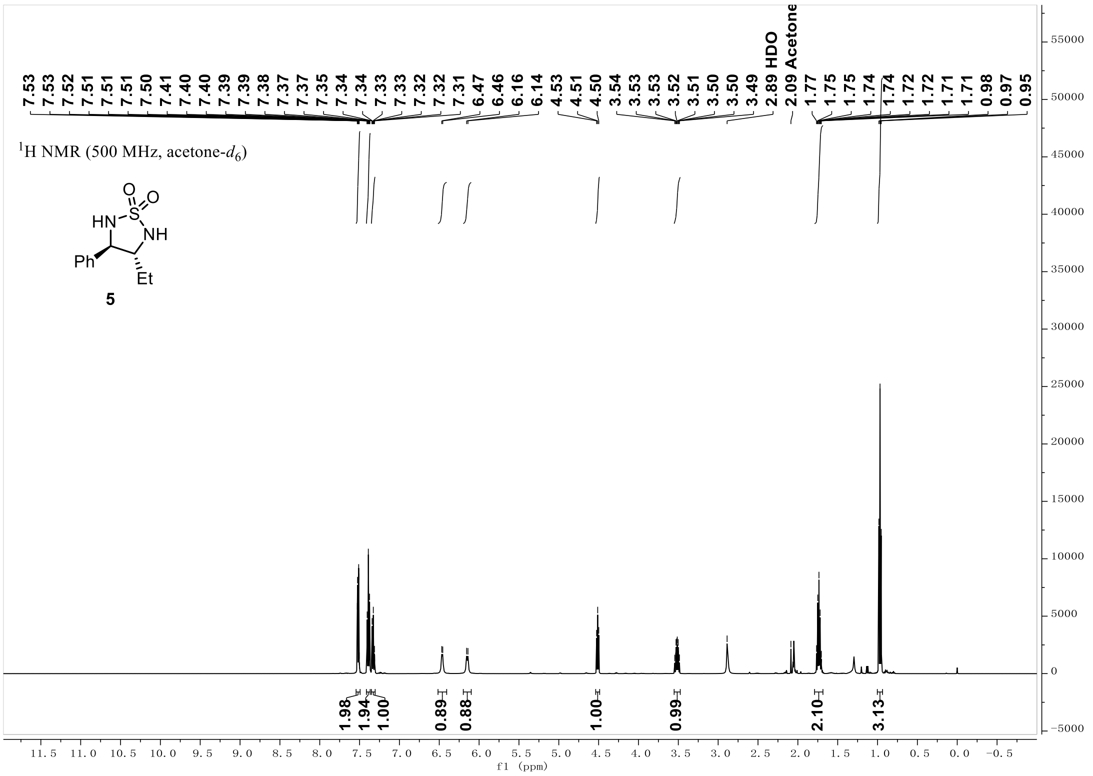

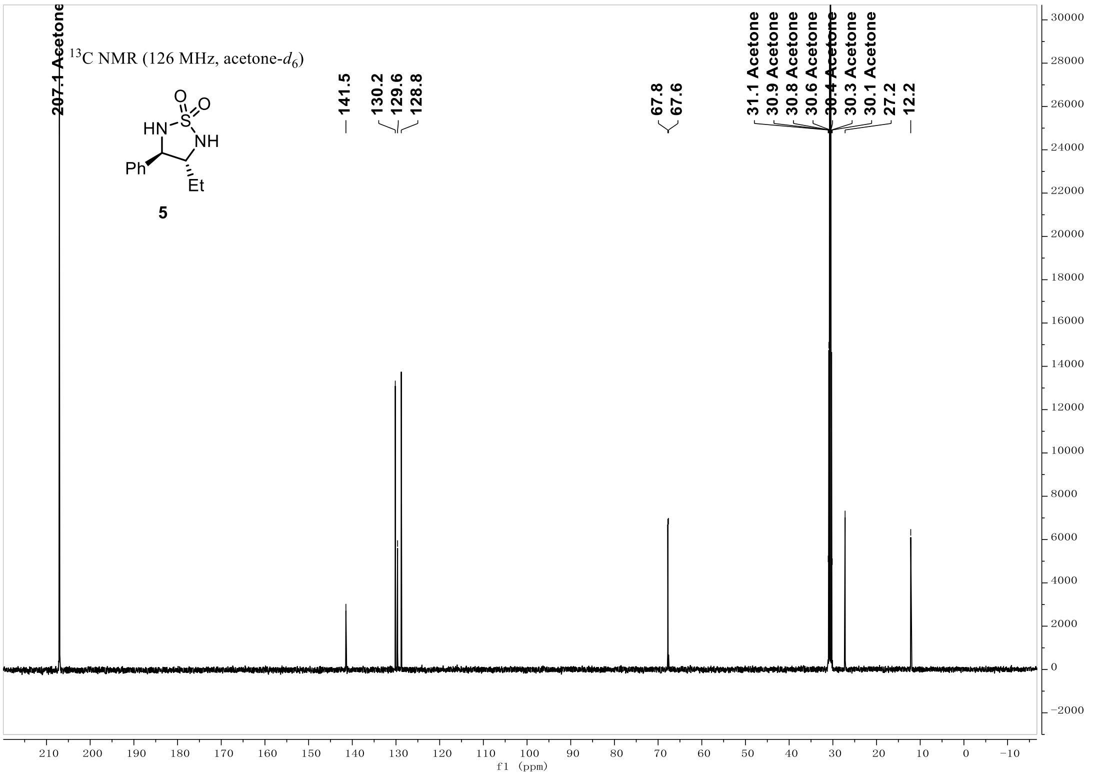

Supplementary Figure 37. ^{1}H NMR and ^{13}C NMR spectra of compound 5.

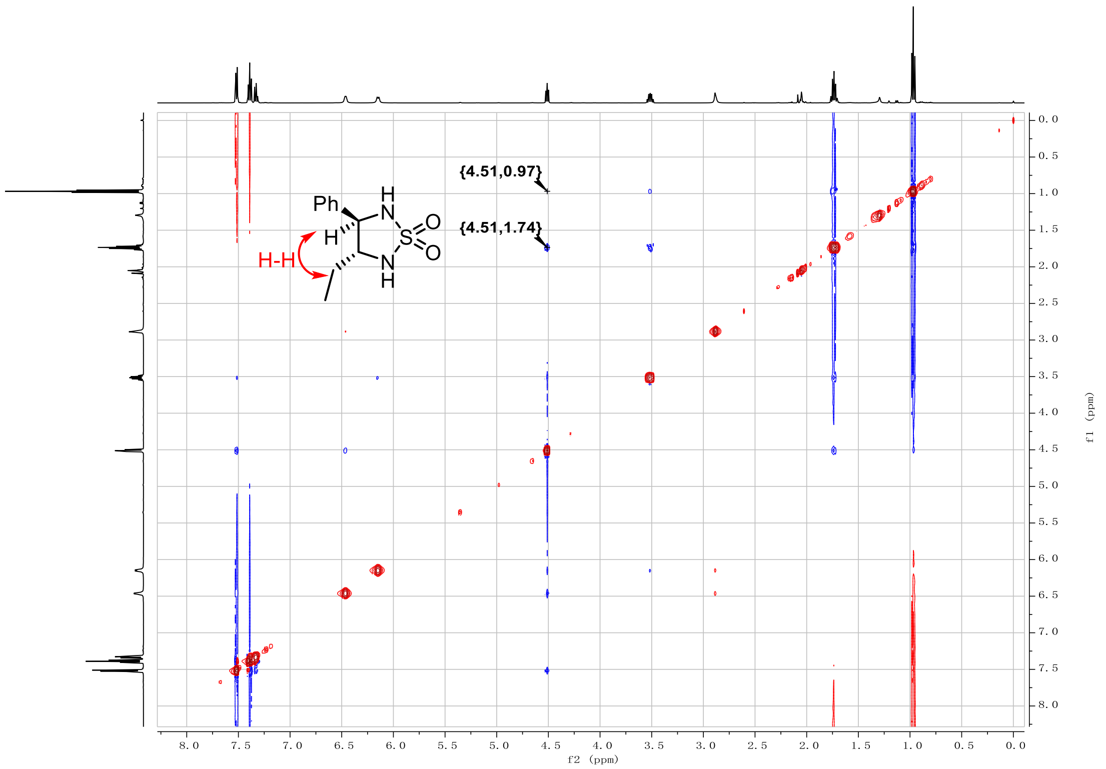

Supplementary Figure 38. 2D NOESY spectrum of compound 5.

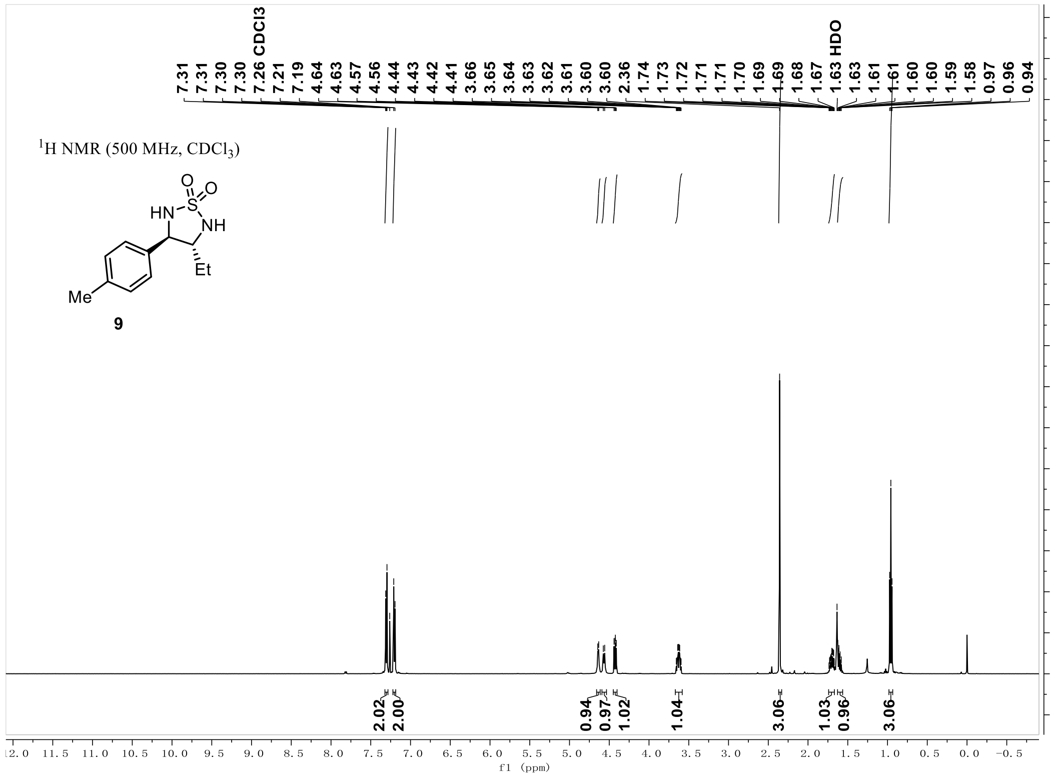

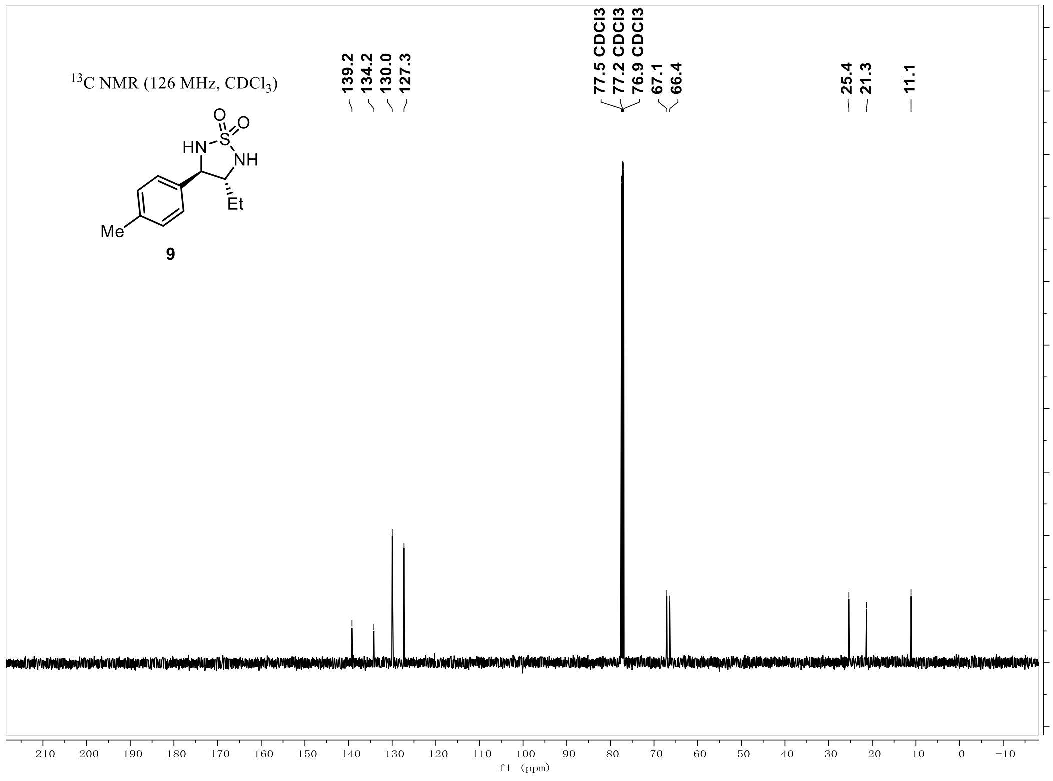

Supplementary Figure 39. ^{1}H NMR and ^{13}C NMR spectra of compound 9.

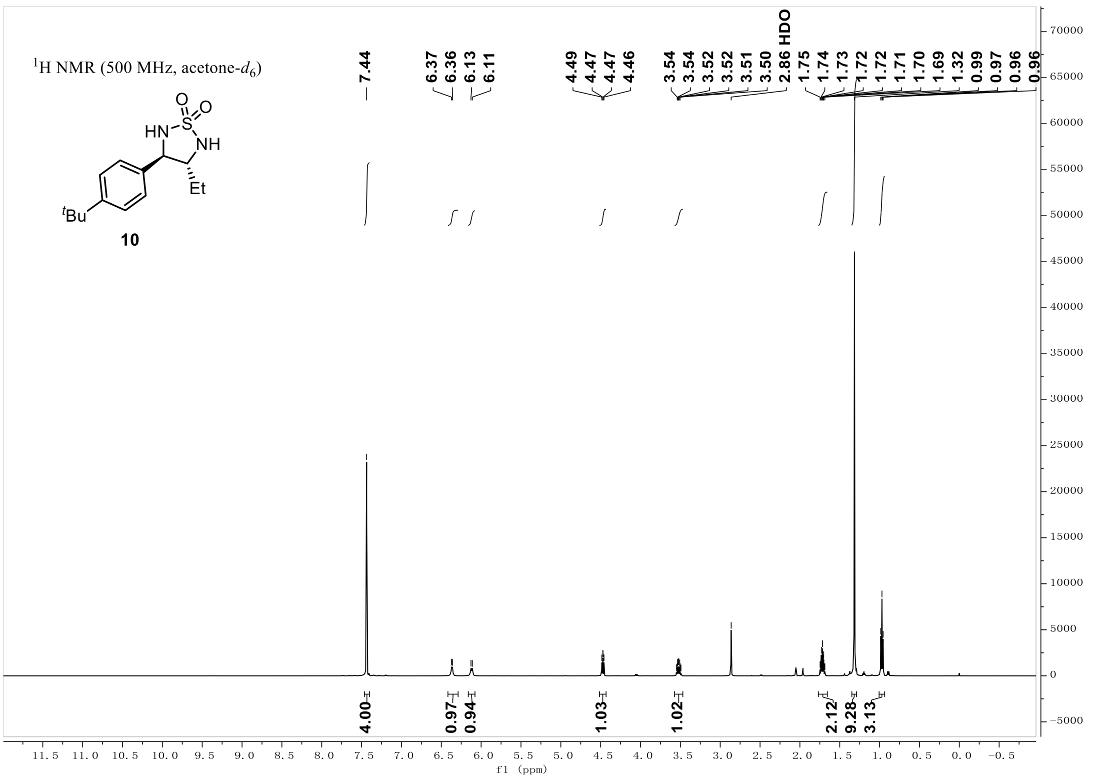

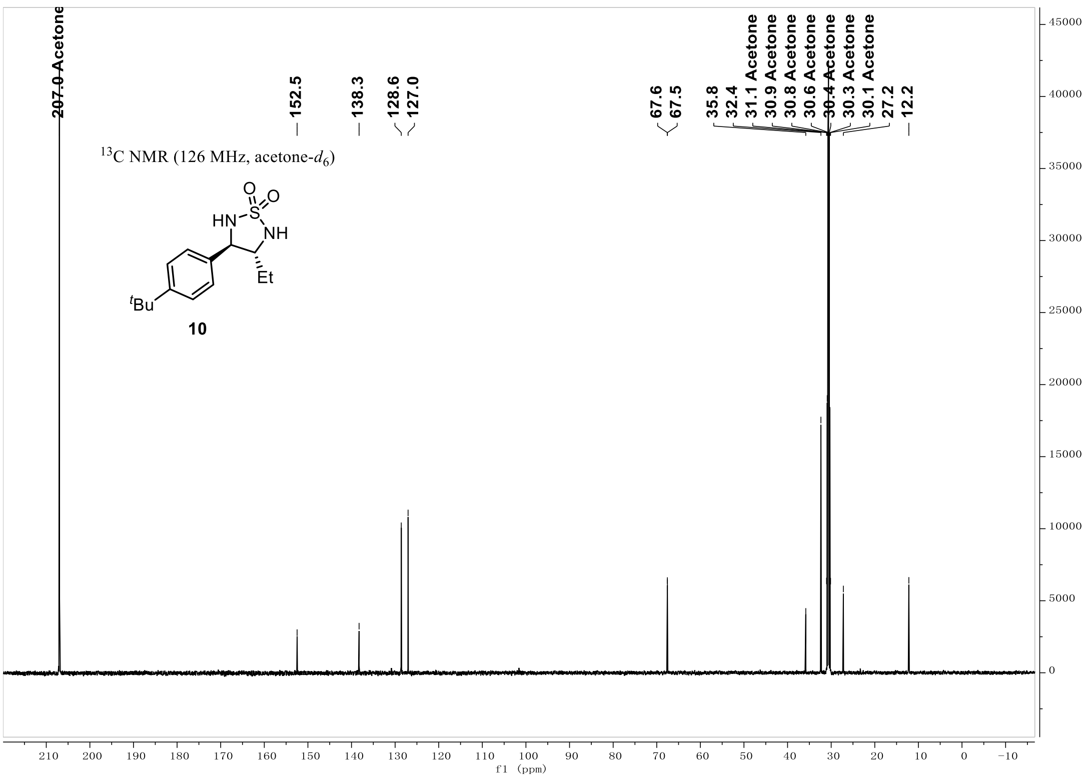

Supplementary Figure 40. ^{1}H NMR and ^{13}C NMR spectra of compound 10.

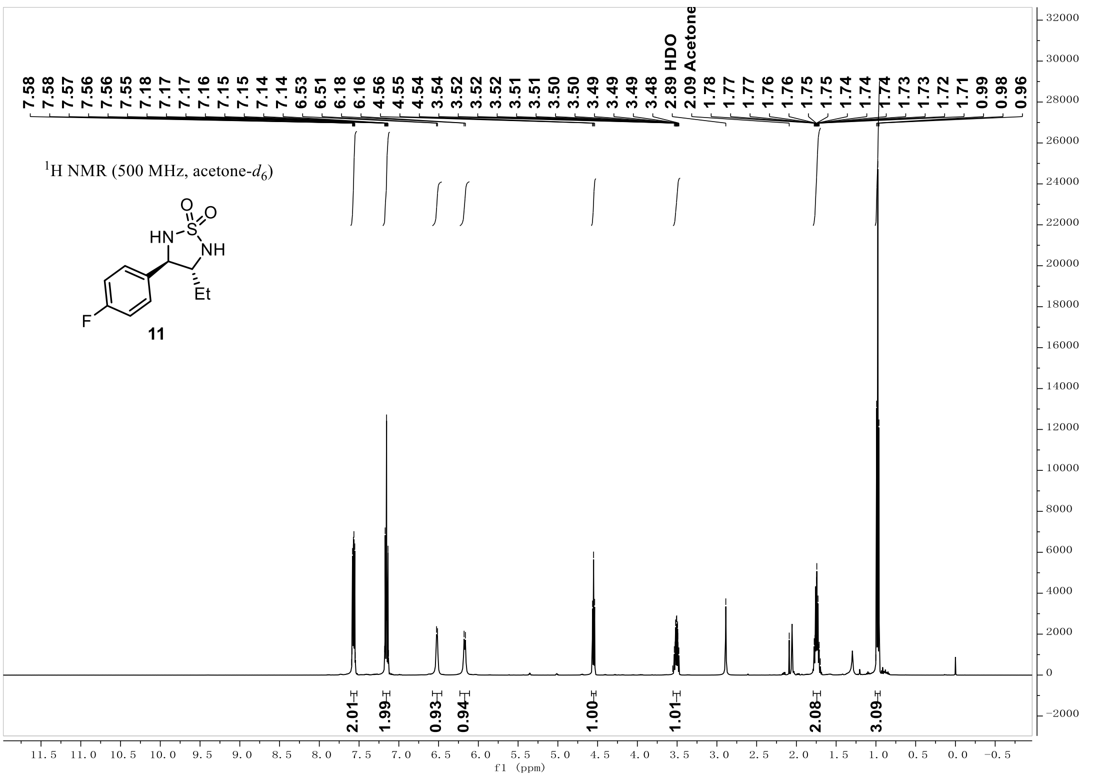

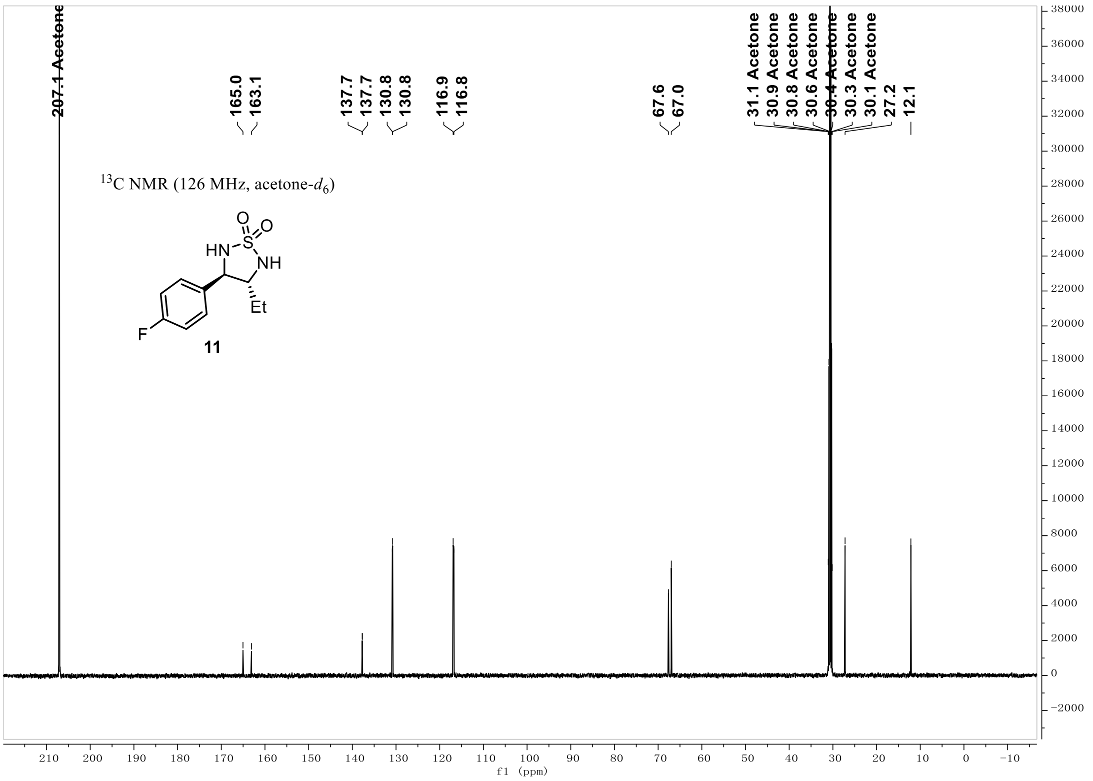

Supplementary Figure 41. ^{1}H NMR and ^{13}C NMR spectra of compound 11.

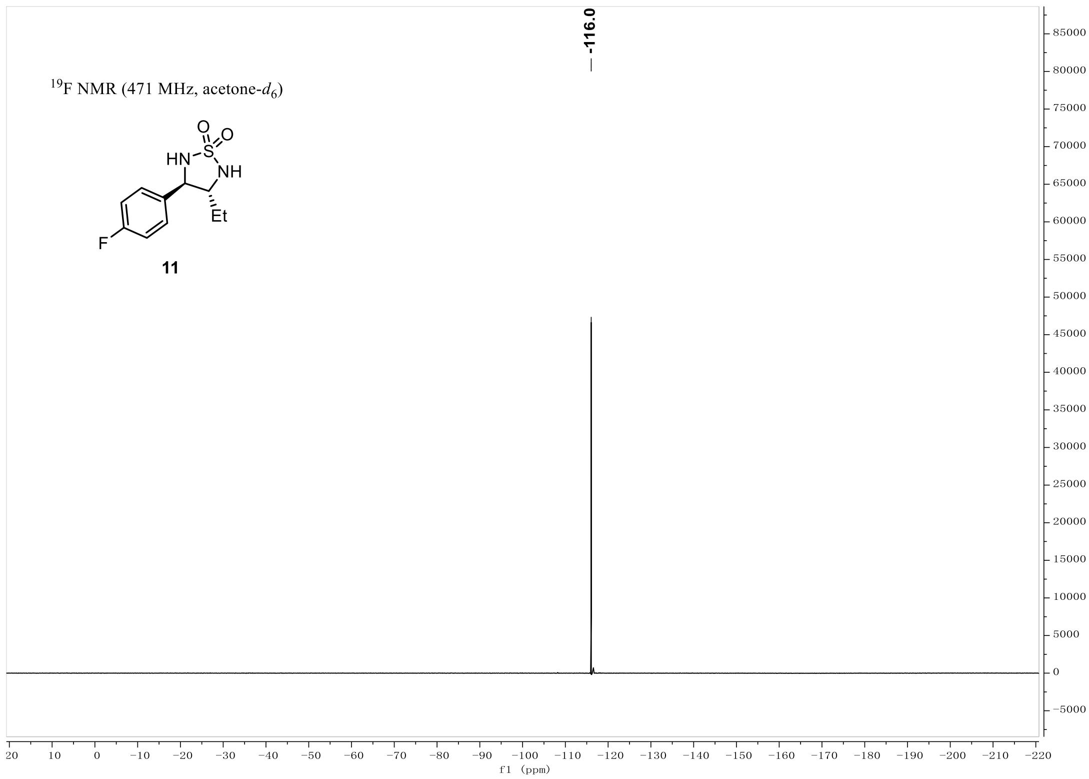

Supplementary Figure 42. ^{19}F NMR spectra of compound 11.

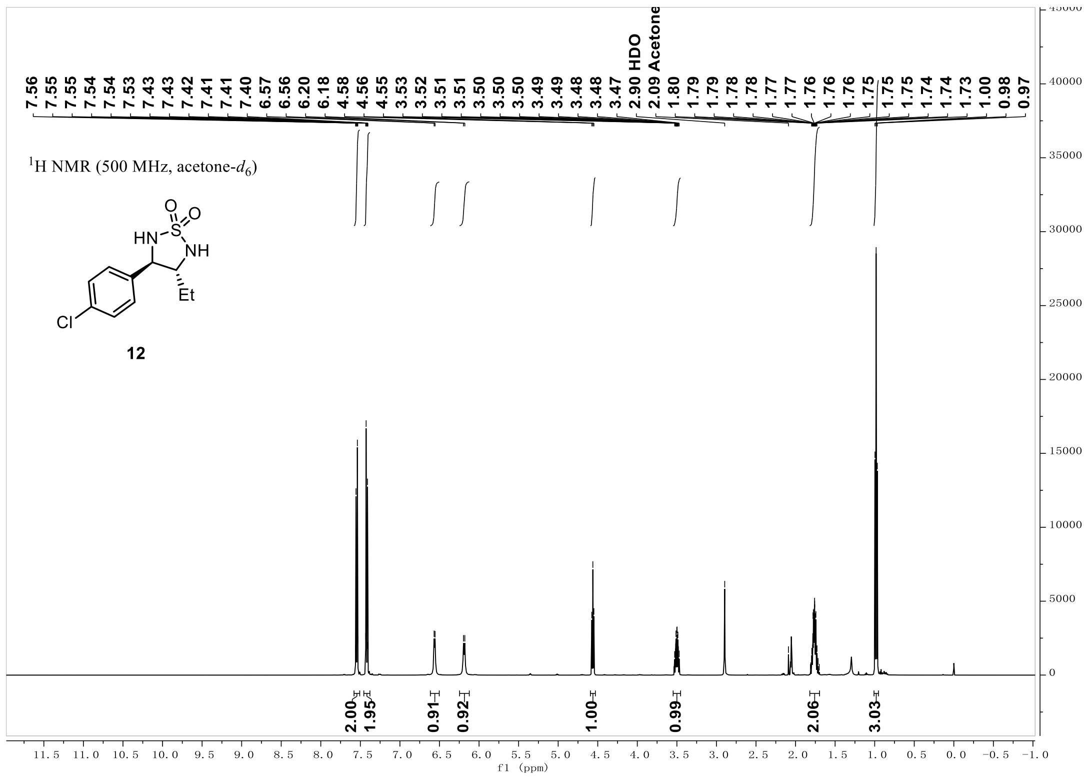

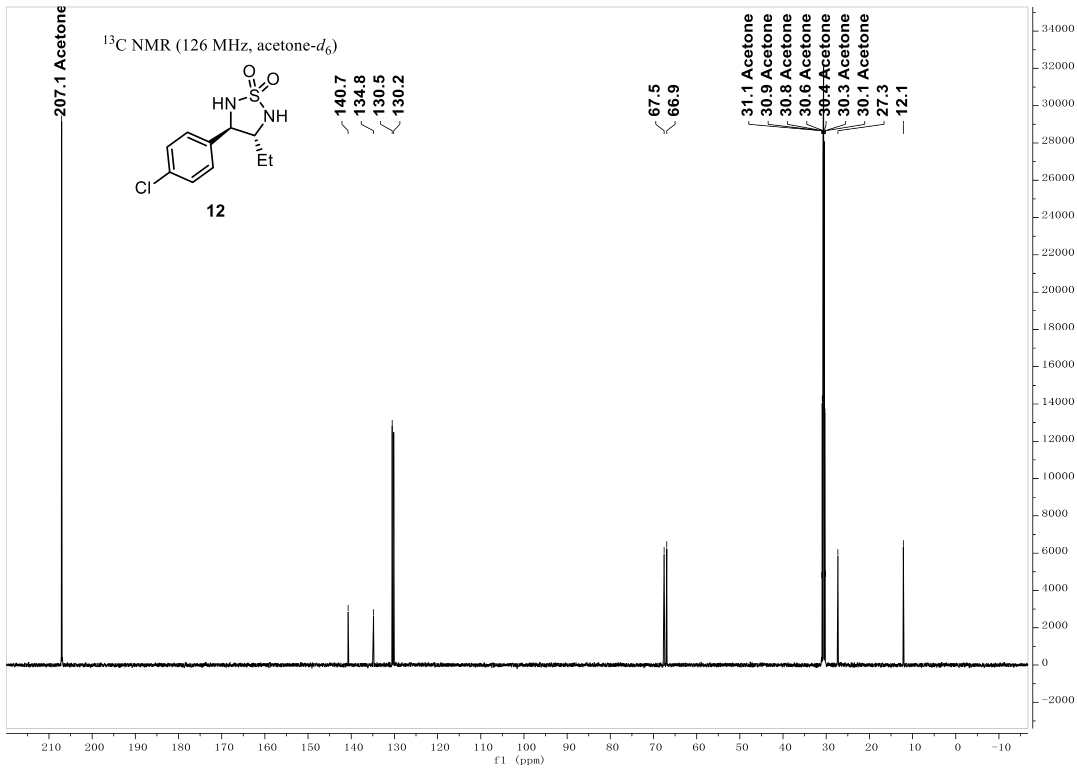

Supplementary Figure 43. ^{1}H NMR and ^{13}C NMR spectra of compound 12.

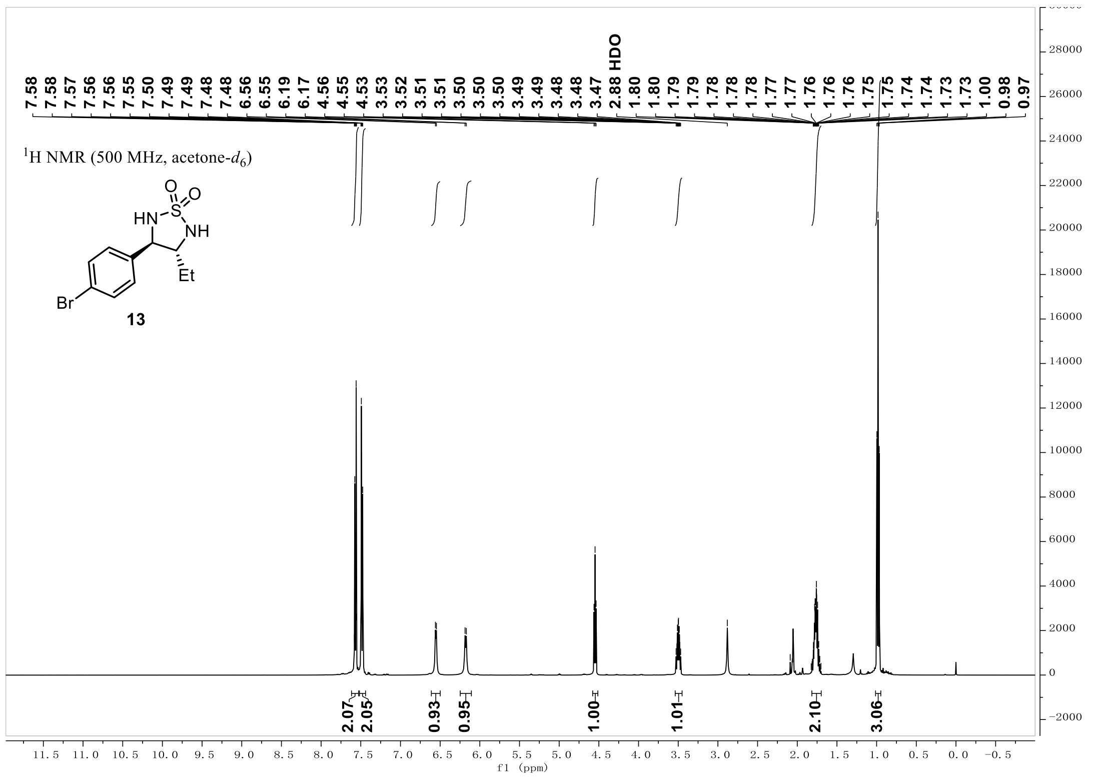

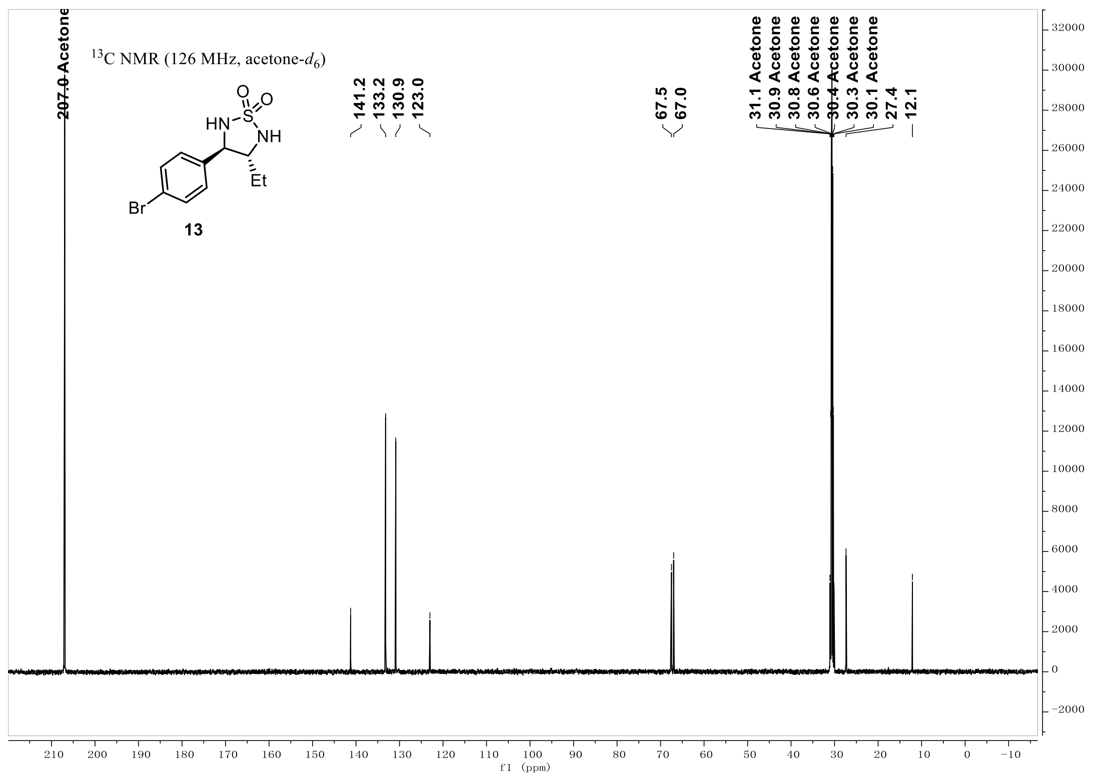

Supplementary Figure 44. ^{1}H NMR and ^{13}C NMR spectra of compound 13.

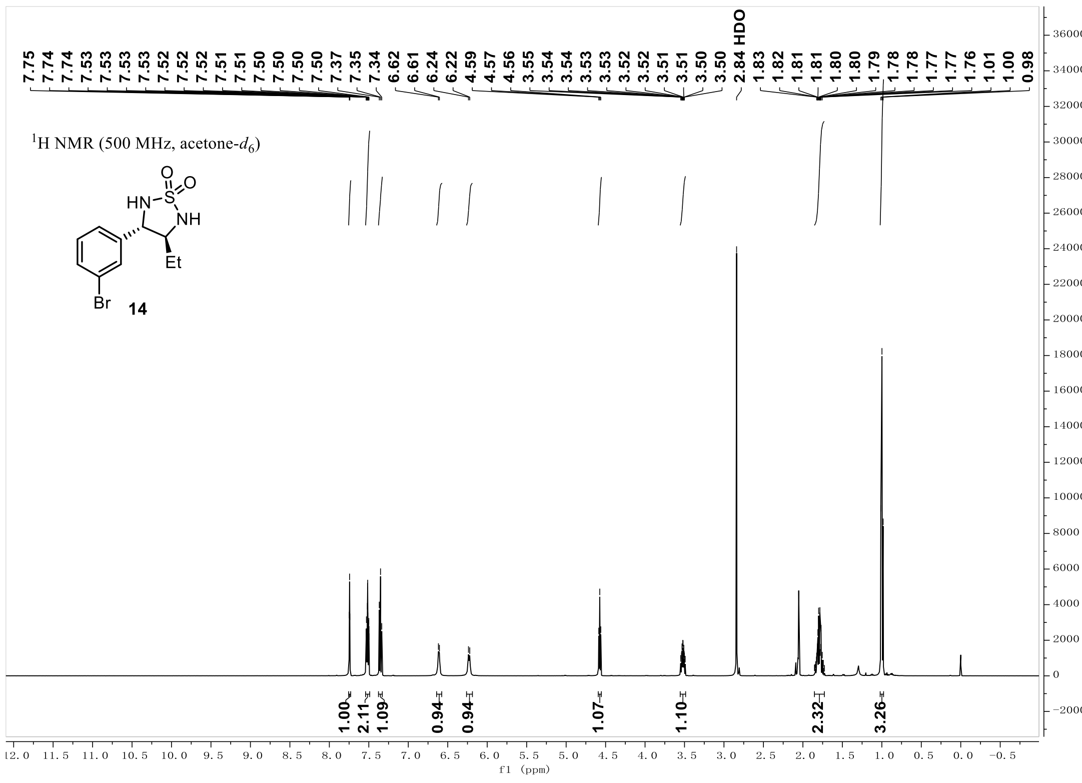

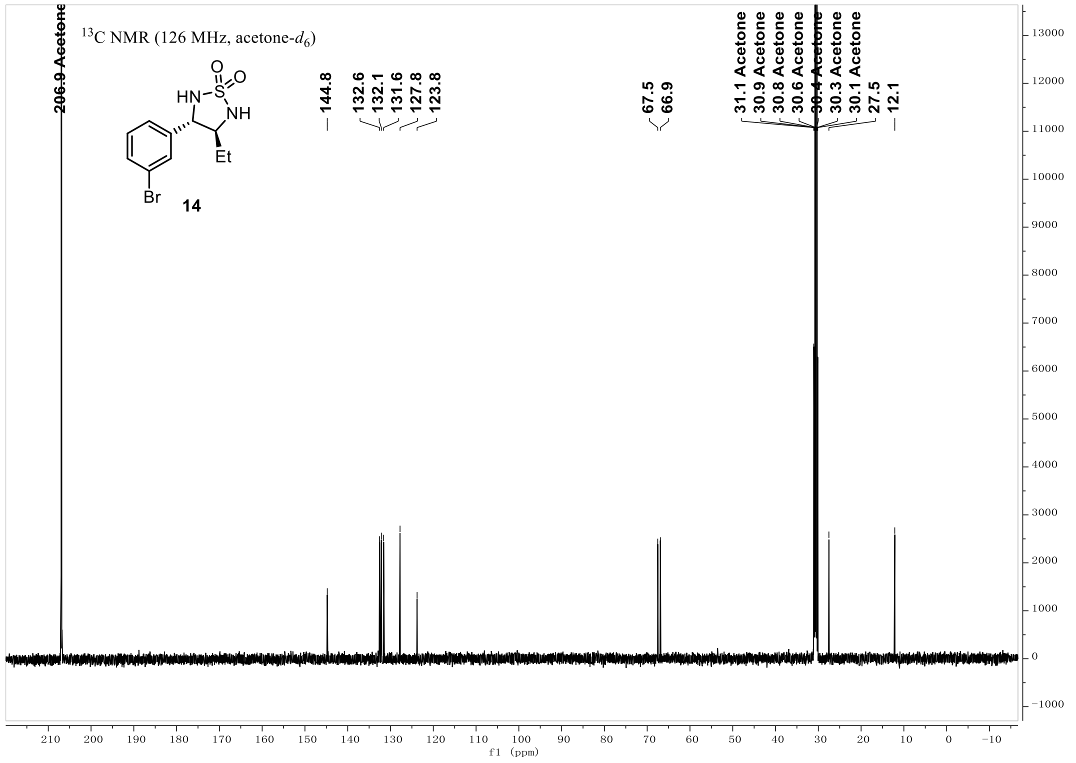

Supplementary Figure 45. ^{1}H NMR and ^{13}C NMR spectra of compound 14.

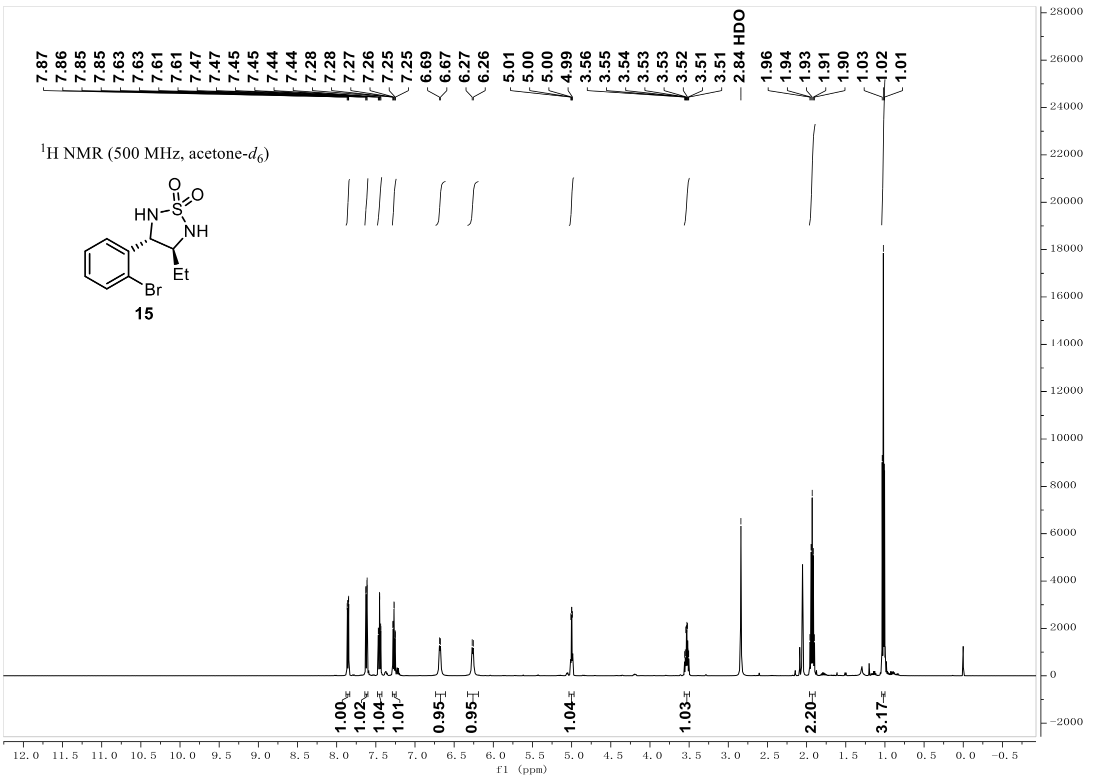

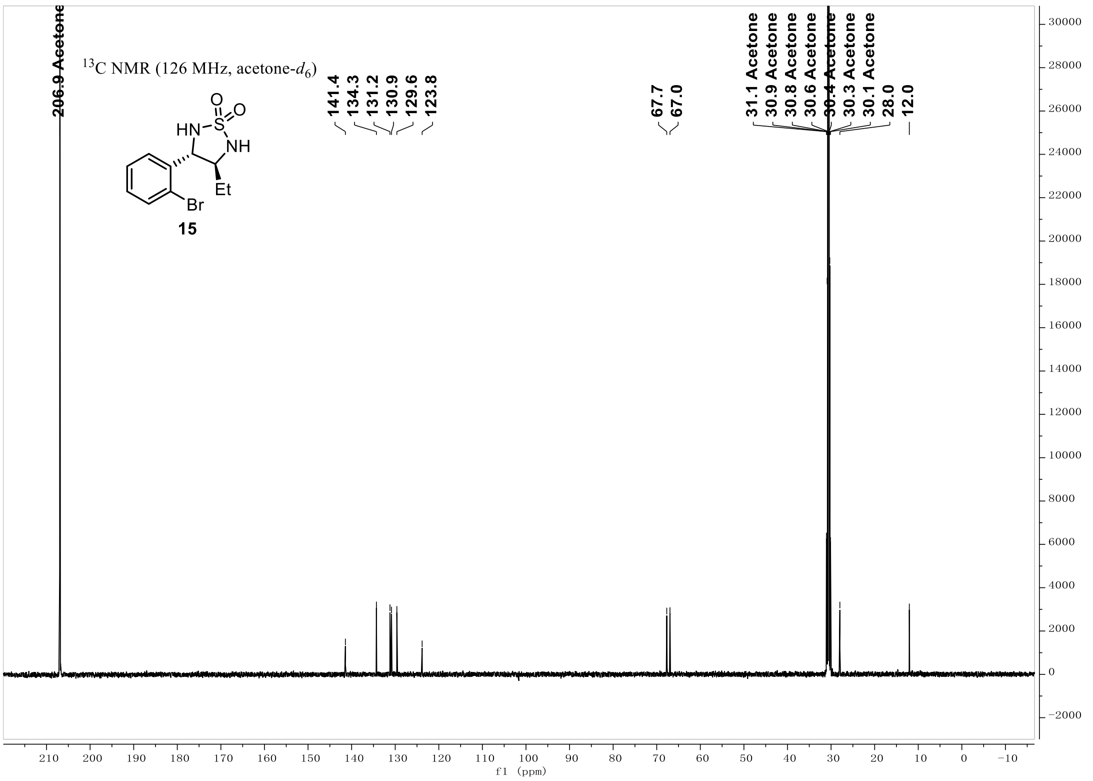

Supplementary Figure 46. ^{1}H NMR and ^{13}C NMR spectra of compound 15.

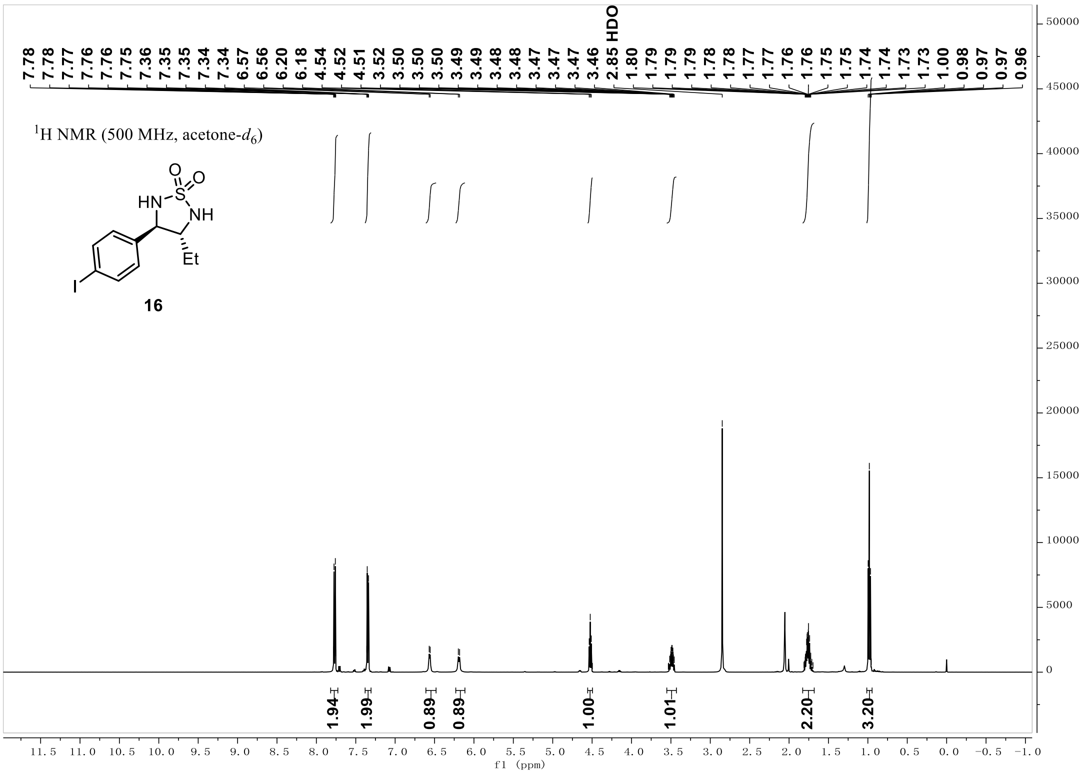

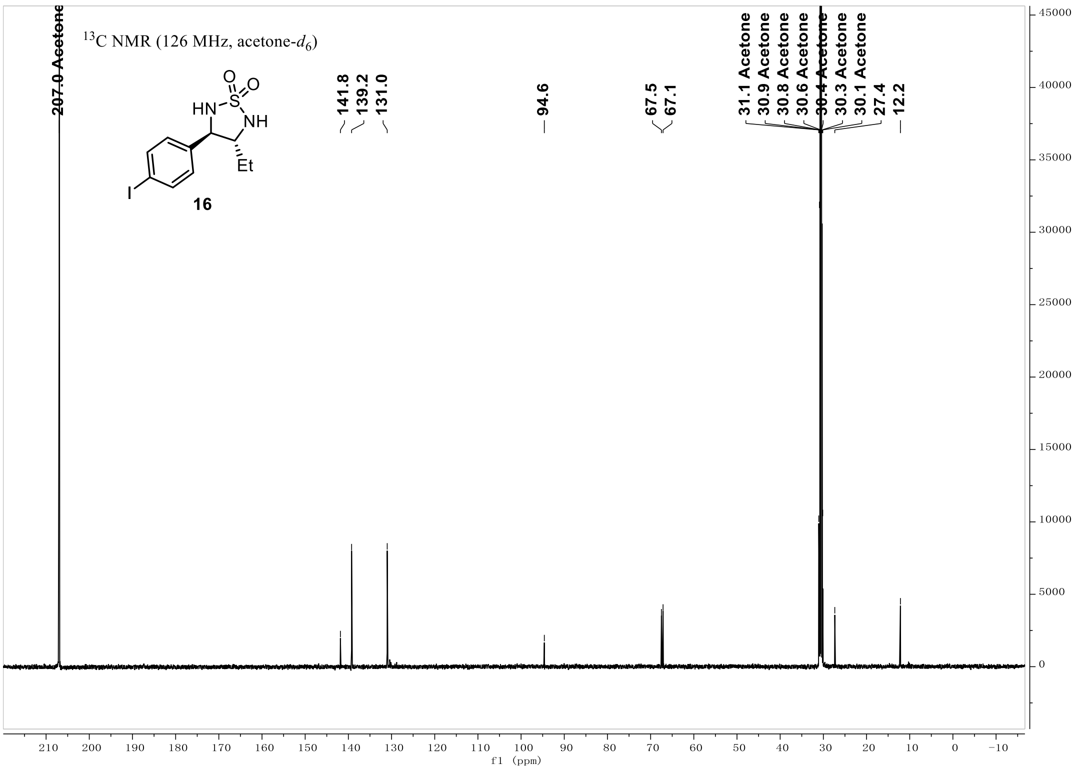

Supplementary Figure 47. ^{1}H NMR and ^{13}C NMR spectra of compound 16.

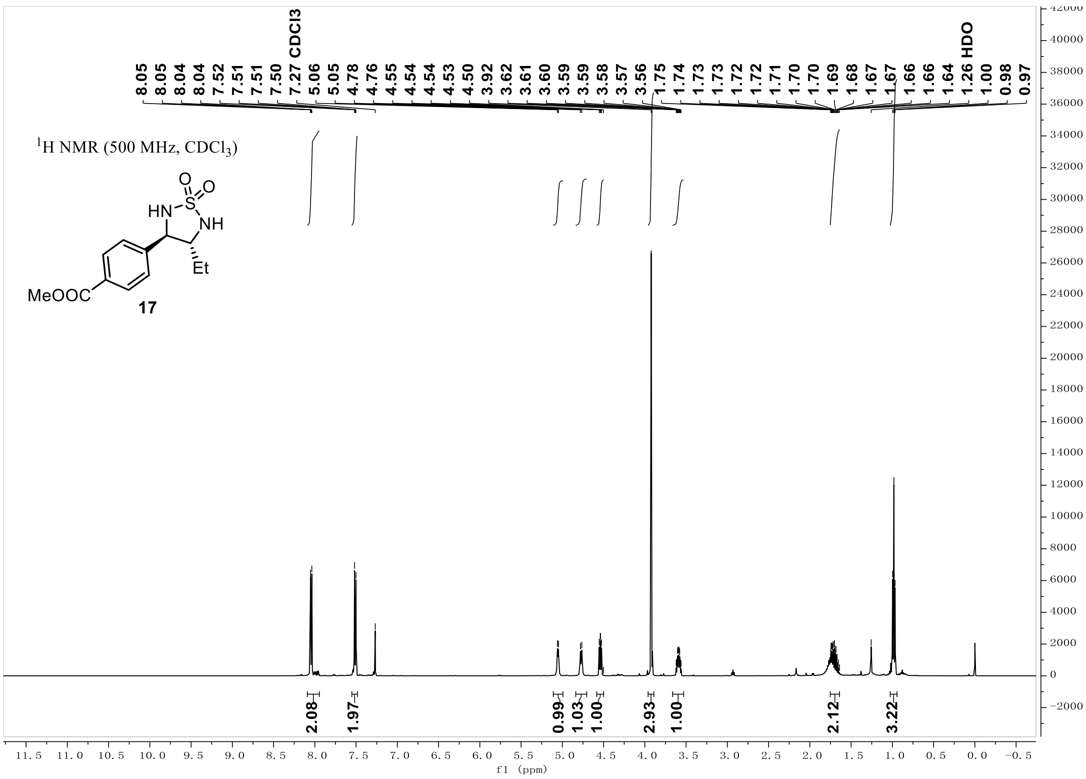

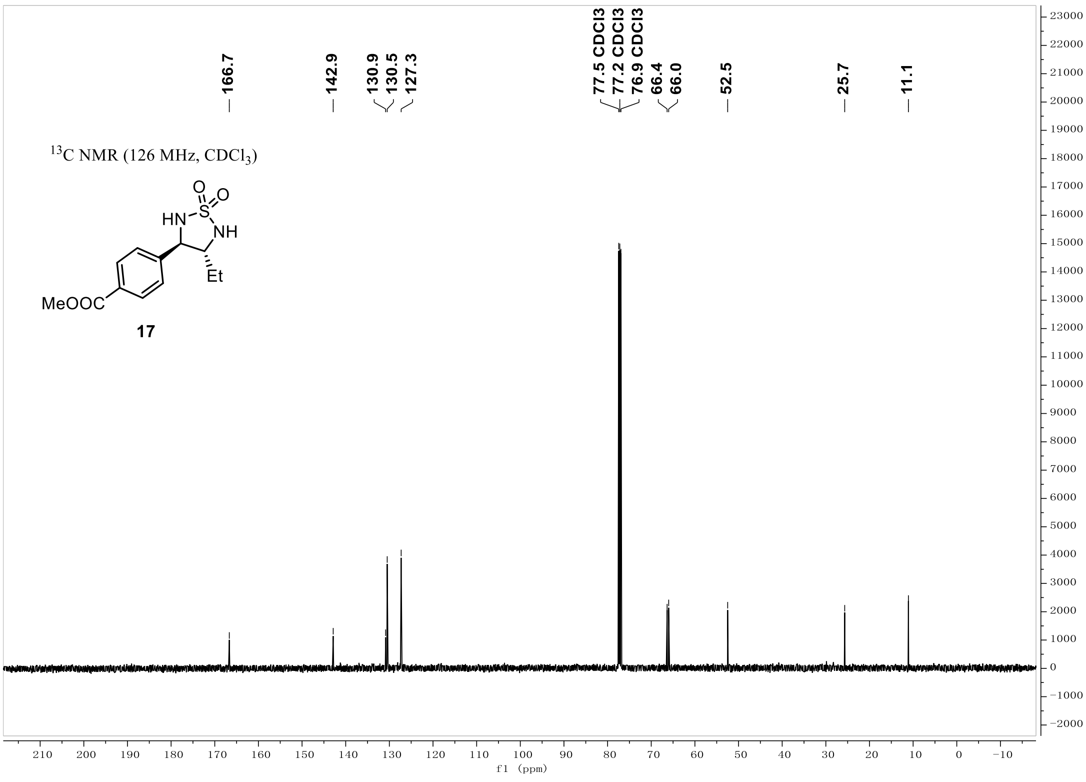

Supplementary Figure 48. ^{1}H NMR and ^{13}C NMR spectra of compound 17.

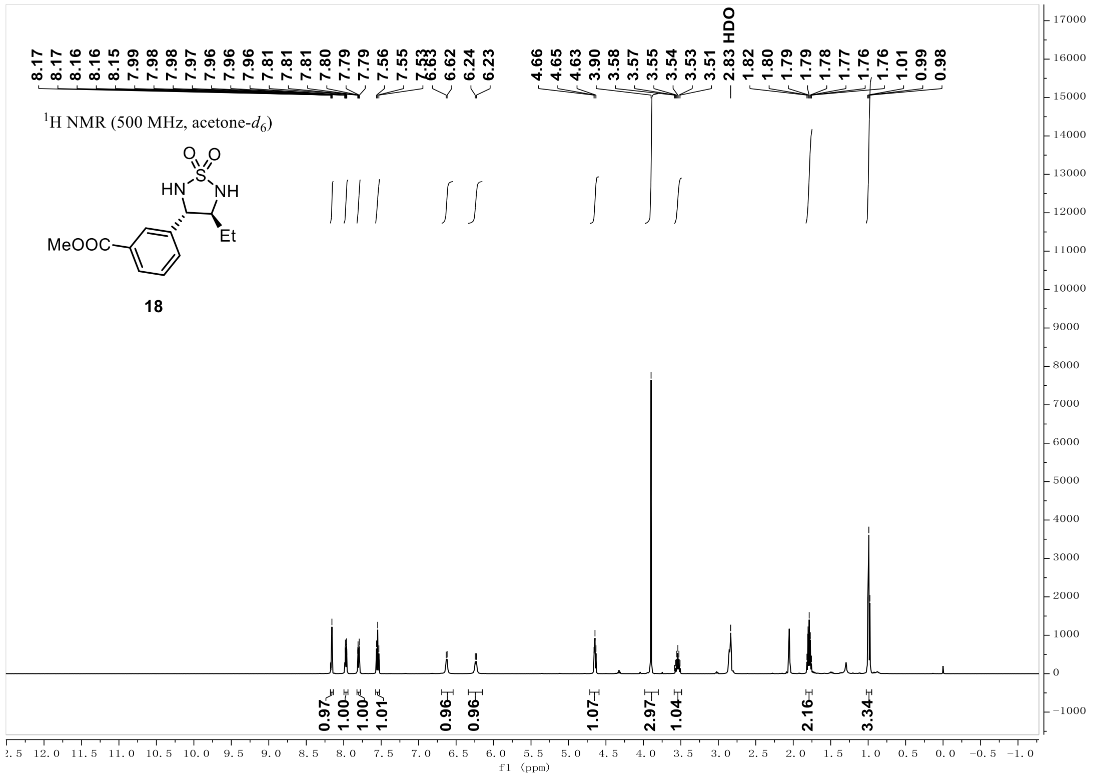

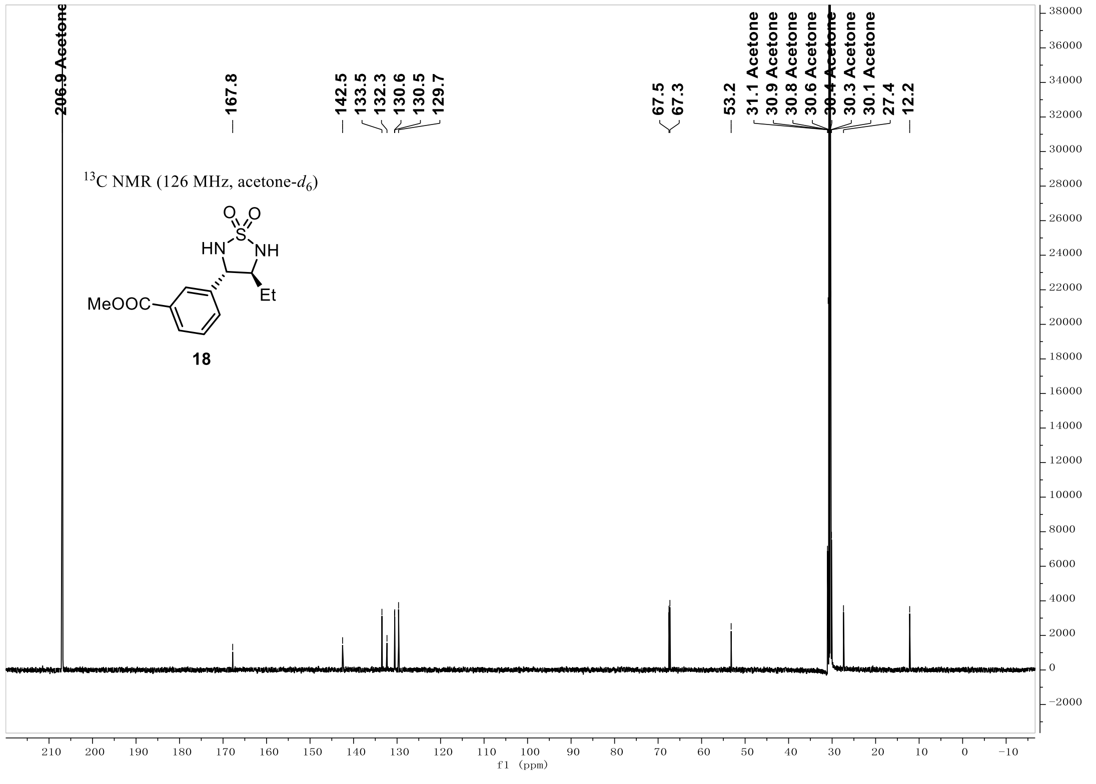

Supplementary Figure 49. ^{1}H NMR and ^{13}C NMR spectra of compound 18.

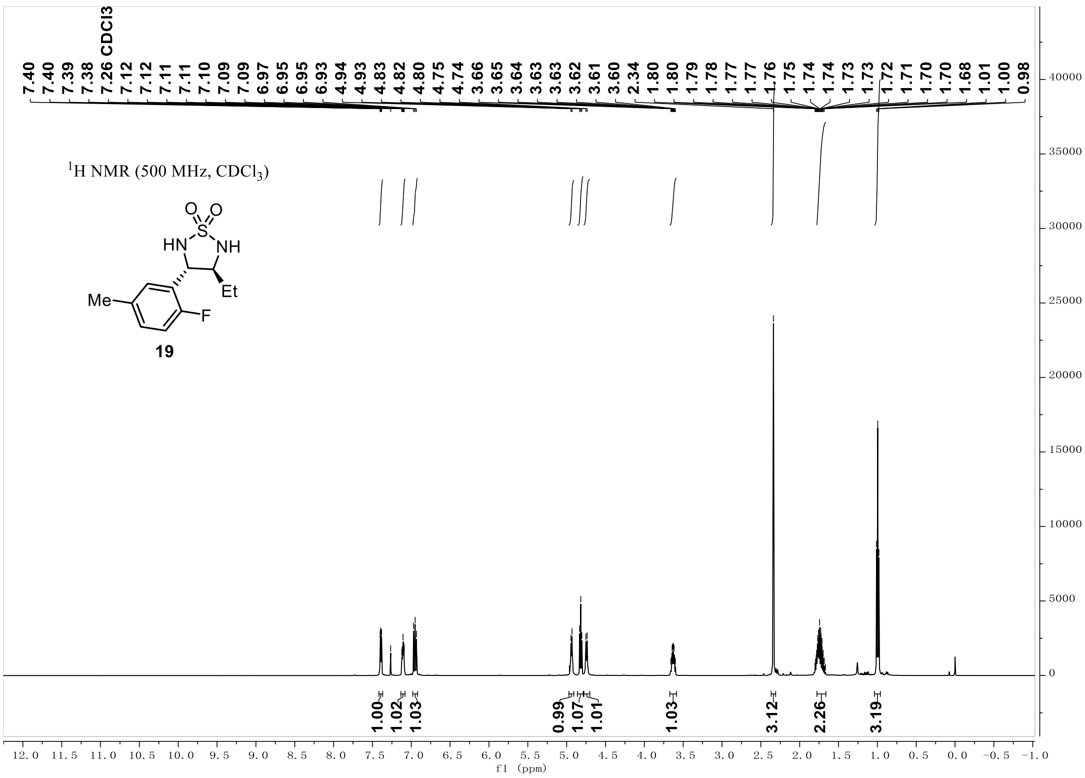

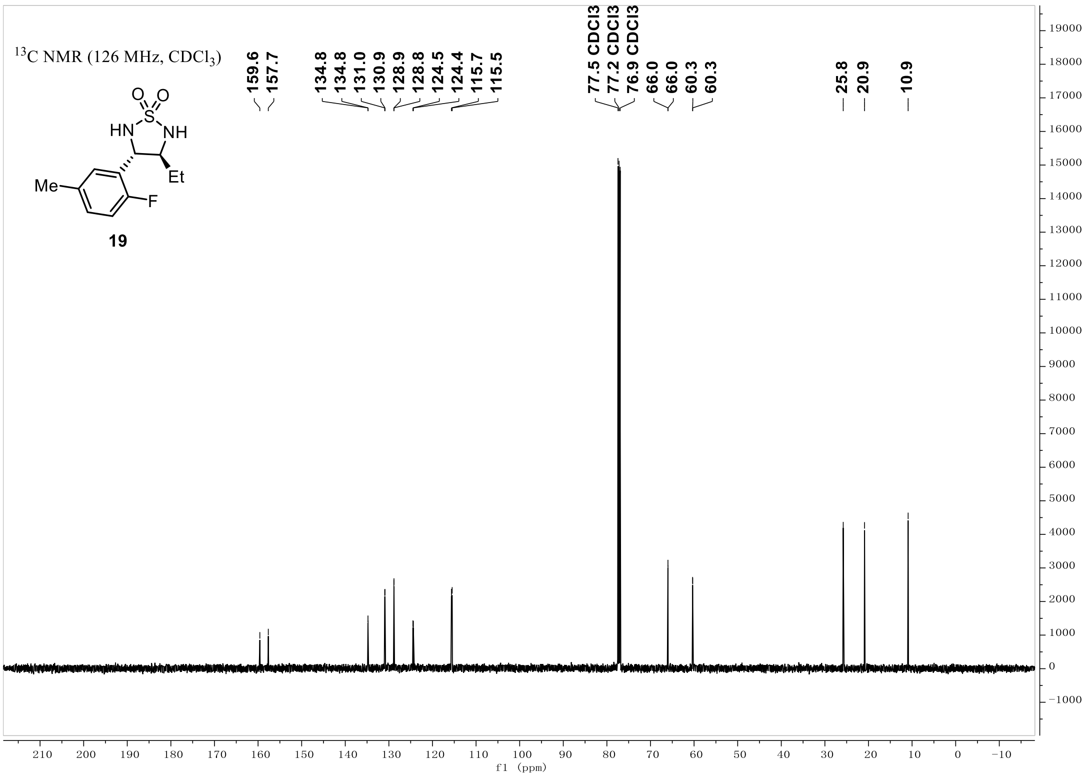

Supplementary Figure 50. ^{1}H NMR and ^{13}C NMR spectra of compound 19.

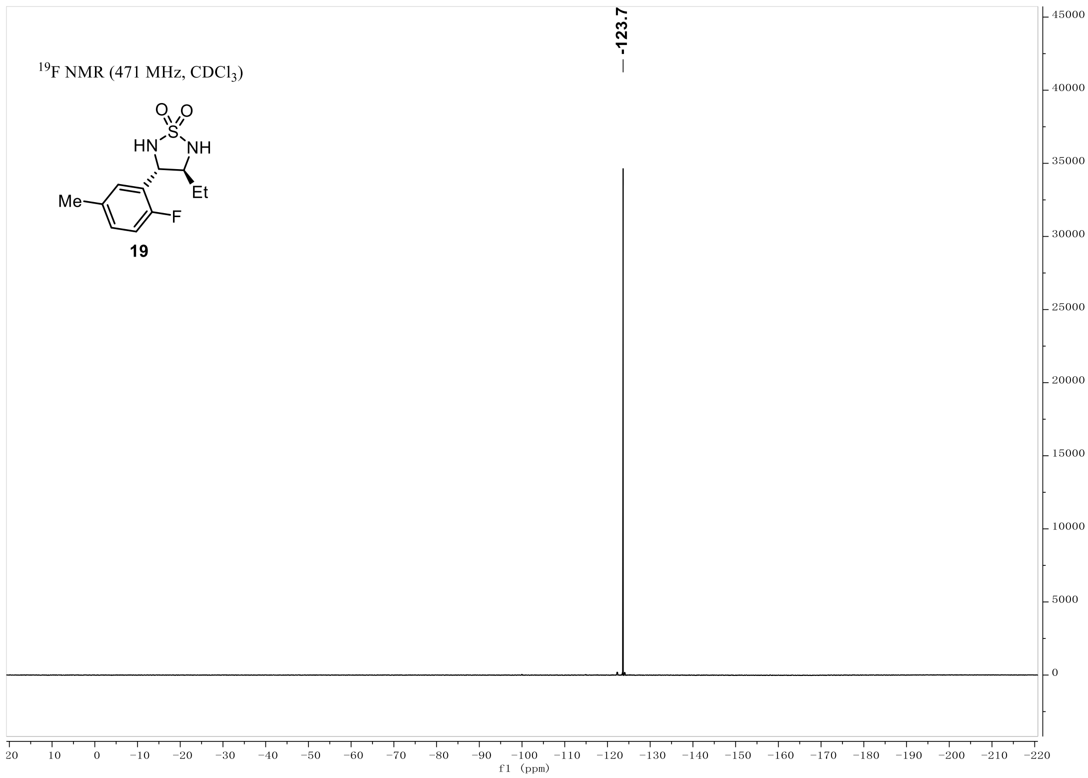

Supplementary Figure 51. ^{19}F NMR spectra of compound 19.

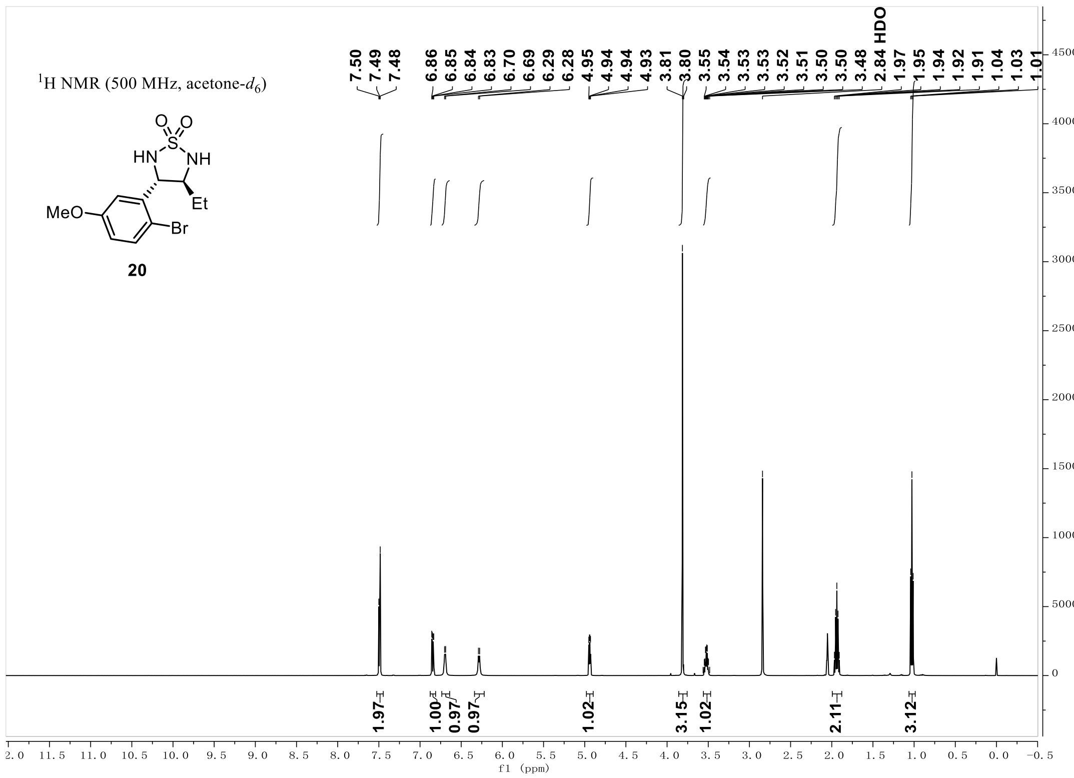

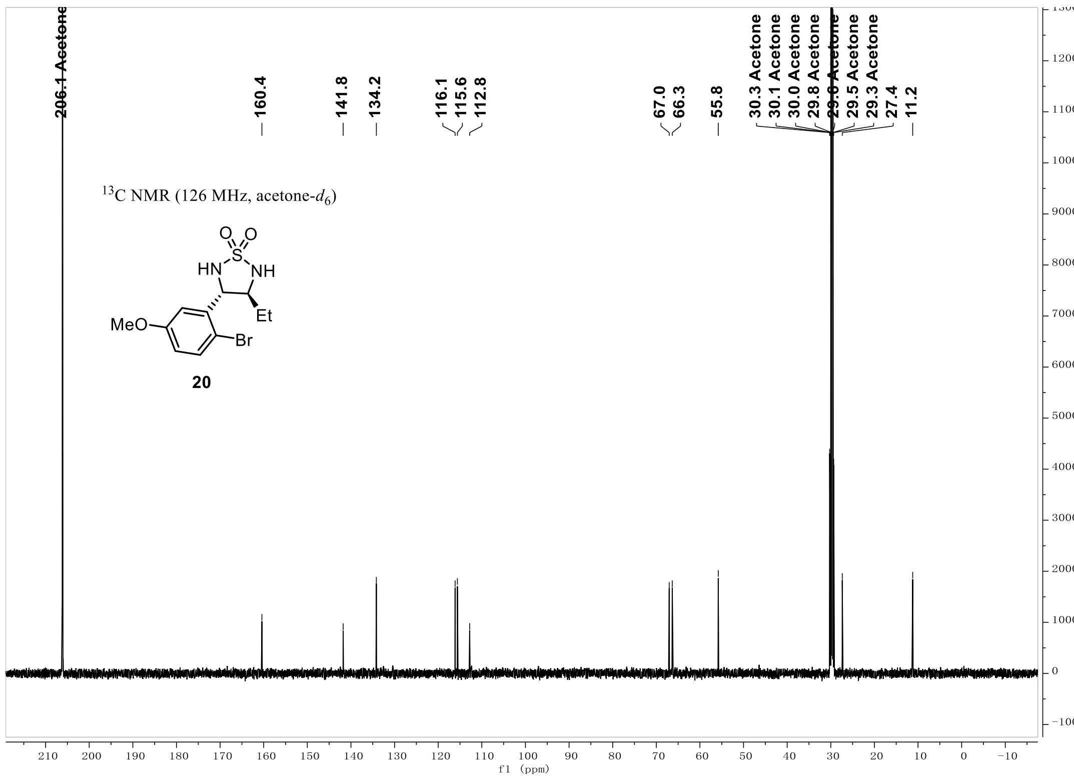

Supplementary Figure 52. ^{1}H NMR and ^{13}C NMR spectra of compound 20.

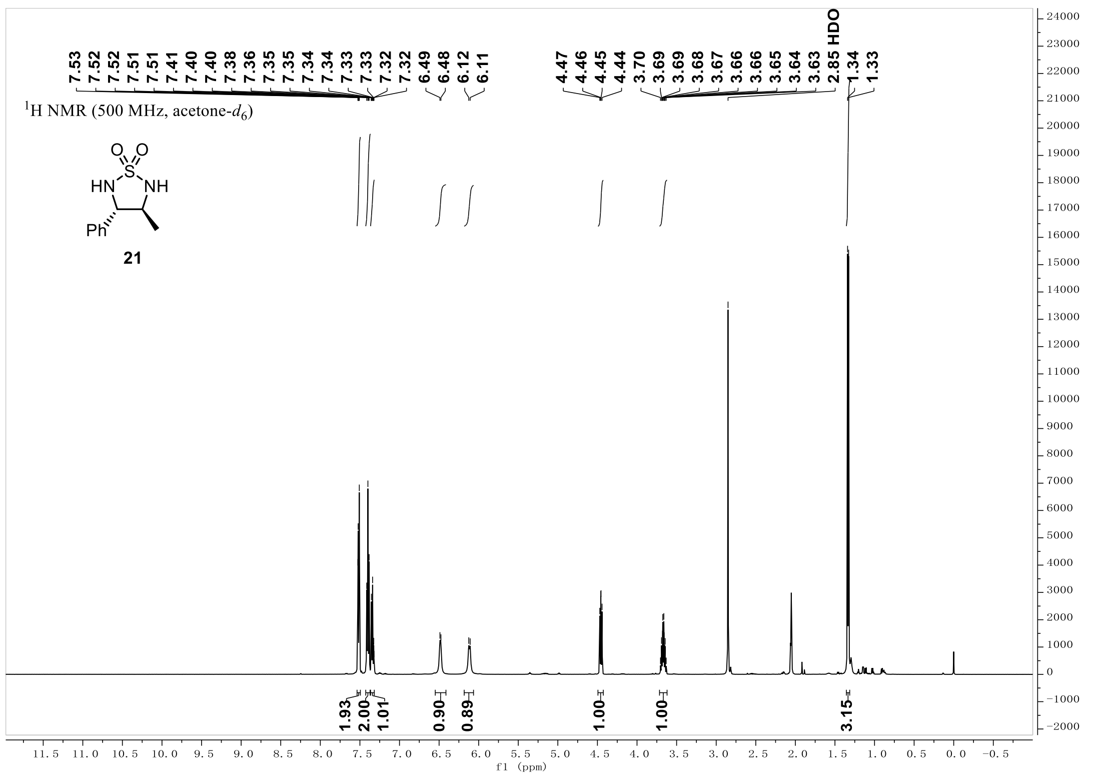

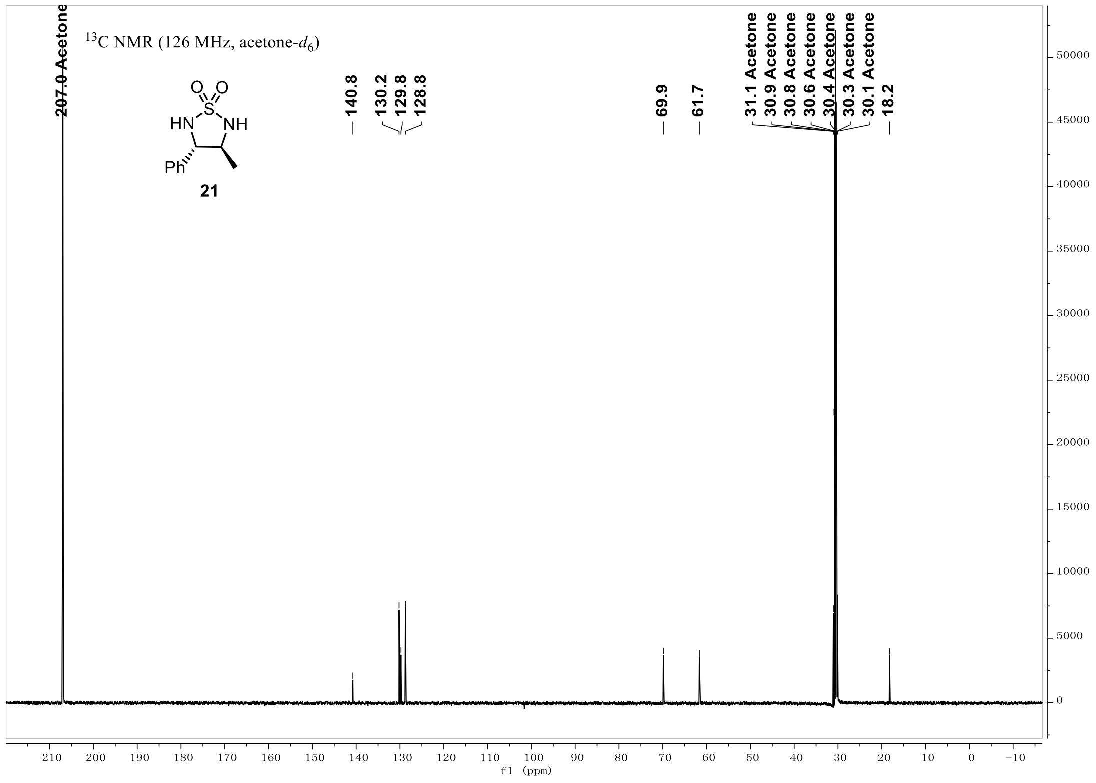

Supplementary Figure 53. ^{1}H NMR and ^{13}C NMR spectra of compound 21.

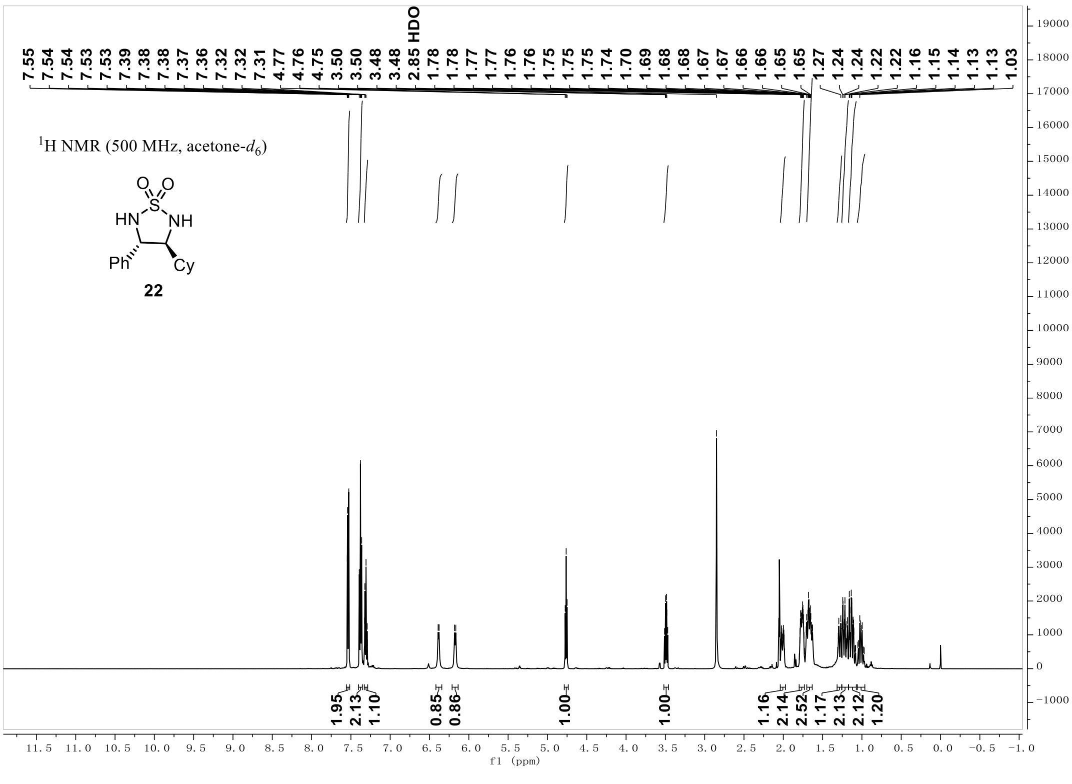

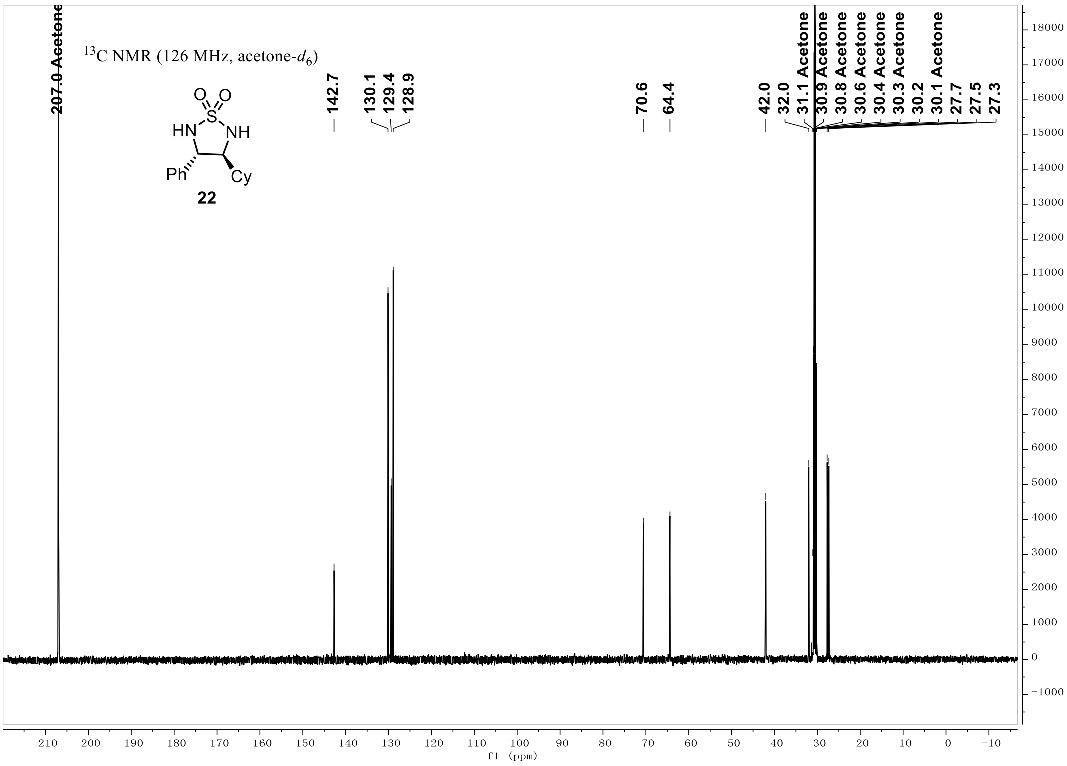

Supplementary Figure 54. ^{1}H NMR and ^{13}C NMR spectra of compound 22.

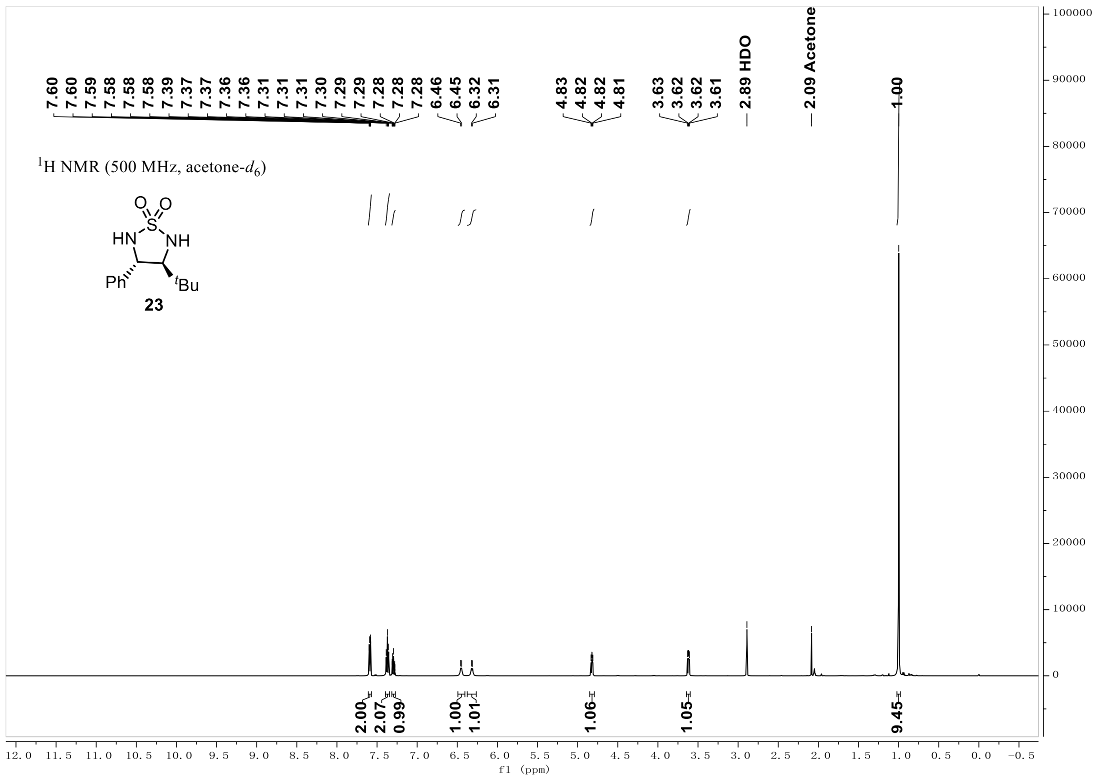

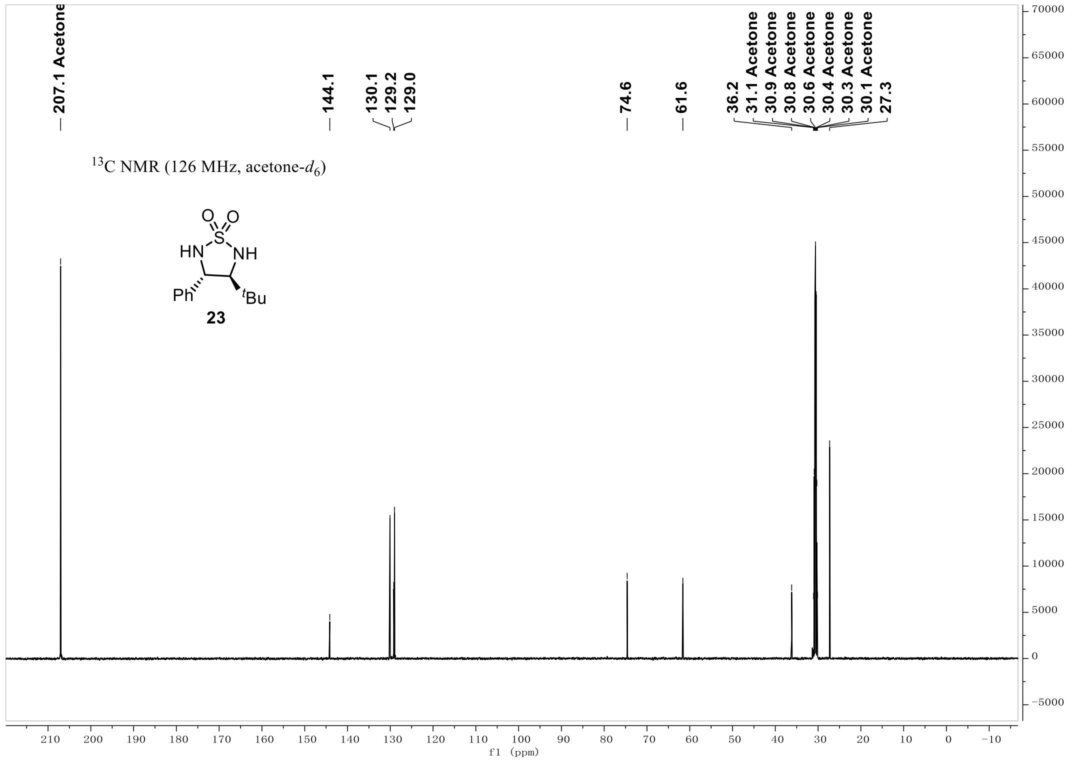

Supplementary Figure 55. ^{1}H NMR and ^{13}C NMR spectra of compound 23.

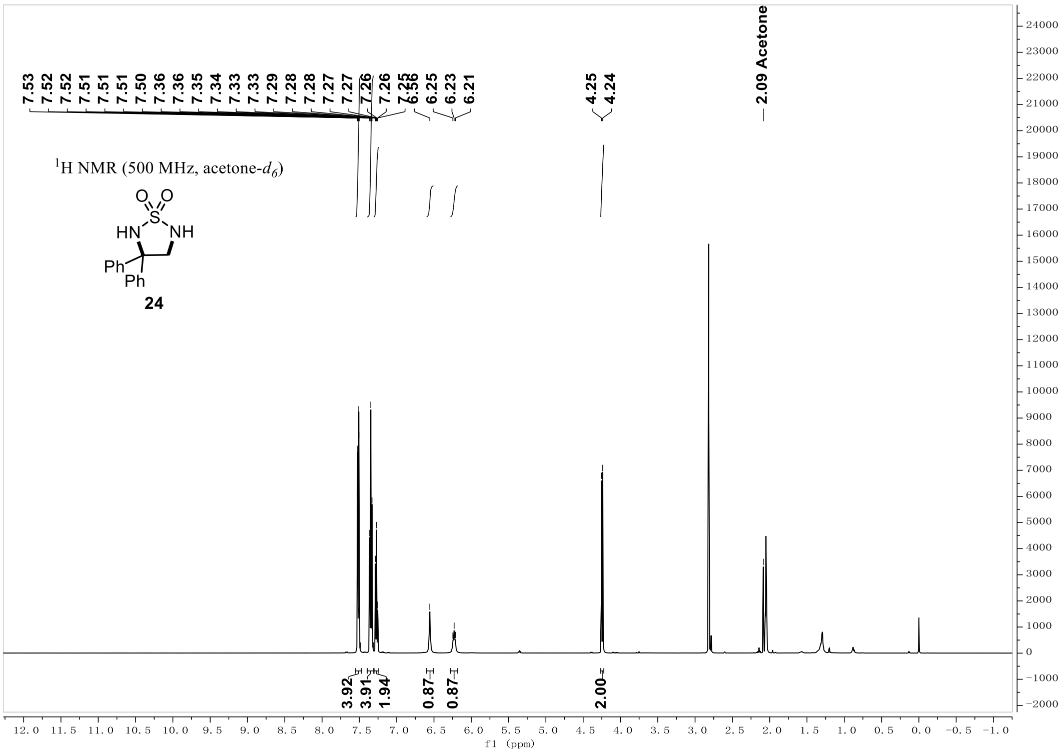

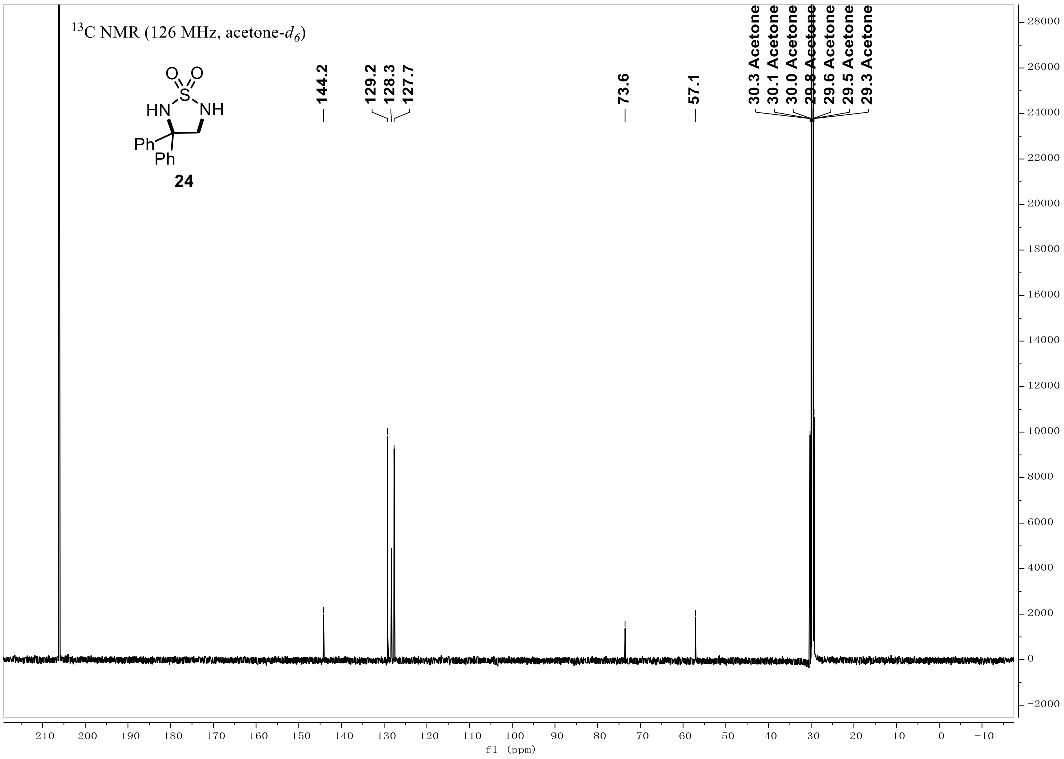

Supplementary Figure 56. ^{1}H NMR and ^{13}C NMR spectra of compound 24.

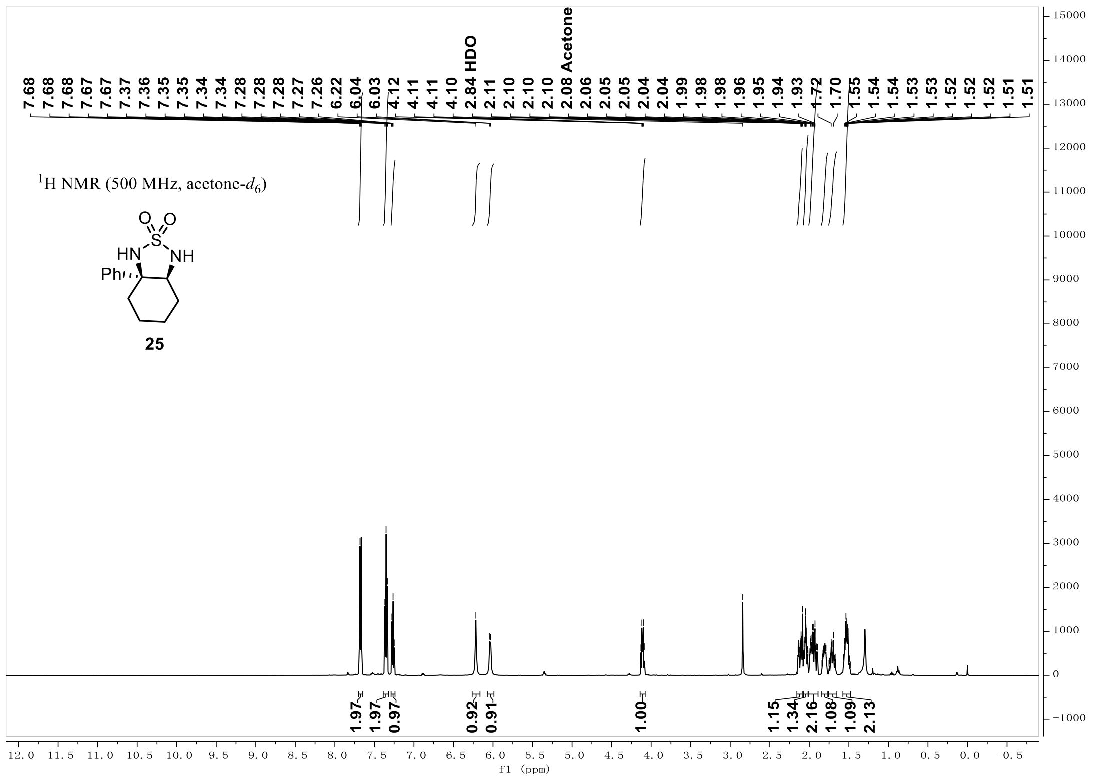

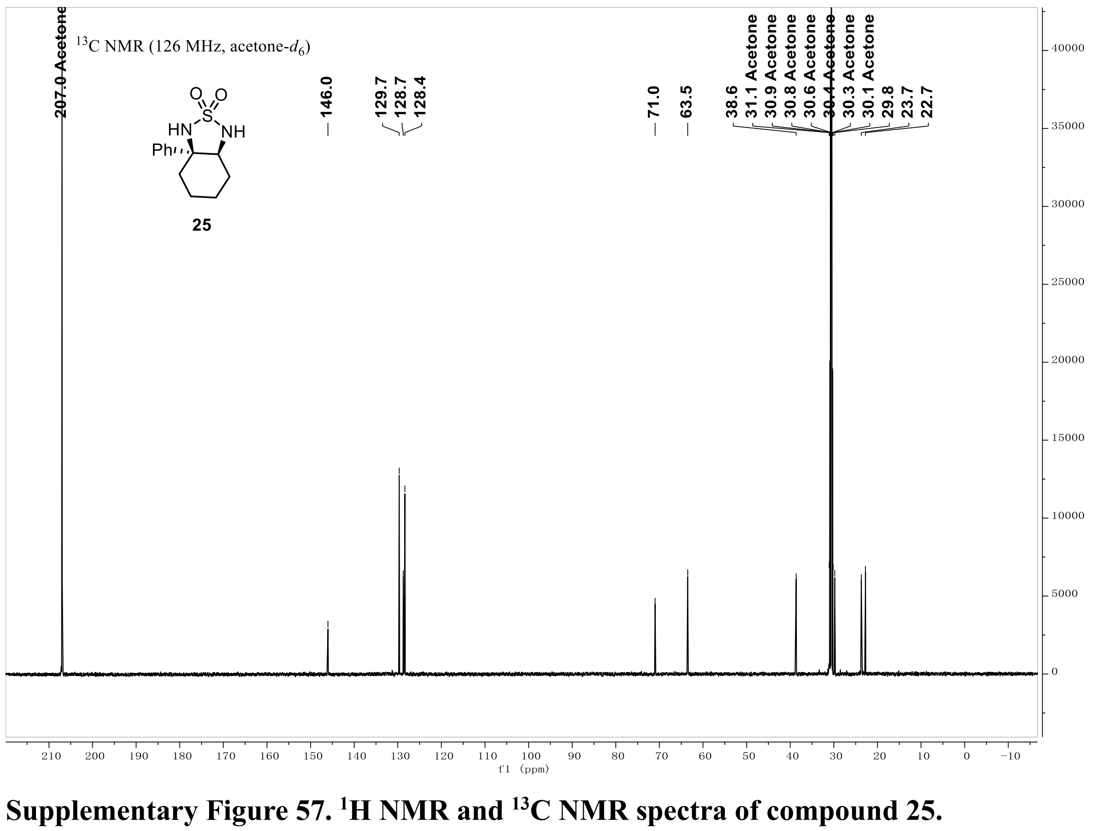

Supplementary Figure 57. ^{1}H NMR and ^{13}C NMR spectra of compound 25.

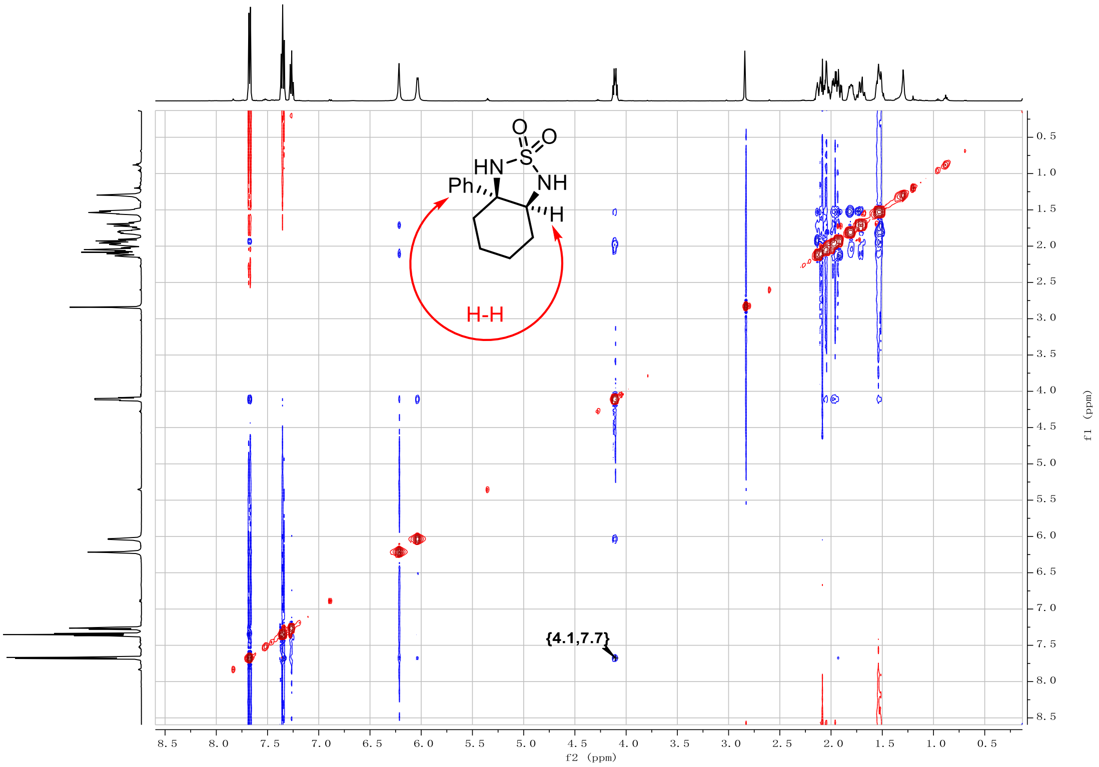

Supplementary Figure 58. 2D NOESY spectra of compound 25.

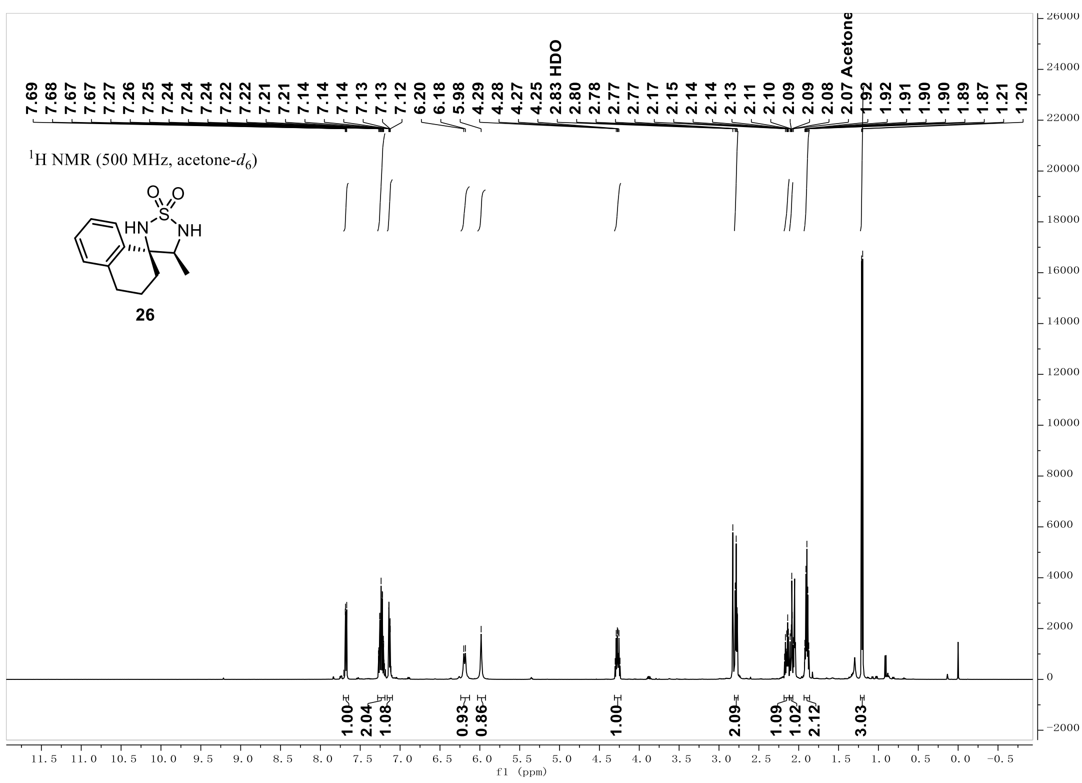

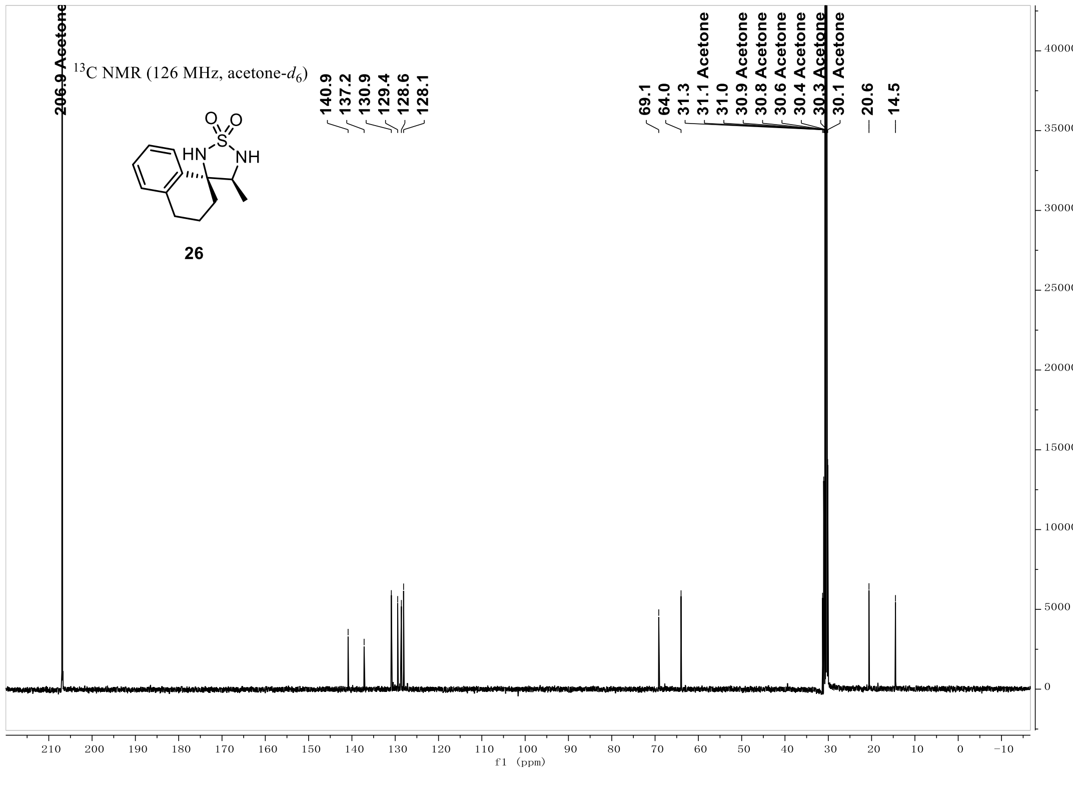

Supplementary Figure 59. ^{1}H NMR and ^{13}C NMR spectra of compound 26.

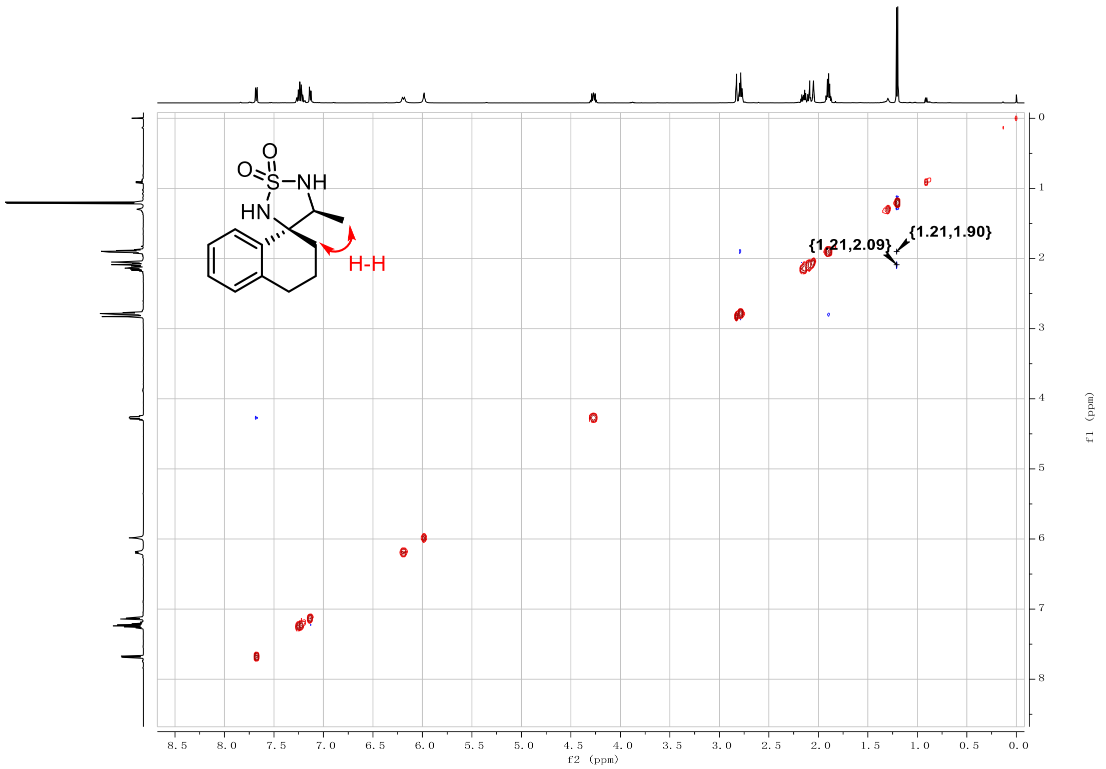

Supplementary Figure 60. 2D NOESY spectra of compound 26.

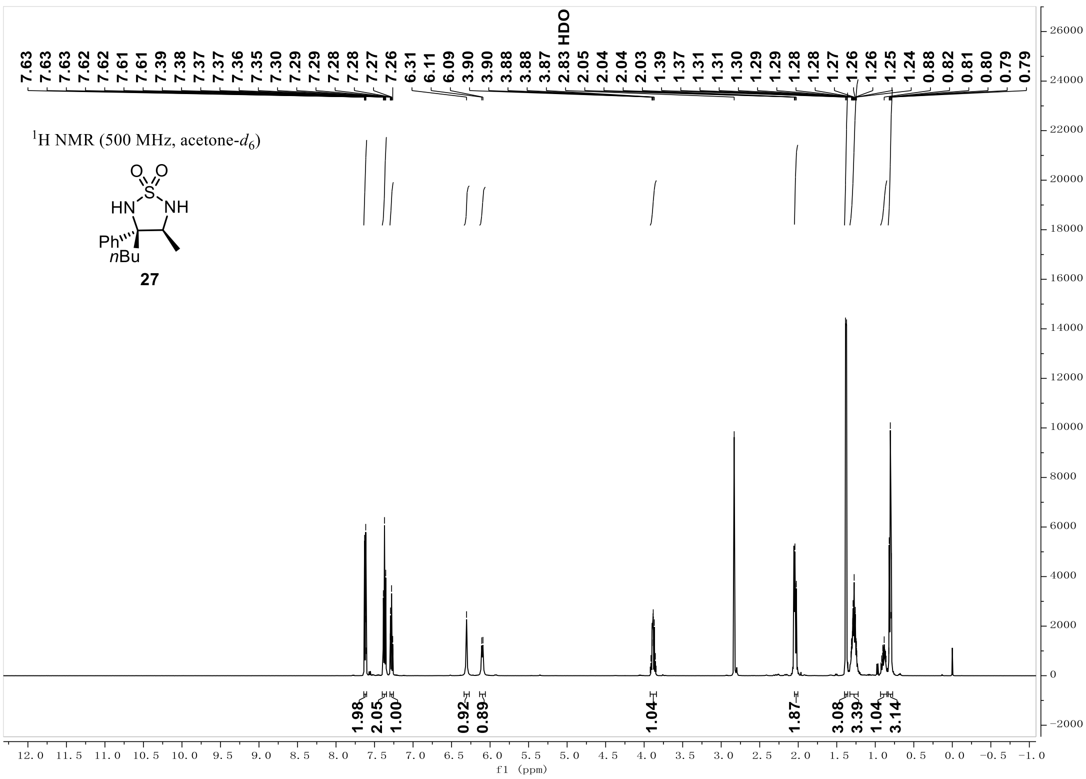

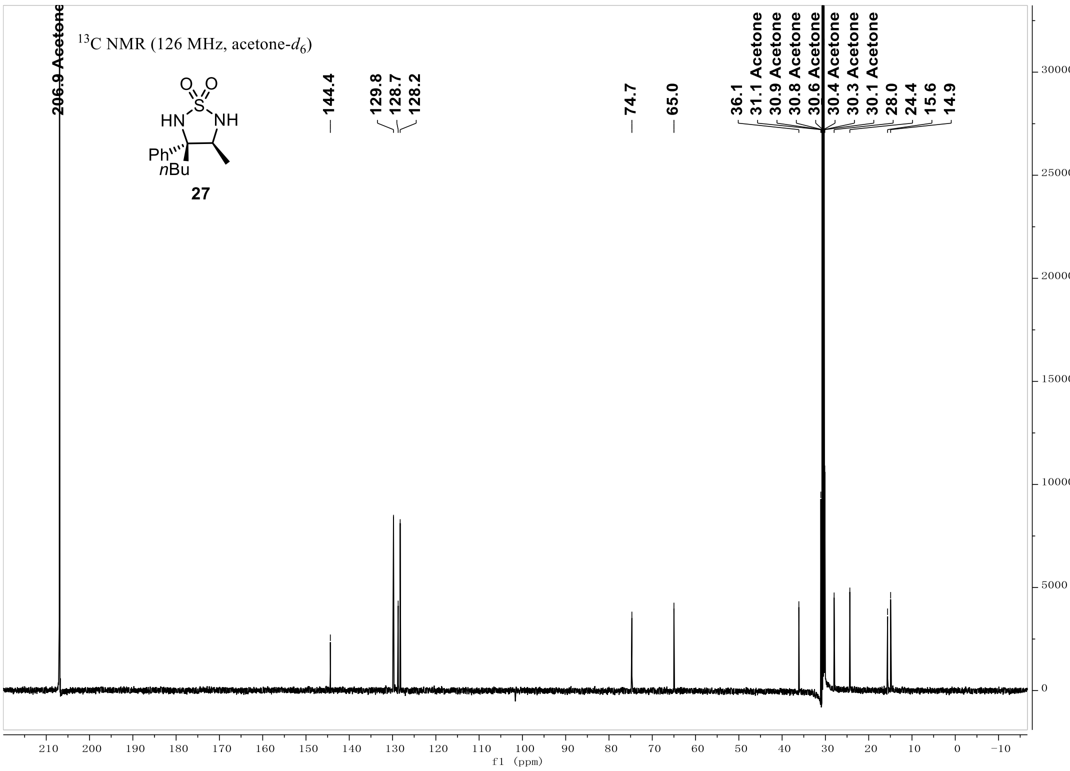

Supplementary Figure 61. ^{1}H NMR and ^{13}C NMR spectra of compound 27.

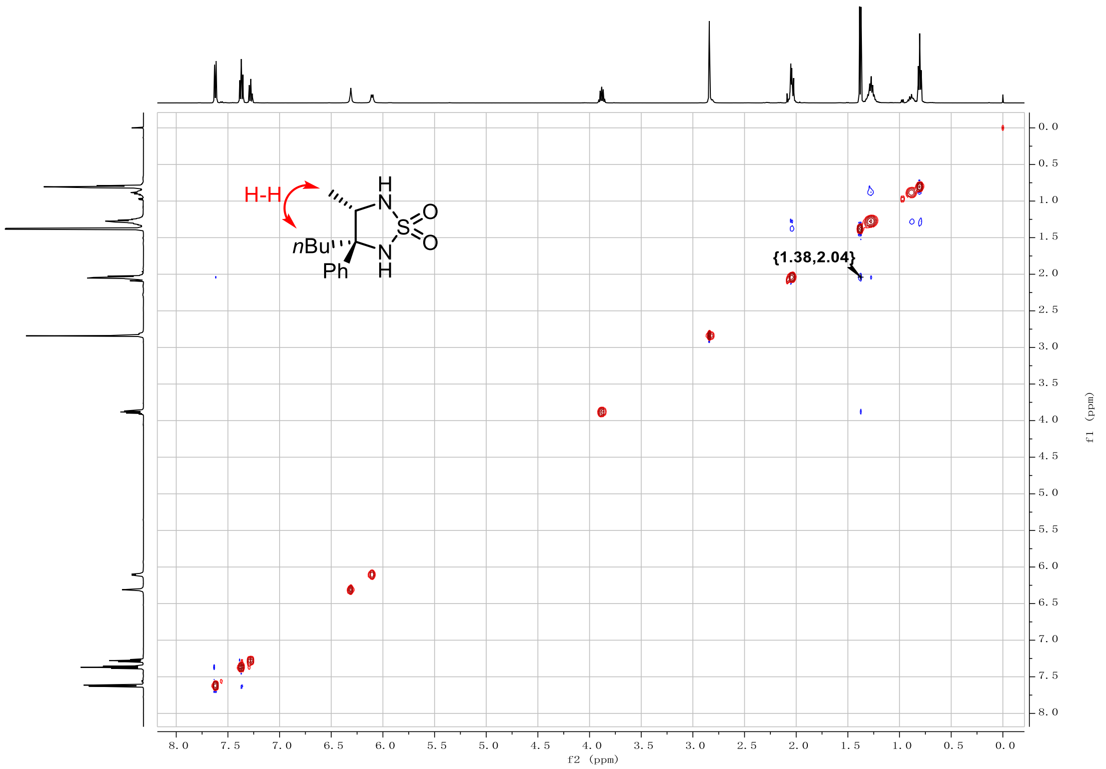

Supplementary Figure 62. 2D NOESY spectra of compound 27.

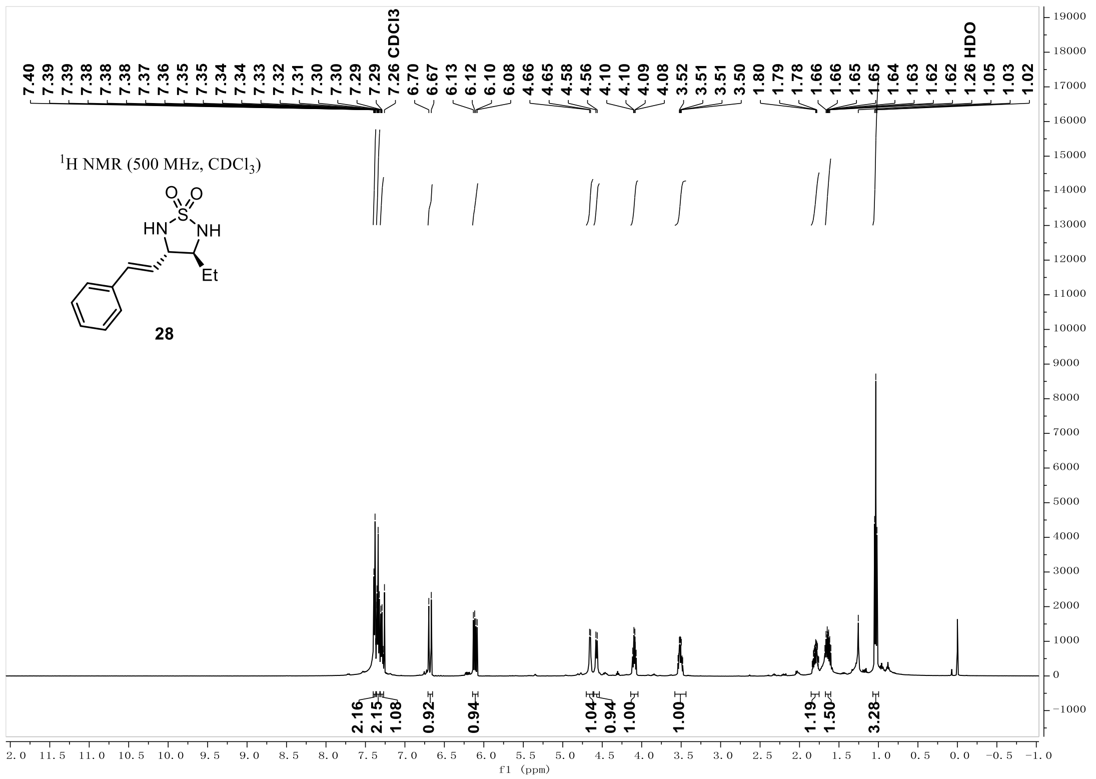

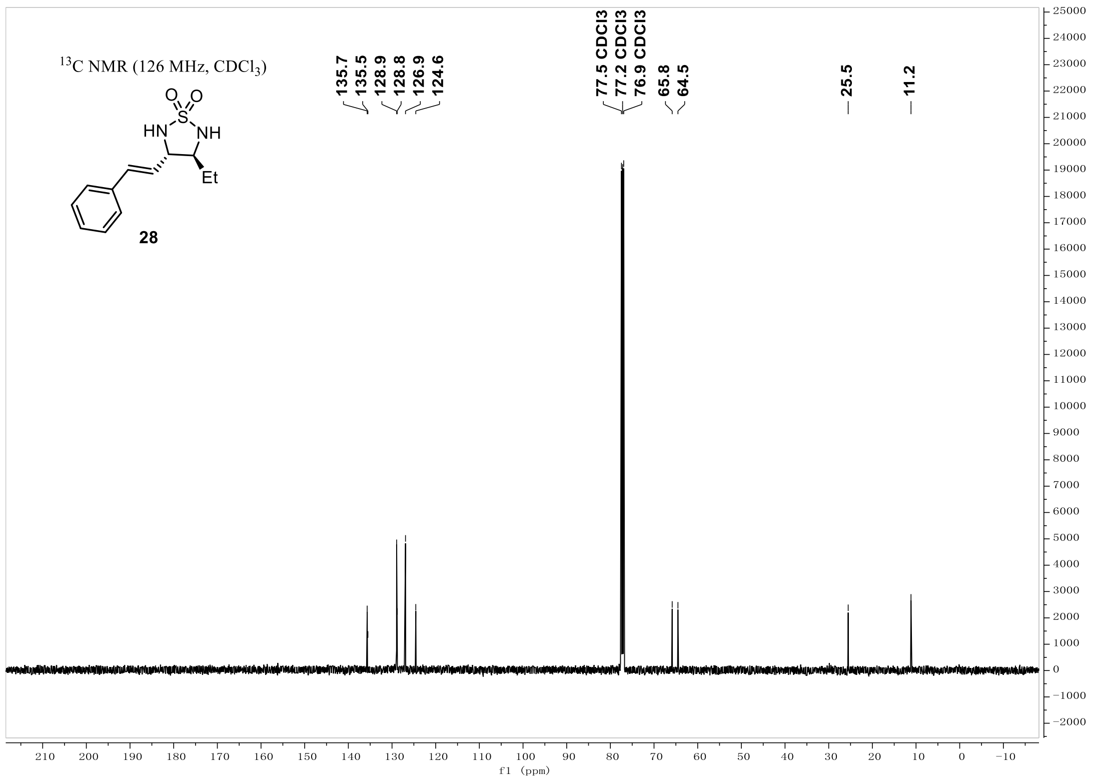

Supplementary Figure 63. ^{1}H NMR and ^{13}C NMR spectra of compound 28.

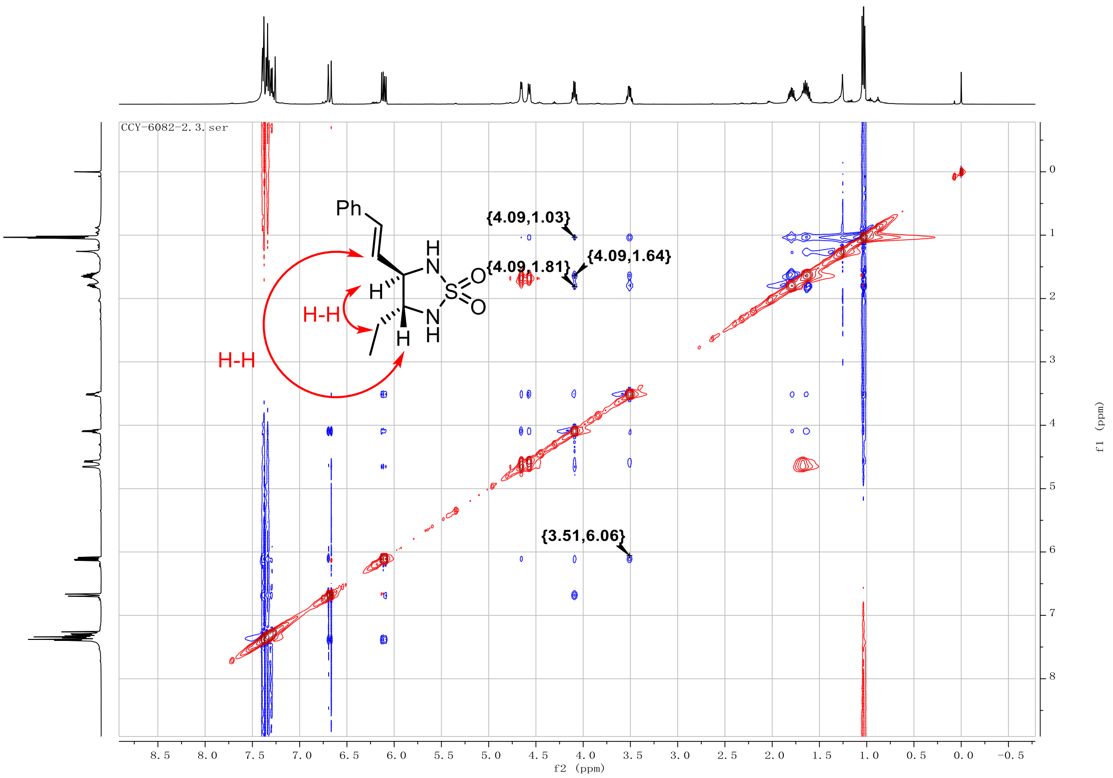

Supplementary Figure 64. 2D NOESY spectra of compound 28.

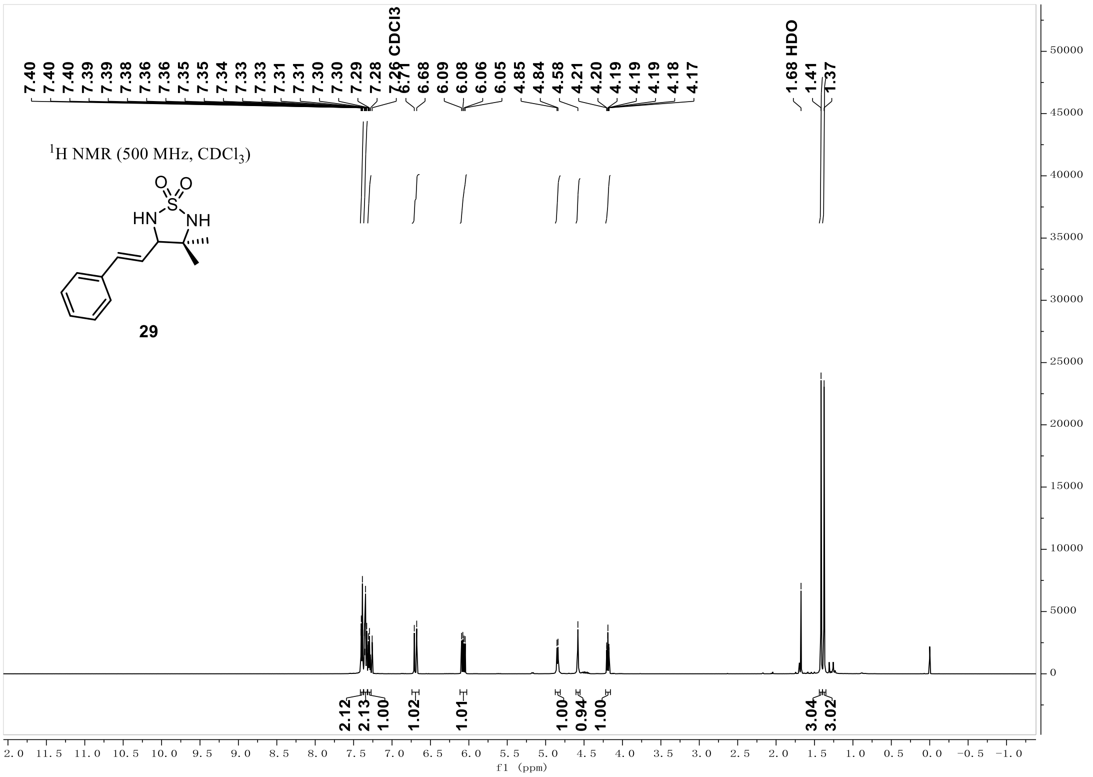

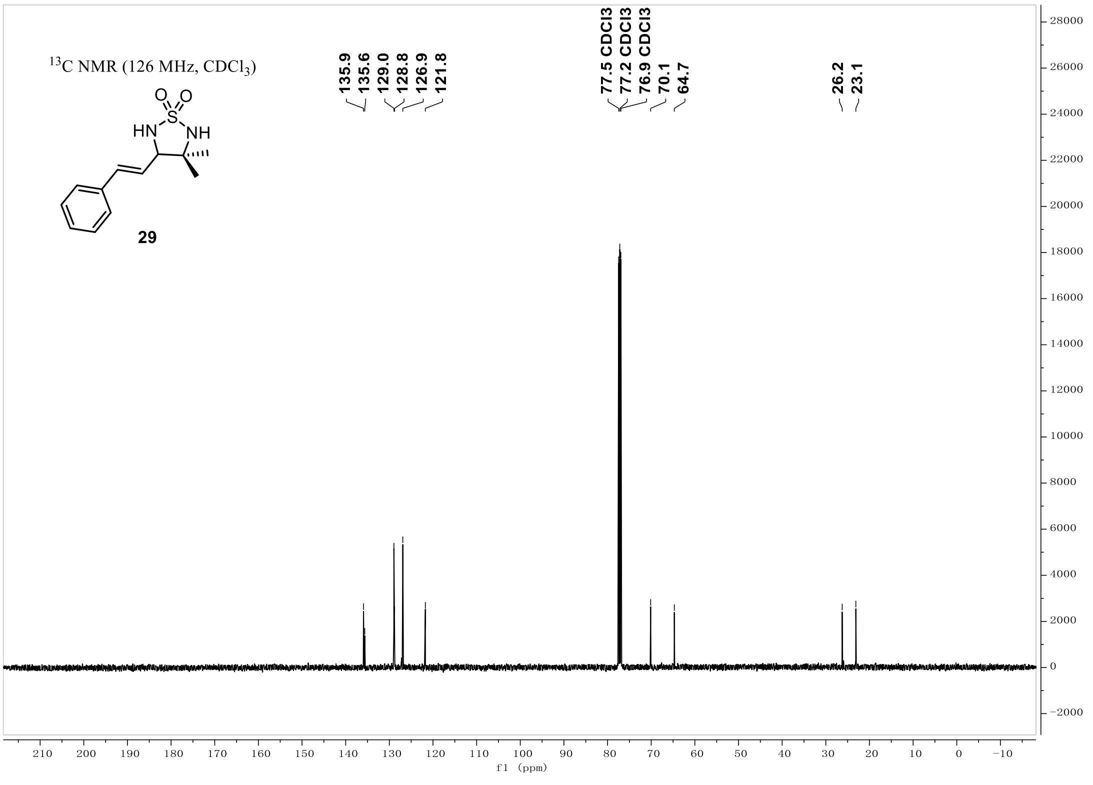

Supplementary Figure 65. ^{1}H NMR and ^{13}C NMR spectra of compound 29.

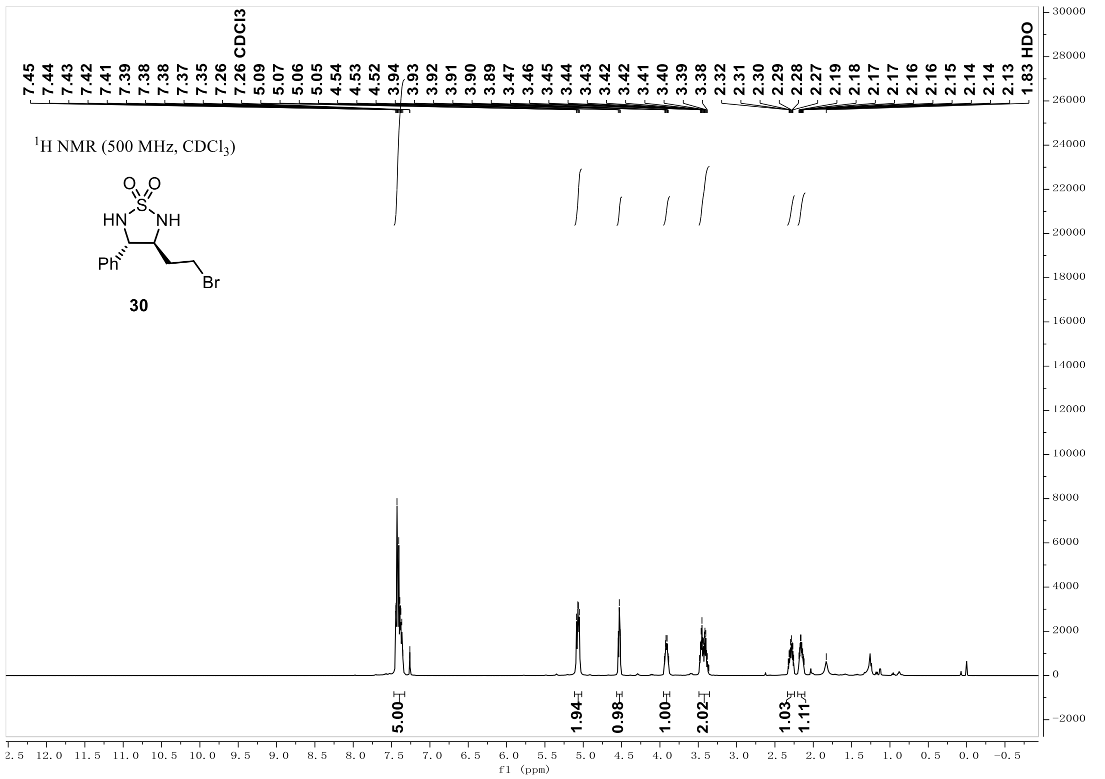

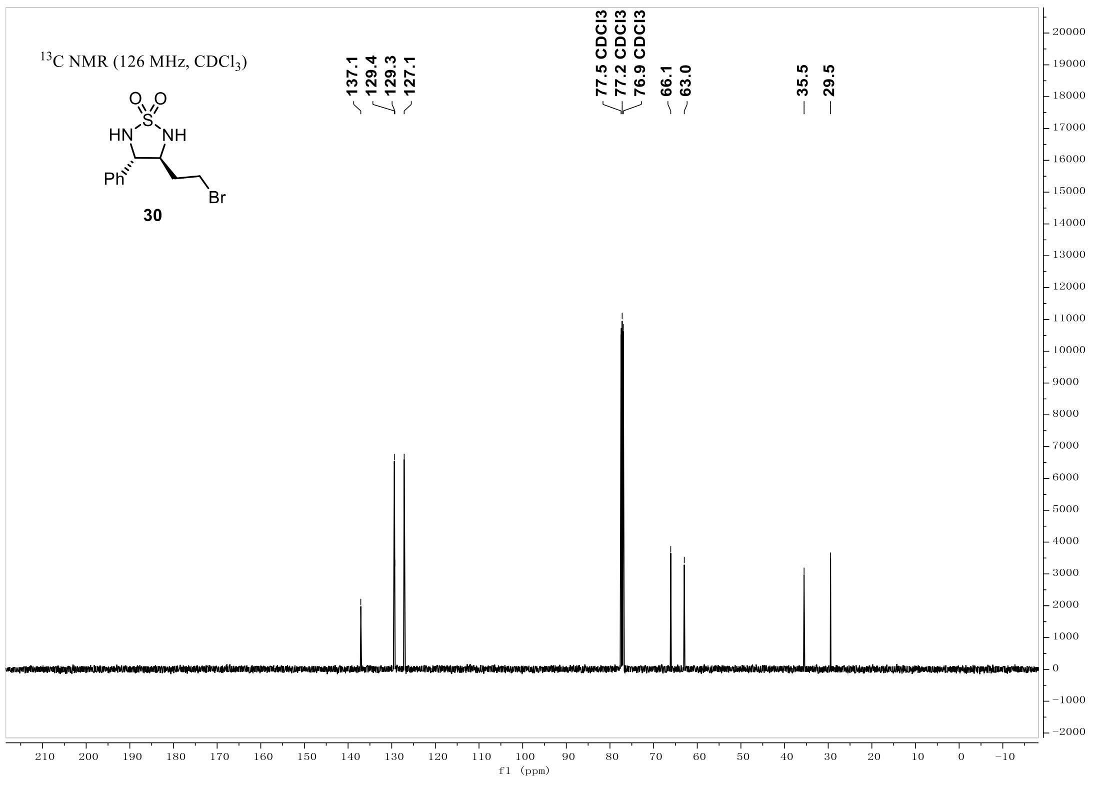

Supplementary Figure 66. ^{1}H NMR and ^{13}C NMR spectra of compound 30.

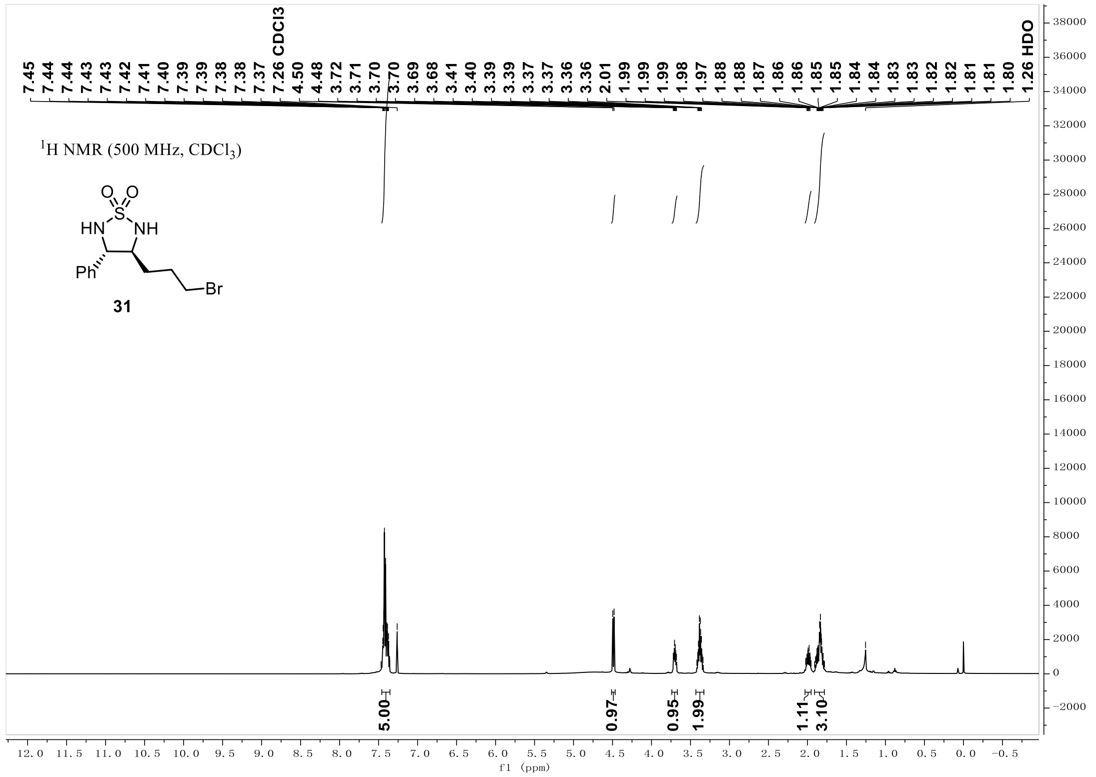

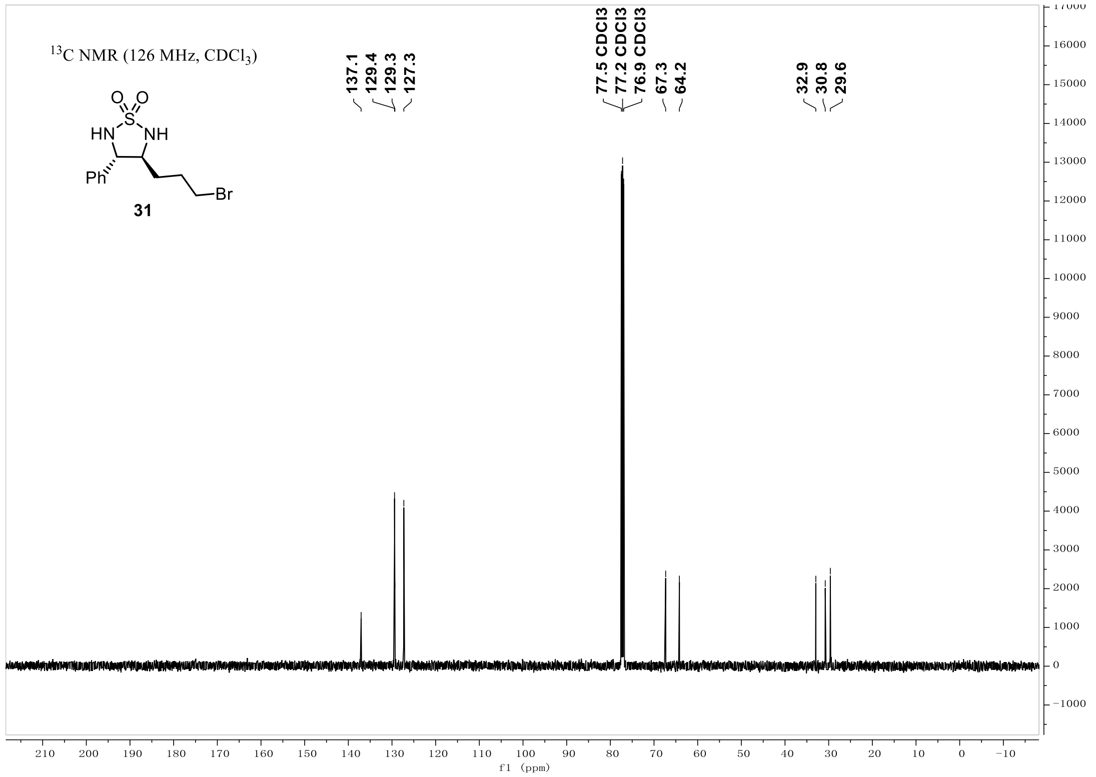

Supplementary Figure 67. ^{1}H NMR and ^{13}C NMR spectra of compound 31.

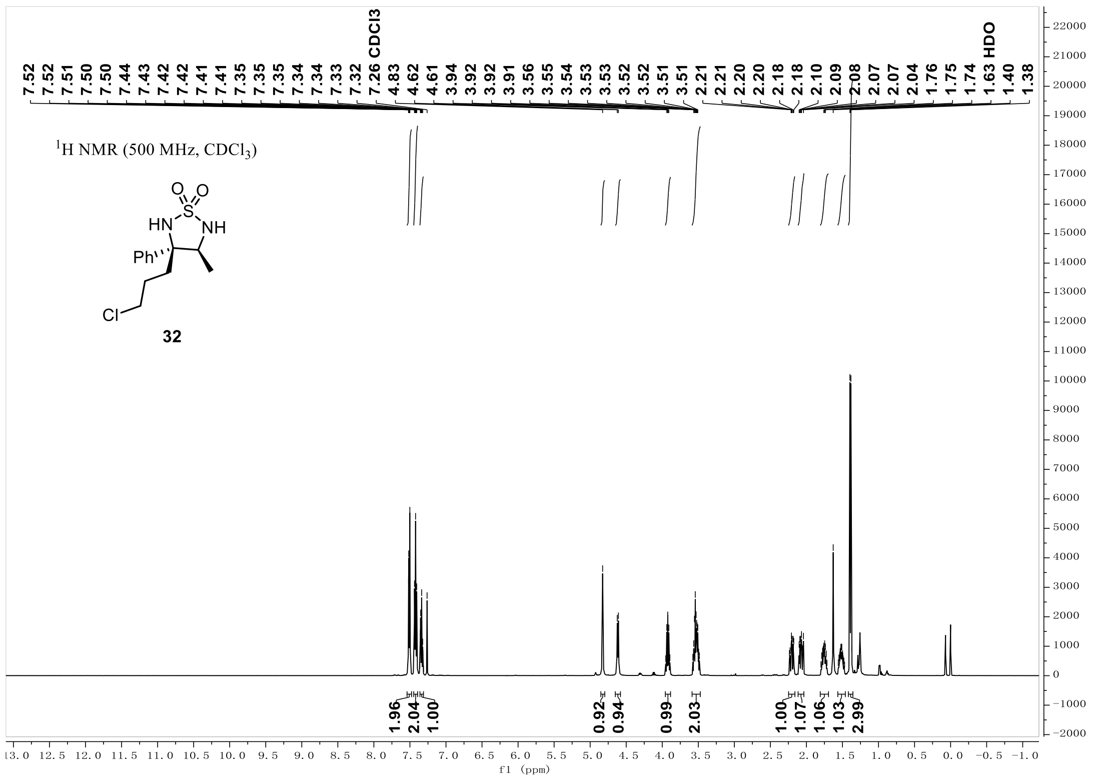

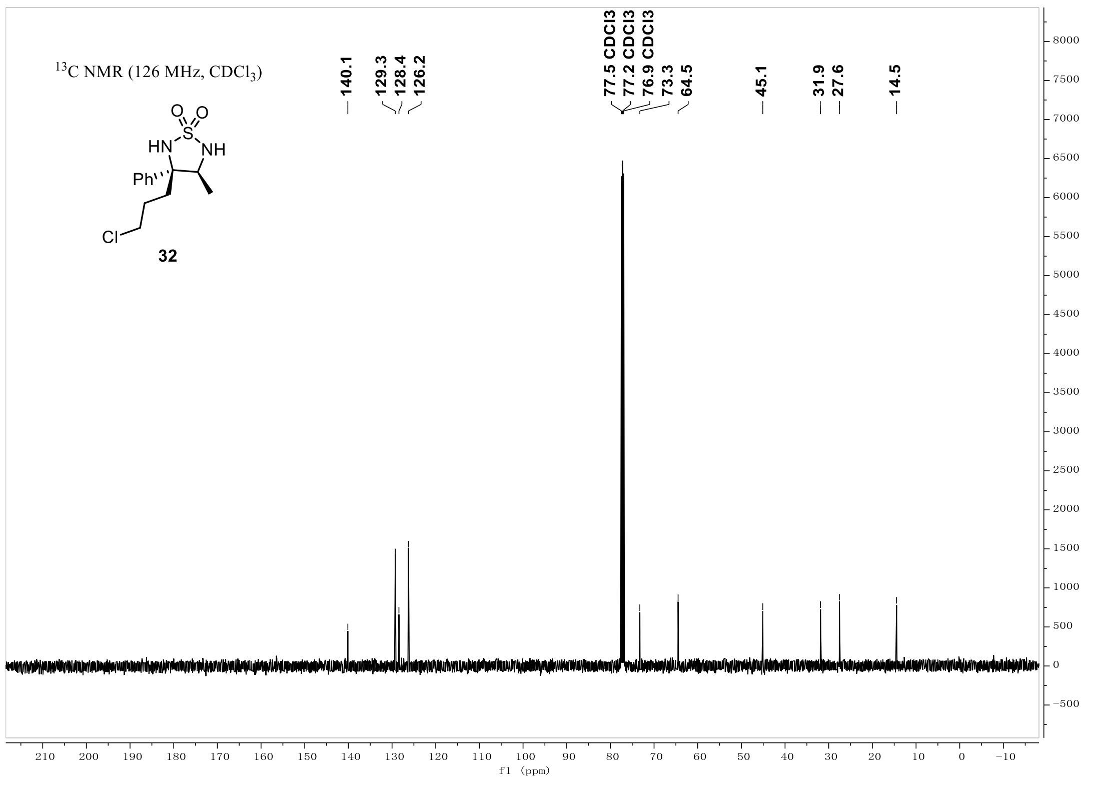

Supplementary Figure 68. ^{1}H NMR and ^{13}C NMR spectra of compound 32.

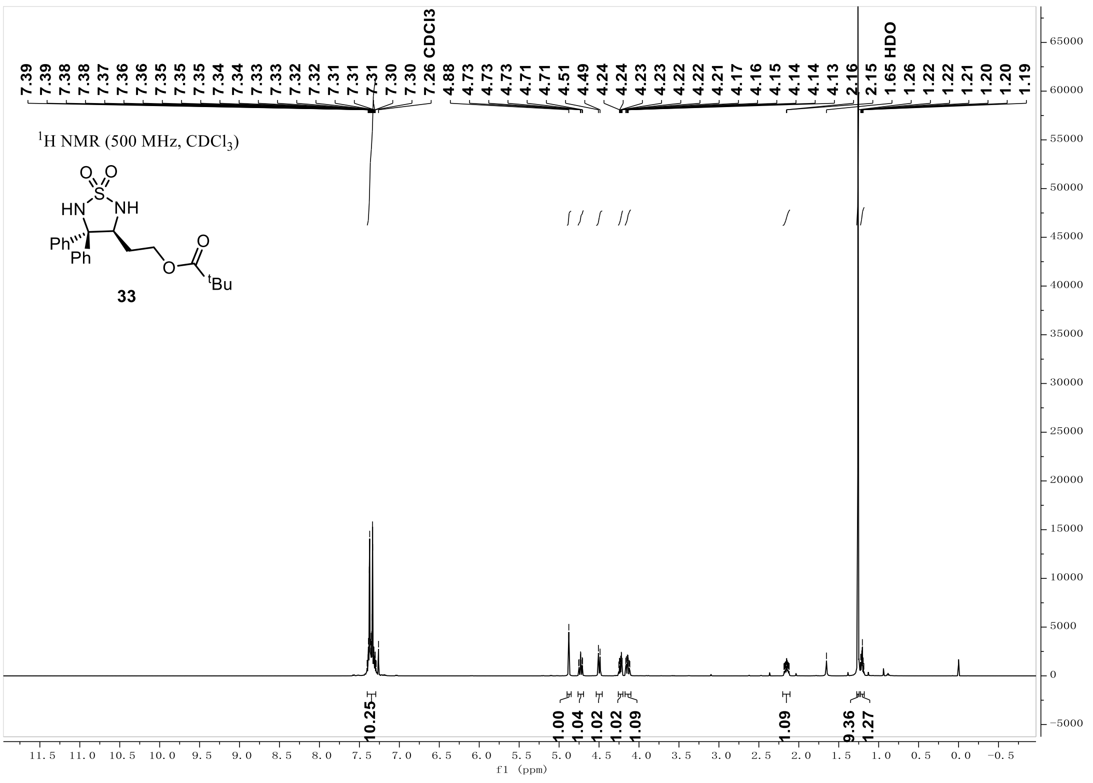

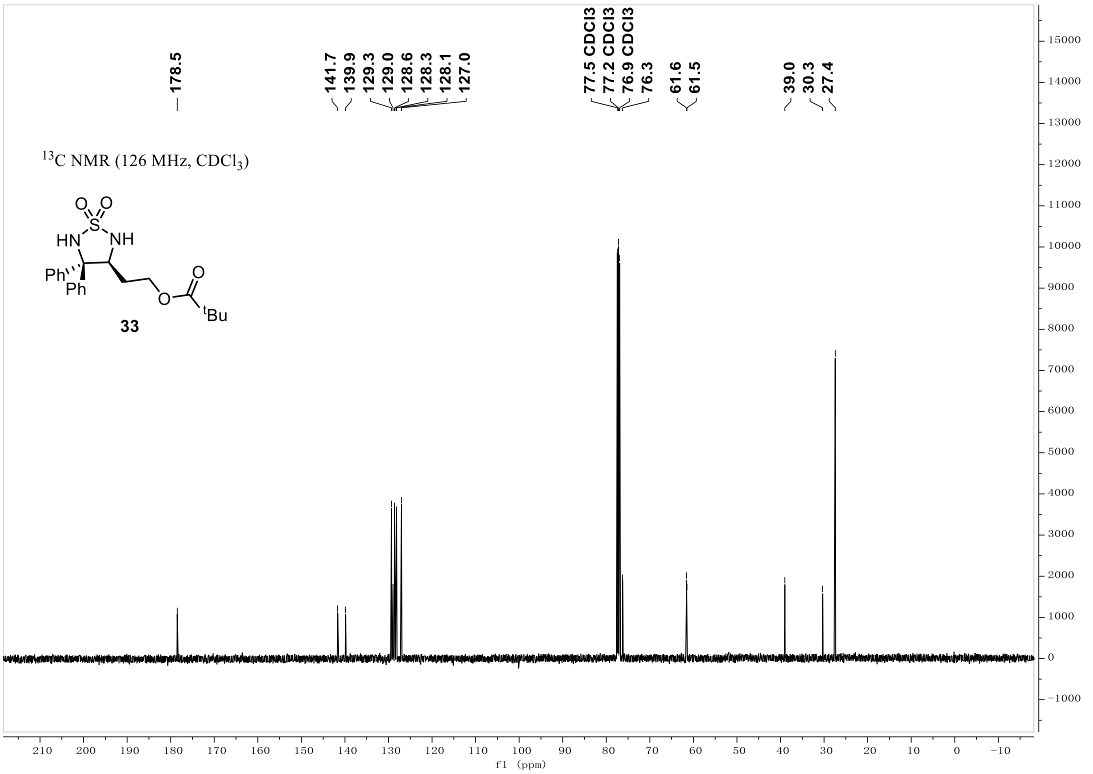

Supplementary Figure 69. ^{1}H NMR and ^{13}C NMR spectra of compound 33.

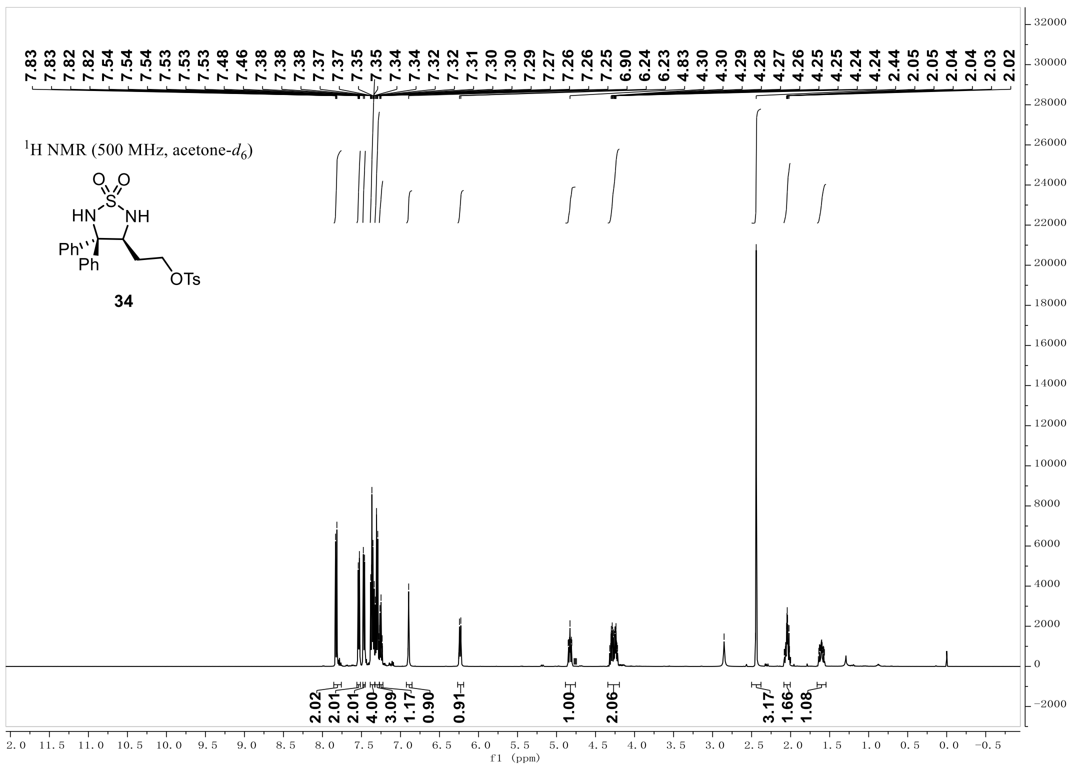

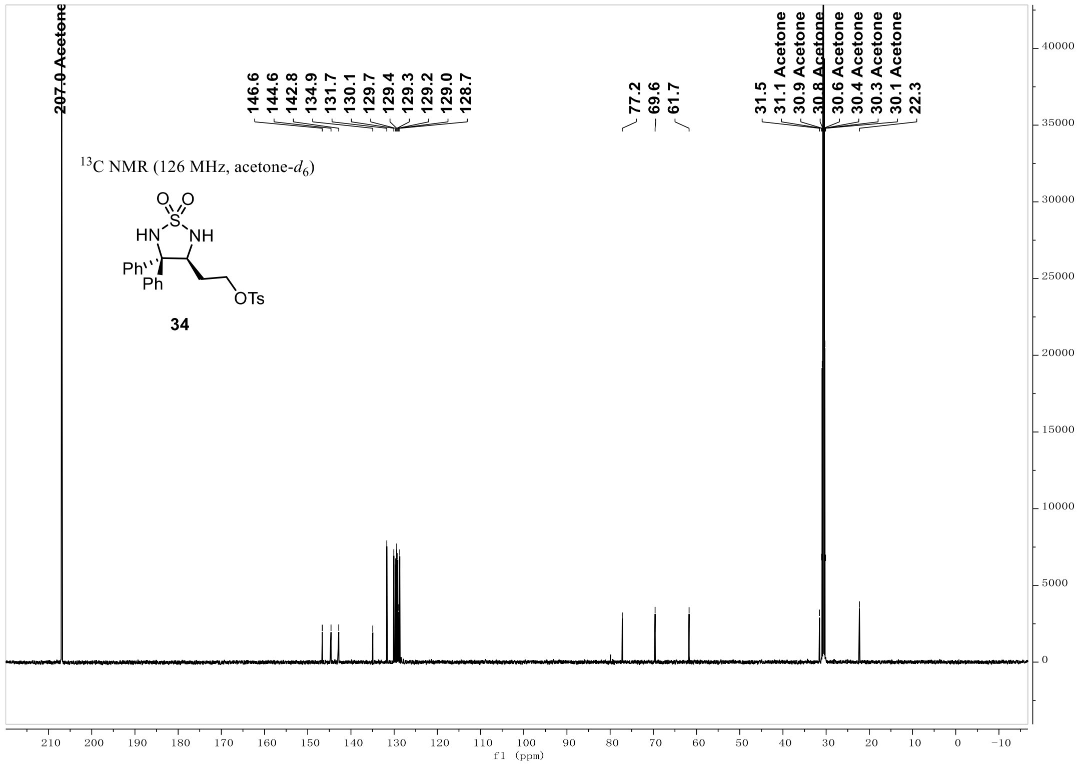

Supplementary Figure 70. ^{1}H NMR and ^{13}C NMR spectra of compound 34.

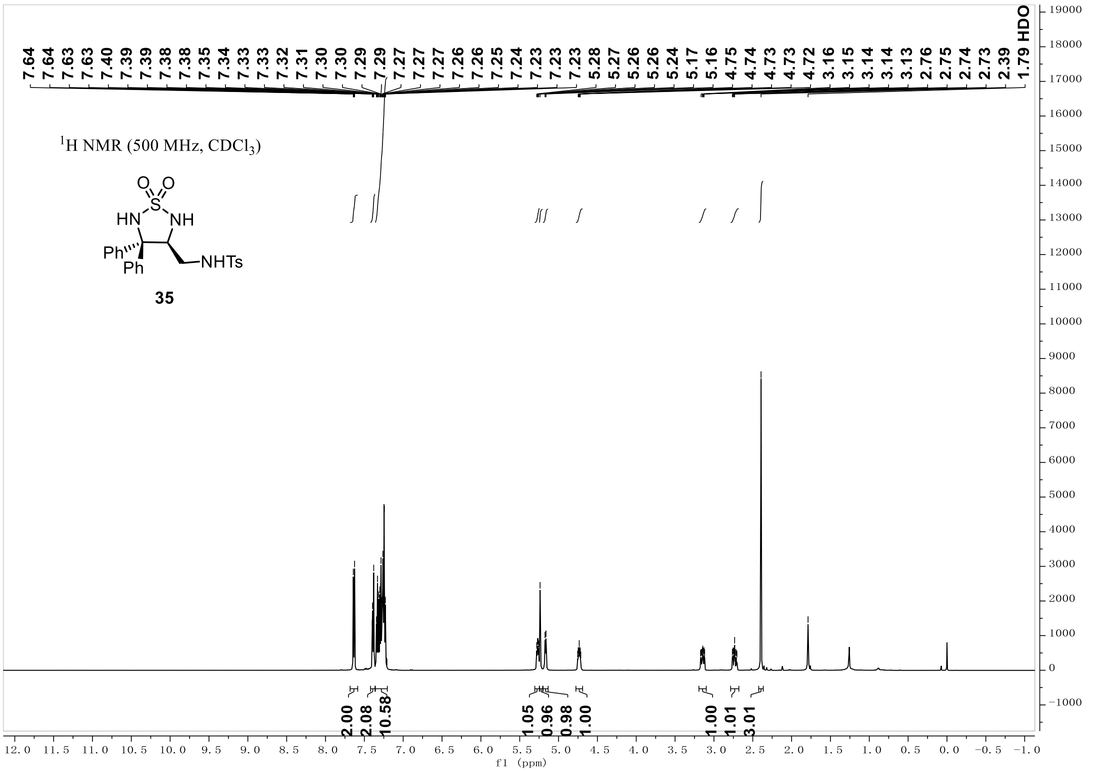

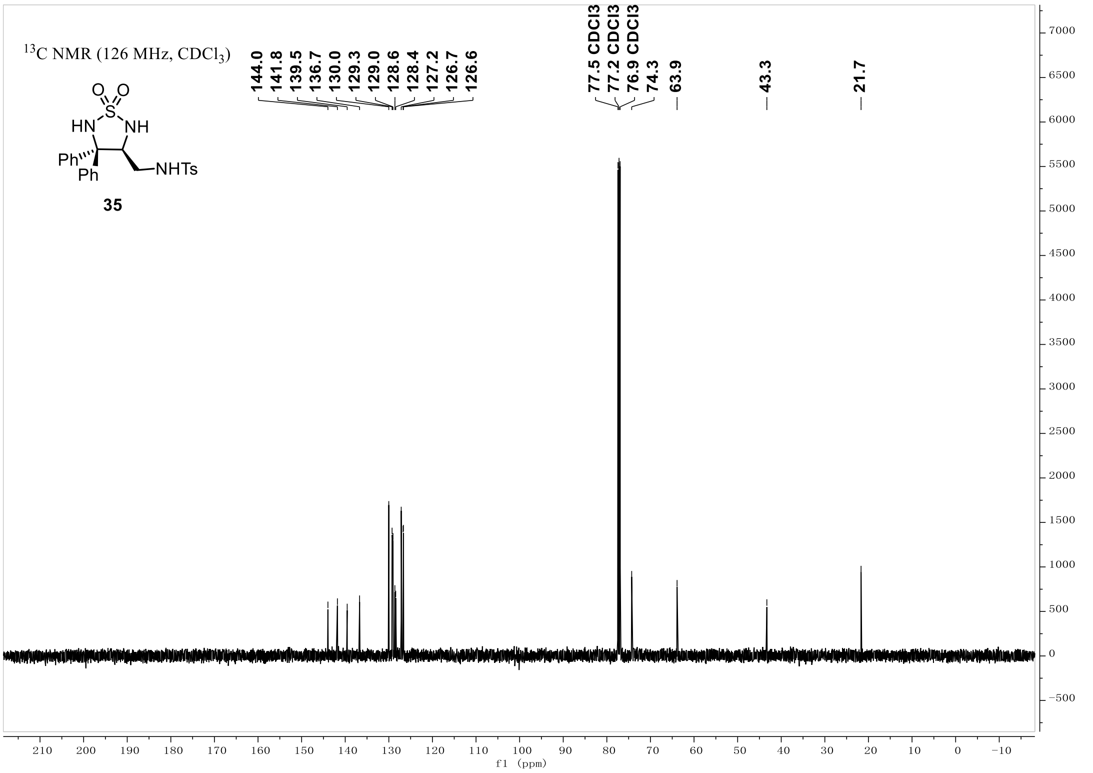

Supplementary Figure 71. ^{1}H NMR and ^{13}C NMR spectra of compound 35.

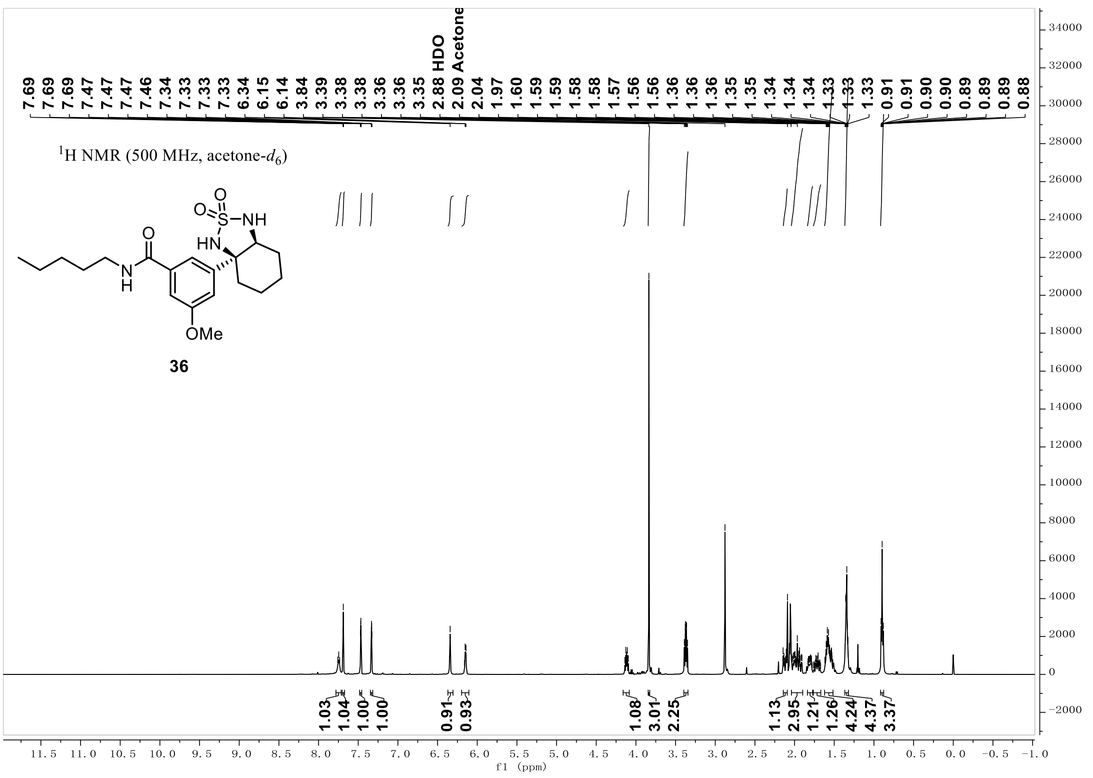

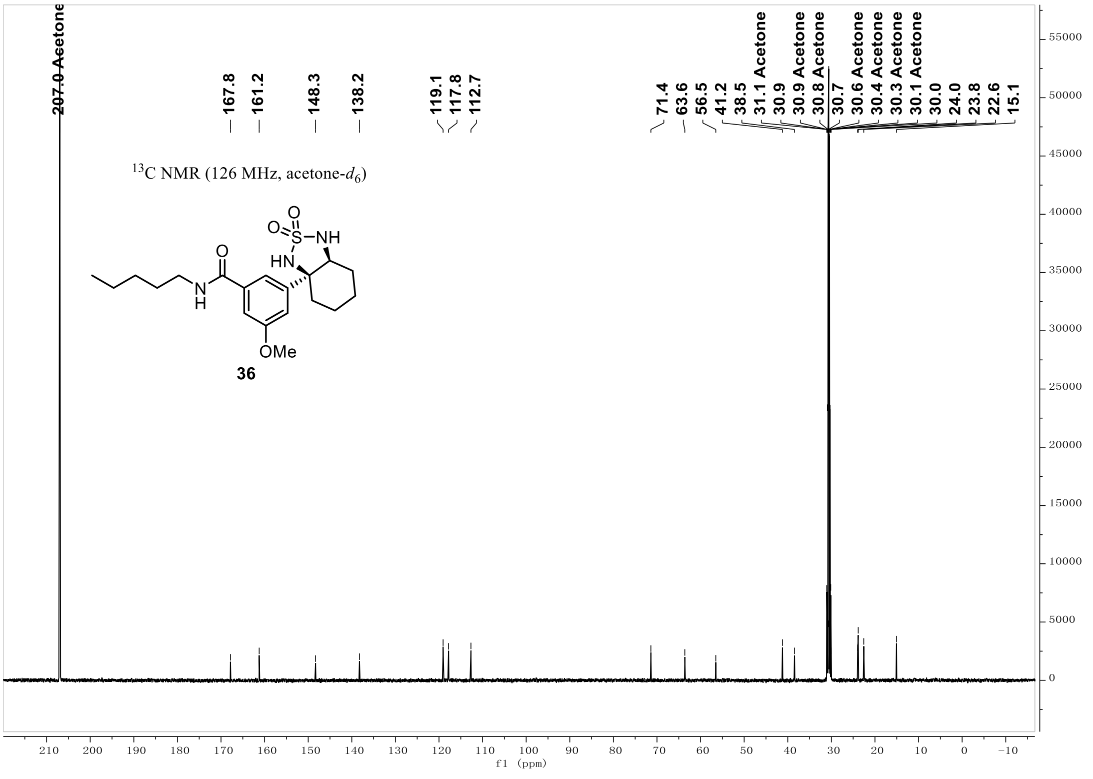

Supplementary Figure 72. ^{1}H NMR and ^{13}C NMR spectra of compound 36.

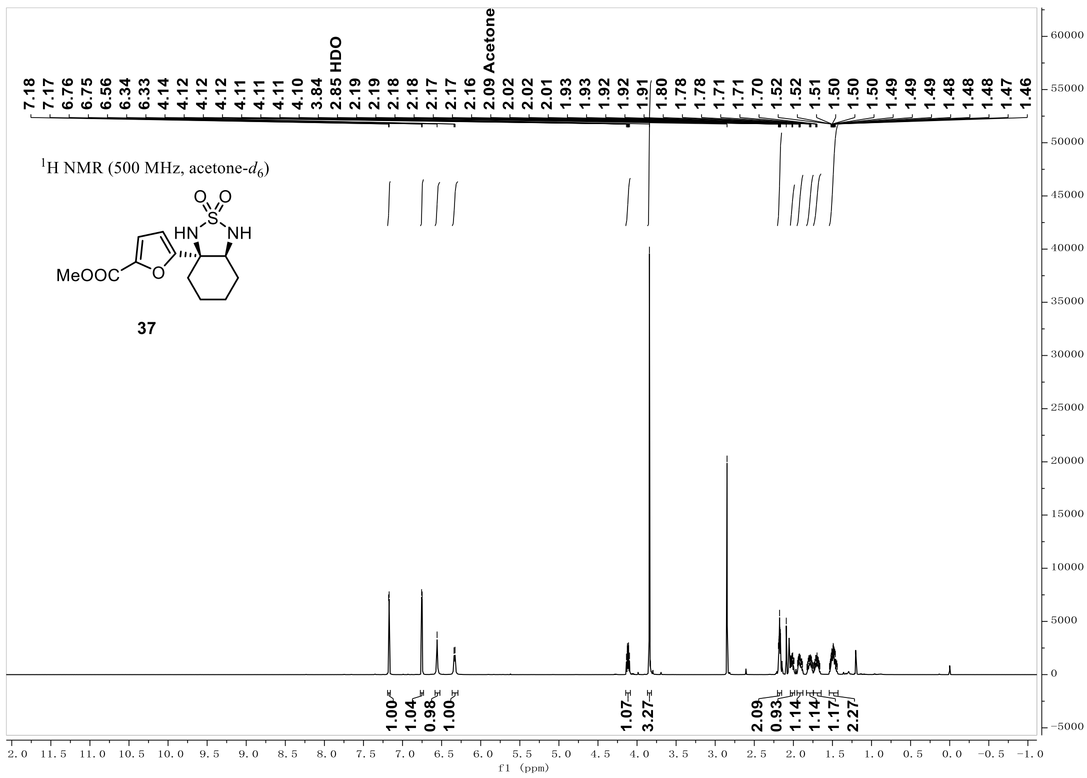

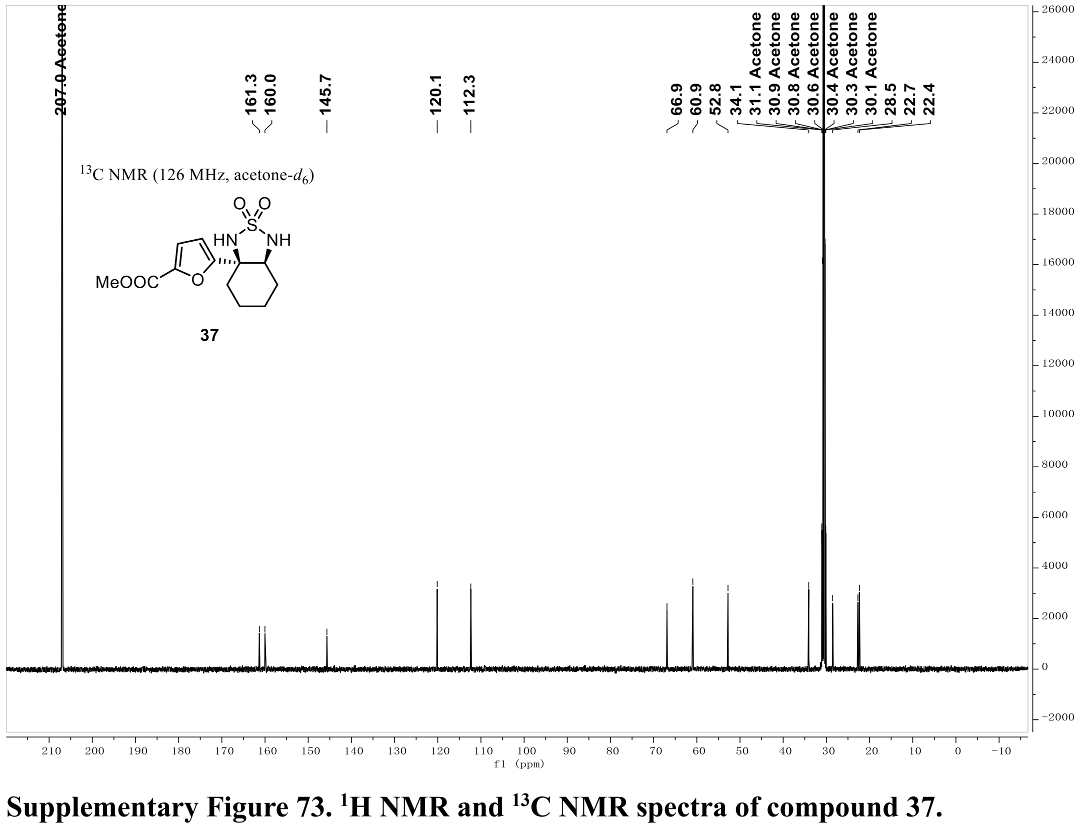

Supplementary Figure 73. ^{1}H NMR and ^{13}C NMR spectra of compound 37.

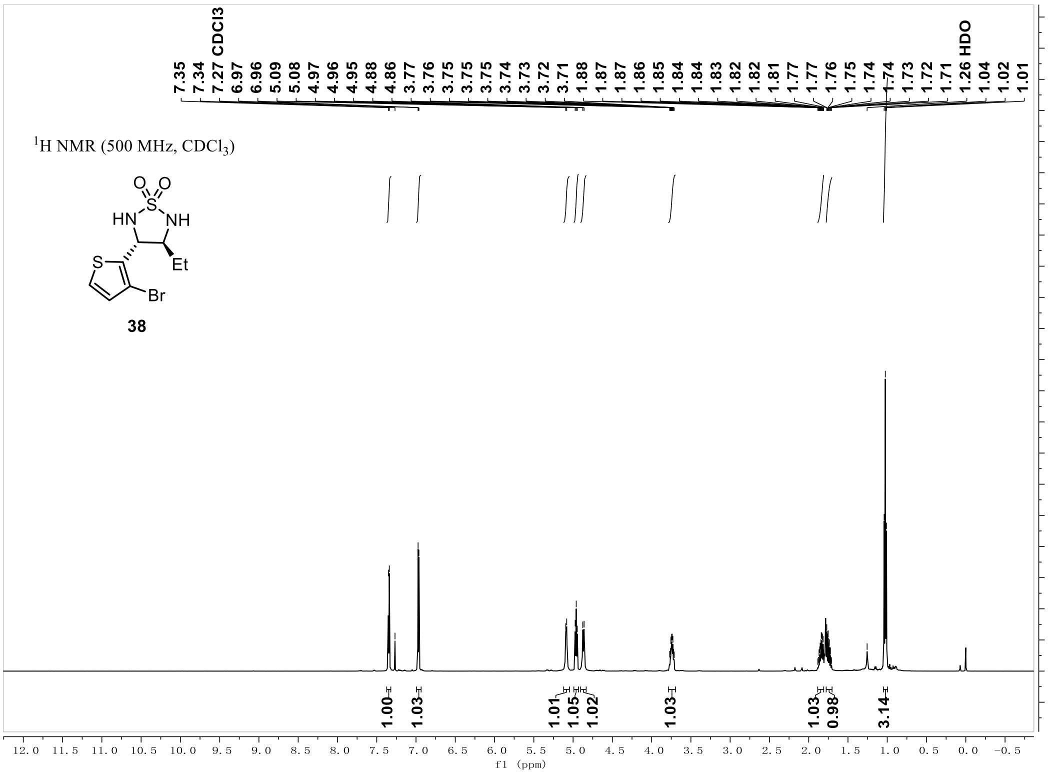

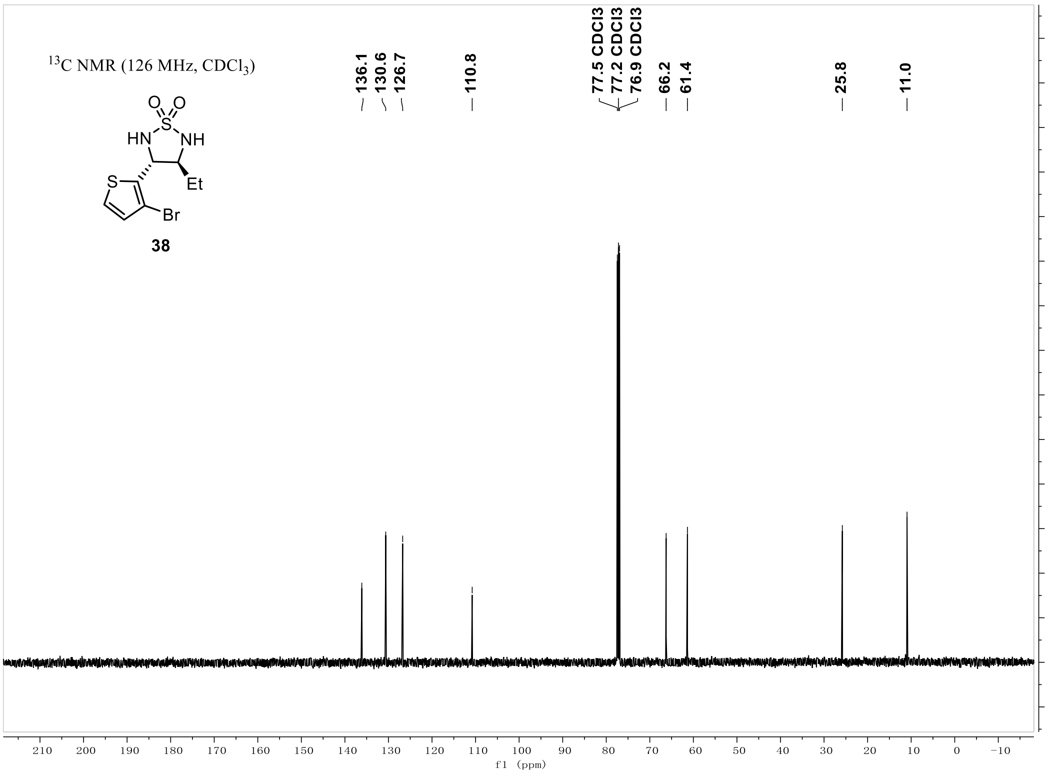

Supplementary Figure 74. ^{1}H NMR and ^{13}C NMR spectra of compound 38.

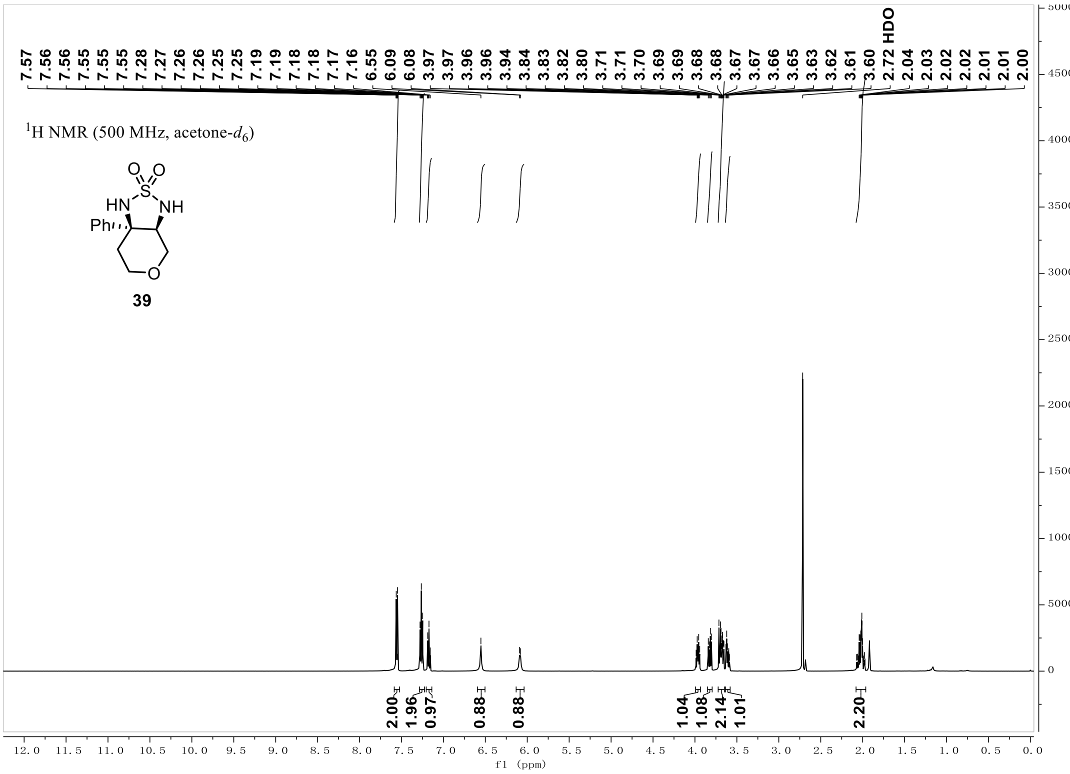

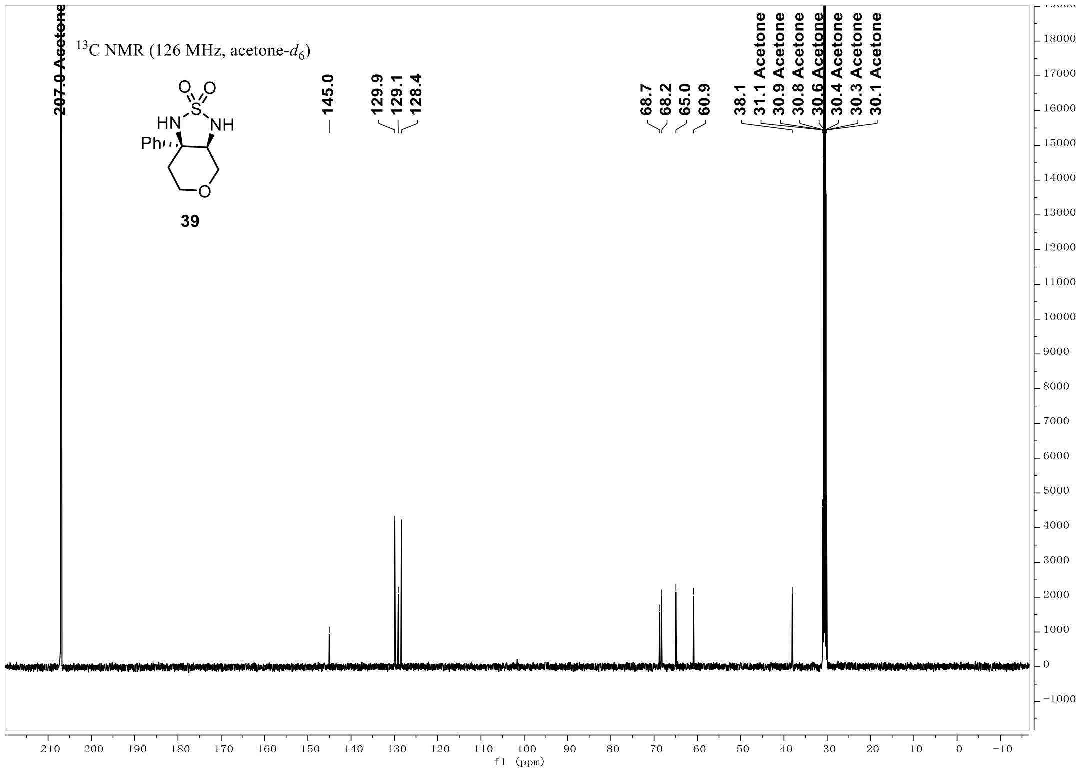

Supplementary Figure 75. ^{1}H NMR and ^{13}C NMR spectra of compound 39.

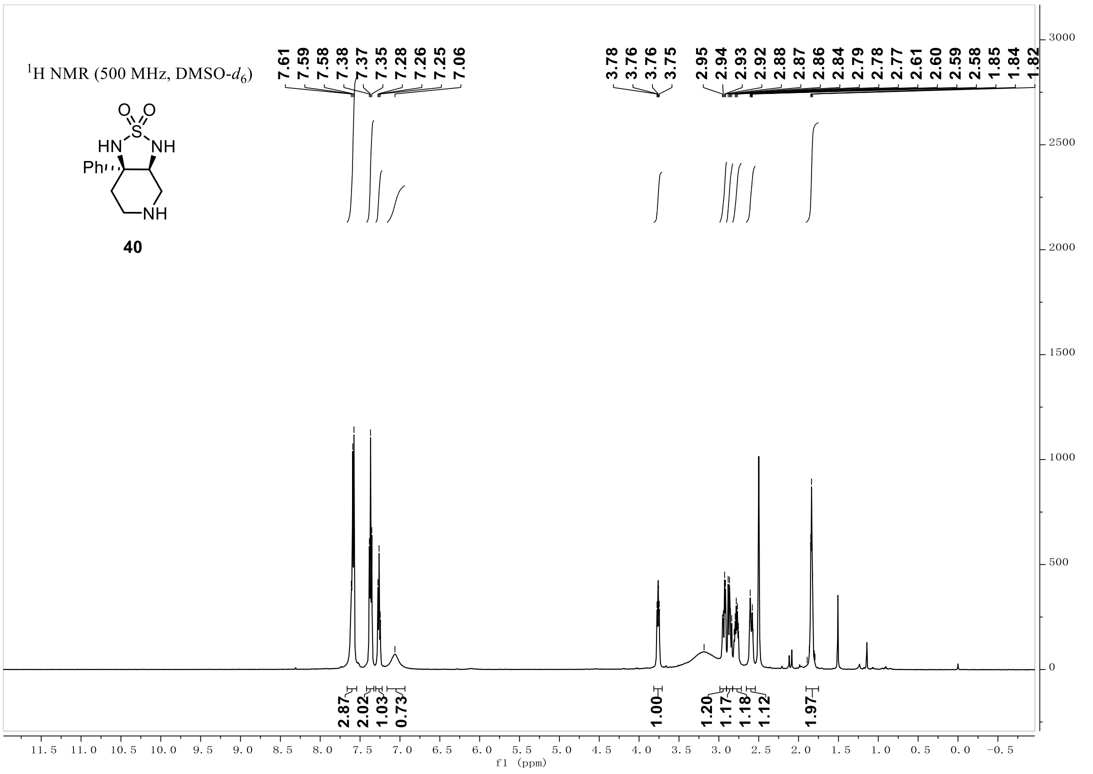

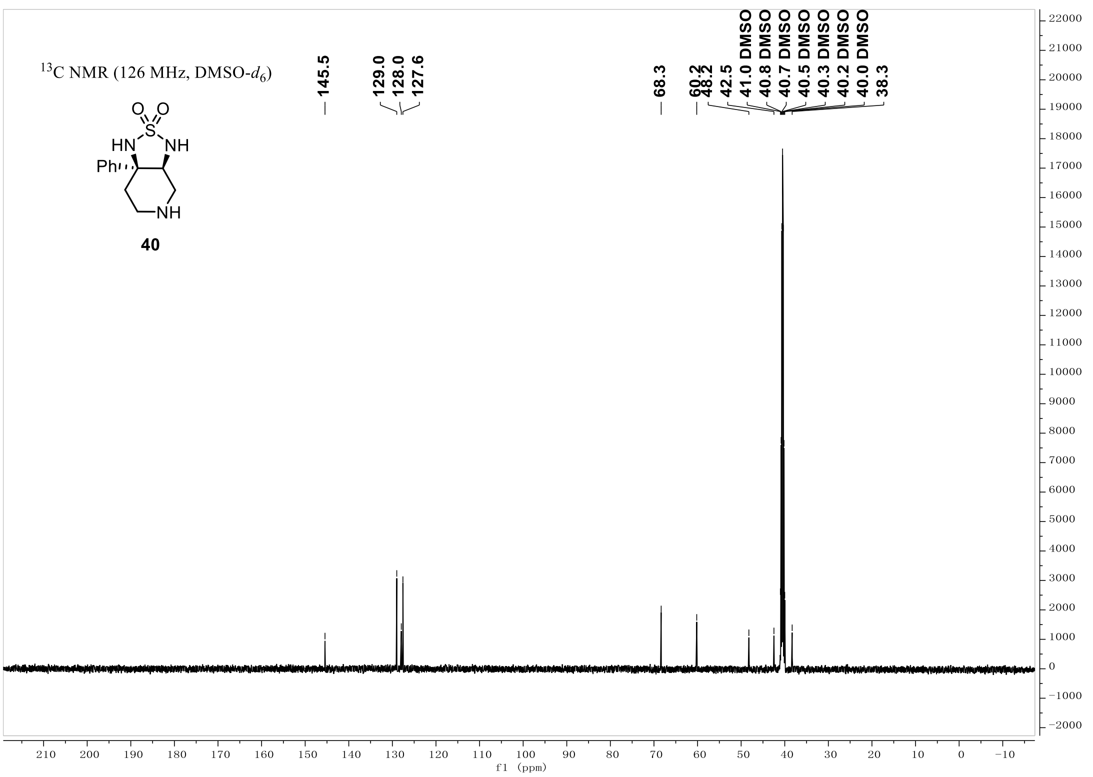

Supplementary Figure 76. ^{1}H NMR and ^{13}C NMR spectra of compound 40.

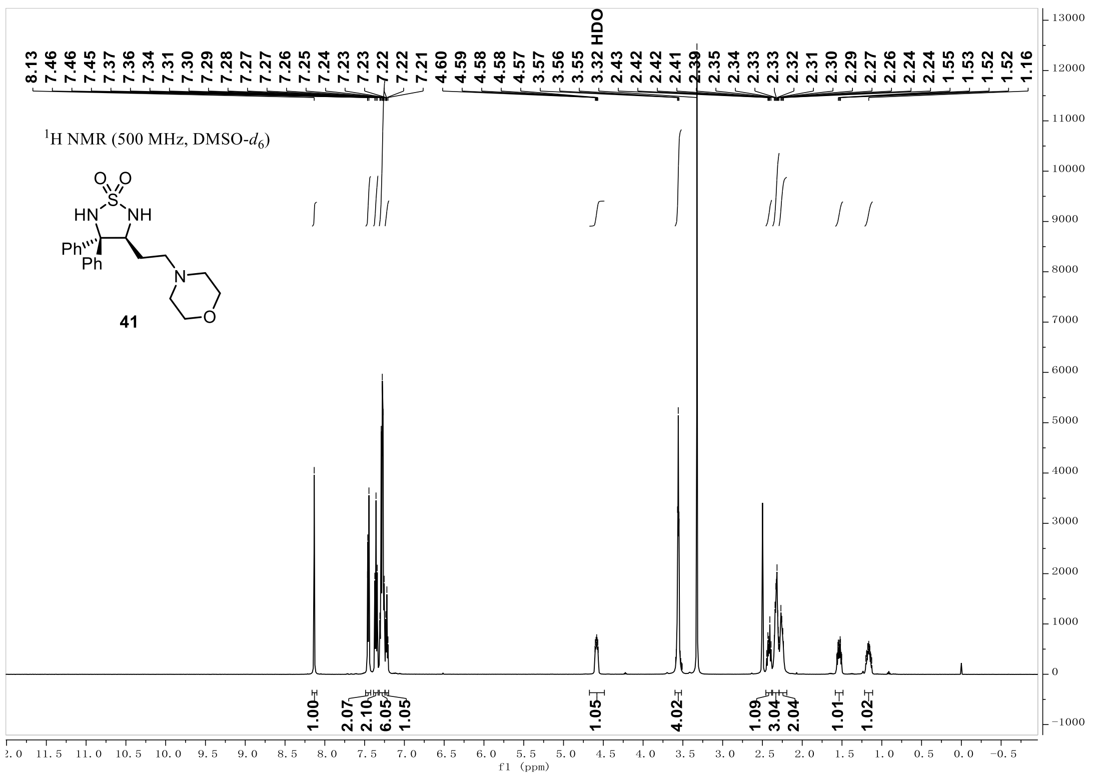

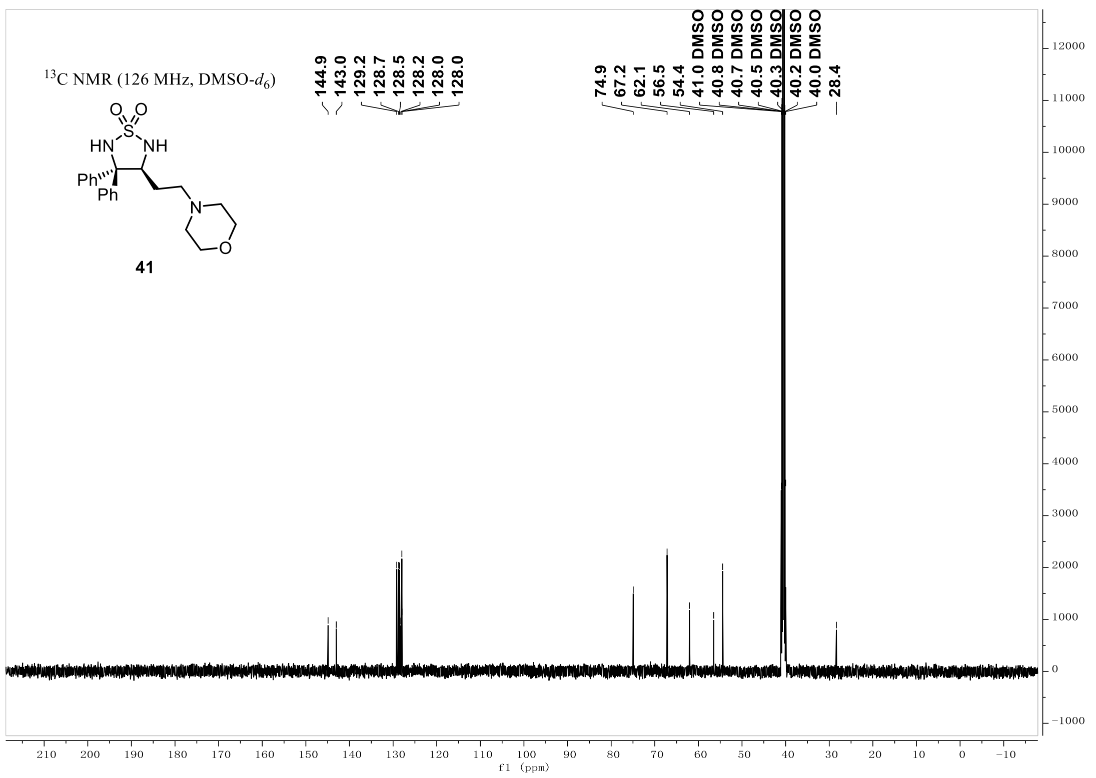

Supplementary Figure 77. ^{1}H NMR and ^{13}C NMR spectra of compound 41.

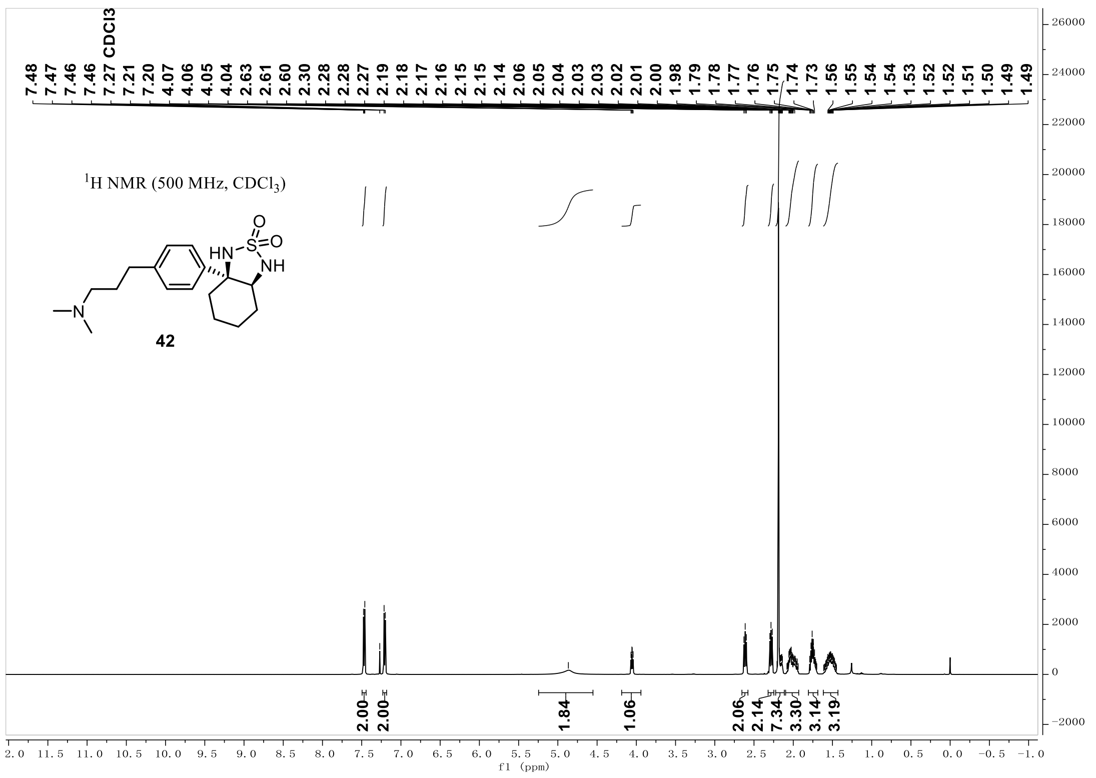

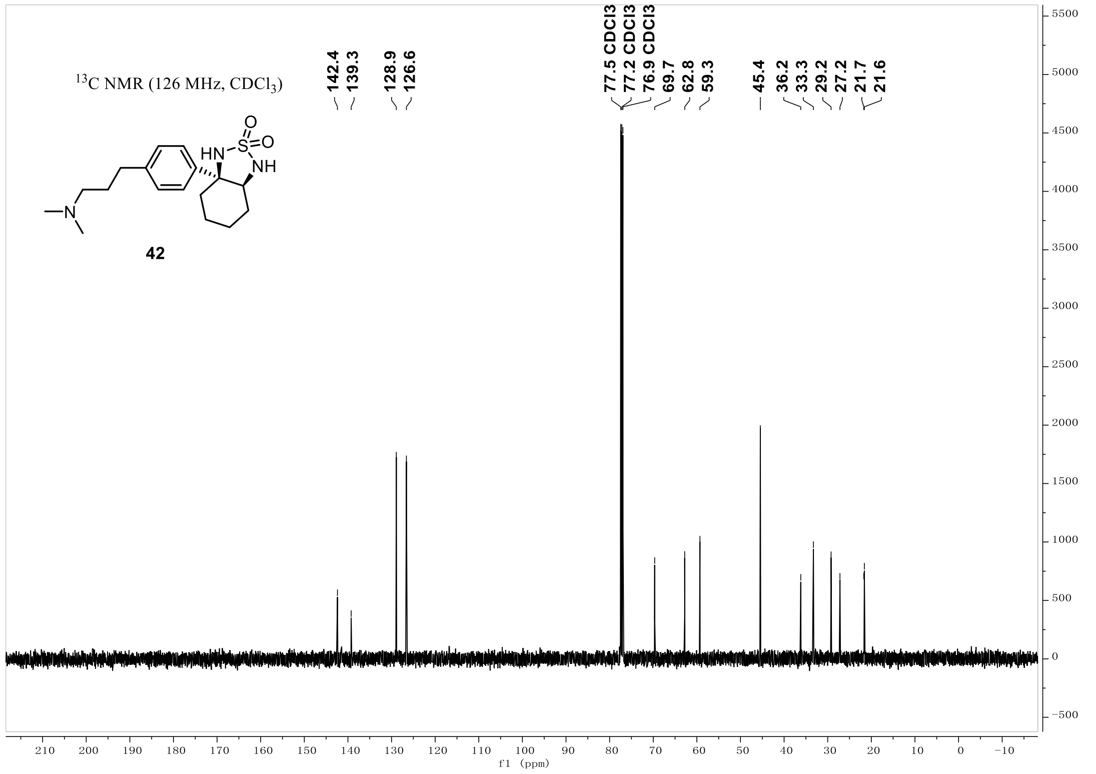

Supplementary Figure 78. ^{1}H NMR and ^{13}C NMR spectra of compound 42.

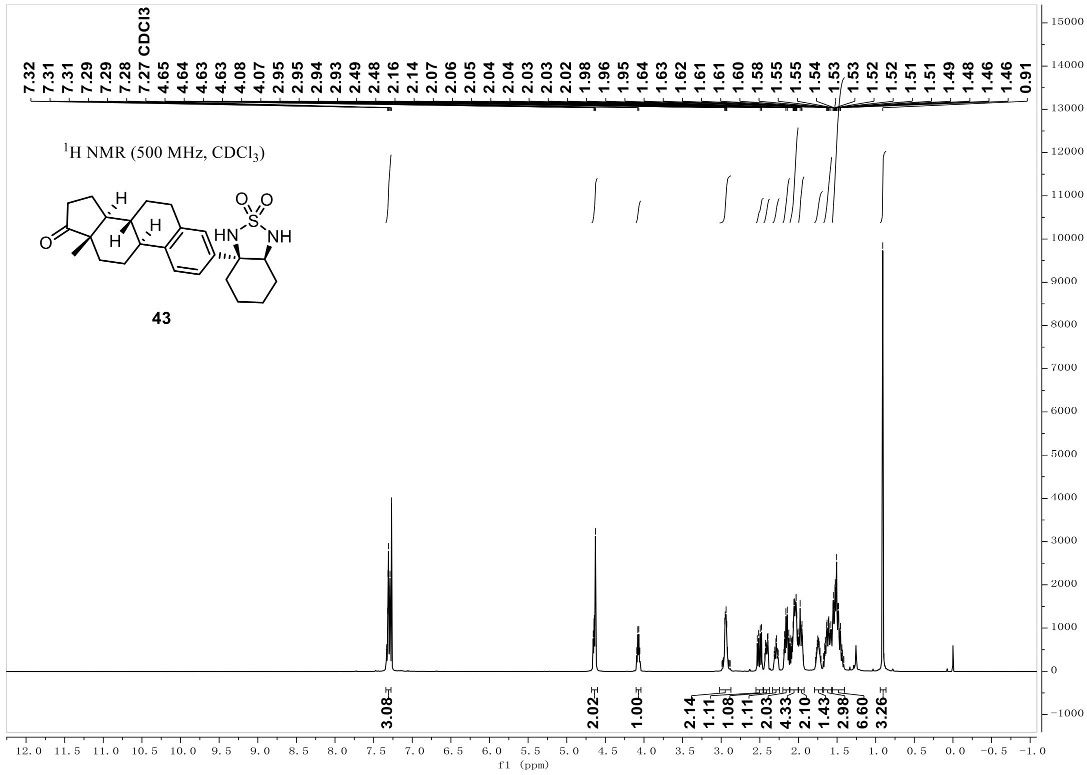

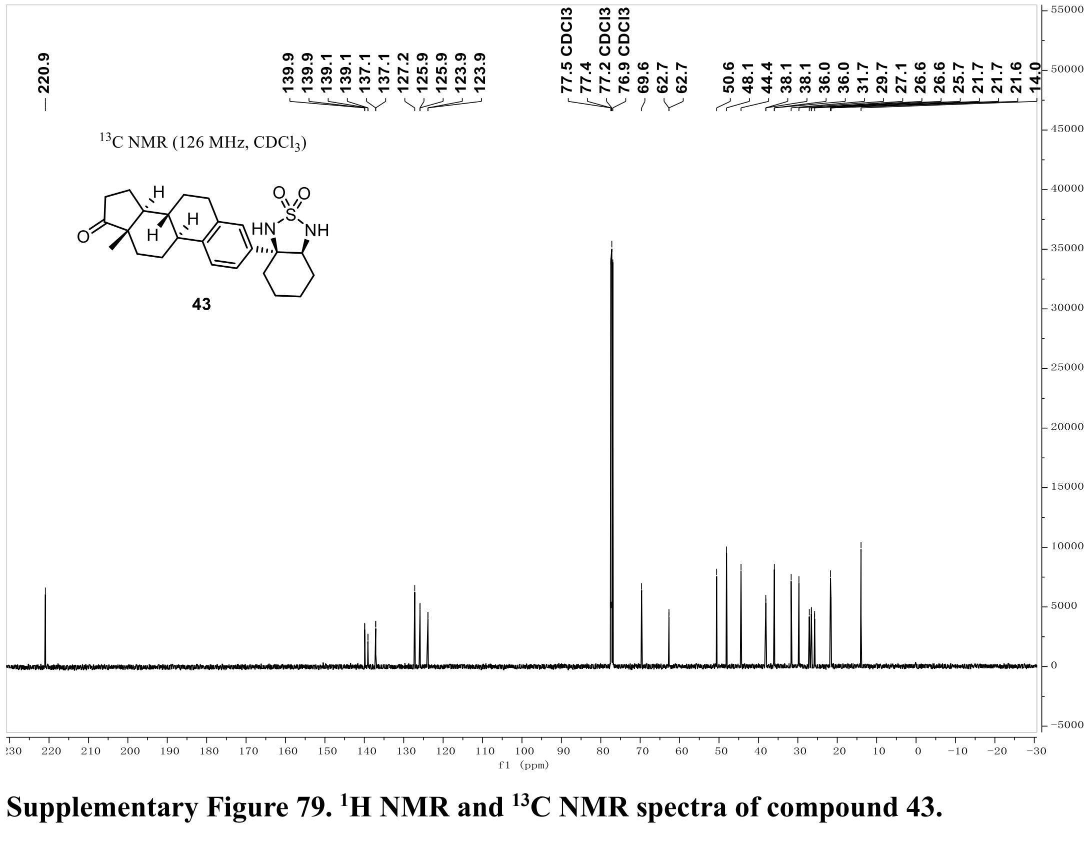

Supplementary Figure 79. ^{1}H NMR and ^{13}C NMR spectra of compound 43.

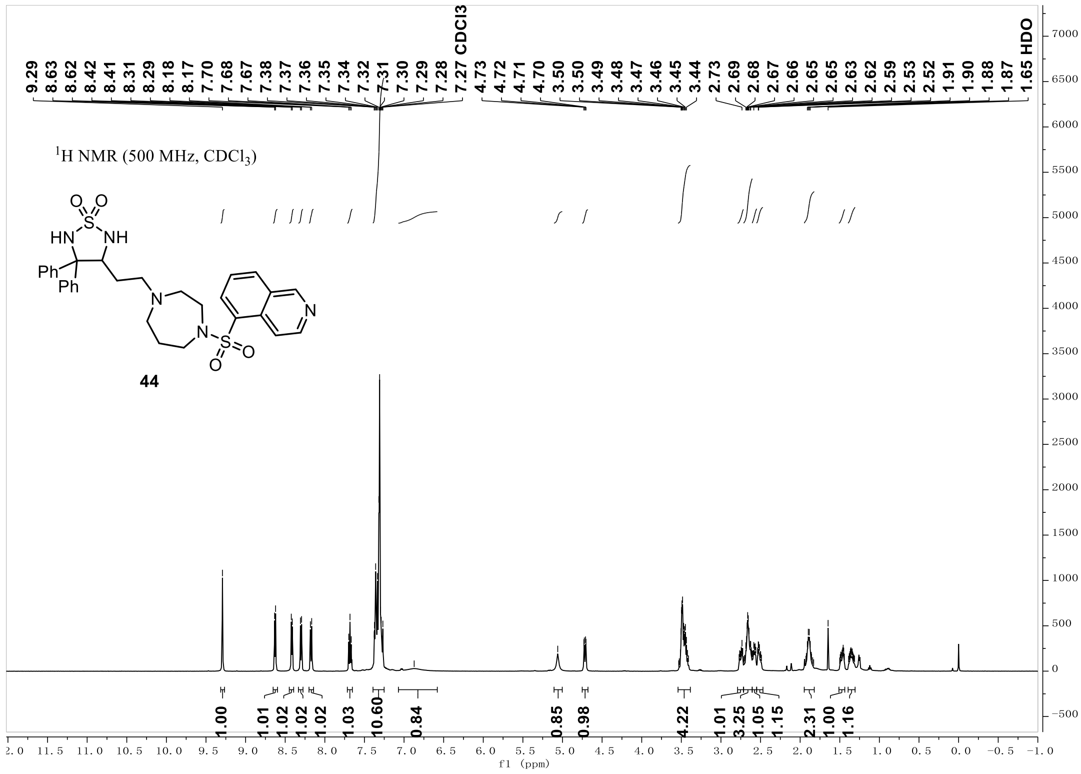

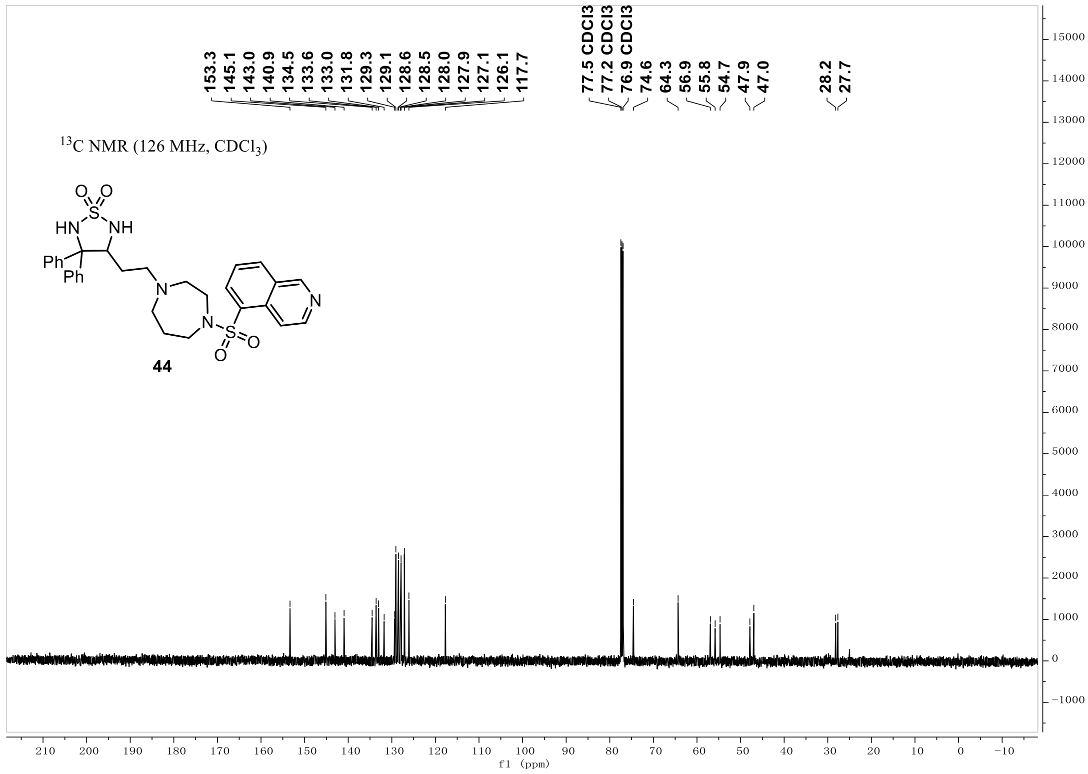

Supplementary Figure 80. ^{1}H NMR and ^{13}C NMR spectra of compound 44.

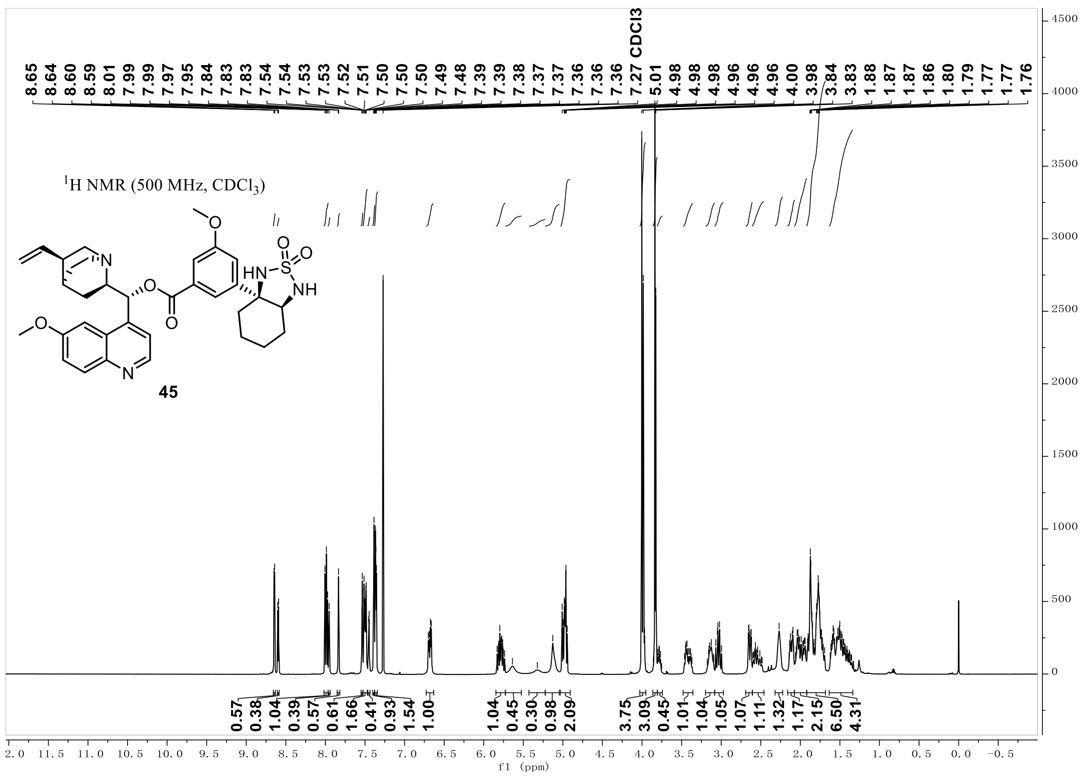

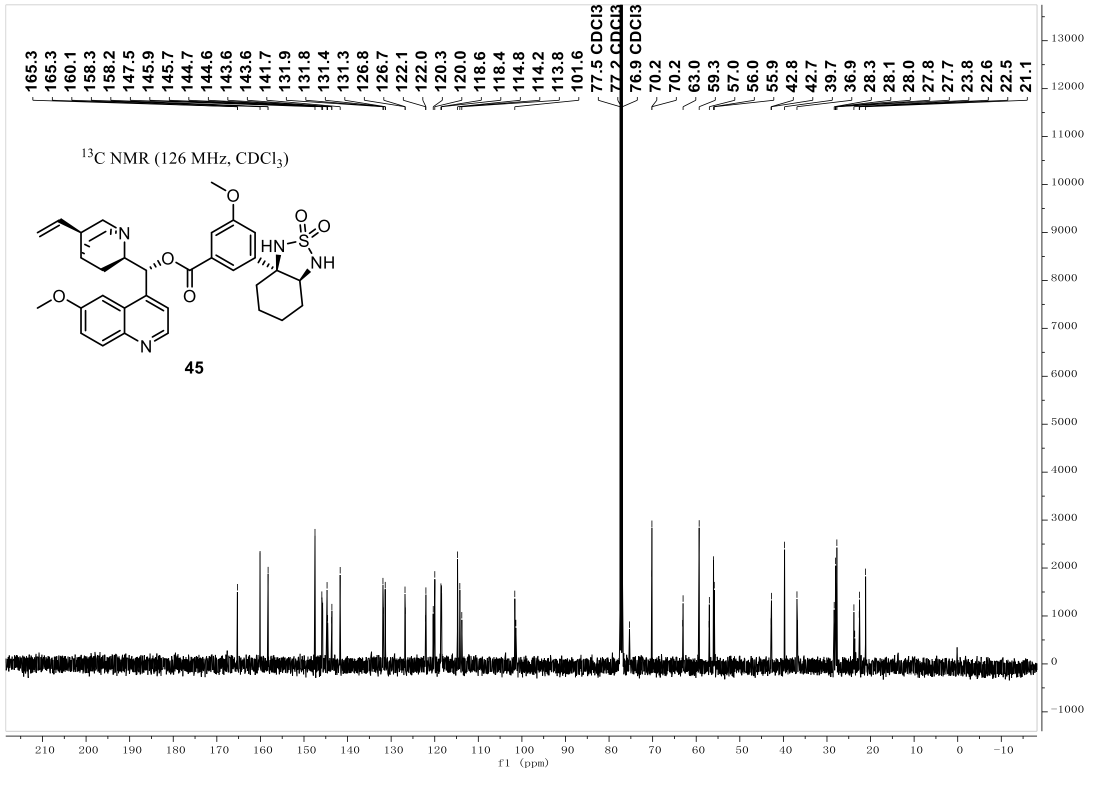

Supplementary Figure 81. ^{1}H NMR and ^{13}C NMR spectra of compound 45.

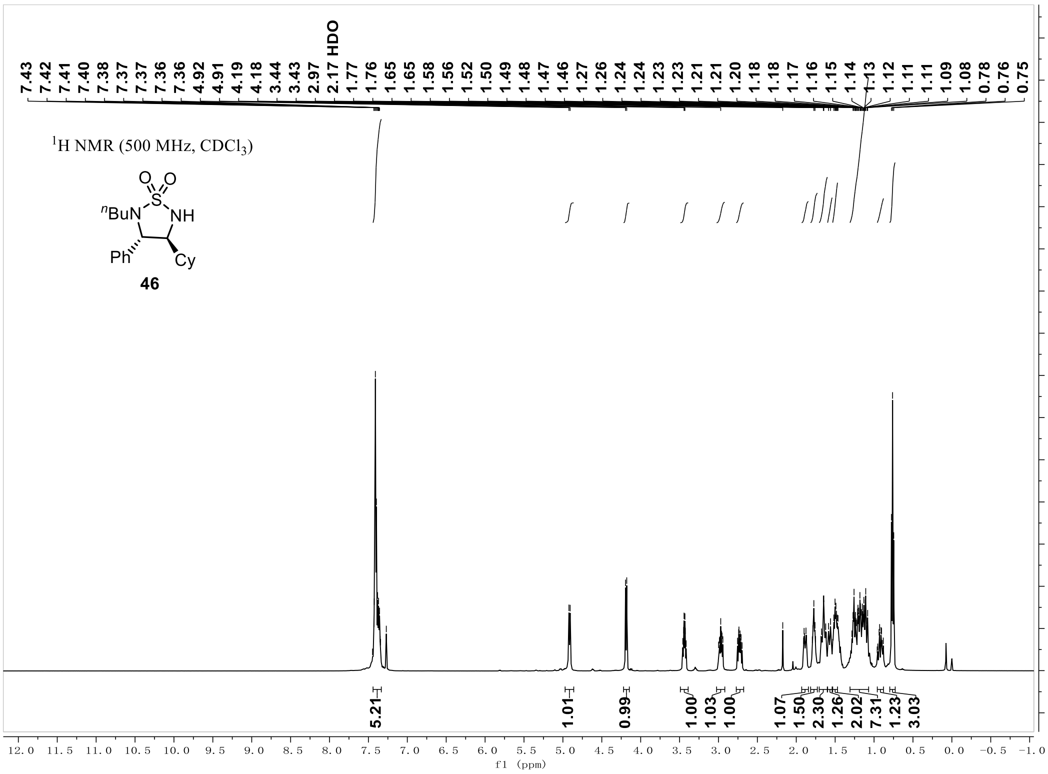

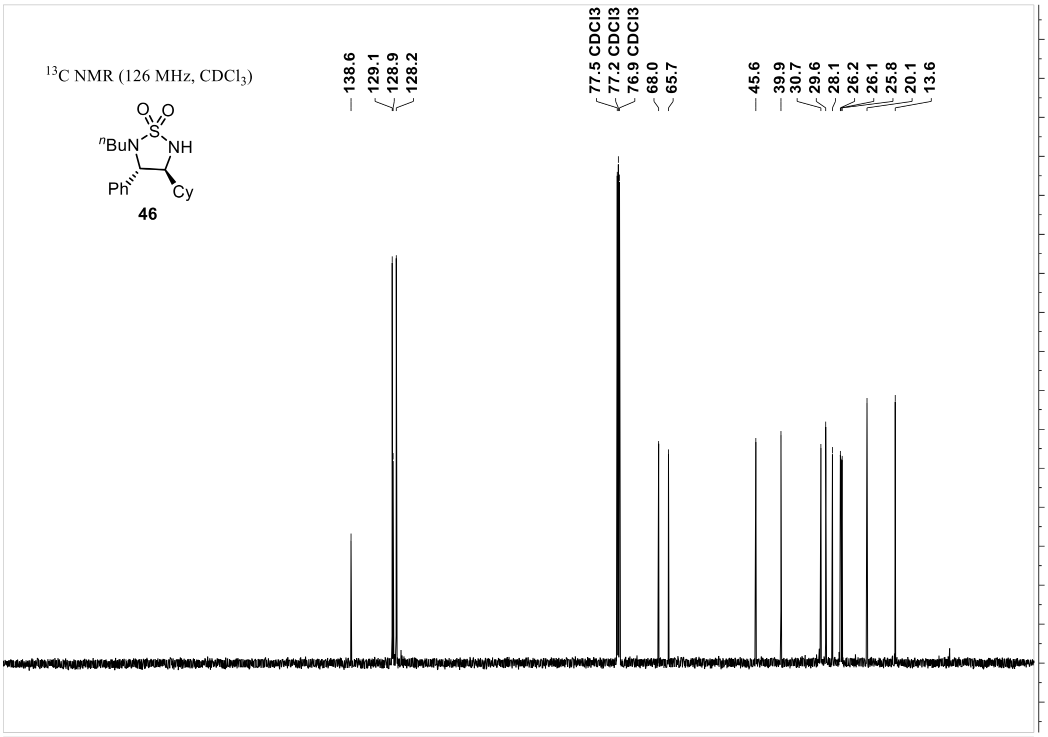

Supplementary Figure 82. ^{1}H NMR and ^{13}C NMR spectra of compound 46.

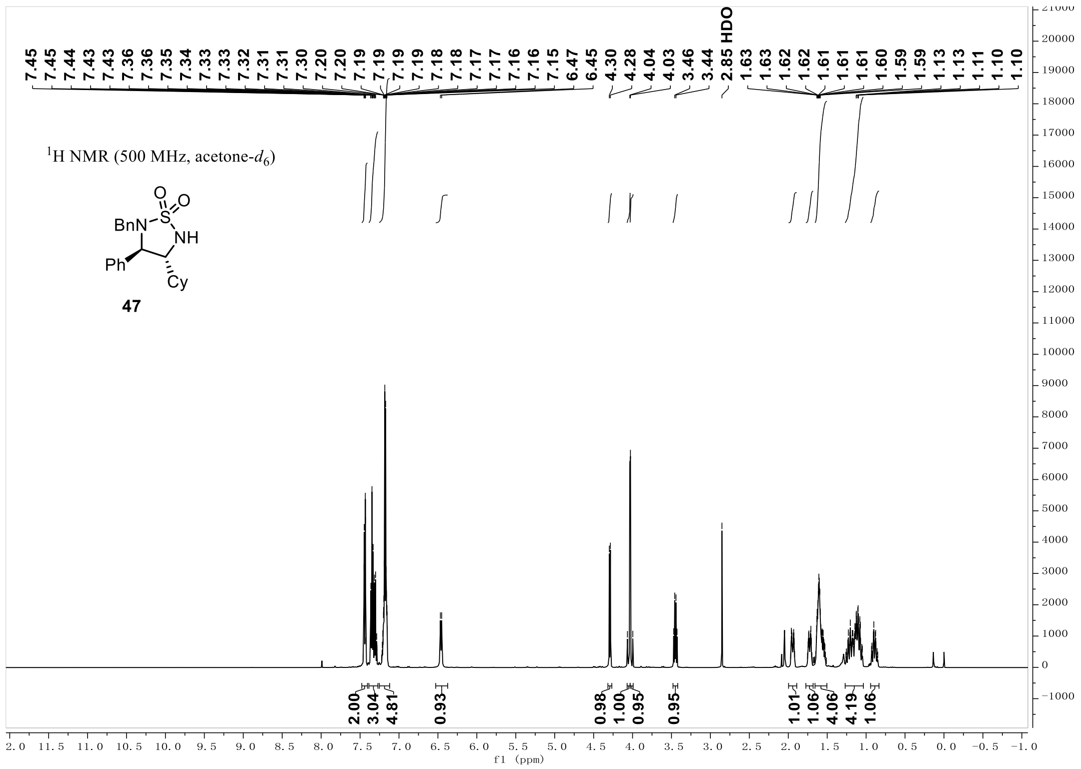

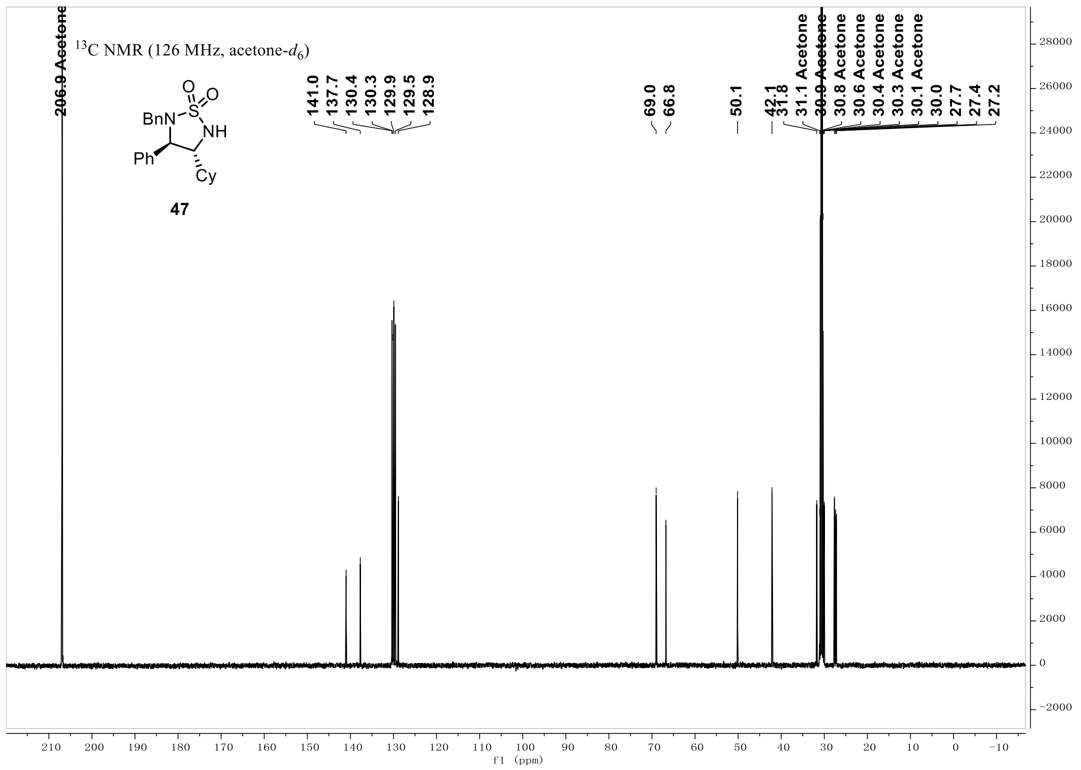

Supplementary Figure 83. ^{1}H NMR and ^{13}C NMR spectra of compound 47.

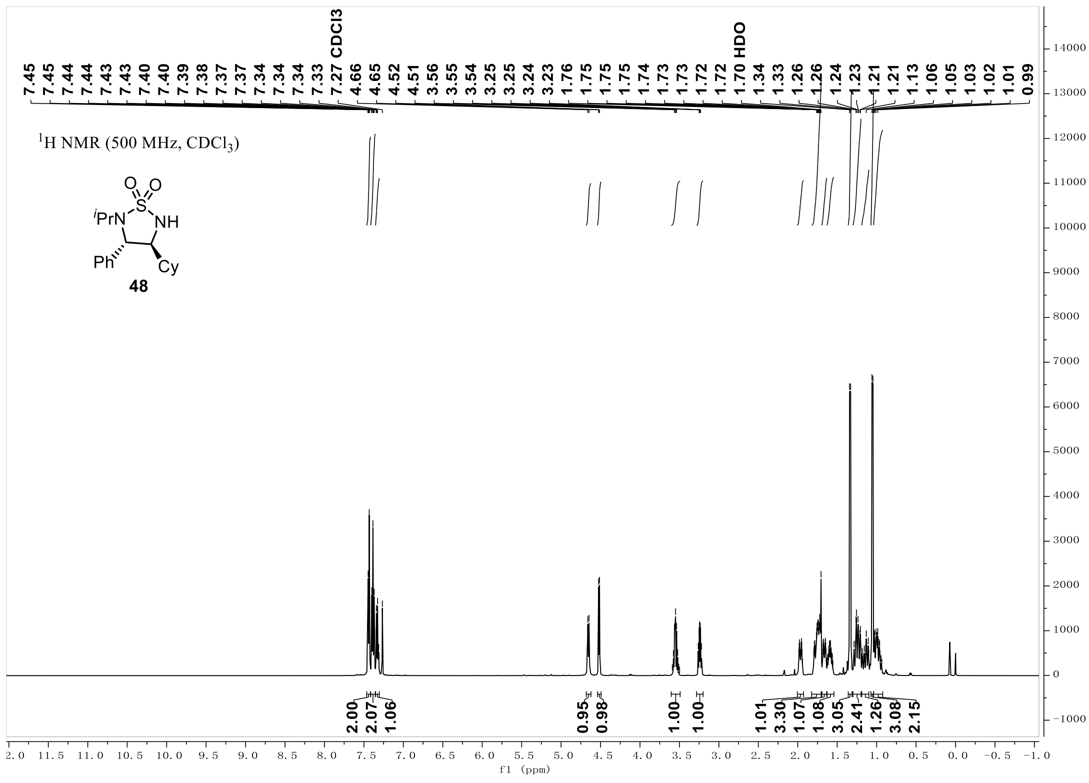

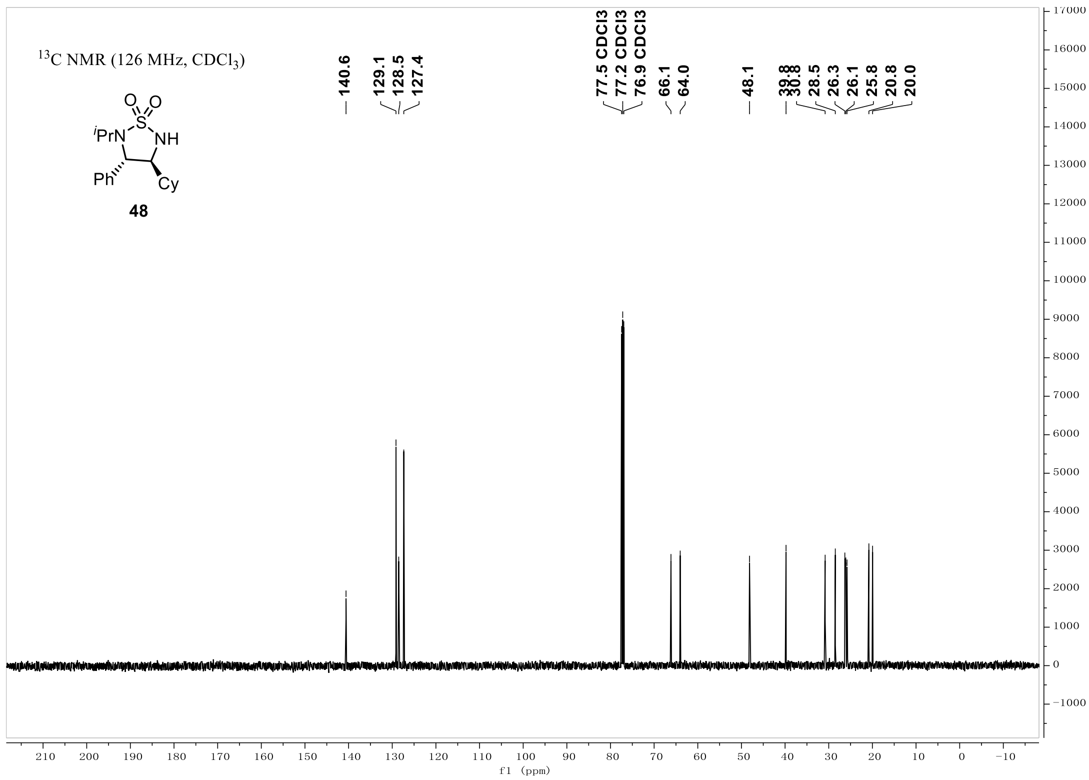

Supplementary Figure 84. ^{1}H NMR and ^{13}C NMR spectra of compound 48.

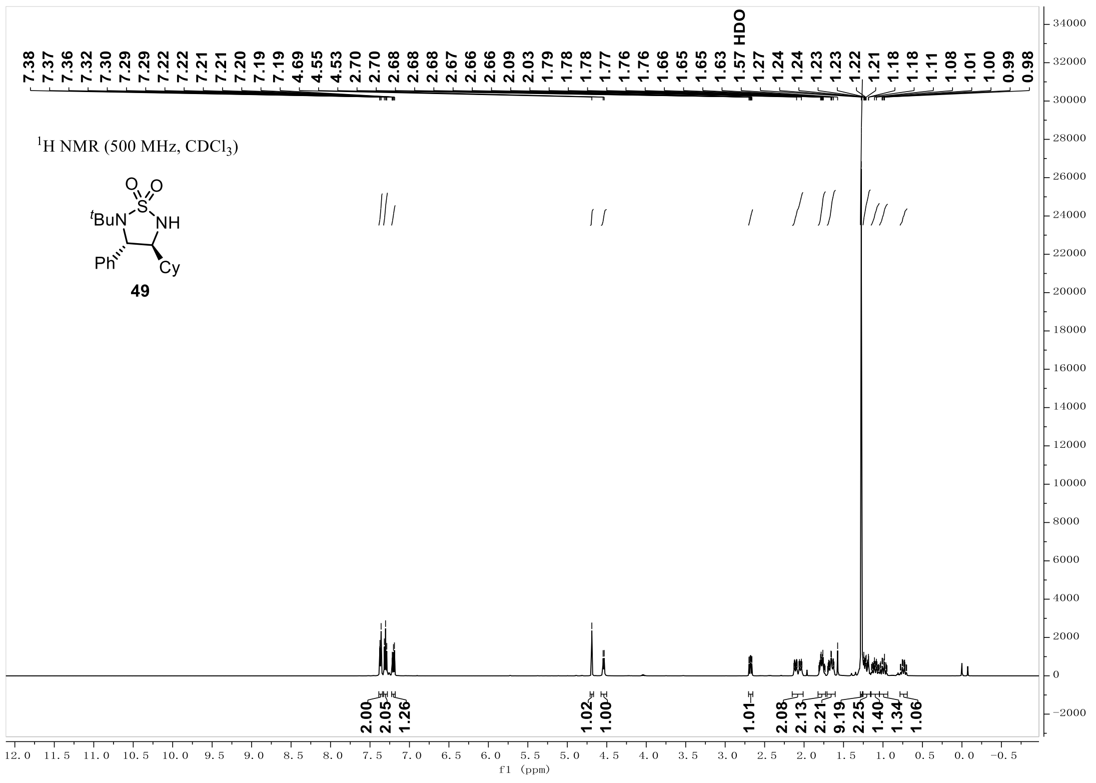

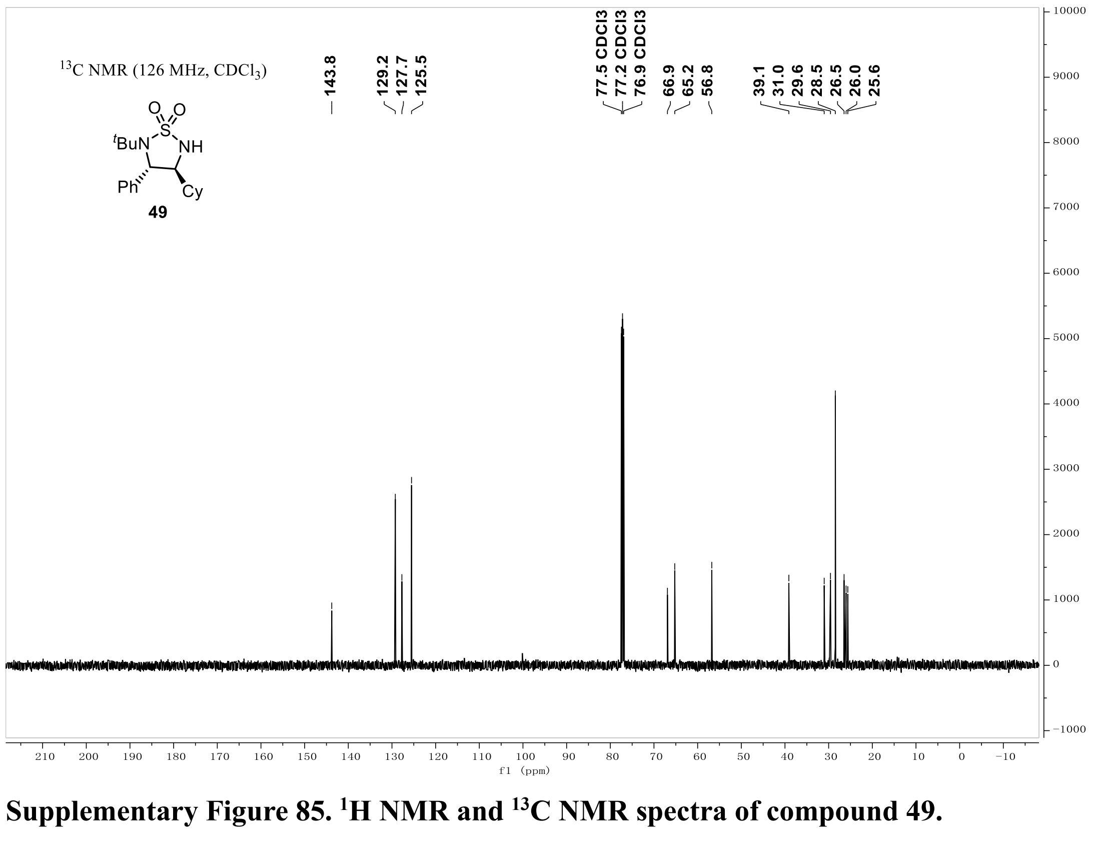

Supplementary Figure 85. ^{1}H NMR and ^{13}C NMR spectra of compound 49.

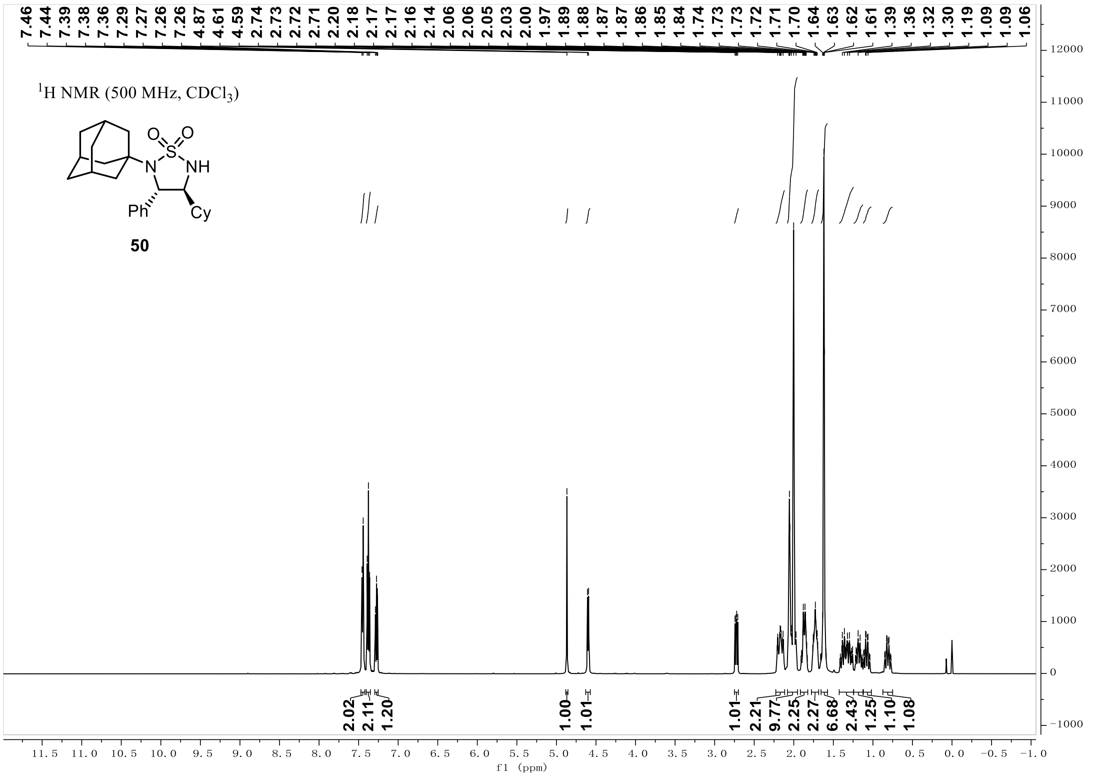

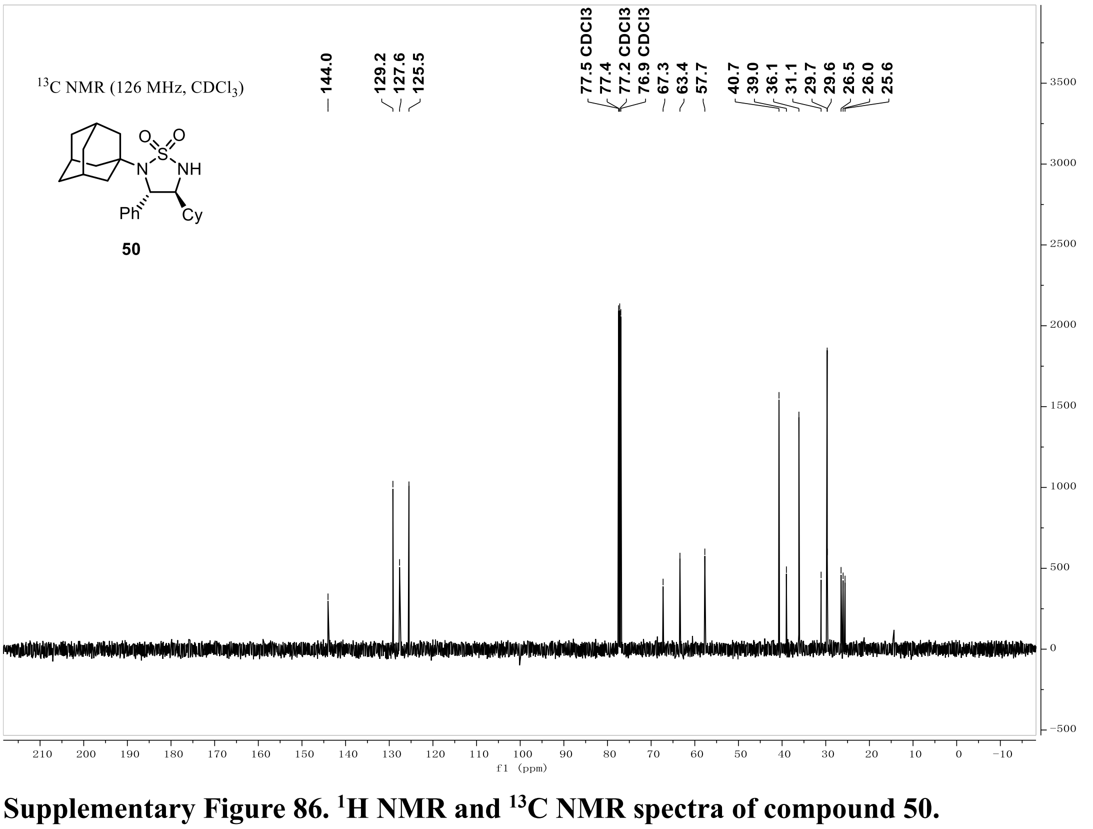

Supplementary Figure 86. ^{1}H NMR and ^{13}C NMR spectra of compound 50.

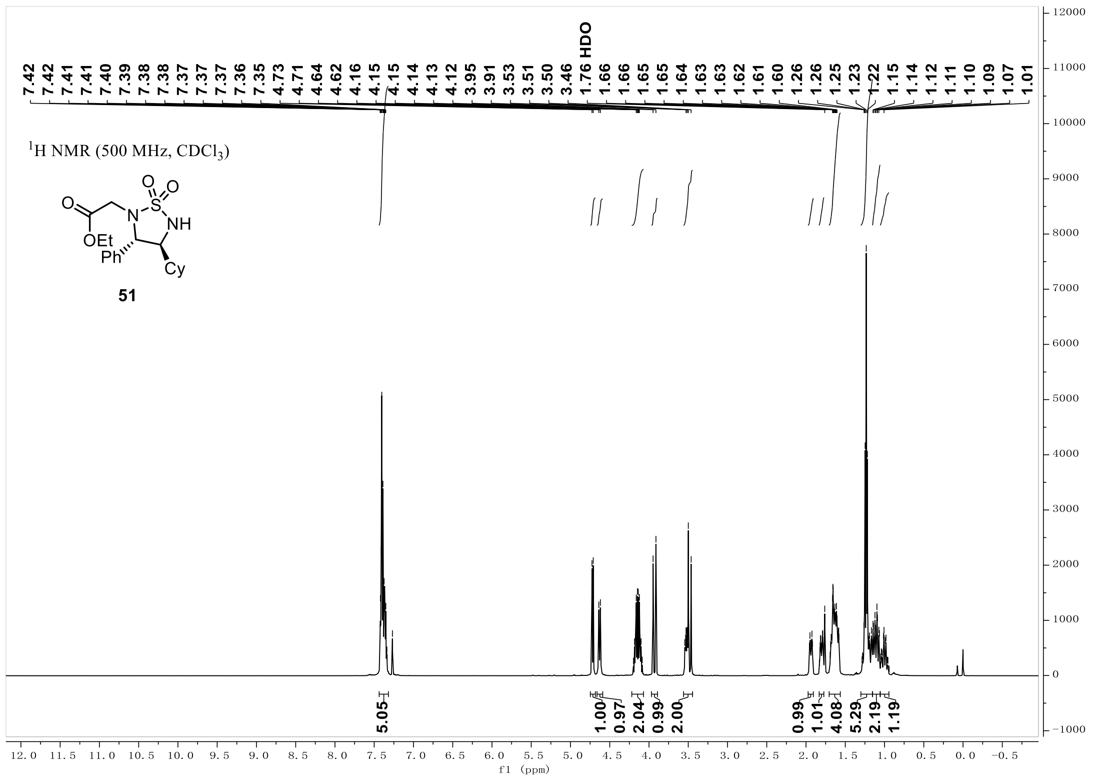

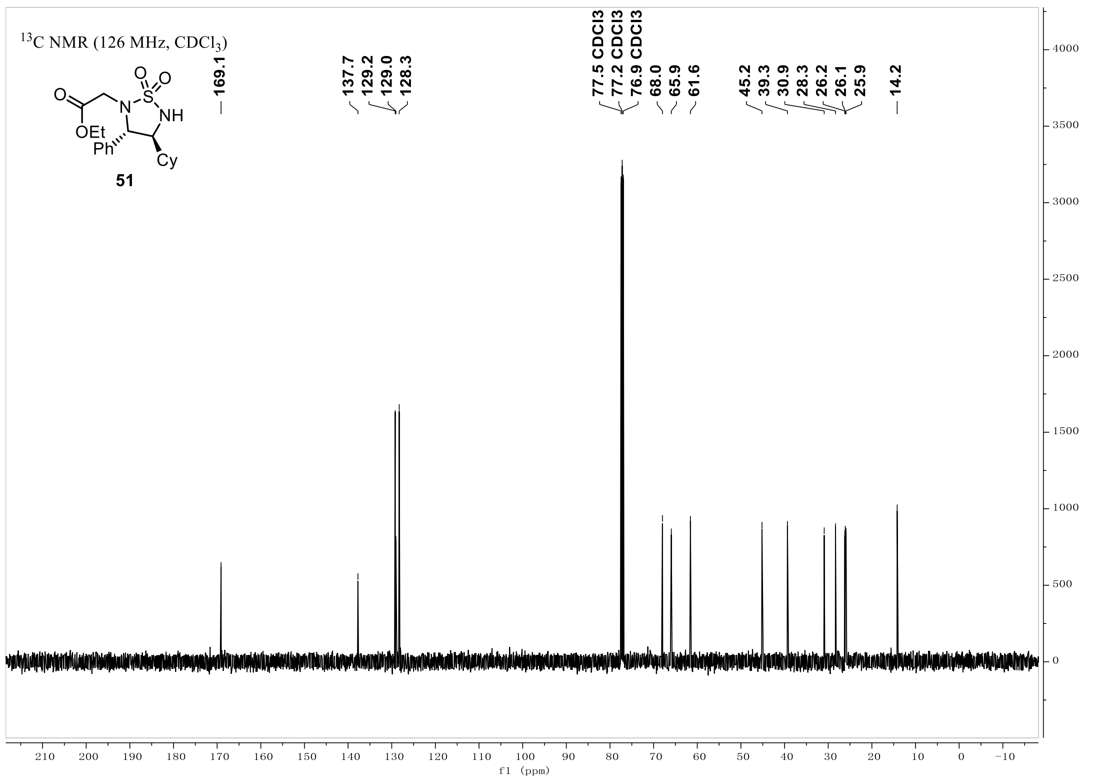

Supplementary Figure 87. ^{1}H NMR and ^{13}C NMR spectra of compound 51.

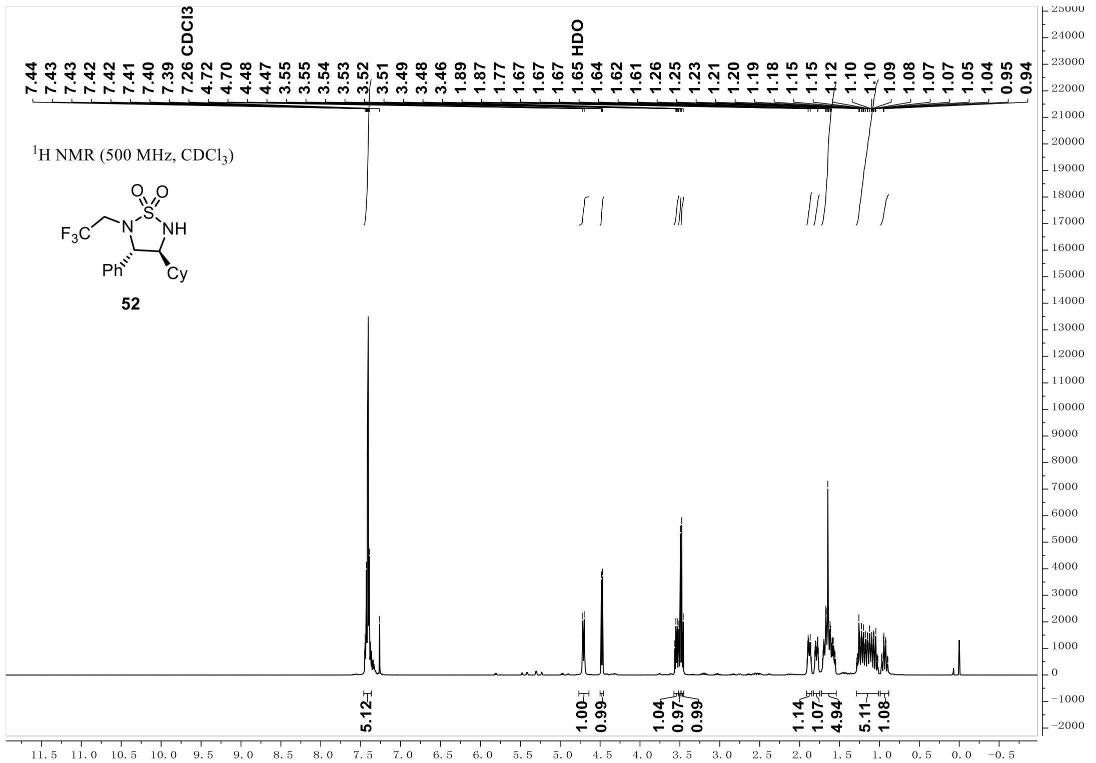

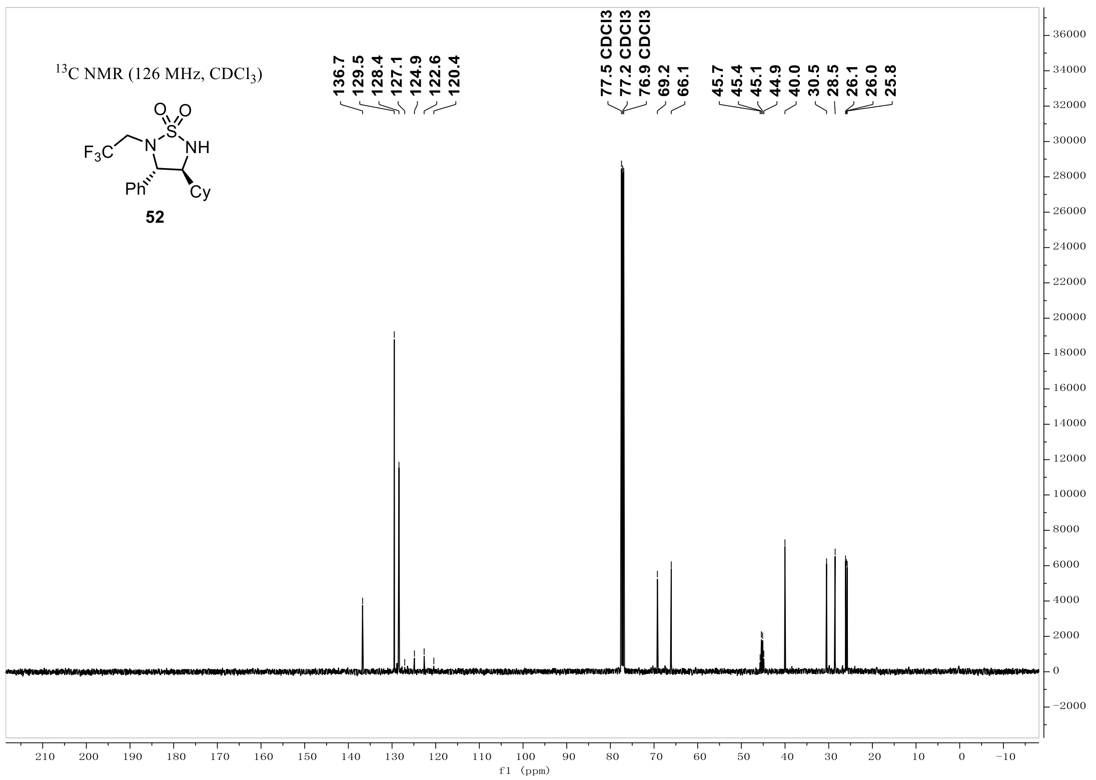

Supplementary Figure 88. ¹H NMR and ¹³C NMR spectra of compound 52.

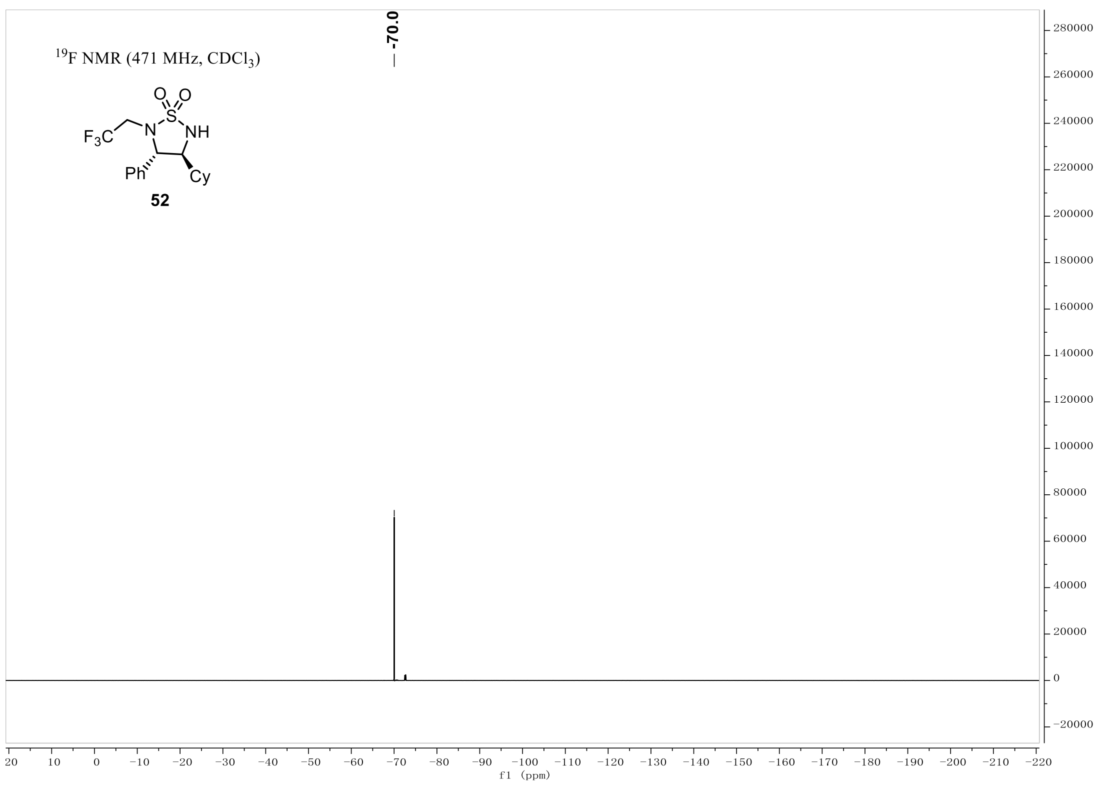

Supplementary Figure 89. ^{19}F NMR spectra of compound 52.

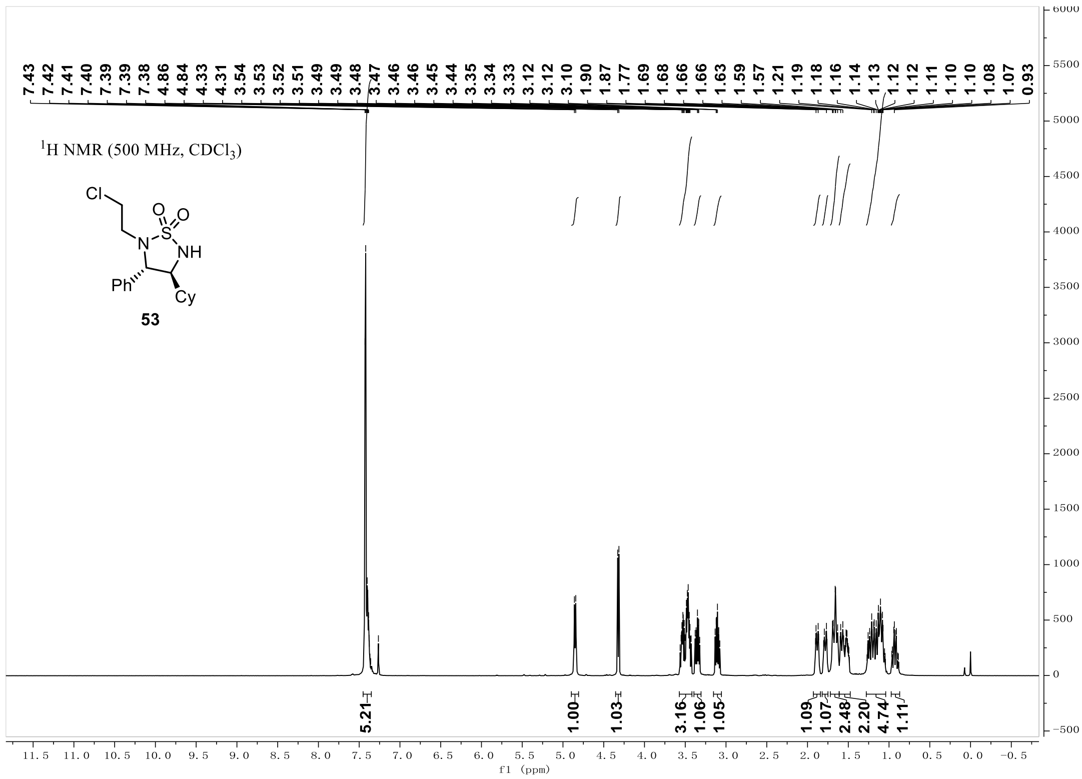

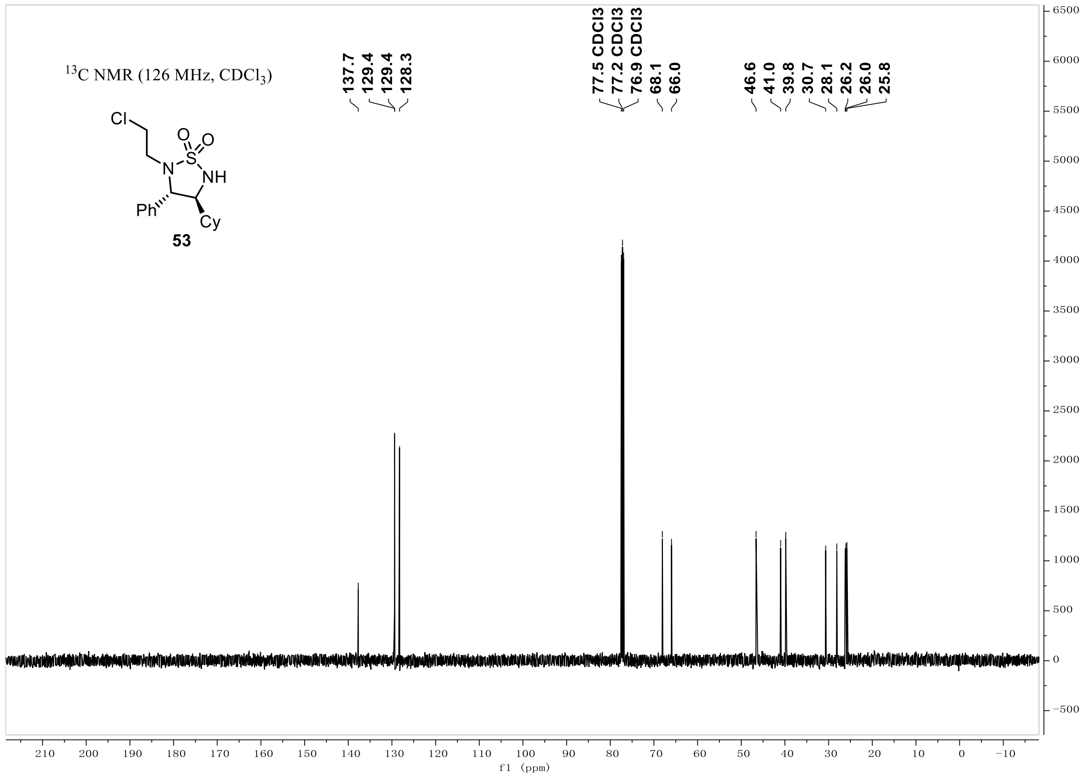

Supplementary Figure 90. ^{1}H NMR and ^{13}C NMR spectra of compound 53.

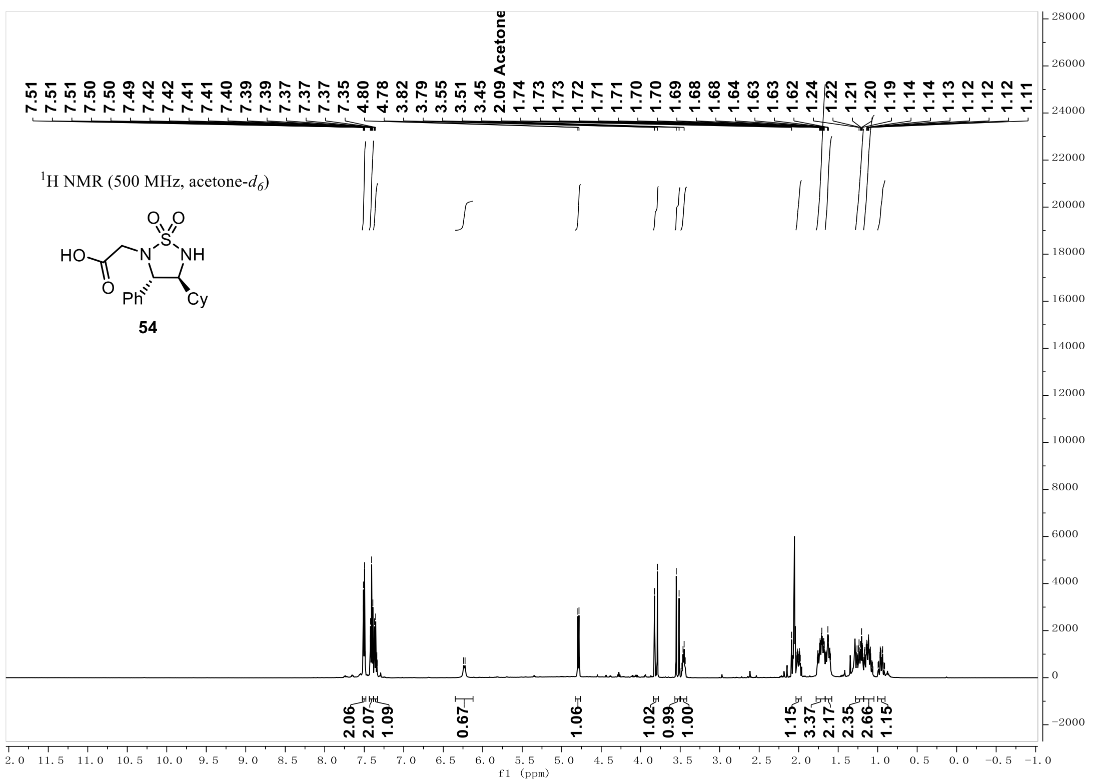

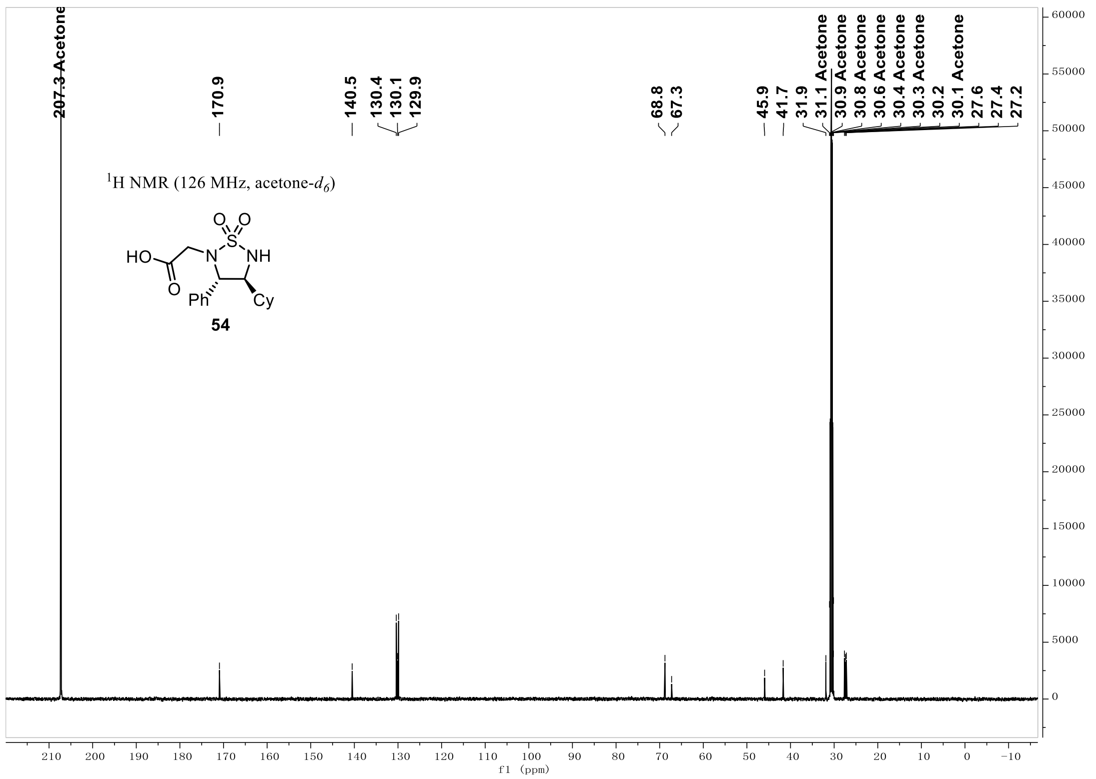

Supplementary Figure 91. ^{1}H NMR and ^{13}C NMR spectra of compound 54.

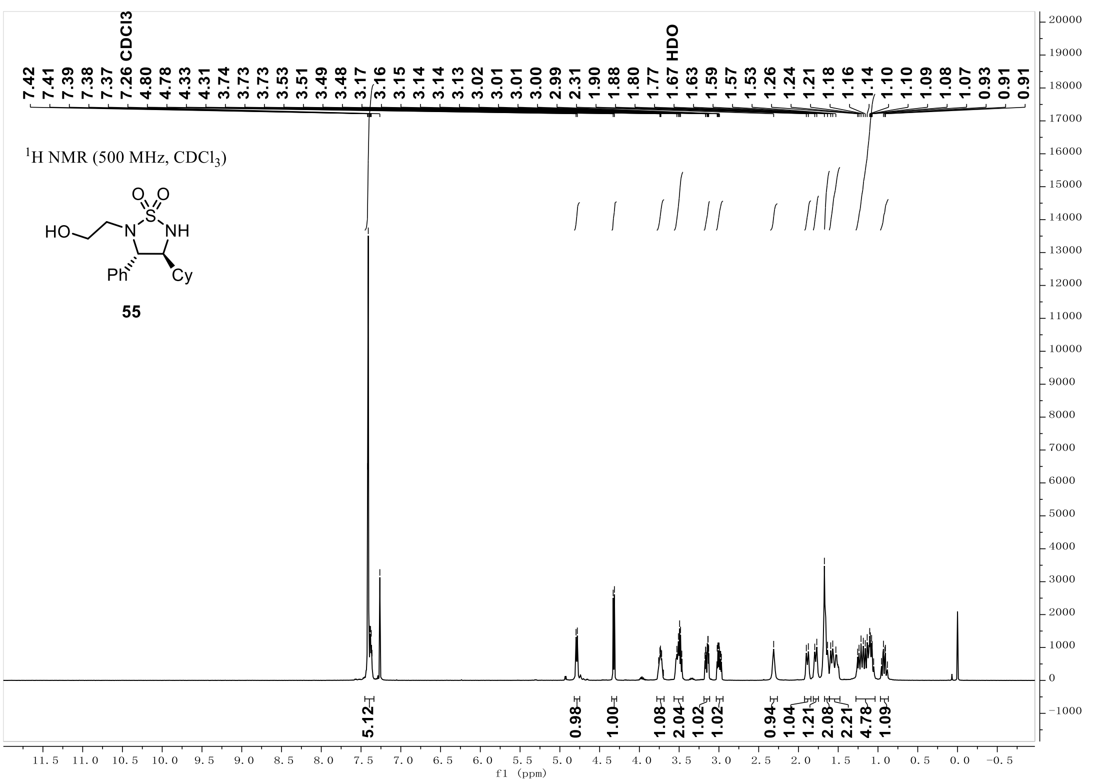

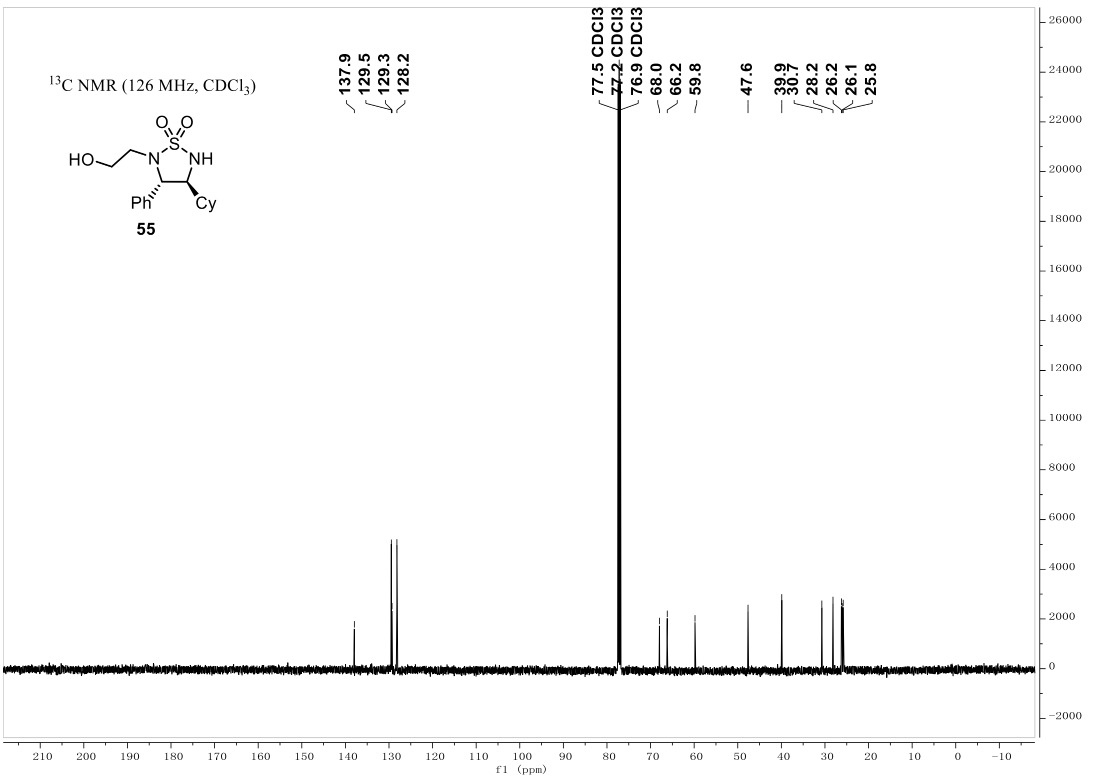

Supplementary Figure 92. ^{1}H NMR and ^{13}C NMR spectra of compound 55.

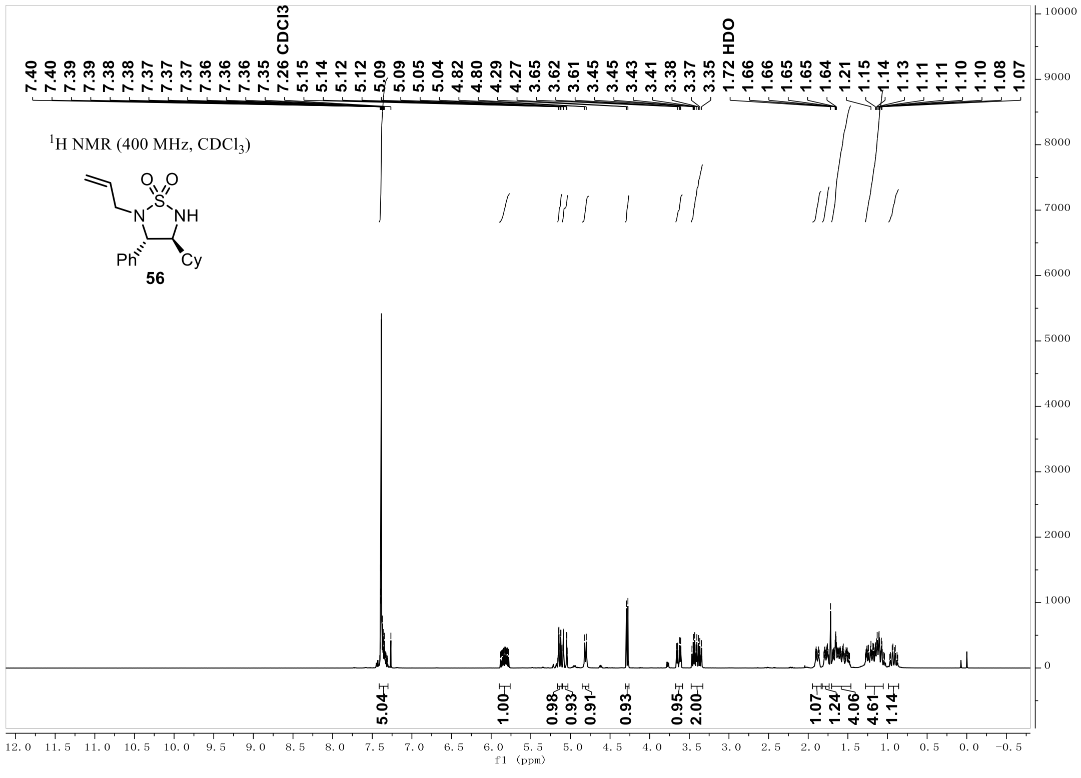

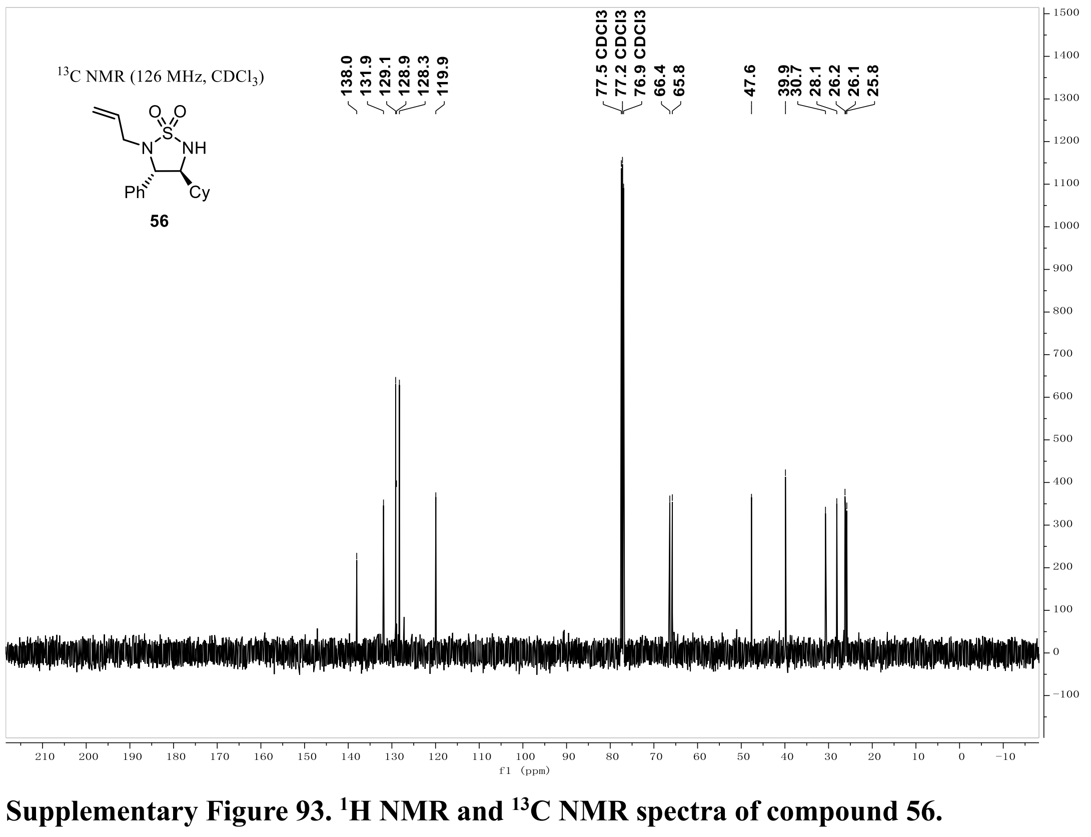

Supplementary Figure 93. ^{1}H NMR and ^{13}C NMR spectra of compound 56.

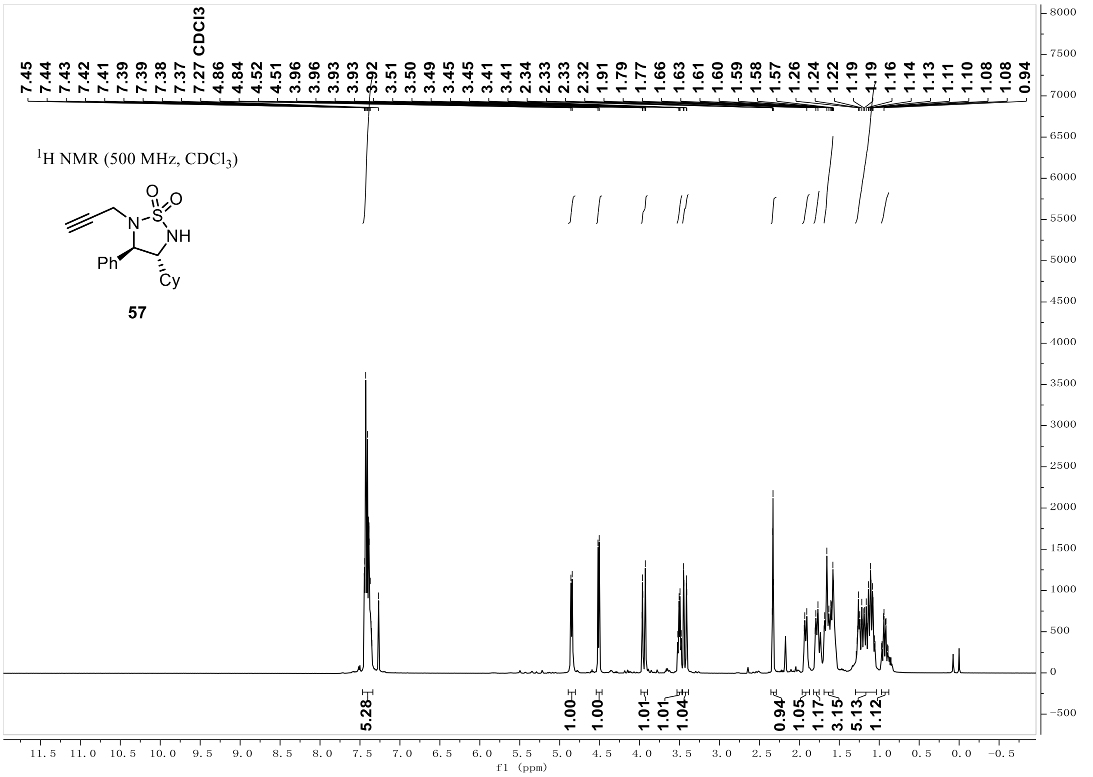

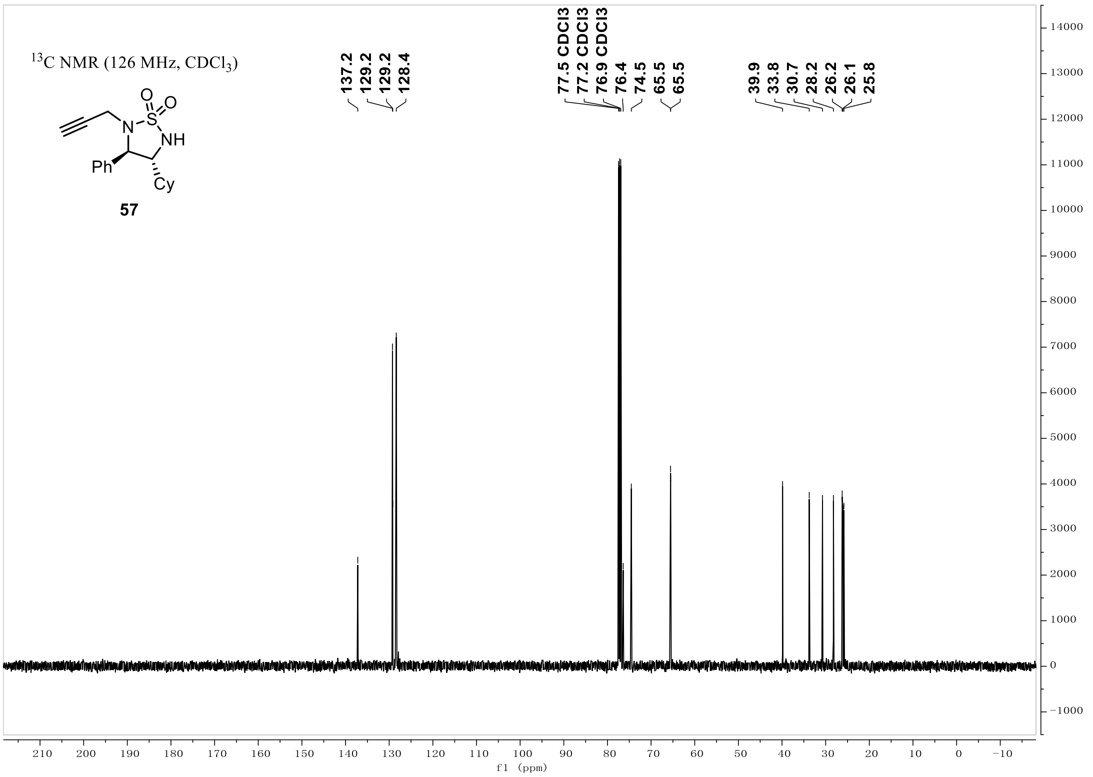

Supplementary Figure 94. ^{1}H NMR and ^{13}C NMR spectra of compound 57.

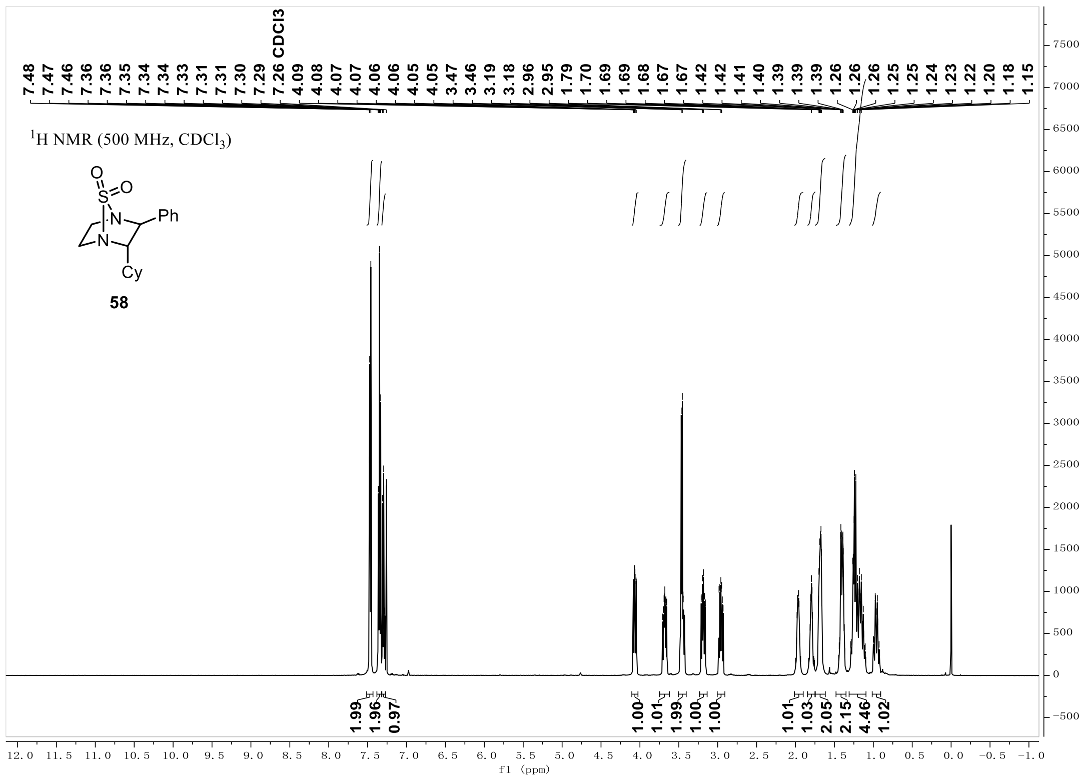

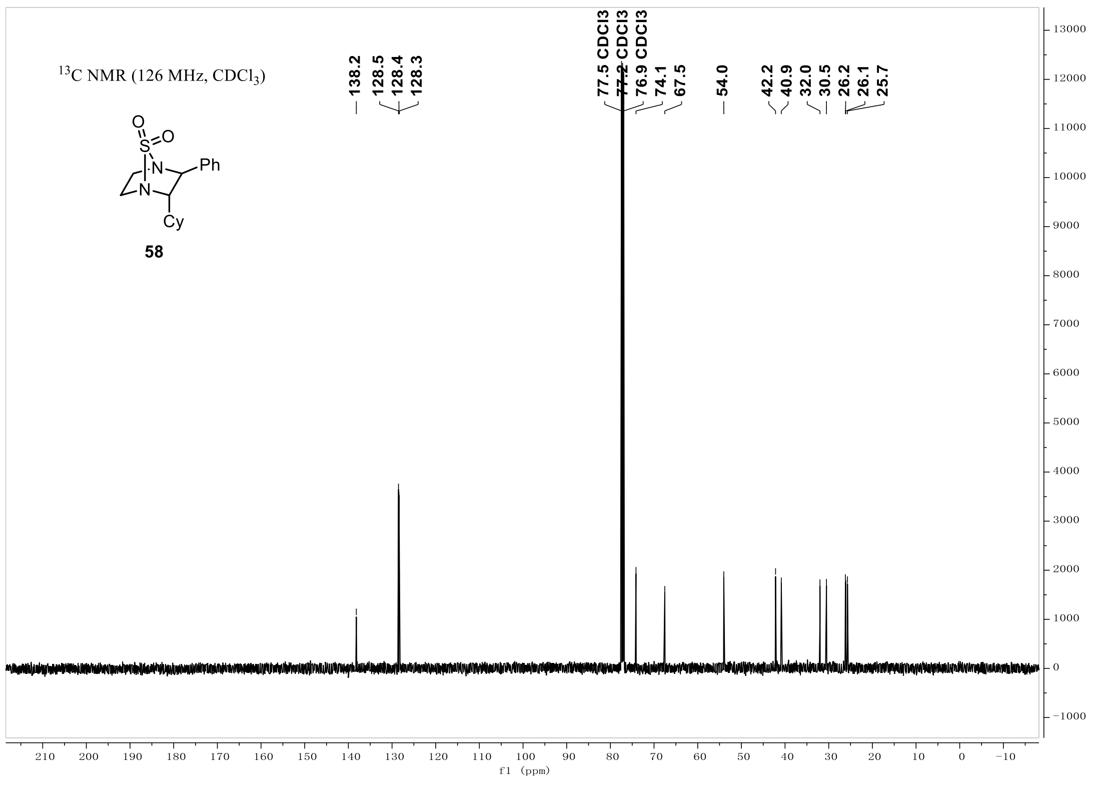

Supplementary Figure 95. ^{1}H NMR and ^{13}C NMR spectra of compound 58.

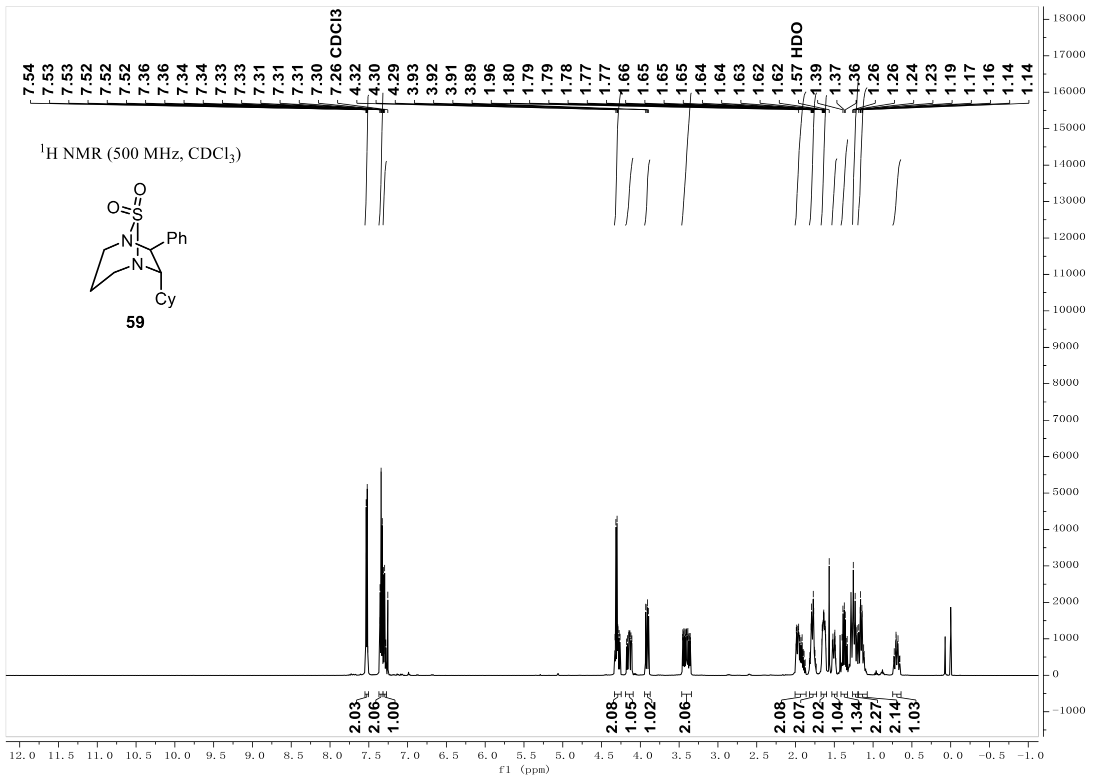

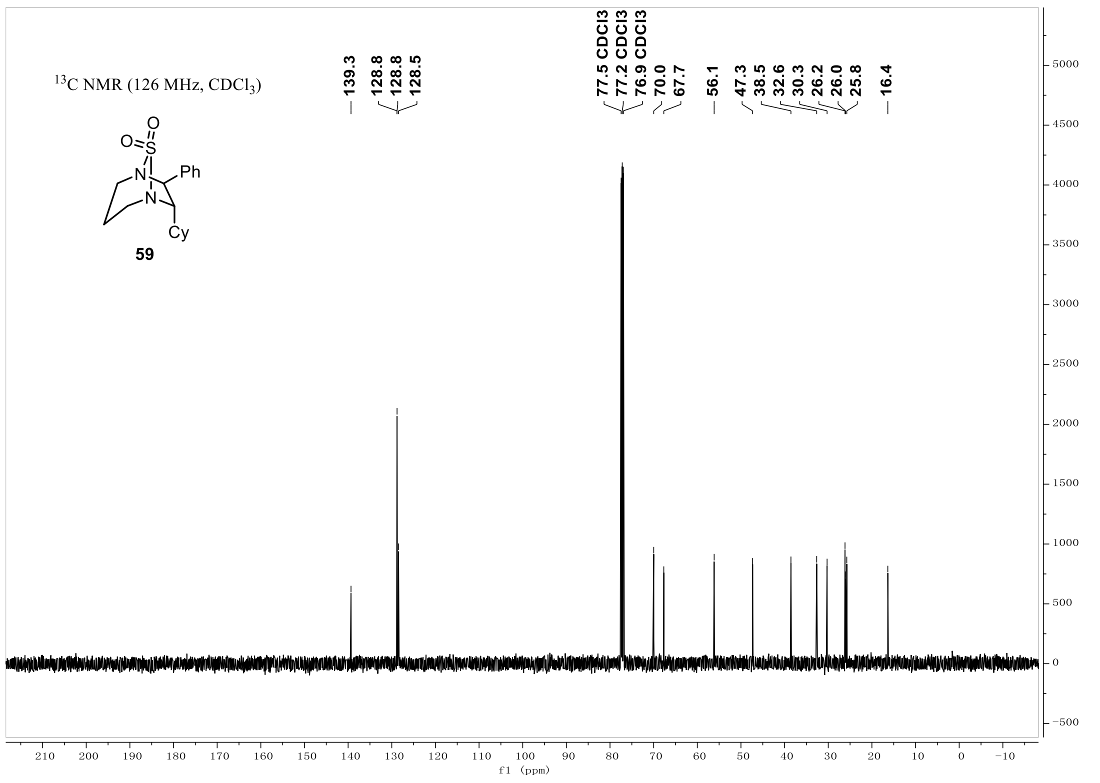

Supplementary Figure 96. ^{1}H NMR and ^{13}C NMR spectra of compound 59.

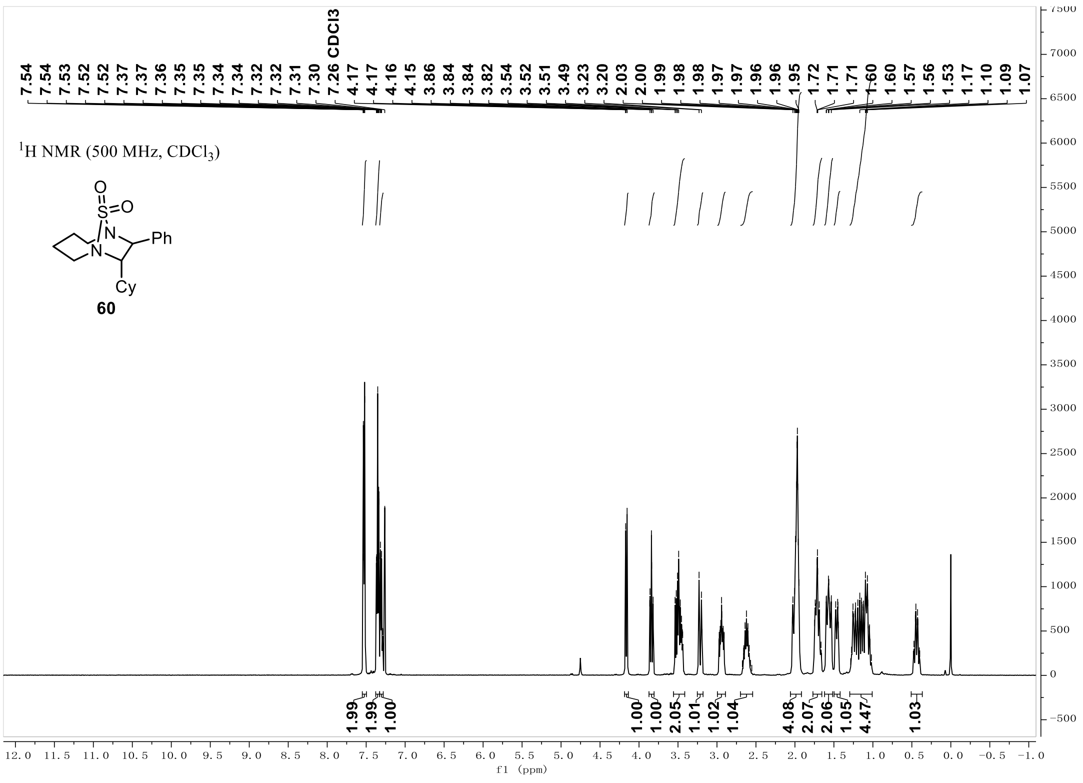

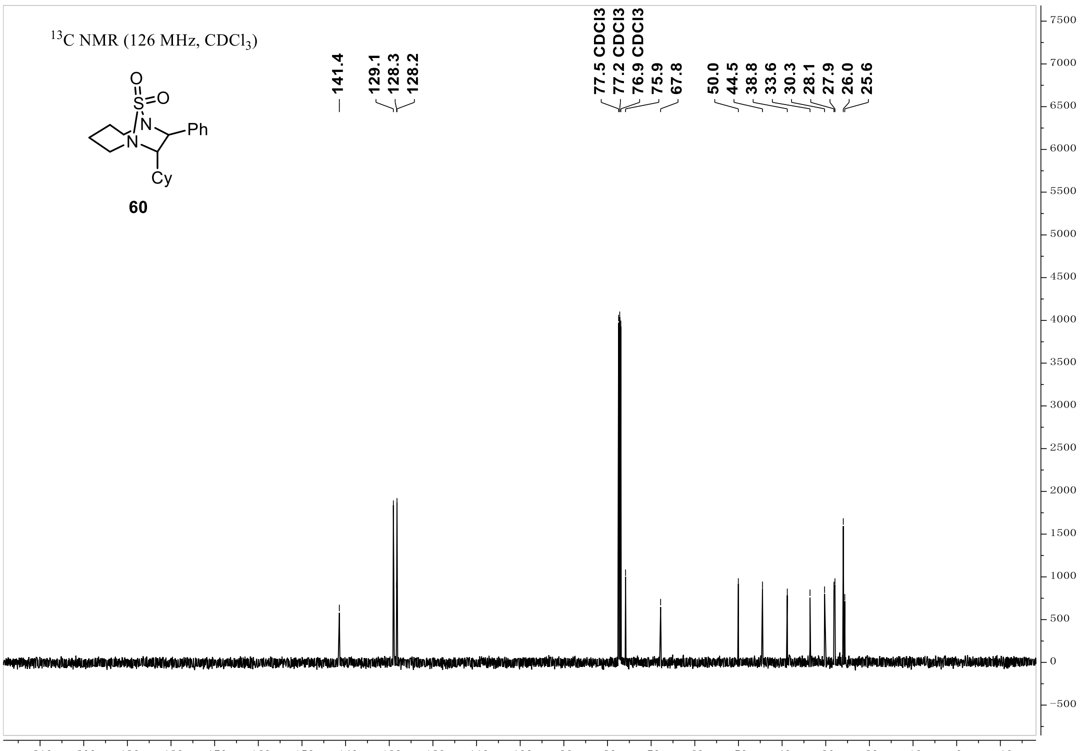

Supplementary Figure 97. ^{1}H NMR and ^{13}C NMR spectra of compound 60.

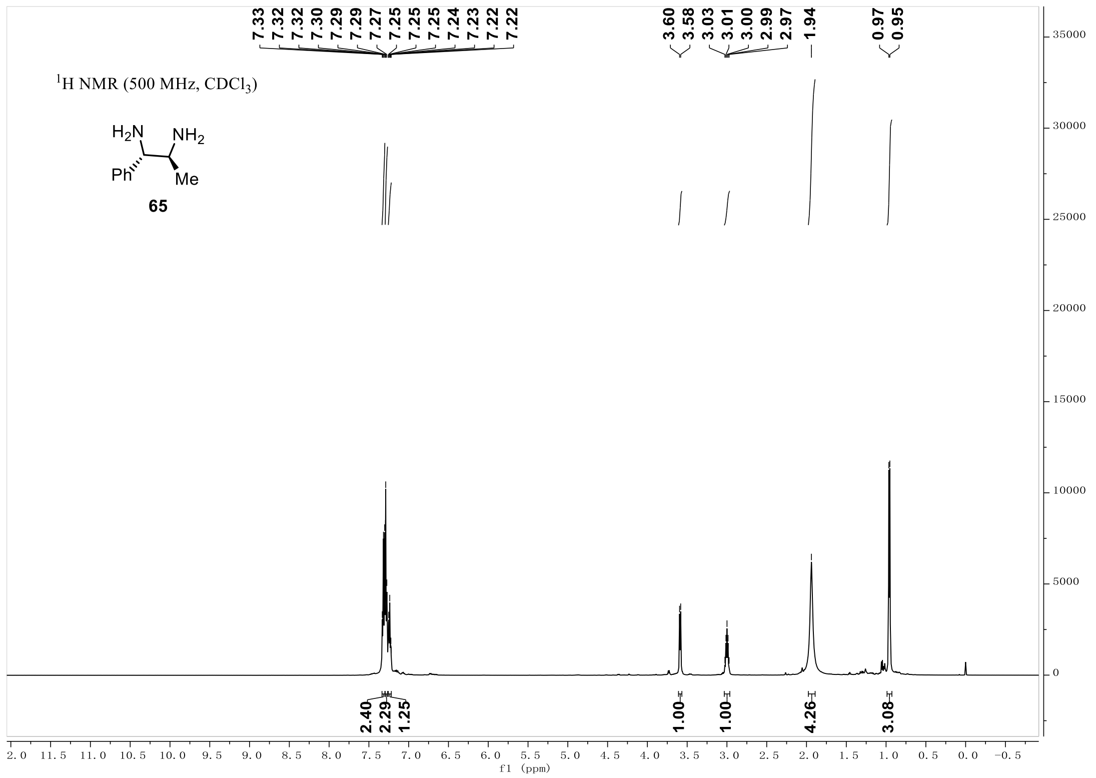

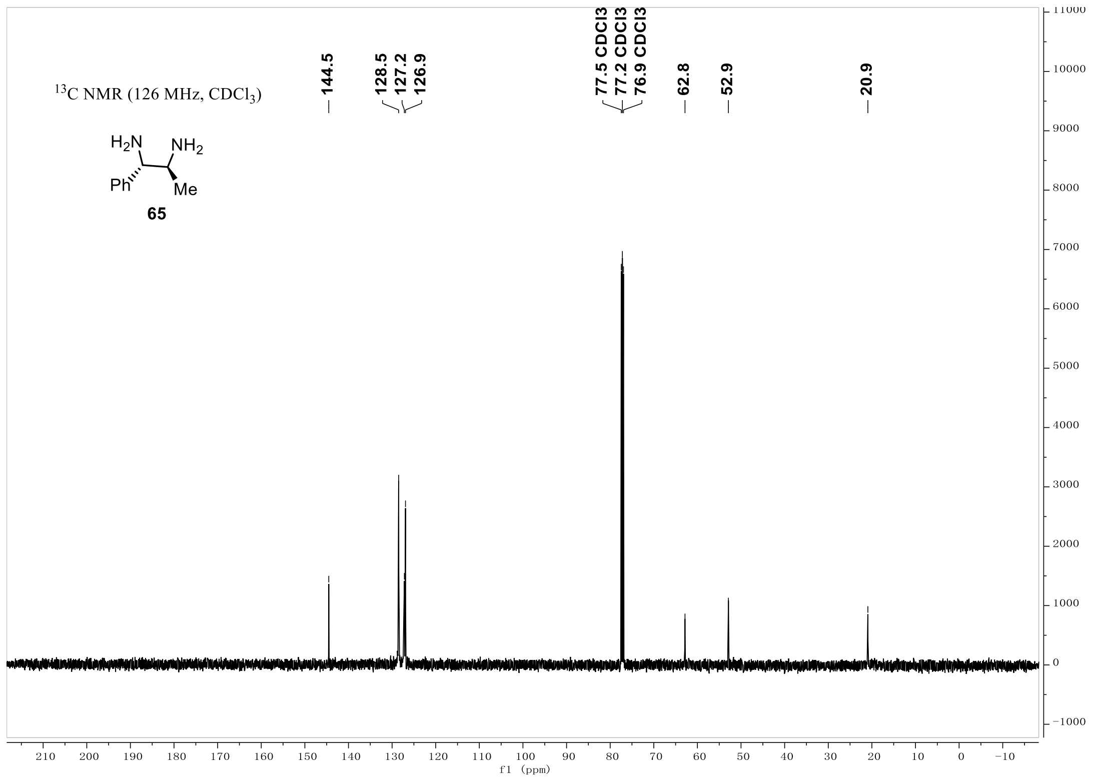

Supplementary Figure 98. ^{1}H NMR and ^{13}C NMR spectra of compound 65.

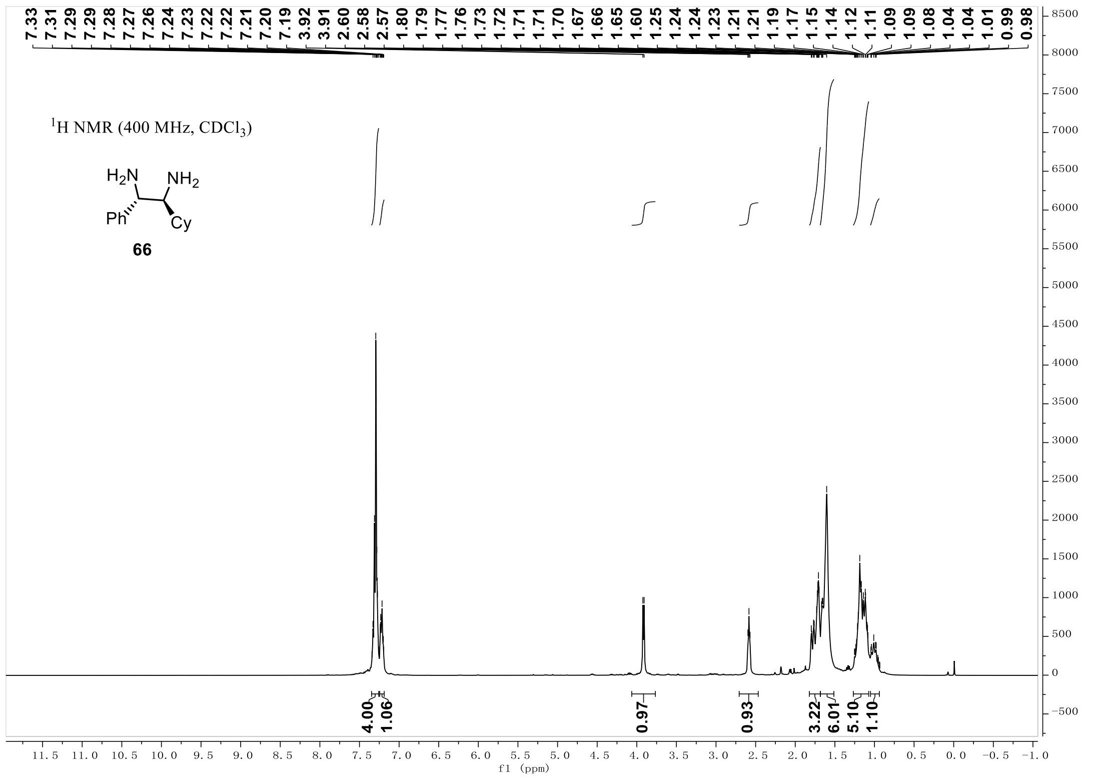

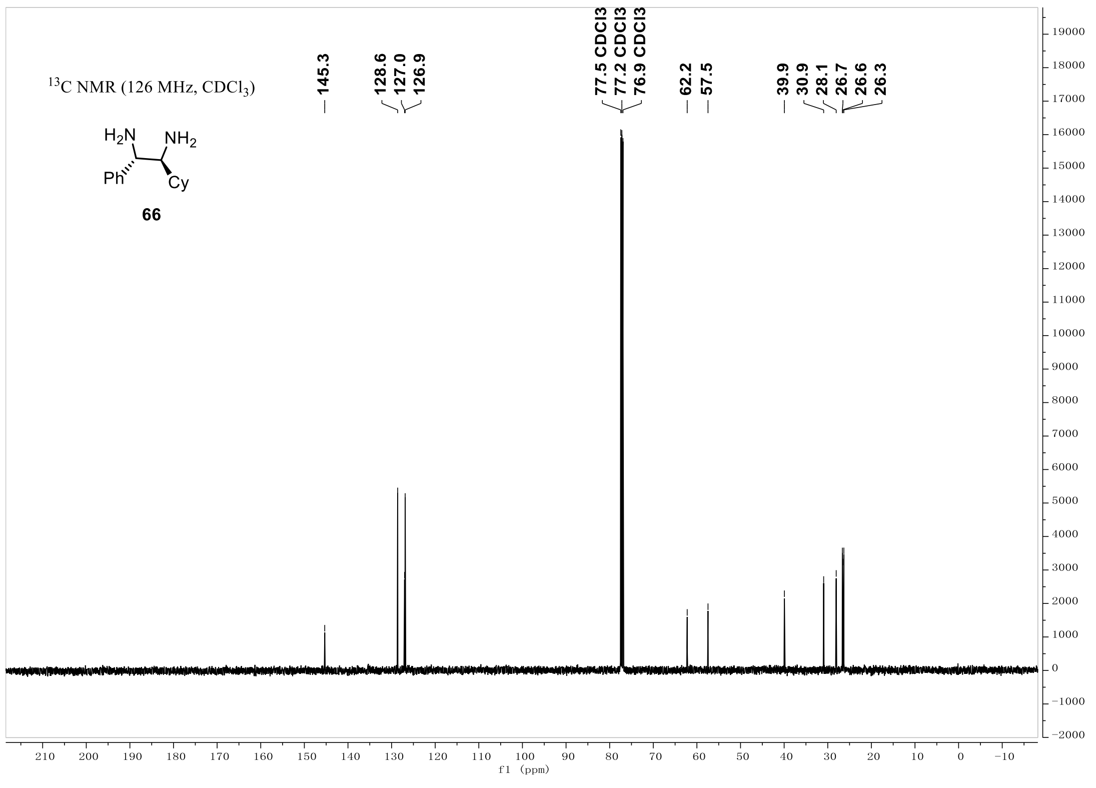

Supplementary Figure 99. ^{1}H NMR and ^{13}C NMR spectra of compound 66.

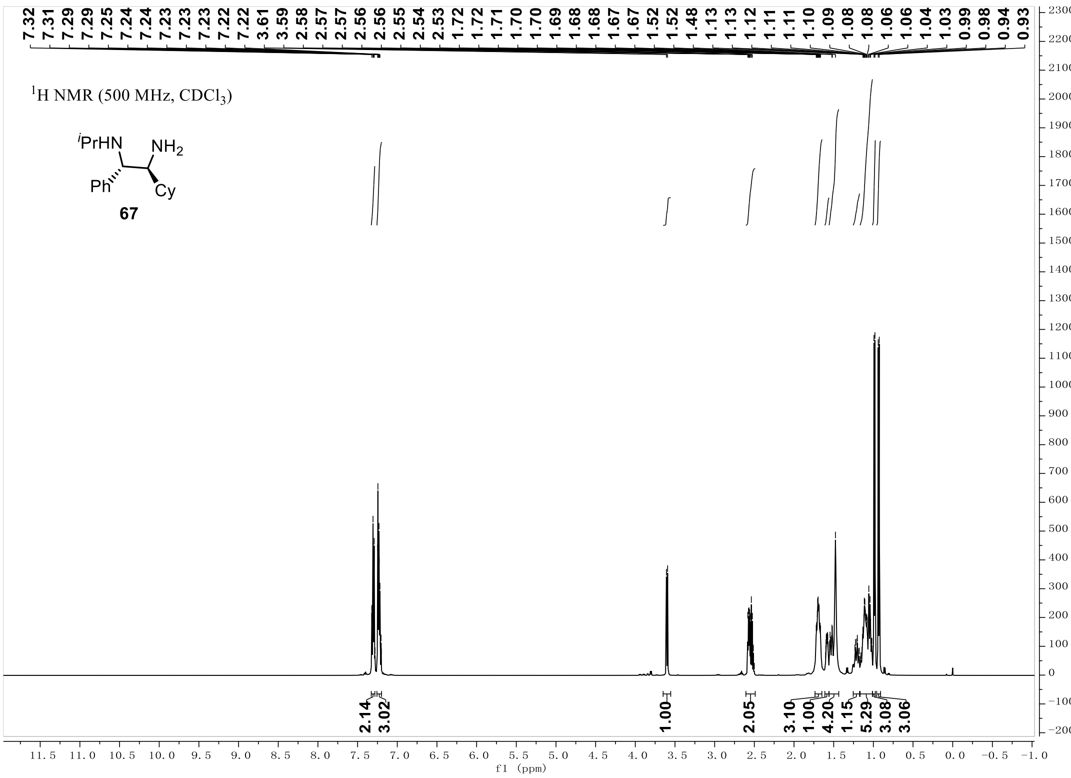

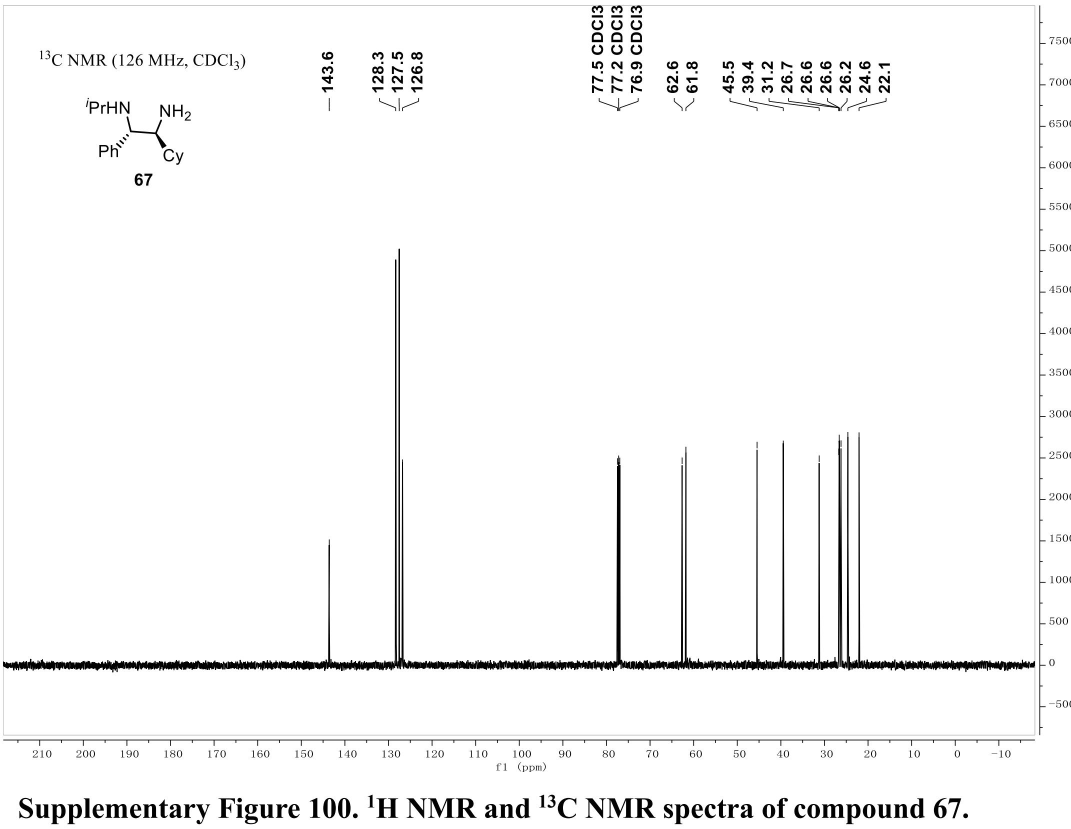

Supplementary Figure 100. ^{1}H NMR and ^{13}C NMR spectra of compound 67.

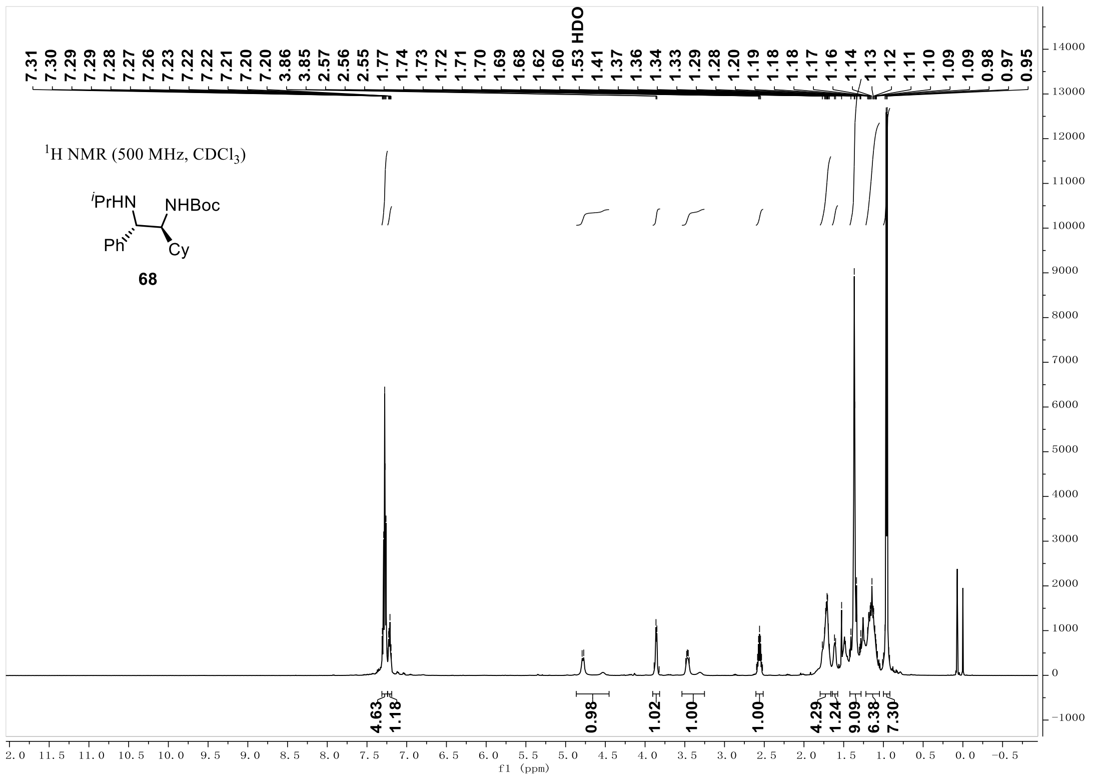

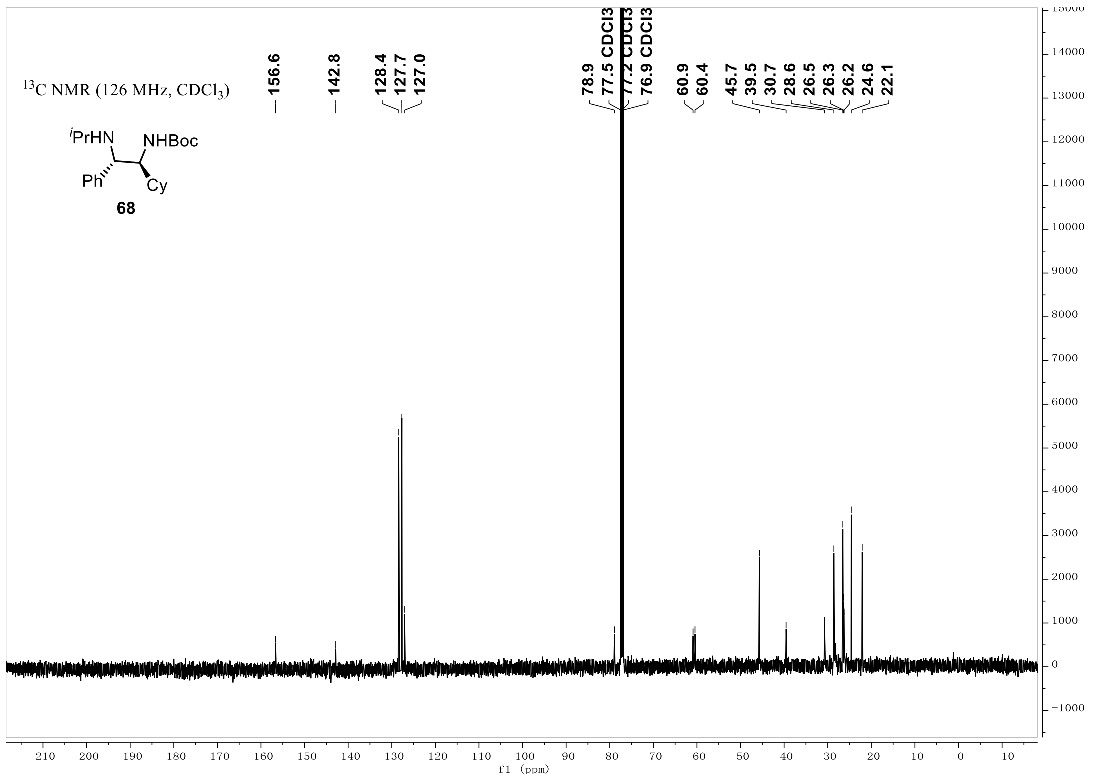

Supplementary Figure 101. ^{1}H NMR and ^{13}C NMR spectra of compound 68.

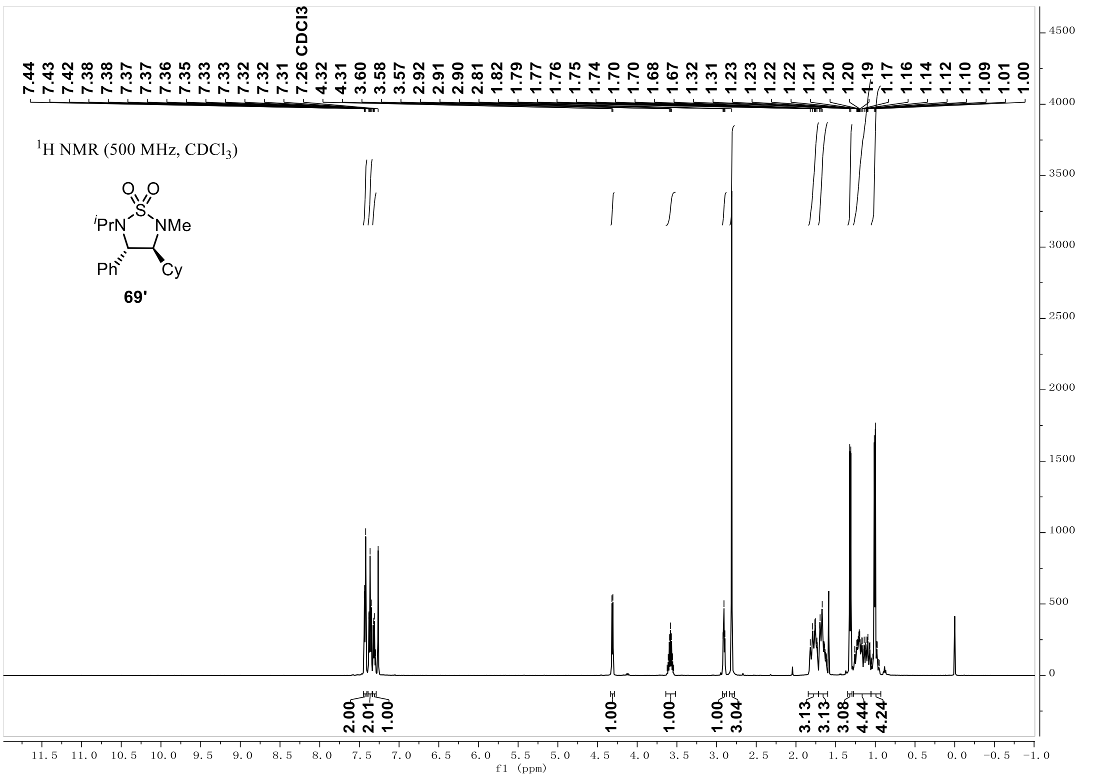

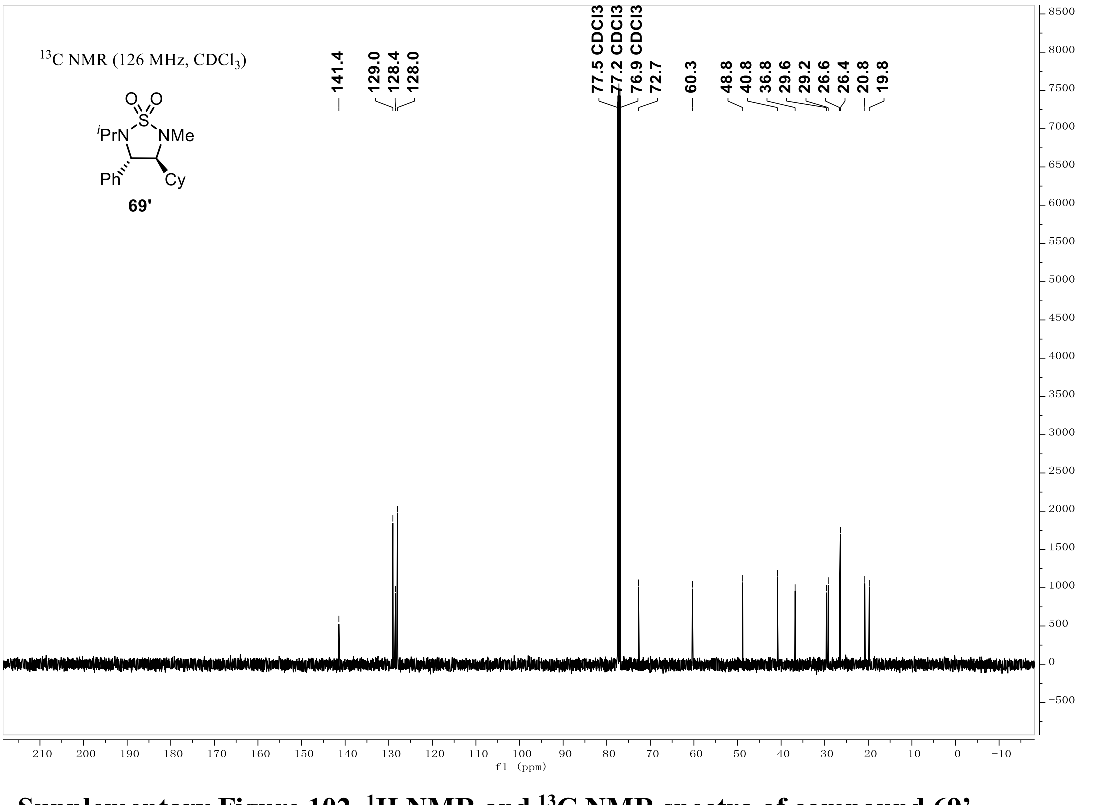

Supplementary Figure 102. ^{1}H NMR and ^{13}C NMR spectra of compound 69'.

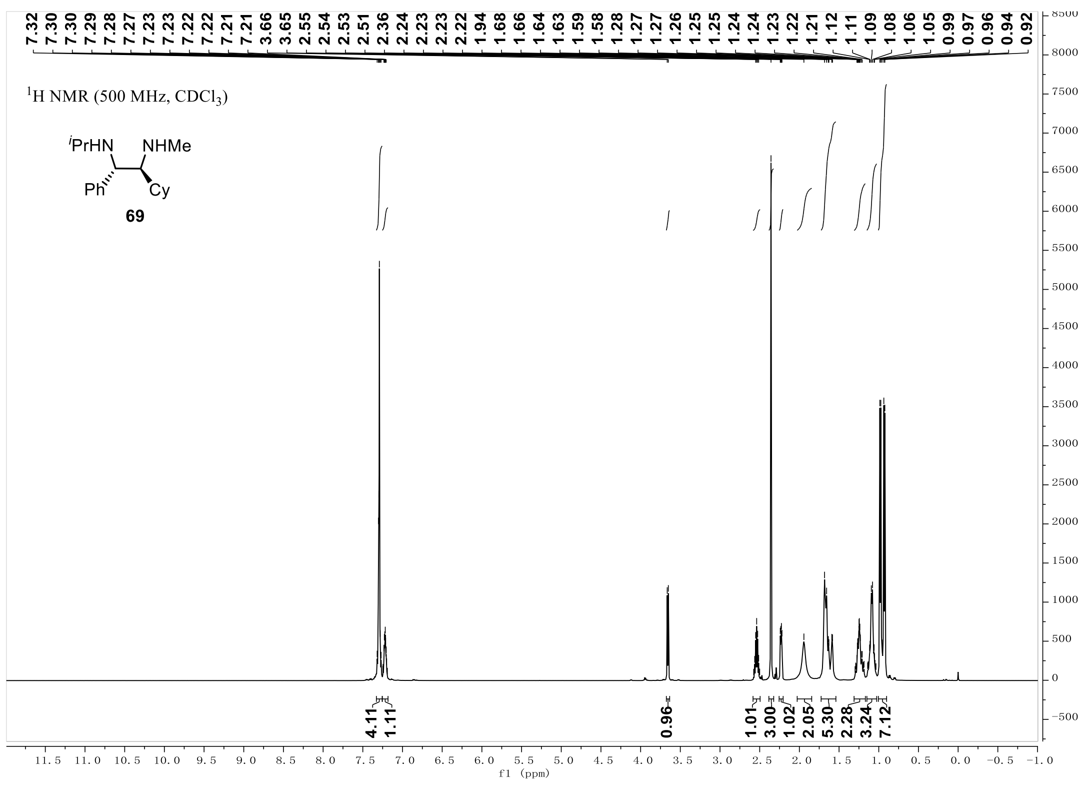

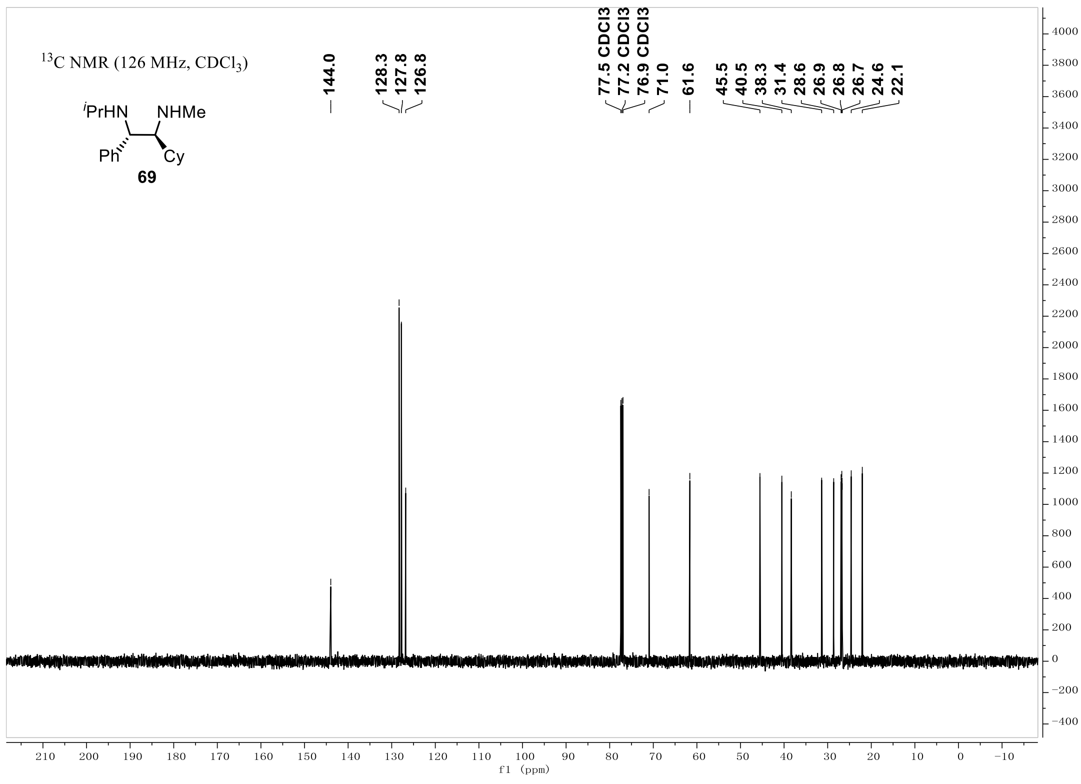

Supplementary Figure 103. ^{1}H NMR and ^{13}C NMR spectra of compound 69.

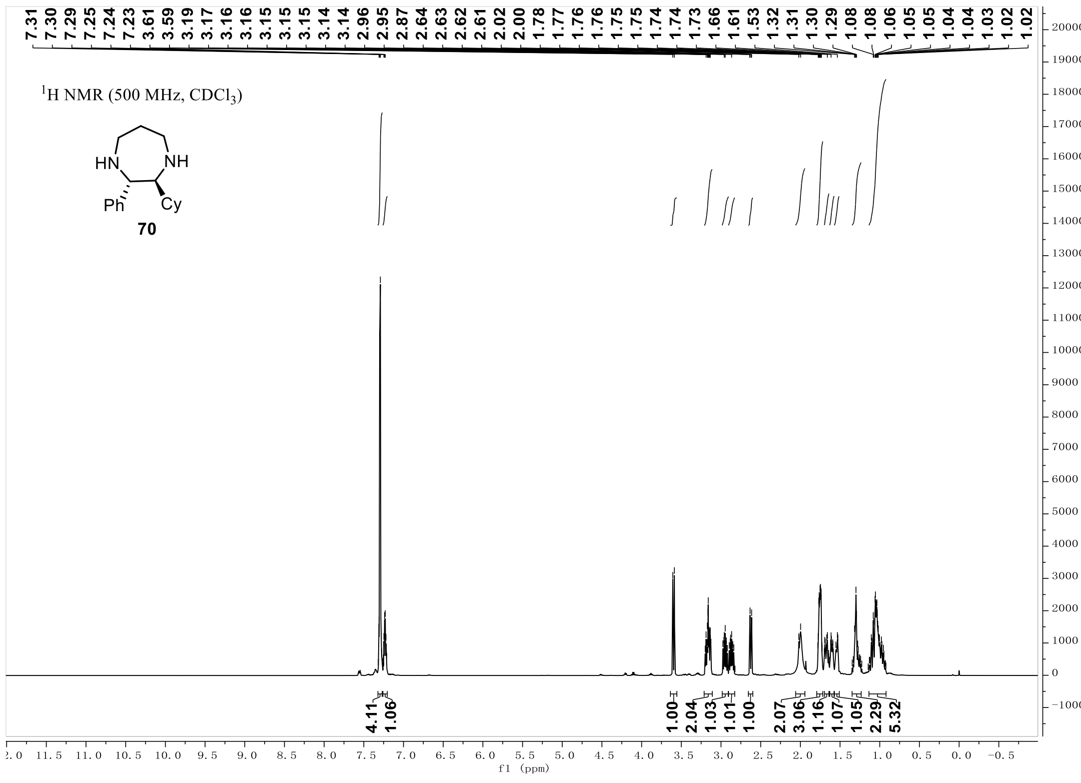

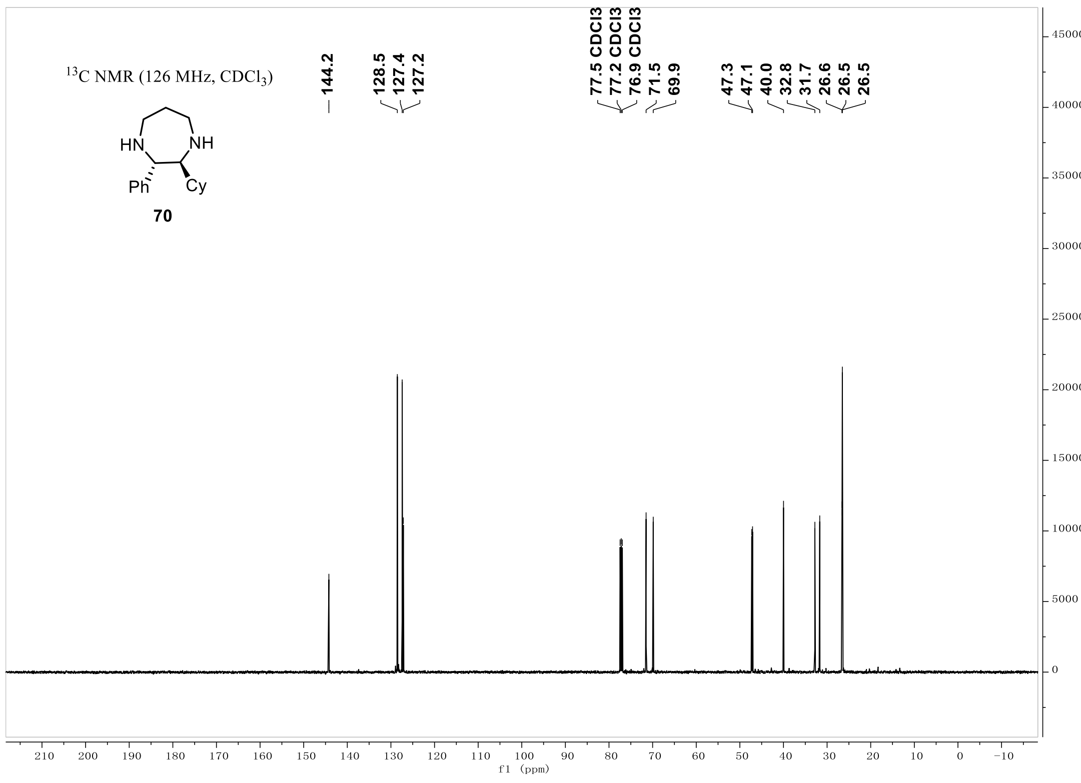

Supplementary Figure 104. ^{1}H NMR and ^{13}C NMR spectra of compound 70.

Supplementary References

1. Yang, H.-T., Lu, X.-W., Xing, M.-L., Sun, X.-Q. & Miao, C.-B. Hypervalent iodine reagent mediated diamination of [60]fullerene with sulfamides or phosphoryl diamides. *Org. Lett.* **16**, 5882−5885 (2014).
2. Cai, C.-Y. & Xu, H.-C. Dehydrogenative reagent-free annulation of alkenes with diols for the synthesis of saturated O-heterocycles. *Nat. Commun*. **9**, 3551−3558 (2018).
3. Gust, R. et al. The stereoselectivity of antitumor active [1,2-diamino-1-phenylpropane]dichloroplatinum(II) complexes. *Inorganica. Chimica. Acta.* **264**, 145−160 (1997).
4. Schüttler, C., Li-Böhmer, Z., Harms, K. & von Zezschwitz, P. Enantioselective synthesis of 3,4-disubstituted *cis*- and *trans*-1,2,5-thiadiazolidine-1,1-dioxides as precursors for chiral 1,2-diamines. *Org. Lett*. **15**, 800−803 (2013).
5. Cornwall, R. G., Zhao, B. & Shi, Y. Catalytic asymmetric synthesis of cyclic sulfamides from conjugated dienes. *Org. Lett*. **15**, 796−799 (2013).
6. Zhang, B., Wang, H., Lin, G.-Q. & Xu, M.-H. Ruthenium(II)-catalyzed asymmetric transfer hydrogenation using unsymmetrical vicinal diamine-based ligands: dramatic substituent effect on catalyst efficiency. *Eur. J. Org. Chem*. **2011**, 4205−4211 (2011).
